# Dataset on ethical leadership and corporate reputation – Nigerian deposit money banks׳ perspective

**DOI:** 10.1016/j.dib.2018.05.094

**Published:** 2018-05-23

**Authors:** Olorunfemi Adebisi Onakoya, Chinonye Love Moses, Oluwole Oladele Iyiola, Odunayo Paul Salau, Ezekiel Omisade Ayoade

**Affiliations:** Department of Business Management, Covenant University, Ota, Ogun-State, Nigeria

**Keywords:** Ethical leadership, Corporate reputation, Nigerian deposit money banks, CEO׳s ethics, Ethical culture, Ethical programs

## Abstract

Banking institutions play a critical role in any economy, and their stability is crucial to the economic development of a nation. The wave of corporate scandals that rocked the industry left the public with a loss of confidence. Efforts have since been channeled by banks towards developing their corporate governance mechanisms, except that the aspect of ethical leadership and how it translates to a bank׳s corporate reputation has not received sufficient attention. The dataset presented the perception of employees in selected deposit money banks in Nigeria. A multistage sampling technique was used to elicit data from the employees. Inferential statistics such as correlation, and regression analysis were adopted. The data collected focused on the impact of ethical leadership on corporate reputation. It also provided information on the significant factors affecting ethical leadership as well as the measures of corporate reputation. The survey data when analysed can be a pointer in determining the unique ethical leadership predictors that could enhance a bank׳s reputation.

**Specifications Table**

**Table:** 

Subject area	*Business Management*
More specific subject area	*Corporate Governance*
Type of data	*Tables and figures*
How data was acquired	*Field Survey (Questionnaire)*
Data format	*Raw, analyzed*
Experimental factors	*Proportionate, stratified, and purposive sampling of bank employees across all grades in eight selected deposit money banks in Lagos, Nigeria*
Experimental features	*Descriptive and inferential statistics*
Data source location	*Lagos, Nigeria*
Data accessibility	*Data are attached to this article*

**Value of the data**

• Dearth of empirical studies on ethics management in Nigeria.
• Data describes perception of the employees on the personal ethics of CEOs, ethical programs, ethical culture, and corporate reputation in their organisations.
• Dataset can be used to explore other research interests such as ethical leadership as a predictor of financial performance, corporate social performance, and earnings management among others.
• Dataset can be used by academia, bank practitioners, and regulatory authorities to evaluate ethics management in banks.
• The dataset can also be used to identify specific ethical leadership factors as predictors of corporate reputation

## 1 Data

**Fig. 1:**
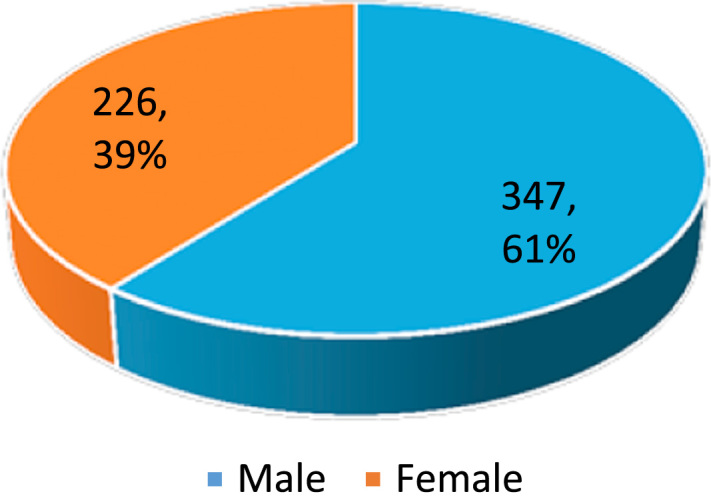
Gender distribution of respondents.

Source: Researcher׳s Field Survey (2017).

**Fig. 2:**
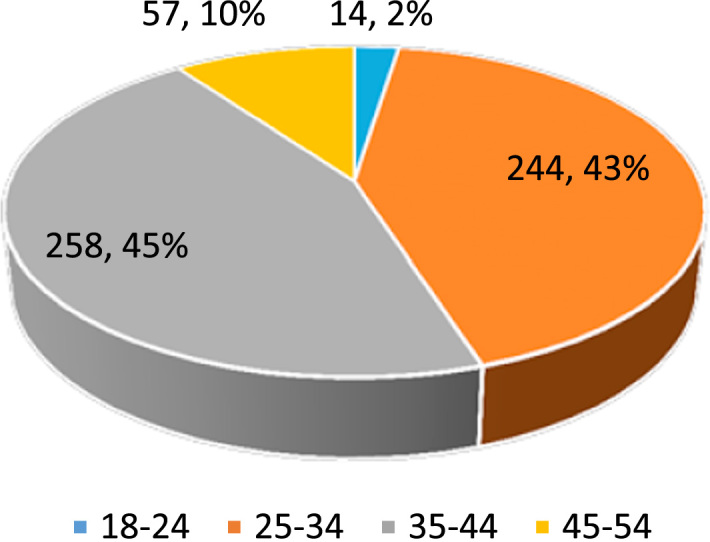
Age distribution of respondents.

Source: Researcher׳s Field Survey (2017).

**Fig. 3:**
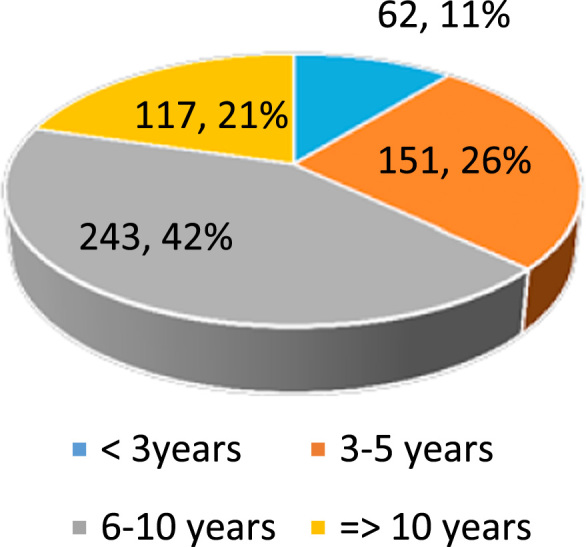
Years spent on job by respondents.

Source: Researcher׳s Field Survey (2017).

**Fig. 4:**
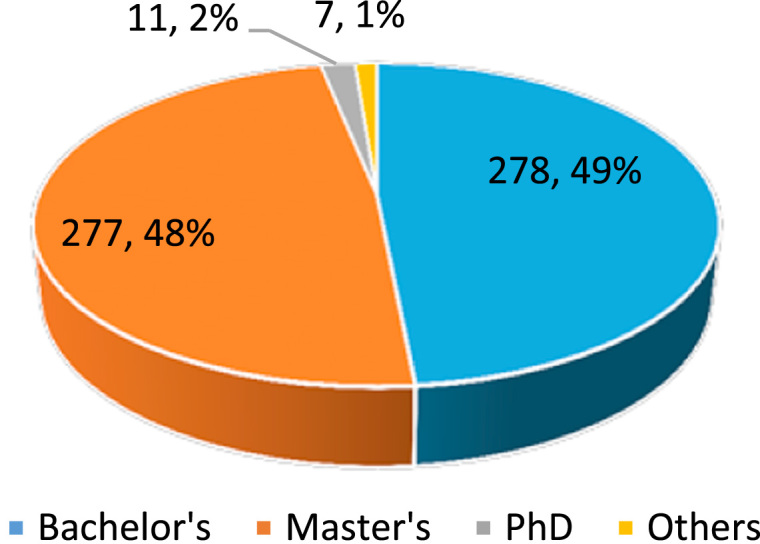
Highest education distribution of respondents.

Source: Researcher׳s Field Survey (2017).

Dataset provides raw descriptive and inferential statistics on the relationship between Ethical Leadership and Corporate Reputation. Fig. 1, Fig. 2, Fig. 3, Fig. 4 provide data on selected characteristics of the sample, such as gender (Fig. 1), age (Fig. 2), years spent on the job (Fig. 3) and highest education level attained (Fig. 4). Table 1 provides the validity test results on the research instrument (questionnaire), Table 2 provides data on correlations for the variables used in the empirical analysis, and Table 3 provides data on the estimates of the regression specification: *Corporate reputation=f (CEO ethics, Ethical Culture, Ethical Programs).*

**Table 1:** Validity test: Ethical leadership and corporate reputation.

**Measurement**	**Loading**	**Indicator reliability**	**Error**	**Compose reliability**	**Ave. variance**
			**variance**		**estimated**
	**≥0.7**		**≤0.5**	**≥0.8**	**≥0.5**
**Ethical leadership**					
Moral	0.7777	0.6048	0.3952	0.9443	0.753
Empathy	0.8522	0.7262	0.2738		
Fairness	0.7228	0.5224	0.4776		
Conflicts	0.8442	0.7127	0.2873		
Long term	0.6977	0.4868	0.5132		
Trust	0.7849	0.6161	0.3839		
Fairness	0.7652	0.5855	0.4145		
Tone	0.6859	0.4705	0.5295		
Reward	0.7666	0.5877	0.4123		
Doc Codes	0.6854	0.4698	0.5302		
Eth Officer	0.7643	0.5842	0.4158		
Train Prog	0.6889	0.4746	0.5254		
Auth	0.7281	0.5301	0.4699		
**Corporate reputation**					
Product Quality	0.8817	0.7774	0.2226	0.9038	0.8707
Financial Soundness	0.8526	0.7269	0.2731		
Global Competitiveness	0.8775	0.77	0.23		

All loadings in Table ** are significant at *p*<0.0001.

**Table 2:** Correlation: Ethical leadership and corporate reputation.

Variable		CEO_Ethics	Eth_Cul	Eth_Prog	Eth_Lead	Corp_Rep
CEO_Ethics	Pearson Correlation	1				
	Sig. (2-tailed)					
Eth_Cul	Pearson Correlation	0.655**	1			
	Sig. (2-tailed)	0.000				
Eth_Prog	Pearson Correlation	0.263**	0.393**	1		
	Sig. (2-tailed)	0.000	0.000			
Eth_Lead	Pearson Correlation	0.829**	0.851**	0.689**	1	
	Sig. (2-tailed)	0.000	0.000	0.000		
Corp_Rep	Pearson Correlation	0.230**	0.324**	0.570**	0.468**	1
	Sig. (2-tailed)	0.000	0.000	0.000	0.000	

** Correlation is significant at the 0.01 level (2-tailed).

**Table 3:** Regression - Influence of Ethical Leadership on Corporate Reputation.

	B	β	Std. Error	*t*-test	*p* value
(Constant)	0.918		.203	4.528	*p* = .000
CEO Ethics	0.026	.026	.047	0.564	*p* = .573
Ethical Culture	0.132	.102	.061	2.149	*p* = .032
Ethical Program	0.613	.523	.043	14.095	*p* = .000
Model Summary: R = .584, R^2^ = .337, Adjusted R^2^ = .334, *F* (3, 569) = 48.824, *p* = .000					

Dependent Variable: Corporate Reputation; Std error- Standard error; B= Unstandardized co-efficient; β = Beta, p value = significance value

**Source:** Researcher’s Field Survey (2017)

## 2 Experimental design, materials and methods

The dataset presented focuses on the influence of ethical leadership on corporate reputation of selected Deposit Money Banks in Nigeria. Data was gathered from employees in selected Deposit Money Banks with the aid of a close-ended questionnaire designed by the authors, based on the works of Refs. [1], [2], [3], [4]. We got responses from five hundred and seventy three (573) participants who duly completed the administered questionnaire. Responses from the questionnaire were extracted into Microsoft Excel, and subsequently coded and inputted into Statistical Package for the Social Sciences (SPSS) Version 22. We performed data analysis by applying inferential statistical tests which involved multiple regression analysis and descriptive analysis. The study population comprised of fourteen-thousand-one-hundred-and- forty-seven (14,147) employees based in the Lagos offices of the eight (8) selected banks, whilst the sample size obtained through the sample size calculator was seven hundred and forty-three (743) employees. Five (5) of the eight (8) banks accounted for 53.7% of the total deposit base, and 53.68% of total asset base of all Deposit Money Banks in Nigeria as at 31 December 2016 [10]. A multi-stage sampling technique involving proportional-to-size, stratified and purposive sampling was adopted for the study. Lagos was chosen as the scope of study because seventy-percent (70%) of commercial activities takes place in Lagos-State, whilst all the selected banks have their head-quarters in Lagos-State. The questionnaire was self-administered to the respondents who voluntarily completed the research instrument. Ethical issues such as prior consent, anonymity, and confidentiality of respondents among others were taken into consideration. The authors established that the respondents were knowledgeable about the background, purpose, and study variables of this research.

The study identified three (3) key measures of ethical leadership from literature [1], [2], [3], [4], [5], and three indicators of corporate reputation [6], [7], [8]. The population comprised of employees from the level of trainee to executive director, and all the stratified job functions in the selected banks under consideration. A cross-sectional survey design using a questionnaire instrument was used to elicit data from the respondents. Similar works that have used field survey instrument to obtain data can be found in works by [1], [9]. The questionnaire was divided into three parts; demographic variables, ethical leadership factors and corporate reputation indicators. Demographic factors reported include respondents׳ gender, age, marital status, highest education level, job function, job position, and years spent on the job. A five-point Likert scale of equal interval (ranging from strongly disagree (1) to strongly agree (5)) was used as the measure of responses. The data was analyzed by multiple regression using the Statistical Package for the Social Sciences (SPSS) software Version 22. The dataset is useful for bank managers to understand the key factors required to enhance ethical leadership and consequently their firms’ corporate reputation.

Table 3 above presents estimates of the analysed data based on the model specification. Estimates show that ethical culture and ethical programs have positive significant influence on corporate reputation, while CEO׳s ethics show a positive but non-significant effect on corporate reputation. The dataset provides useful insights for bank practitioners, regulators and other stakeholders to understand the role of different measures of ethical leadership in influencing corporate reputation.
