# Supplementary material for: Dataset on ethical leadership and corporate reputation – Nigerian deposit money banks׳ perspective
Source: Data Brief. 2018 May 23;19:847–52. doi: 10.1016/j.dib.2018.05.094 (PMC5997921; doi:10.1016/j.dib.2018.05.094)
Supplement: Supplementary file 2 — Supplementary material [file mmc2.pdf]

# CORPORATE GOVERNANCE DIMENSIONS AND SUSTAINABILITY: A STUDY OF SELECTED DEPOSIT MONEY BANKS IN NIGERIA


**ONAKOYA, OLORUNFEMI ADEBISI**

**Matriculation Number: 14PAB00805**


**MARCH, 2018**

# CORPORATE GOVERNANCE DIMENSIONS AND SUSTAINABILITY: A STUDY OF SELECTED DEPOSIT MONEY BANKS IN NIGERIA

**ONAKOYA, OLORUNFEMI ADEBISI**
B. Sc, MBA, FCA
(Matriculation Number: 14PAB00805)

**A THESIS SUBMITTED TO THE DEPARTMENT OF BUSINESS MANAGEMENT, COLLEGE OF BUSINESS AND SOCIAL SCIENCES, COVENANT UNIVERSITY, OTA, OGUN STATE, NIGERIA IN PARTIAL FULFILMENT OF THE REQUIREMENTS FOR THE AWARD OF DOCTOR OF PHILOSOPHY (Ph.D) DEGREE IN BUSINESS ADMINISTRATION**

**MARCH, 2018**

## Acceptance

This is to attest that this thesis is accepted in partial fulfilment of the requirements for the award of the degree of the Doctor of Philosophy in Business Administration in the Department of Business Management, College of Business and Social Sciences, Covenant University, Ota, Ogun State, Nigeria.

Mr. Philip John Ainwokhai …………………………………..
Secretary, School of Postgraduate Studies Signature & Date

Professor Samuel Wara ………………………………
Dean, School of Postgraduate Studies Signature & Date

## Certification

We certify that the thesis titled "Corporate Governance Dimensions and Sustainability: A Study of Selected Deposit Money Banks in Nigeria" is an original work conducted by ONAKOYA Olorunfemi Adebisi, (14PAB00805), of Business Administration Programme in the Department of Business Management, College of Business and Social Sciences, Covenant University, Canaanland, Ota, Ogun State, Nigeria.

We have examined the work and found it acceptable for the award of a degree of Doctor of Philosophy in Business Administration.

| | |
|---|---|
| Prof. Oladele, Patrick. O. | ……………………………….. |
| | Supervisor Signature & Date |
| Prof. Moses, Chinonye. L. | …………………………….…. |
| | Co-Supervisor Signature & Date |
| Dr. Iyiola, Oluwole O. | ……………………………….. |
| Head, Department Business Management | Signature & Date |
| Professor Alege, P.O. | ……………………………….. |
| Dean, College of Business and Social Sciences | Signature & Date |
| Professor Wara, Samuel | …………………………........ |
| Dean, School of Postgraduate Studies | Signature & Date |

# Declaration

I, **ONAKOYA Olorunfemi Adebisi**, (14PAB00805), declare that this research work was conducted by me under the supervision of Prof. Patrick O. Oladele and Prof. Chinonye L. Moses of the Department of Business Management, Covenant University, Ota, Ogun State. I attest that the thesis has not been presented either wholly or partly for the award of any degree elsewhere. All sources of data and scholarly information used in this thesis are duly acknowledged.

**ONAKOYA Olorunfemi Adebisi** ……………………………

Signature & Date

# Dedication

This thesis is dedicated to my Lord and Saviour, Jesus Christ the author and finisher of our faith whose strength is always made perfect in my weaknesses. I also dedicate this to my precious family, Toluwanimi, Iyanuolauwa, Ayokunle, and Olapeju Onakoya, the love of my life.

## Acknowledgements

I acknowledge and remain forever grateful to God Almighty for directing me to embark on this journey, and for keeping faithful to His words in seeing me through. Without Him, there would not have been this thesis.

My appreciation goes to the Chancellor of this life-transforming institution (Covenant University, Ota), Dr. David Oyedepo for receiving the divine mandate to raise a godly generation and running with it. Thank you, Sir, for providing our generation a platform to stand on the shoulders of giants.

I sincerely thank the management of the school under the able leadership of Professor Aaron Aderemi Atayero (The Vice Chancellor) and Professor Shalom Chinedu (Deputy Vice Chancellor). I deeply appreciate the support of Professor P.O. Alege (Dean, College of Business and Social Sciences), Professor Samuel Wara (Dean, School of Postgraduate Studies), Prof. Abiodun Adebayo (Sub-Dean, School of Postgraduate Studies), and Prof. Oluwole Iyiola (Head, Business Management Department).

My special appreciation goes to my supervisor and co-supervisor, Professor Patrick O. Oladele and Professor Chinonye Love Moses for their valuable contributions, support, and encouragement in achieving the success of this work. The Lord will bless you both richly and continually enlarge your coast (Amen).

Equally, I wish to express my gratitude to many other academics whose inputs and contributions made this work a success. I want to specially thank Dr. Oluwole Iyiola, Prof. Rowland Worlu, Dr. Oladele Kehinde, Prof. David Akinnusi, Dr. Kayode Aweda, Dr. Leke Ogunnaike, Dr. Omotayo Osibanjo, Dr. Anthonia Adeniji, Dr. Odunayo Salau, Dr. Hezekiah Falola, Dr. Steve Ibidunni, Dr. Olabode Oyewunmi, Dr. Adebukola Oyewunmi, Dr. Maxwell Olokundun, and Dr. Mayowa Agboola. I thank my examiners, Dr. Abiola Babajide, Dr. Ebenezer Bowale, and Dr. Jonathan Odukoya; and my colleagues on this adventure Fatai Lawal, Ezekiel Ayoade, Deborah Motilewa, Akeem Taiwo, Bolaji Olaoye, Bisi Ibidunni, and Uche Okorie.

I place on record my appreciation to every resource person who facilitated my fieldwork – Taiwo Urhue-Duke, Isioma Gogo-Anazodo, Tony Ibikunle, Dele Dopemu, Moshood Adelotan, Amara

Okogbue, Bisi Adeyemi, Malachy Anakwe, Joshua Ohioma, Awele Ajibola, David Adesanmi, Damilola Oluwaniyi, Tomisona Adegunle, Boluwatife Ogunojuwo, my questionnaire respondents (too many to list), my interviewees (whose identities have to be protected in line with ethical consideration), and others too numerous to mention.

Finally, to a very special people, my family network support group, for providing the moral support and prayer: Dr. Bunmi Aluko, Rev. & Rev. (Mrs) Aigbogun, Rev. & Rev. (Mrs) Isaac Oni, Olaolu Atokileso, Abolade and Itunu Ajayi, and for the MVP award winners, Toluwanimi, Iyanuoluwa, and Ayokunle Onakoya for their tremendous understanding coping with an almost-absentee dad, and to the love of my life, Olapeju Onakoya for being there for me through the years. The Lord shall reward you beyond imagination.

Thank you.

# Table of Contents


| | | Page |
|---|---|---|
| Cover Page | | |
| Title Page | | i |
| Acceptance Page | | i i |
| Certification | | iii |
| Declaration | | iv |
| Dedication | | v |
| Acknowledgements | | vi |
| Table of Contents | | viii |
| List of Tables | | xi |
| List of Figures | | xiv |
| List of Appendices | | xv |
| List of Abbreviations | | xvi |
| Abstract | | xvii |
| **CHAPTER ONE: INTRODUCTION** | | |
| 1.1 | Background to the Study | 1 |
| 1.2 | Statement of the Research Problem | 3 |
| 1.3 | Research Objectives | 6 |
| 1.4 | Research Questions | 7 |
| 1.5 | Research Hypotheses | 7 |
| 1.6 | Significance of the Study | 8 |
| 1.7 | Scope of the Study | 10 |
| 1.8 | Operationalisation of Variables and Model Specification | 11 |
| 1.9 | Schematic Model | 12 |
| 1.10 | Operational Definition of Terms | 13 |
| **CHAPTER TWO: LITERATURE REVIEW** | | |
| 2.1 | Conceptual Framework | 15 |
| 2.1.1 | Corporate Governance: Approaches and Definitions | 15 |
| 2.1.2 | Sustainability: An Overview | 17 |
| 2.1.3 | History of Corporate Governance | 18 |
| 2.1.4 | Global Corporate Governance Principles | 20 |
| 2.1.5 | Corporate Governance Practices Worldwide: Selected Countries and Models | 26 |
| 2.1.6 | Governance Value Models | 27 |
| 2.1.7 | Legislative and Institutional Framework | 30 |
| 2.1.8 | Status of Corporate Governance in Nigeria | 33 |
| 2.1.9 | Nigeria's Financial System | 36 |
| 2.1.10 | Nigeria's Banking Industry | 36 |
| 2.1.11 | Nigeria Deposit Insurance Company (NDIC) | 40 |
| 2.1.12 | Governance in the Banking Industry | 40 |
| 2.1.13 | Banking Supervision – Emergence of BASEL Capital Accord | 43 |
| 2.1.14 | Framework for Corporate Governance | 45 |
| 2.1.15 | Framework for Sustainability | 65 |
| 2.1.16 | Corporate Governance Dimensions and Sustainability: A Conceptual Perspective | 76 |

2.2 Theoretical Literature 84
2.2.1 Agency Theory 84
2.2.2 The Stakeholder theory 87
2.2.3 Institutional Theory 90
2.2.4 Theoretical Framework 92
2.3 Empirical Framework 94
2.3.1 Gaps in Literature Reviewed 106

**CHAPTER THREE: METHODOLOGY**

3.1 Research Design 111
3.2 Population of the Study 112
3.3 Sample Size Determination 113
3.4 Sample Frame 114
3.5 Sampling Technique 114
3.6 Method and Sources of Data Collection 115
3.7 Measurement of the Research Variables 117
3.7.1 Construction of Index – Corporate Governance and Sustainability Indices 120
3.8 Design of Research Instrument 125
3.8.1 The Questionnaire Survey 126
3.8.2 The Semi-Structured Interview 126
3.8.3 Content Analysis 126
3.9 Pre-distribution of Research Instruments –Pilot Testing 127
3.10 Validity of Research Instrument 127
3.11 Reliability of Research Instrument 130
3.12 Methods of Data Analysis 131
3.12.1 Quantitative Data 131
3.12.2 Qualitative Data 133
3.13 Ethical Considerations 133

**CHAPTER FOUR: DATA PRESENTATION AND ANALYSIS**

4.1 Data Presentation 134
4.2 Demographic Characteristics of Respondents 135
4.3 Descriptive Statistics on Classification of Research Variables 139
4.3.1 Descriptive Statistics of responses on Independent Variables 140
4.3.2 Descriptive Statistics of responses on Dependent Variables 148
4.4 Test of Hypotheses 155
4.4.1 Test of Hypothesis One 155
4.4.2 Test of Hypothesis Two 160
4.4.3 Test of Hypothesis Three 165
4.4.4 Test of Hypothesis Four 173
4.4.5 Test of Hypothesis Five 187
4.4.6 Test of Hypothesis Six 196
4.5 Qualitative Findings Based on Thematic Analysis 213
4.5.1 Theme 1: The Role of the Board 214
4.5.2 Theme 2: Ownership Structure and Shareholders’ Rights 216

4.5.3 Theme 3: The Role of Executives 218
4.5.4 Theme 4: Risk Management 218
4.5.5 Theme 5: Ethics 219
4.5.6 Theme 6: Strategy 220
4.5.7 Theme 7: Formal and Informal Institutions 220
4.5.8 Theme 8: Performance and Effectiveness 224
4.5.9 Theme 9: Sustainability and Corporate Reputation 224

**CHAPTER FIVE: DISCUSSION OF FINDINGS**

5.1 Discussion of Theoretical Findings 229
5.2 Discussion of Empirical Findings 230

**CHAPTER SIX: CONCLUSION AND RECOMMENDATIONS**

6.1 Summary of the Study 249
6.2 Conclusion 252
6.3 Recommendations 253
6.4 Contributions to Knowledge 257
6.5 Limitations of the study 259
6.6 Suggestions for Further Studies 260
7.0 References 262
8.0 Appendices 298

# List of Tables


| | | |
|---|---|---|
| 2.1 | Review of Empirical Findings | 95 |
| 3.1a | Selected Banks' Staff Strength and Branch Network | 112 |
| 3.1b | Study population - Lagos Branches | 113 |
| 3.2 | Allocation of copies of questionnaire | 115 |
| 3.3 | Measurement of Research Variables | 118 |
| 3.4a | Sample Scoring Guide on Environmental Sustainability | 122 |
| 3.4b | Scoring Guide on Financial Performance | 123 |
| 3.5 | Sustainability Reporting Index Measuring Scale | 124 |
| 3.6 | Corporate Governance Practices Disclosure Index Measuring Scale | 124 |
| 3.7 | Measurement of financial performance | 125 |
| 3.8 | Validity Test | 127 |
| 3.9 | Cronbach alpha statistics interpretation | 130 |
| 4.1 | Breakdown (in Aggregate) of Questionnaire Distribution and Retrieval | 134 |
| 4.2 | Demographic Characteristics of Respondents | 135 |
| 4.2.1 | Code for sampled banks | 137 |
| 4.3a | Descriptive Statistics Internal Governance Controls – Board of Directors | 140 |
| 4.3b | Descriptive Statistics Internal Governance Controls – Management | 141 |
| 4.3c | Descriptive Statistics Internal Governance Controls – Ownership | 142 |
| 4.4 | Descriptive Statistics Regulation | 143 |
| 4.5a | Descriptive Statistics Ethical leadership – CEO's Personal Ethics | 145 |
| 4.5b | Descriptive Statistics Ethical leadership – Ethical Programmes | 146 |
| 4.5c | Descriptive Statistics Ethical leadership –Ethical Culture | 147 |
| 4.6a | Descriptive Statistics Corporate Social Performance –Employees | 148 |
| 4.6b | Descriptive Statistics Corporate Social Performance –Customers | 149 |
| 4.6c | Descriptive Statistics Corporate Social Performance – Community | 150 |
| 4.6d | Descriptive Statistics Corporate Social Performance –Environmental Performance | 151 |
| 4.7 | Descriptive Statistics Financial Innovation | 152 |
| 4.8 | Descriptive Statistics Corporate Reputation | 153 |
| 4.9 | Descriptive Statistics Financial Performance | 154 |
| 4.10 | Correlation Matrix Internal Governance Controls and Corporate Social Performance | 156 |

| | | |
|---|---|---|
| 4.11 | Model Summary -Internal Governance Controls and CSP | 157 |
| 4.12 | ANOVA - Internal Governance Controls and CSP | 158 |
| 4.13 | Coefficients - Internal Governance Controls and CSP | 158 |
| 4.14 | Correlation Coefficient - Ethical leadership and Corporate Reputation | 161 |
| 4.15 | Model Summary -Ethical leadership and Corporate Reputation | 162 |
| 4.16 | ANOVA-Ethical leadership and Corporate Reputation | 163 |
| 4.17 | Coefficients-Ethical leadership and Corporate Reputation | 163 |
| 4.18 | Correlation Coefficient - Regulation and Financial Innovation | 166 |
| 4.19 | Model Summary-Regulation and Financial Innovation | 167 |
| 4.20 | ANOVA-Regulation and Financial Innovation | 167 |
| 4.21 | Coefficients-Regulation and Financial Innovation | 168 |
| 4.22 | Model fit index - Hypothesis 1-3 | 170 |
| 4.23 | Standardized Regression Weights | 171 |
| 4.24 | Descriptive Statistics -Agency Mechanisms and Financial Performance | 174 |
| 4.25 | Source of Data for Agency Mechanisms | 175 |
| 4.26 | Correlation Matrix and Variance Inflation Factors | 176 |
| 4.27a | Dependent Variable: LROA (Hypothesis 4) | 178 |
| 4.27b | Dependent Variable: LROE (Hypothesis 4) | 180 |
| 4.27c | Dependent Variable: LTBQ (Hypothesis 4) | 182 |
| 4.27d | Dependent Variable: LNIM (Hypothesis 4) | 184 |
| 4.27e | Dependent Variable: LNPL (Hypothesis 4) | 185 |
| 4.28 | Summary of Significance Tests on Financial Performance | 187 |
| 4.29 | Descriptive Statistics -Bank Characteristics | 188 |
| 4.30a | Dependent Variable: LROA (Hypothesis 5) | 190 |
| 4.30b | Dependent Variable: LROE (Hypothesis 5) | 191 |
| 4.30c | Dependent Variable: LTBQ (Hypothesis 5) | 192 |
| 4.30d | Dependent Variable: LNIM (Hypothesis 5) | 193 |
| 4.30e | Dependent Variable: LNPL (Hypothesis 5) | 195 |
| 4.31 | Summary of Significance Tests - Bank characteristics | 196 |
| 4.32 | Descriptive Statistics on Governance and Sustainability Index | 197 |
| 4.33 | Customers' Complaints - Volume and Value (2016) | 204 |

4.34 Correlation Coefficient of Corporate Governance Practices Disclosure and Sustainability Reporting 210
4.35 Model Summary - Governance Practices Disclosure and Sustainability Reporting 211
4.36 ANOVA-Corporate Governance Practices Disclosure and Sustainability Reporting 211
4.37 Coefficients-Corporate Governance Practices Disclosure and Sustainability Reporting 212
4.38 Rejection or Acceptance of the Null hypotheses 212
4.39 Interviewee Profile 213
4.4 Emergent themes and Sub-themes from Interview Respondents 213

## List of Figures


| | | |
|---|---|---|
| 1.1 | Schematic Model of the Study | 13 |
| 2.1 | Corporate Governance Framework - Agency Theory | 86 |
| 2.2 | Corporate Governance Framework - Stakeholder Theory | 89 |
| 2.3 | Corporate Governance Framework - Institutional Theory | 92 |
| 4.1 | Model Fit – Hypothesis 1-3 | 171 |
| 4.2 | Corporate Governance Practices Disclosure | 198 |
| 4.3 | Sustainability Performance Reporting Index | 199 |
| 4.4a | Economic Performance | 200 |
| 4.4b | Social Performance | 202 |
| 4.4c | Environmental Performance | 206 |
| 6.1 | Corporate governance – sustainability Model | 259 |

# List of Appendices


| | | |
|---|---|---|
| A | Questionnaire | 290 |
| B | Interview Questions | 295 |
| C | Hausman Tests – Agency Mechanisms on Financial Performance | 296 |
| D | Criteria for Selecting Study Population | 307 |
| E | Corporate Governance Practices Disclosure Index | 308 |
| F | Sustainability Performance Reporting Index | 312 |
| G | Sensitivity Analysis – Agency Mechanisms on Financial Performance | 317 |
| H | Further Analysis –Internal Governance Controls and Corporate Social Performance | |

# List of Abbreviations

| | |
|---|---|
| **CBN** | Central Bank of Nigeria |
| **IFRS** | International Financial Reporting Standards |
| **NDIC** | Nigeria Deposit Insurance Corporation |
| **CAR** | Capital Adequacy Ratio |
| **ROA** | Return on Asset |
| **ROE** | Return on Equity |
| **NIM** | Net Interest Margin |
| **MBV** | Market-to-Book Value |
| **NPLR** | Non-Performing Loan Ratio |
| **WBCSD** | World Business Council for Sustainable Development |
| **SEC** | Securities and Exchange Commission |
| **FRCN** | Financial Reporting Council of Nigeria |
| **ICAN** | Institute of Chartered Accountants of Nigeria |
| **OECD** | Organisation for Economic Co-operation and Development |
| **BCCI** | Bank of Credit and Commerce International |
| **CAMA** | Companies and Allied Matters Act |
| **ISA** | Investment and Securities Act |
| **BOFIA** | Banks and Other Financial Institutions Act |
| **MNC** | Multinational Corporations |
| **PENCOM** | National Pension Commission |
| **NAICOM** | National Insurance Commission |
| **AMCON** | Asset Management Corporation of Nigeria |
| **NSE** | Nigerian Stock Exchange |
| **BVN** | Bank Verification Number |
| **TSA** | Treasury Single Account |
| **NBA** | Nigerian Bar Association |
| **NMA** | Nigerian Medical Association |
| **COREN** | Council for the Regulation of Engineering in Nigeria |
| **EFCC** | Economic and Financial Crimes Commission |

## Abstract

There is an undeniable nexus between corporate governance and the sustainability indices of structured business entities; but the verifiable contributions of this quantitative and qualitative interaction are subject to contextual variances. This study examined the influence of corporate governance dimensions on sustainability within selected deposit money banks operating in Nigeria. Specifically, the study investigated the effects of internal governance controls on banks' corporate social performance; analysed the influence of ethical leadership on corporate reputation; evaluated the role of regulation on financial innovation; determined the influence of agency mechanisms on financial performance, the role of bank characteristics in moderating financial performance; and examined the contributions of corporate governance practices disclosure on banks' sustainability performance reporting. The study is underpinned by the agency and stakeholder theories and adopts a combination of mixed-method, ex-post facto, descriptive and inferential research design. Five hundred and seventy-three respondents (representing 74% of administered questionnaires), fifteen interviewees, composite index for 2013-2016, and secondary data for 2006-2016 formed the study instruments. A multi-stage sampling technique was adopted in choosing the samples from the eight-bank study population. Findings supported the six alternate hypotheses formulated in this study. Agency mechanisms such as board ethnicity and CEO's age significantly influence financial performance positively, while CEO's remuneration, insider ownership, and frequency of board meetings contributed negatively. Having female board members had negative association with financial performance and was only significant at 10% on Tobin's Q. Social and environmental performance of banks is considered weak. Thematic analysis of qualitative findings identified the importance of culture, ethics, and strategy in corporate governance. The study concludes that sustainability is achievable through an effective corporate governance framework anchored on a responsibility not only to shareholders but all relevant stakeholders. The study recommended legislation of female representation on the board; publication of pay ratios between CEO and average employee; minimum age of fifty-four (54) for CEOs; and capping insider share ownership at two percent (2%). Business opportunities exist for independent rating agencies; whilst it's imperative for the professional accounting body to develop accounting standards on sustainability performance reporting.



**Keywords:** *Regulation, Corporate governance Dimensions, Corporate reputation, Ethical leadership, Financial Innovation and Performance, Sustainability.*

# CHAPTER ONE

# INTRODUCTION

## 1.1 Background to the Study

The objectives of contemporary business organizations include growth, profitability and sustainability. However, only thirty-three percent (33%) of businesses successfully transit from first to second generation, and a little below 4.5% survive beyond the third generation (Forbes, 2013; White, 2006). Economic indices have substantial impact on every facet of national and organisational existence. This is a motivation for various countries and organisations to continually formulate and implement policies and practices aimed at promoting sustainable growth, specifically in terms of the development and management of business. However, in both developed and developing countries, there are diverse challenges with respect to ensuring a veritable balance in the governance matrix which underpins corporate activities. Issues synonymous with developed countries in this regard include, manipulative reporting standards (Baker and Anderson, 2010), breach of privacy and anti-trust concerns. In developing countries, there are underlying factors such as the separation of ownership and management, profit allocation, succession planning, equitable representation, transparency and disclosure practices (Aina, 2016).

The wave of global financial crisis that originated in Asia in 1997 and corporate scandals that rocked corporations such as Enron, World.com, Tyco and Parmalat in the first decade of the twenty-first (21st) century endangered the stability of the global economic system. This prompted questions about lack of accountability on the part of top executives, oversight by the board – and by extension corporate governance failure. The impact of the crises led to losses by stakeholders; criminal prosecution of executives, and bankruptcy filings (Monks and Minnow, 2008), prompting a loss of confidence by shareholders, businesses and the public in the financial industry (Angelides and Thomas, 2011). The scandals exposed the moral deficiency of the companies and their executives, leading to a more active awareness and

scrutiny of ethical issues in firms. Like many financial organisations around the world, Nigerian banks experienced the spill-over effects of the global crises (Ugwuanyi, 2014). The forced acquisition of several Nigerian banks such as Intercontinental bank, Oceanic bank among others is attributed to poor corporate governance which manifested in false disclosure of banks' financial positions as well as uneven regulatory supervision (Ajibo, 2015). The costs and impacts of corporate greed and misconduct arising from unethical practices and legal violation justify the need for business ethics. The extent to which business decisions reflect ethical values and principles is a key to long-term success, therefore the imperative for ethical behaviours and practices to be part of governance has arguably never been more important (Casson, 2013). This is even more applicable in a developing country with weak institutions, grappling with corruption, fraudulent dealing, and regulatory non-compliance among others. Corporate reputation therefore is a very valuable asset that needs to be jealously guarded by the board and a key responsibility of corporate governance. The regulators also have a critical role to play in serving as external monitor. In response, regulation and supervision have been enhanced both as a complement to the corporate governance of financial institutions and sometimes as a substitute for internal governance mechanisms (Ferrarini, 2017). The Central Bank of Nigeria (CBN) introduced reforms and initiatives such as bank consolidation, direct interventions; and adoption of International Financial Reporting Standards (IFRS) among others (Sanusi, 2012). Despite these interventions, Nigeria's banking sector is still grappling with several governance related issues such as continued deterioration in asset quality, financial innovation risks, unethical practices, and regulatory non-compliance as manifested through the Treasury Single Account (TSA) policy and Bank Verification Number (BVN) initiative (Oyewunmi, Olusanmi, Olujobi and Adegboye, 2017; CBN, 2015). Indeed, the regulators take the issue of bad loans seriously enough to rule that banks cannot pay dividends to shareholders if they have significant non-performing loans portfolio (The Vanguard Newspaper, 2018). This suffices to say that corporate governance as a whole and its attendant dimensions remain burning issues for Nigeria's banking sector (Adeoye and Amupitan, 2015).

Corporate governance offers several benefits as a strategic tool for competitive edge (Masouros, 2014). It enables low operating costs, attraction of valuable employees, as well as investors who are willing to pay a premium for a firm with good corporate governance

practices (Levine, 2005). It also promotes a firm's reputation, enhances economic performance, stimulates growth, and strengthens shareholders' confidence (Fanta, Kemal, and Waka, 2013). While internal corporate governance mechanisms may assist organisations in protecting and maximising shareholders' wealth, a banking institution owes obligation to several stakeholders such as depositors, employees, and the community among others. The primary objective of corporate governance should therefore be safeguarding an array of stakeholders' interests on a sustainable basis (Basel, 2015).

Sustainability according to the World Business Council for Sustainable Development (WBCSD, 2003) entails the coordination of an organisation's economic, social and environmental objectives in the delivery of its core business activities in order to maximise value. Whilst managements are held responsible for their short-term performances, their actions in meeting the corporate objectives may create problems for the future, which is the basis for the Sustainable Development Goals (SDGs) agenda. The external pressure to be better corporate citizens therefore increased especially during and after the financial crisis (Robins and Krosinsky, 2008; Yeoh, 2009) with firms demonstrating commitments to sustainable development (Baumgartner and Ebner, 2010). In 2013, the CBN in pursuit of the sustainable development agenda, introduced the principles of sustainable banking as a benchmark for banks in Nigeria. Achieving sustainability requires a business paradigm committed to addressing issues such as governance, regulation, and ethical leadership in order to gain a competitive edge through superior corporate social and financial performance, financial innovation, and enhanced corporate reputation (Amaeshi, 2017) in the light of the contextual dynamics in Nigerian banking sector.

The major preoccupation of this study in the light of the foregoing, is to investigate the influence of specific corporate governance dimensions (i.e. internal governance, ethical leadership, and regulation) on sustainability in selected deposit-money banks in Nigeria.

## 1.2 Statement of the Research Problem

Corporate governance offers several benefits leading to organisational sustainability. It has however been largely studied restrictively from the perspective of internal mechanisms (such as the board, management, ownership structure, and risk management), ignoring the

complementary role of external mechanisms (such as regulation), and stakeholders' management (such as ethical leadership, and governance practices disclosure). Individual governance mechanisms do not work in isolation but are often interrelated at different levels of analyses (Walls, Berrone, and Phan, 2012), hence the need to combine mechanisms to determine their efficiency, complementarity or substitutability (Aguilera, Desender and de Castro, 2012; Al-Baidhani, 2014). Studies have generally focused on financial performance as a proxy for firm performance (Peni, 2014; Ujunwa, 2012). The role of corporate governance in driving sustainability through promoting corporate social performance, corporate reputation, and financial innovation as measures of sustainability have not received sufficient attention in literature (Chardine-Baumann and Botta-Genoulaz, 2014). This study therefore seeks to remedy the imbalance by investigating the relationships amongst the dimensions of corporate governance (internal governance controls, ethical leadership, regulation, agency mechanisms and governance practices disclosure) and sustainability, i.e. economic (encompassing financial performance and financial innovation), social and environmental (comprising corporate social performance and corporate reputation), and sustainability performance reporting.

Internal governance controls have largely been concentrated on improving financial performance of banks, but stakeholder pressure and relations have an impact on the reputational risk and value of banks (Brown and Whysall, 2010; Evangelinos and Nikolaou, 2009). Social responsibility in Nigeria focuses on philanthropy, with less or no attention to socially responsible products and services, employee relations, as well as the protection of the environment (Amaeshi, Adi, Ogbechie and Amao, 2006). Whilst banks may not be directly impacting the environment substantially, their significant exposure to the oil and gas sector raises the risk of potential losses from borrowing firms vulnerable to practices that are not socially responsible (CBN, 2016). Banks have embraced the CBN's principles of sustainable banking, but there is a need to understand the driving force behind corporate social performance (i.e. social and environmental performance) if it is based on a mind-set, business strategy, or fear of regulatory penalty. Most studies (Wong and Wong, 2015; Akpan and Amran, 2014) on corporate social performance have focused on philanthropy, non-banks, or ignored the interaction effects of internal governance controls in enhancing corporate social performance.

CBN (2016) reports the increase in cases of frauds and forgeries, insider credits, other insider-related dealings, and customer complaints, leading to the sack of some bank boards and a further loss of confidence in the banking sector. Yidawi (2005) attributes most of the cases to insiders, whose activities have materially affected the reputation of banks. Thus the questions of ethics remain inherent in all aspects of corporate governance. Surveys (Weber Shandwick, 2015; Burson-Marsteller, 2003) have shown that forty-nine percent (49%) of a company's reputation is tied to the CEO, whilst corporate reputation confers competitive advantage through attraction of employees, customers, and investors, as well as allowing for premium prices (Deephouse 2000; Fombrun 1996). Despite the benefits and importance of ethical practices, only recently have managers and academics focused on ethics management (Ho, 2005; Trevino and Brown, 2005), and even at that, the available studies (Love, Lim and Bednar, 2017; Eisenbeiss, Knippenberg, and Fahrbach, 2015), have mostly been in developed countries, with few studies empirically linking ethical leadership to corporate reputation.

Banks engage in financial innovation to reduce cost, achieve efficiency, customer satisfaction, and ultimately to gain competitive edge in pursuit of sustainability (Frame and White, 2002). It is however a double-edged sword that could either lead to growth, or destruction in the industry – as was witnessed in 2008/9 when new products and services on mortgage were introduced by banks without proper risks-monitoring by the regulators (Trichet, 2009; Ferrarini, 2017). Regulators therefore have a critical role to play in serving as external corporate governance mechanism. Findings from regulation activities of the CBN revealed that the volume and value of electronic transaction has increased significantly, raising the issue of cybercrime, money-laundering, and terrorism financing as key operational risks resulting from financial innovation (CBN, 2016). Yet, most studies on regulation in corporate governance (Alam, 2012; Ugwuanyi, 2015) have been in relation to risk-taking, stability, and efficiency, or not empirically-based (Stewart, 2010; Racic, Cvijanovic and Aralica, 2007) when linked to financial innovation.

Agency mechanisms in corporate governance aim to maximise shareholders' value, and while profitability is the first line of defence for sustainability, it does not tell the full story. Most

studies on agency mechanisms either exclude financial institutions or combine them with firms from other industrial sectors. Others focus on only one or two corporate governance mechanisms as explanatory variables; or rely on sources (such as Compustat, Worldscope and Factbook) which provide 'tertiary', limited data. In addition, financial performance is mostly measured through traditional indicators such as Return on Asset/Equity (Irshad, 2015, Kajola, 2008) ignoring peculiar situations typified by the Nigerian banking sector on issues such as deteriorating non-performing loans, operational inefficiency, and a weak response to vagaries of macro-economic variables. Only limited studies (Akpan and Riman, 2012; Islam, 2014) have considered the importance of asset quality and efficiency as performance indicators.

Bank stakeholders want accountability. Corporate governance practices disclosure prompts transparency, which can only be achieved through sustainability reporting (WBCSD, 2003). This requires ensuring the integrity of the nested interdependency of the economic, social and environmental performance (Amaeshi, 2017), except that this objective is not coherently connected to facilitate better understanding of an organisation's progress towards sustainability (Bradford, Earp, Showalter and Williams, 2017). Sustainability reporting and sustainability performance are still limited and largely fragmented (Huang and Watson, 2015; Rao and Tilt, 2016). Few studies (Weber and Oni, 2015; Babalola and Adedipe, 2014) have conceptualised governance practices disclosure and sustainability reporting, yet there is a relative dearth of empirical studies that test hypotheses or otherwise provide a causative quantitative analysis of their relationship. This study therefore seeks to examine corporate governance dimensions in relation to sustainability in selected deposit-money banks in Nigeria.

## 1.3 Research Objectives

The broad objective of this study is to investigate the influence of corporate governance dimensions on the achievement of sustainability in selected deposit-money banks in Nigeria. The specific objectives of this study are to:

i. assess the effects of internal governance controls on corporate social performance in selected deposit-money banks in Nigeria.
ii. examine the extent to which ethical leadership influence corporate reputation.
iii. evaluate the role of regulation on financial innovation.

iv. ascertain the influence of agency mechanisms on financial performance
v. determine the role of bank characteristics in moderating the influence of agency mechanisms on financial performance
vi. evaluate the impact of corporate governance practices disclosure on banks' sustainability reporting.

## 1.4 Research Questions

Based on these objectives, the study addresses the following questions:

i. To what extent do internal governance controls influence corporate social performance of deposit-money banks in Nigeria?
ii. What effect does ethical leadership have on banks' corporate reputation?
iii. To what degree does regulation influence banks' financial innovation?
iv. In what way do agency mechanisms influence financial performance?
v. To what extent do bank characteristics moderate the influence of agency mechanisms on financial performance?
vi. To what extent does corporate governance practices disclosure enhance sustainability reporting by banks?

## 1.5 Research Hypotheses

**Hypothesis One:**

$H_0$: Internal governance controls do not have significant effect on corporate social performance in selected deposit-money banks in Nigeria.

**Hypothesis Two:**

$H_0$: Ethical leadership does not have significant effect on corporate reputation in selected deposit-money banks in Nigeria.

**Hypothesis Three:**

$H_0$: Regulation does not have significant effect on financial innovation in selected deposit-money banks in Nigeria.

**Hypothesis Four:**

$H_0$: Agency mechanisms do not have significant influence on financial performance.

**Hypothesis Five:**

$H_0$: Bank characteristics do not have significant influence in moderating the relationship between agency mechanisms and financial performance.

**Hypothesis Six:**

$H_0$: Corporate governance practices disclosure does not have significant effect on sustainability performance reporting.

## 1.6 Significance of the Study

This study is of significance to the following beneficiaries:

**Practitioners**

This study provides suggestions to boards, owners, and managers of banks to set up appropriate board committees, engage appropriate resource personnel, develop strategic partnerships, and formulate strategic plans and metrics to measure performance in corporate governance and sustainability indicators. It also provides a guide on the communication strategy required to achieve sustainability objectives. In addition, the understanding gained from this study aids the resolution of goal conflicts faced by (bank) managers and other stakeholders in an organisation. Study findings also help practitioners in implementing recommendations to strengthen corporate governance mechanisms with a view to influencing their sustainability.

**Regulators**

Relevant regulators in the industry include CBN, NDIC, Securities and Exchange Commission (SEC), Financial Reporting Council of Nigeria (FRCN), Institute of Chartered Accountants of Nigeria (ICAN) etc. Findings from the study are useful to regulators in implementing policy changes hindering financial innovation, and sustainability reporting. It also identifies training and recruitment gaps for regulators and supervisors; potential for a robust risk management framework to be developed; policy changes with regards to current voluntary sustainability reporting, restriction of activities by banks and the deposit insurance scheme; as well as rewards and sanctions framework for bank practitioners.

The study identified challenges for the professional accounting regulatory body, ICAN to develop accounting standards, an integrated sustainability report combining economic, social and environmental results; and provide education and training for users of financial statements.

In addition, the findings make it imperative for rating agencies to offer credible services on ratings of deposit money banks on corporate governance, financial innovation, corporate reputation, and social responsibility.

**Primary Stakeholders**

This study provides a checklist (under sustainability reporting) of banks' responsibilities to employees, customers, and the community among others. Stakeholders should use the checklist as a tool to evaluate the banks and consider appropriate action for banks performing below expectations.

**Government**

Findings from this study provide input to government/policy makers to design policies, promulgate laws (for instance on female representation on boards), and create enabling environment (such as relaxation of rules on portfolio investment, foreign exchange restrictions, intellectual property rights etc) for banks to engage in sustainable practices.

**Investors**

Investors attach great importance to the corporate governance practices of their would-be investee. It is the only assurance that their investments will not be expropriated or inefficiently utilised. This study is useful for investors to gain an understanding of some corporate governance practices in Nigeria's banking sector.

**Academia**

This study provides the academia several suggestions for further studies. In addition, the findings and contributions to knowledge provide a platform for others to build on. For instance, this study encourages other researchers to embark on a long study (say over a twenty-year period) of the outcome of sustainable practices in banks using an experimental research design.

**Public**

The general public benefits by understanding the concept and practices of corporate governance, and how it helps the banks in particular, and the society in general. The study on social responsibility (that is social and environmental performance) raises the consciousness of the society in holding firms (banks and non-banks) accountable for their social and environmental responsibilities. Non-Governmental Organisations (NGOs) may therefore be in a better position to take up causes in social and environmental demands.

## 1.7 Scope of the Study

Corporate governance mechanisms across industries present heterogeneous issues and results, whereas a focus on the banking industry overcomes this challenge because of the uniformed way banks report their financial information as guided by designated regulatory authorities. This therefore gives a better indicator of the effect of corporate governance practice. This study on corporate governance dimensions and sustainability, also recognised the critical importance of banking industry to economic development, source of external finance for other firms, and the systemic effect of bank failures on a nation's economy. Most importantly, the industry's regulatory authority (CBN) intensified the drive for good corporate governance after the consolidation of banks in 2006 with the introduction of Code of Corporate Governance, and the Sustainable Banking Principles in 2013. Year 2006 marked the beginning of the post-consolidated era in Nigeria's banking industry, and covering 2006-2016 will provide sufficient time span for data analysis.

The selected deposit money banks for the study were based on certain parameters (Verhoef and Hilsden, 2004; Yermack, 1996; Ntim, 2009). The evaluation criteria (in appendix "E") specify:

i. Being a publicly listed deposit money bank and in operation at the end of the consolidation exercise in 2005;
ii. Must have maintained their business name since 2005 till end of 2016;
iii. Have not been acquired or enjoyed CBN's intervention;
iv. Have their financial statements for the years of study (2006-2016) available in the public domain.

The selected banks include five (5) Tier-1 banks - Access Bank, First Bank, Guaranty Trust Bank (GT Bank), United Bank for Africa (UBA), and Zenith Bank; and three (3) Tier-2 banks - Diamond Bank, First City Monument Bank (FCMB), and Fidelity Bank. Lagos was the geographical research horizon for the study as it is regarded as the commercial headquarter of Nigeria with most of the deposit-money banks having their headquarters located in Lagos State. The head-office seats the executive management, divisional heads at top management level, and specialised functions.

## 1.8 Operationalisation of Variables and Model Specification

The operationalisation of variables and model specification for the study objectives is depicted below:

$Z = f(X)$ …………………..………………. (1)

$Z = f(Y)$ ………………..…………………. (2)

$Z = f(X) + f(Y)$……………………………. (3)

$Z = f(X+Y)$ …………..……………………. (4)

Where,

X = Independent variable

Y = Moderating variable

Z = Dependent variable

Substituting for X, Y and Z

Therefore,

X= Corporate Governance Dimensions (CGD)

Y= Bank Characteristics (BC)

Z= Sustainability (S)

The independent variable, Corporate Governance Dimensions (CGD), is represented by several subvariables, which are expressed as:

X = (x1, x2, x3, x4, x5… xn)

Where,

$X_1$ = Internal Governance Controls (IGC),

$X_2$ = Ethical Leadership (EL);

$X_3$ = Regulation (R),

$X_4$ = Agency Mechanisms (AM),

$X_5$ = Governance Practices Disclosure (GPD).

That is: CGD = (IGC, EL, R, AM, GPD)

The moderating variable of Bank Characteristics (BC), is a vector of several variables, where Y is represented by the expression Y = (y1, y2, y3, …, yn)

Where,

y1 = Bank Size (BS)

y2 = Bank Leverage (BL)

y3 = Bank Age (BA)

That is: BC = (BS, BL, BA)

The dependent variable of Sustainability (S), is a vector of several variables, where Z is represented by the expression Z = (z1, z2, z3, …, zn)

Where,

z1 = Corporate Social Performance (CSP)

z2 = Corporate Reputation (CR)

z3 = Financial Innovation (FI)

z4 = Financial performance (FP)

z5 = Sustainability Performance Reporting (SPR)

That is: S = (CSP, CR, FI, FP, SPR)

## 1.9 Schematic Model

This research examines the influence of corporate governance dimensions on sustainability in selected deposit money banks in Nigeria. It attempts to measure corporate governance dimensions based on the works of Jensen and Meckling, 1976; Fama and Jensen, 1983 on agency theory; Freeman, 1984; Donaldson and Preston, 1995; Hart, 1995; John and Senbet, 1998; and OECD, 2004, 2015 on stakeholders' theory; and Eisenbeiss, Knippenberg, and Fahrbach (2015) on ethical leadership.

Sustainability is conceptualised from the works of WBCSD (2003), and Elkington (1997) on "triple bottom line" metrics of economic, social and environmental performance, Fombrun (2002) on corporate reputation; and Arnaboldi and Rossignoli (2015) on financial innovation. The study is conceptualised to show the influence of corporate governance (dimensions) on sustainability. Based on the above, the framework for the researcher's schematic model is depicted below:

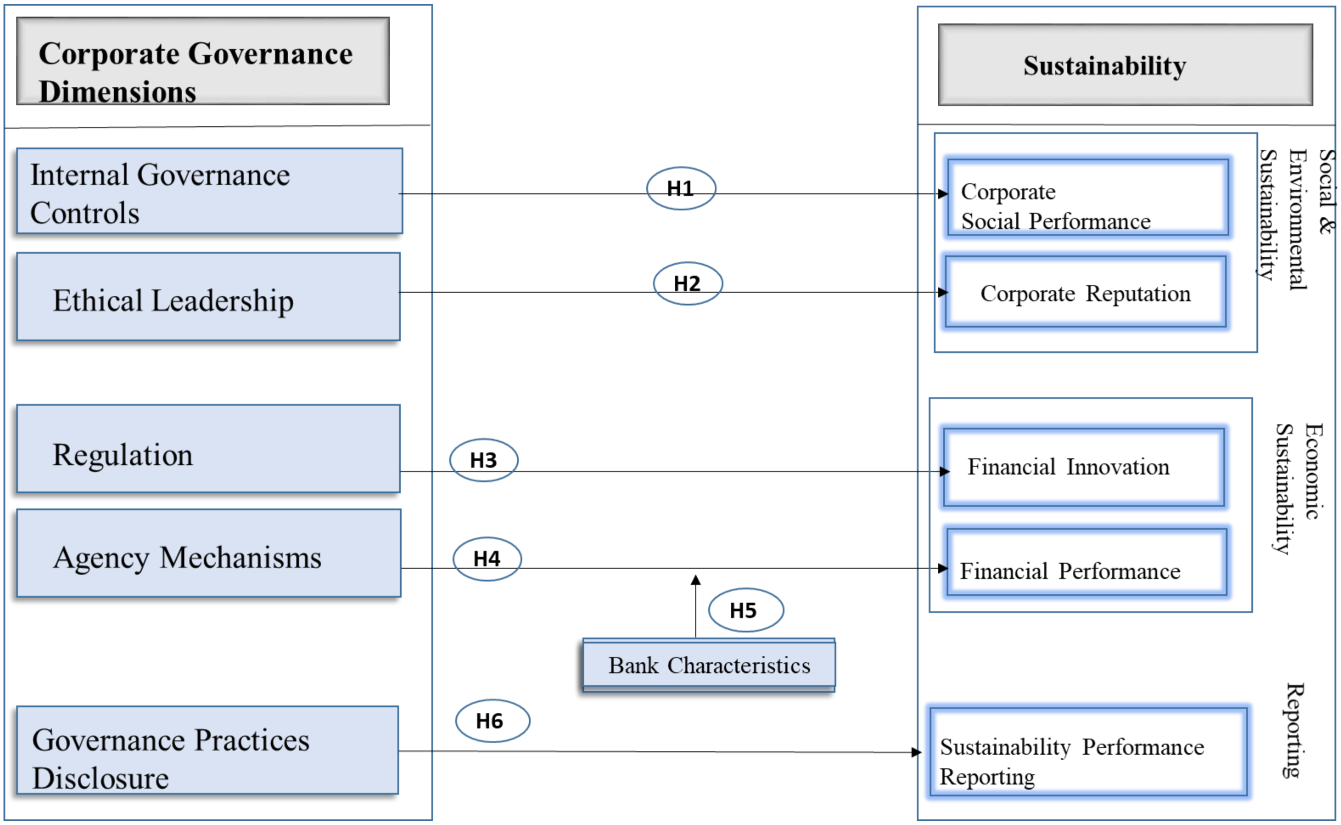


**Fig. 1.1: Schematic model of the study**

**Source:** ***Adapted from Elkington (1997), Freeman (1984), Jensen and Meckling (1976)***

## 1.10 Operational Definition of Terms

The following are contextual definitions as used by the author in this study:

**Corporate Governance**: a range of structures integrating regulatory governance, market governance, stakeholder governance and internal governance.

**Corporate Governance Dimensions** – Different aspects of corporate governance framework from different perspectives involving internal and external mechanisms, and stakeholder management.

**Regulation** - refers to the regulatory and supervisory activities of the central bank in monitoring the operations of the deposit money banks.

**Ethical leadership** – refers to a demonstration of ethical virtues by the CEO, the promotion of such conduct to employees and other stakeholders through bilateral communication, reinforcement, and decision.

**Sustainability** – a holistic measure of a bank's performance in economic, social and environmental categories with a view to business continuity.

**Financial Performance** – a measure of a bank's overall financial health over a given period of time as measured by accounting and market ratios.

**Corporate Social Performance –** Outcome of treating stakeholders in an ethical and responsible manner, encompassing an integration of social and environmental concerns in business operations.

**Post Consolidated-Era** – The period from 1 January 2006, when Nigerian banks just completed the consolidation programme, which entailed mergers and acquisitions, raising each bank's capital base from N2 billion to a minimum of N25 billion, and the reduction of the number of banks from 89 to 25 by 31 December, 2005.

**Deposit Money Banks -** A financial institution licensed by the regulatory authority to mobilise deposits from the surplus unit, channel the funds through loans to the deficit unit and perform other financial services activities.

**Financial Innovation –** A process that results in new financial products, financial markets, improved process efficiency, and delivery channels

**Corporate Reputation -**How an organisation is perceived by employees and management.

**Internal Governance Controls -** corporate governance mechanisms within the control of the firm, which include the board, management, and ownership structure.

**Agency Mechanisms** – Selected corporate governance mechanisms prescribed by the agency theory to maximise the shareholders' value.

# CHAPTER TWO

## LITERATURE REVIEW

### 2.0 Preamble

This chapter covers the conceptual, theoretical and empirical frameworks as well as the gap highlighted in literature relevant to this study.

### 2.1 Conceptual Framework

The conceptual framework discusses various concepts and constructs relevant to the stated objectives of the study.

#### 2.1.1 Corporate Governance: Approaches and Definitions

There are several definitions of corporate governance, with each definition narrowly or broadly defined to address a value orientation (the shareholder or stakeholder orientation); the evolution or developmental stage of corporate governance practice; or an expansion of scope to address emerging issues not clearly addressed by the extant definitions. In its narrow definition, corporate governance refers to the relationship between or amongst the key parties - owners, managers and the board, and in some cases other stakeholders. At the broad level, it extends to the issues of regulations and laws, as well as individual firms' practices to achieve economic, social and environmental objectives. Corporate governance definition also has a leaning towards the model in practice in individual countries. For instance, countries practising the Anglo-Saxon model are more favourably disposed towards a focus on the primacy of shareholders, whilst the Japanese and German models focus on the expectations of the society. The common ground in various definitions is a recognition of separate ownership of a corporation from the operational control. Hence, the need for independent systems to monitor, guide or control managerial opportunism. The following are some approaches to corporate governance:

**i. Shareholder-centric Approach**

Hess (1996) conceptualized corporate governance as the control and administration of a firm's resources for the benefit of the owners, while Sternberg (1998) defined it as ways of

ensuring resources are utilised to achieve the corporate objectives established by the shareholders. Similarly, Shleifer and Vishny (1997) attributed corporate governance to "ways in which suppliers of finance to corporations assure themselves of getting a return on their investment". La Porta, Lopez-de-Silanes, Shleifer and Vishny (2000) view it as a set of mechanisms through which outside investors protect themselves against expropriation by insiders. A narrow perspective by Lipton and Lorsch (1992) questioned the expansion of corporate governance to encompass society as a whole. They argued that this approach is flawed because management is not equipped to deal with issues of general public interest.

**ii. Stakeholder-centric Approach**

Sustentare-SAM (2010) defined corporate governance as a system which controls and manages company activities. This system consists of the policies, processes and people, which are necessary to meet the expectations of the company's shareholders and other stakeholders with integrity, respect, transparency and in a responsible manner. The Basel principles and the Organisation for Economic Co-operation and Development (OECD) share similar views on corporate governance as "a set of relationships between a company's management, its board, its shareholders and other stakeholders which provides the structure through which the objectives of the company are set, and the means of attaining those objectives and monitoring performance are determined" (OECD, 2015).

**iii. Institution-centric Approach**

Other definitions of corporate governance focus on structure and regulations. For instance, Monks and Minow (2008) define corporate governance as a non-abstract concept that is based on "setting up the right structure to make sure the right questions get asked and the right checks and balances are in place, to make sure that the answers reflect what is best for the creation of long-term sustainable value". Cadbury (1992) describes corporate governance as "the system by which companies are directed and controlled", and a broad definition of corporate governance being concerned with: what the purpose of the corporation is, in whose interest the corporation is run, who controls the corporation, and how the risks and returns from the governing activities are allocated (Blair, 1995). It is a structure established by firms to ensure the attainment of their long-term strategic goals, which encompasses managing the expectations of stakeholders and achieving social

responsibility through legal compliance and ethical behaviour (Gillibran, 2004). Corporate governance encourages the ethics of responsibility, fairness, straightforwardness, accountability, transparency, and obligation in corporations, thereby promoting sustainability. Therefore, the entrenchment of a sound corporate governance system in organisation is desirable as it contributes to the attainment of organisational objectives. Good corporate governance raises efficiency and growth, especially for countries that raise capital mainly through the stock market. At the firm level, organisations with good corporate governance create a competitive advantage (Carney, 2005), have access to cheaper funds than firms without, as well as enable management to allocate resources effectively, which leads to higher returns.

iv. **Governance Bundles Approach**

In recognition of the problems on mixed results on corporate governance studies, Ward, Brown and Rodriguez (2009) developed the 'governance bundles' approach which recognizes that effective governance requires an interplay of internal and external governance mechanisms, which through their interaction reveal the 'complementarity and, or substitutability' of the mechanisms in solving agency problems. Further work has been done on this approach by Aguilera, Desender and de Castro, 2012; and Al-Baidhani, 2014.

The definition adapted in this study is proffered by Sun, Stewart and Pollard (2012), which refers to corporate governance system as the 'whole set of regulatory, market, stakeholder and internal governance'. This definition is simple but holistic as it covers the interdependence of shareholders, other stakeholders, and institutions. It also recognizes the need for a combination of internal and external dimensions of corporate governance mechanisms, whilst underscoring the soft premise of ethicality in the interplay of actors within the governance system.

### 2.1.2 Sustainability: An Overview

Sustainability is described by IFC (2005) as ensuring long-term business success, while contributing to society's present and future social, environmental and economic needs. Wilson (2003) traced the history of sustainability to four main pillars - sustainable development, corporate social responsibility, stakeholder theory and accountability.

Sustainable development derived its origin from the work of Brundtland (1987), which refers to the concept as the “development that meets the needs of the present without compromising the ability of the future generations to meet their own needs”. Similar to this term, Corporate Social Responsibility (CSR) is a philosophical concept promoting the ethical responsibility of corporate managers to the needs of the society. Its central tenet holds that managers are not only obligated to protect shareholders or their own personal interests, but also those of the society. Stakeholder theory emerged as a strategic management concept which recognizes importance of the interdependence of several stakeholders in achieving a firm’s corporate objective. It is therefore imperative for managers as a business argument to build strong relationships with the internal and external stakeholders based on fairness, transparency, respect and cooperation. The final pillar derives from the ethical or legal responsibility of managers to render account of their stewardship to both the shareholders (who appointed them into office) and other stakeholders (who have contractual arrangements with the firm, such as customers, suppliers, employees, government etc.). At the forefront of accountability reporting are the works of Elkington (1997) who introduced the Triple Bottom Line (TBL) accounting system to expand the traditional reporting framework to take into account environmental and social performance in addition to financial performance. In a similar manner, Kaplan and Norton (1992) proposed the Balanced Scorecard (BSC) as a management tool which integrates financial and non-financial performance measures and builds a connection between organizational strategy and operational activity.

### 2.1.3 History of Corporate Governance

The history of what has emerged as corporate governance in the United States of America can be traced to developments in the late 70s, when corporation executives were found to have acted illegitimately by engaging in bribes and illegal payments to foreign officials, unknown to the outside directors (Cheffins, 2012). This led to introduction of regulations to guide the conduct of the corporations, starting with the issue of board composition and structure. Other challenges such as the passivism of the shareholders, and a political shift to the right however stalled the corporate governance reforms. Notwithstanding, the emergence of the institutional shareholders as power brokers stirred up interest in corporate governance once again. Due to

the rapid growth in their proportion of shareholding in public corporations, they were able to take on executive management perceived to be running public corporations poorly. They also introduced some changes, such as policy statements to evaluate directors and boards (Wilcox, 1997), replacement of poor performing directors, and a change in executive compensation structure (Dobbin and Zorn, 2005). As a reaction, management introduced anti-takeover measures such as “green mail” payments and “poison pills”, leading to a conflict between management and institutional shareholders. The judiciary often stepped-in for a resolution, which helped to promote the outside director as an important player in the corporate governance framework (Gordon, 2007). The role of the USA as a pioneer and model for other countries in corporate governance became threatened in the 1990s when the US economy faltered during the recession. Japanese and German companies emerged better-run, and more competitive with better performance, which was traced to the difference in emphasis placed on performance. Whilst US companies were faced with pressures to deliver short-term performance, their German and Japanese counterparts did not face such challenges because their corporate governance model was more concerned about long-term relational investment (Blair, 1995). This shows that there is a linkage between a country’s corporate governance model and its firms’ competitive strategy (Guillen, 2002).

The internationalisation of corporate governance spread to Britain in the early 1990s with the commissioning of a committee led by Sir Adrian Cadbury to review the financial aspects of corporate governance. This initiative coincided with the collapse of a number of British corporations, thereby signalled the lack of accountability on the part of top executives and lack of oversight on the part of the board (Cheffins, 1997). The Cadbury Committee went on to codify corporate governance principles for British companies to comply with or explain non-compliance (Cheffins, 1997). Thus, laying the foundation for universal adoption by more countries around the world. The Asian countries experienced their first major contact with corporate governance following the Asian market crash of 1997, which was attributed to the weak practices in family-owned organisations.

The boom and bust cycle in world economies tends to suppress or elevate corporate governance discourse respectively. By 2001, the financial crisis that engulfed the likes of Enron,

Worldcom, and Andersen led to the unanimity of opinion among stakeholders (such as investors, policy makers, executives) in ascribing the lack of accountability, poor board structure and shareholder passivism as the bedrock of “corporate governance” study. The relative importance of corporate governance study in recent history can then be traced to several factors. Hilb (2006) identified four (4) major factors for the public interest in corporate governance. They are the technological failure of the dot-com bubble, corporate scandals, ~~too~~ risky strategies manifesting in risk management failure, and the lack of integrity by the board and management. Becht, Bolton and Roell (2003) traced its importance to key economic developments such as the privatisation wave, deregulation and integration of capital markets, wave of mergers and takeover, pension fund reform and growth of private savings, and financial crises. Herrigel (2006) linked the emergence of corporate governance to its root in the factors that shape the nature of corporate property relations. Property relations in this context refers to ownership of the corporation, which could either be in form of concentrated ownership or dispersed ownership. It is often defined in each country by the financial system in place, the governance role of stakeholders versus stockholders, and the political governance of the economy.

### 2.1.4 Global Corporate Governance Principles

Following the spate of corporate governance weaknesses identified in corporations and countries, the need emerged for approaches to guide the implementation of good governance practices. Generally, there are two (2) approaches; the rule-based and principle-based. The rule-based approach treats codes of corporate governance as laws, for which non-compliance is sanctioned whilst the principles-based approach prescribes beneficial practices for which companies are required to comply or explain their non-compliance. The Cadbury report in the United Kingdom, the OECD principles of corporate governance, the Sarbanes-Oxley act in US, and the Basel principles of corporate governance are key global principles towards corporate governance practice.

#### i. Cadbury report - principles of corporate governance

The Cadbury committee was set up to address public concern about lack of investor confidence in the honesty and accountability of UK listed companies. This became important because of

the collapse of companies such as Wallpaper group, Polly Peck that had hitherto falsely published healthy financial statements. Corporate scandals in Bank of Credit and Commerce International (BCCI), and Maxwell group also provided a motivation.

The key recommendations of the Cadbury committee include:

a. "Comply or explain" voluntary principle of corporate governance
b. The board of directors' responsibilities on regular meeting, control over company, monitoring of executive management, and reporting and control of company's position.
c. Separation of CEO and chairperson position, otherwise appointment of a strong independent element, and recognised senior member on the board
d. Majority outside directors on the Board, independent, appointed through a formal process for a specified period
e. Majority non-executive directors on the Board remuneration committees
f. Appointment of an Audit Committee with at least three (3) non-executive directors.
g. Executive directors contracted for not more than three (3) years, remunerated based on remunerations' committee recommendations, for which there should be a full disclosure of total emoluments (Cadbury committee, 1992).

**ii. OECD principles of corporate governance**

The OECD was officially established in 1961 to facilitate cooperation through recognising the interdependence of economies. It provides a platform for nations to identify problems, discuss and analyse them, and promote policies to resolve them. Through its various committees, the OECD Steering Group on Corporate Governance was tasked on reviewing the principles to assure sound financial systems and stability. Millstein (1998) wrote a report on the four (4) important focus areas to achieve effective corporate governance, which are fairness, transparency, accountability, and responsibility. The OECD established a taskforce to operationalise Millstein's recommendations and developed what is now referred to as the OECD principles (Mohamad, 2004). The principles of corporate governance serve as guidelines for countries (OECD and non-OECD) and policy makers to measure the effectiveness or otherwise of their corporate governance framework, with a view to improving the legal, regulatory or institutional structures. The principles provide stakeholders in an organization the right incentives to perform their roles within a

framework of checks and balances, with an overarching objective to support economic efficiency, sustainable growth and financial stability (OECD, 2015).

The first OECD publication on corporate governance principles was in 1999, followed by a reviewed set of guidelines in 2004. The third and latest review was made, adopted, and endorsed in 2015. The review was necessitated by changes in the corporate and financial sectors. The 2015 principles are arranged along six (6) dimensions (unlike the five (5) themes in the 2004 publication), which are: ensuring the basis for an effective corporate governance framework; the rights and equitable treatments of shareholders and key ownership functions; institutional investors, stock markets, and other intermediaries (the new addition in 2015); the role of stakeholders; disclosure and transparency; and the responsibilities of the board. The principles are discussed in brief details as follows:

**a. Ensuring the basis for an effective corporate governance framework**

Organisations are required to develop a corporate governance framework with a view to impacting on overall economic performance, market integrity and the incentives it creates for market participants and the promotion of transparent and well-functioning markets. The legal and regulatory requirements that affect corporate governance practices should be consistent with the rule of law, transparent and enforceable. The division of responsibilities among different authorities should be clearly articulated and designed to serve the public interest. Stock market regulation should support effective corporate governance, while cross-border co-operation should be enhanced, including through bilateral and multilateral arrangements for exchange of information (OECD, 2004; 2015).

**b. The rights and equitable treatments of shareholders and key ownership functions**

Under the 2004 principles, the above theme was split into two, rights of shareholders, and equitable treatment of shareholders. Basic shareholder rights should include secure methods of ownership registration, convey or transfer of shares, obtain relevant and material information on the corporation on a timely and regular basis; participate and vote in general shareholder meetings; elect and remove members of the board; and share in the profits of the corporation. Shareholders should be sufficiently informed about amendments to the statutes, or articles of incorporation or similar governing documents of the company; the authorization of additional

shares; extraordinary transactions; including the transfer of all or a substantial measure of assets that in effect result in the sale of the company.

Shareholders should have the opportunity to participate effectively and vote in general shareholder meetings, ask the board questions; including questions relating to the annual external audit; place items on the agenda of general meetings, and propose resolutions, subject to reasonable limitations; and participate in key corporate governance decisions, such as the nomination and election of board members. Impediments to cross-border voting should be eliminated (OECD, 2004; 2015).

**c. Institutional investors, stock markets, and other intermediaries**

Institutional investors acting in a fiduciary capacity should disclose their corporate governance with respect to their investments, including the procedures. The corporate governance framework should require that advisors, analysts, brokers, rating agencies and others that provide analysis or advice relevant to decisions by investors, disclose and minimise conflicts of interest. Insider trading and market manipulation should be prohibited, applicable rules enforced, and stock markets should provide fair and efficient price discovery. This will be a foundation to promoting effective corporate governance.

**d. The role of stakeholders**

In protecting the rights of stakeholders, the OECD principles require organisations to respect the rights of stakeholders that are established by law or through mutual agreements. They are also required to provide stakeholders the opportunity to obtain effective redress for violation of their rights; provide access to reliable information on a timely and regular basis where the stakeholders participate in the corporate governance process. In addition, the OECD requires organisations to provide mechanisms for employee participation in corporate governance to develop. Stakeholders, including individual employees and their representative bodies, should be able to freely communicate their concerns about illegal or unethical practices to the board and to competent public authorities. The rights of stakeholders should not be compromised for doing this (OECD, 2004; 2015).

**e. Disclosure and transparency**

Disclosure and transparency includes requirements for organisations to transparently disclose their financial and operating results; objectives and non-financial information; major share ownership, including beneficial owners, and voting rights; remuneration of members of the board and key executives; and related party transactions.

**f. The responsibilities of the board**

Board members should act on a fully informed basis, in good faith, with due diligence and care, and in the best interest of the company and the shareholders. Where board decisions may affect different shareholder groups differently, the board should treat all shareholders fairly. The board should apply high ethical standards, and take into account the interests of stakeholders. The board is also responsible for monitoring the effectiveness of the company's governance practices and making needed changes; selecting, compensating, monitoring and, when necessary, replacing key executives and overseeing succession planning. It is also responsible for ensuring a formal and transparent board nomination and election process (OECD, 2004; 2015).

**iii. Sarbanes-Oxley (SOX)**

The SOX act (US) came into force in 2002 following the corporate scandals involving Enron, and Worldcom. The legislation introduced the following key measures:

a. CEO and Chairman to sign a certificate to SEC on the integrity of their financial statements.
b. Forfeiture of bonuses previously earned in the event of a restatement of accounts due to material non-compliance with accounting standards
c. Restriction of external auditors on performance of non-audit works in the same company
d. Rotation of senior audit partner after every five (5) years
e. Enforcement of professional standard in accounting and auditing through an independent board –Public Company Oversight Board
f. Stricter rules on disclosure of off-balance sheet transaction

g. Prohibition of directors from dealing in their companies' shares at sensitive times (Kaplan, 2012)

**iv. Basel's Principles on Corporate Governance**

The Basel Committee in 2010 developed principles for enhancing corporate governance in the wake of the 2007-2008 financial crisis. The principles were reviewed and revised in 2015 to identify additional areas for strengthening a bank's risk governance framework. The 2015 framework was built on thirteen (13) principles stated as follows:

a. Board's overall responsibilities – on strategies, management oversight, and governance framework and corporate culture
b. Board qualifications and composition – sufficient to exercise sound, objective judgment about the affairs of the bank
c. Board's own structure and practices – defined by the board with means for review to achieve effectiveness
d. Senior management –executes company's strategy under the direction of the board.
e. Governance of group structures – a responsibility of the parent company's board
f. Risk management function – independently executed under the Chief Risk Officer with reporting responsibility to the Board.
g. Risk identification, monitoring and controlling – on an on-going departmental and bank-wide basis
h. Risk communication – across the organisation and through reporting to the board and senior management
i. Compliance –A function to be established and approved by the board for risk management.
j. Internal audit – provides independent assurance, and jointly works with the board/management for effective corporate governance
k. Compensation – a structure that supports sound corporate governance and risk management
l. Disclosure and transparency – to shareholders, depositors and other relevant stakeholders

m. The role of supervisors – in providing guidance, supervising and evaluating corporate governance.

### 2.1.5 Corporate Governance Practices Worldwide: Selected Countries and Models

The corporate governance model is dynamic in each country as it responds to local environments and conditions. The approach adopted by a country is largely influenced by a host of factors, which include the cultural, economic, historical, technological, social and legal background of a country, as well as of that of the business environment (Ungureanu, 2008). The approaches are also determined by the prevalent ownership structure, international economic interaction, and the government structure and programmes. Charkham (1994) identified dynamism and accountability as key principles of good corporate governance system. Dynamism allows managers to run the firms as they wish without undue fear of displacement, government interference or litigation. Accountability allows managers independent decisions provided the outcomes meet at least certain pre-defined standards, or appropriate remedial actions can be taken in a timely way. Corporate governance models around the world could generally be classified into one-tier, two-tier or in-between. With a one-tier model, all duties relating to a firm's monitoring and executive functions are assigned to one board, as obtains in US. The two-tier model however allots these functions to the supervisory and the management boards, as obtains in Germany. Switzerland and France are in between the models (Schneider and Chan, 2000), while firms in Switzerland have the leeway to choose any suitable option.

The anglo-saxon model practiced in countries such as U.K and U.S recognises three key governance parties, the shareholders, directors and managers. The model is influenced by the capital market, regulatory institutions and an independent board. The continental European model practiced in Germany, and Italy provides participatory role for shareholders to manage and control enterprises, whilst managers have a wider responsibility to stakeholders. The Japanese model in contrast is built on a nexus of relationships between industrial groups, managers, and keiretsu (a network of loyal customers and suppliers). Banks play key role in monitoring and financing businesses (Ungureanu, 2013). Charkham suggested that there is

virtually no government interference policy in the UK, very little in Germany, many in France and the most in Japan. The threat of litigation keeps US managers on their toes and makes them acutely conscious of the possibility of any potential lawsuits. In other countries, litigation is much less prevalent. German and Japanese systems are more effective against mismanagement, as managers' decisions are far less influenced by the potential responses of stock markets and the threats of takeovers (Charkham, 1994).

### 2.1.6 Governance Value Models

#### i. Shareholder Value Model

The Shareholder Value Model has served corporations and different economies for decades because of its perceived benefits and structural framework. The model builds on the relationship between the shareholders (owners of the company) and the board of directors. The shareholder value model promotes the equity market as the best benchmark for determining a company's value, and executive compensation tied to share incentive as the most efficient way to align managers and owners' interests. In addition, the model has three (3) salient features, a competitive market for equity capital, alignment of managers' and shareholders' interests, and a set of gatekeepers (Vitols and Kluge, 2011). The model postulates that the market controls the quality of management of boards of directors, whose role is to maximize the shareholders' value. Where the board fails in its duty, the shares of the company will attract less demand, thus making the company vulnerable for hostile takeover or a replacement of the board. The board therefore seeks ways to steer the company towards prosperity in order to protect their own jobs and reputation (Sustentare-SAM, 2010). This model is however fraught with traps. For instance, boards of directors in some climes have incorporated "golden parachute" payments in their contracts, while managers have their remuneration tied to short term performance. These incentives have been counter-productive in some instances leading to unethical and illegal practices which accounted for the fall of companies such as Enron, Andersen, and Parmalat. With financial scandals and economic crises recurring around the world, many researchers and policy makers have attributed the shareholder value model as a major contributory factor; thus, its questionable status as the primary corporate governance theory.

The underlying assumptions of the shareholder value model appear not to be holding up to scrutiny. For a start, some of the theoretical assumptions are questionable. It is not always valid that shareholders and potential shareholders have access to all relevant information to take well informed decisions, neither can they buy and sell their shares without any limitation in form of market barrier (Sustentare-SAM, 2010). Share price does not reflect strong relationship with company performance as it tends to correlate with the general movement of the stock market, neither is it a holistic performance measure to the extent that social and environmental costs are not fully factored in its components. Other flaws include the unintended consequences of using share-based incentive schemes to motivate CEO's performance; ironic choice by institutional investors; and the inability of gatekeepers to protect interests of the shareholders. The share-based incentive provides opportunity for fraud and manipulation as institutional investors adopt short term stock-holding strategies, whilst the gatekeepers, such as regulators and rating agencies have differing interests or lack experience or knowledge to perform their roles effectively (Vitols and Kluge, 2011). Determining what is in the best interests of shareholders may not even be straightforward, depending on the type of shareholder. The objective of a founder/family shareholder is quite different from an institutional investor who came in on the back of an initial public offer, or venture capitalists looking for an early exit from the business (Nordberg, 2007).

### ii. Stakeholder Value Model

Stakeholder value model has long recognised that the company is a social organization dependent upon the contribution of different groups to the production of goods and services (Freeman and Reed 1983; Freeman 1984; Hutton 1995). Employees offer their skills, suppliers offer goods or services, investors provide capital, and community provides infrastructure. These stakeholders have expectations in terms of jobs, products or services, dividends, and tax respectively. Nevertheless, the objectives of the owners and other stakeholders have often brought them in conflict, with the owners more concerned about profit rather than meeting the needs of other stakeholders (considered by owners as a value eroding strategy). The post-World War II period provided opportunities for expanding the field of players in corporate governance to include major role for employees (Jackson 2001; Streeck and Yamamura 2001).

The stakeholder value model holds companies responsible for value creation and distribution to not only the shareholders but all its stakeholders. This argument is based on an ethical and moral basis, and therefore expects the board to represent the interests of all stakeholders. While the model is generally criticized for its impossible demands on companies to follow multiple stakeholders' goals which may also be contradictory, the response of the proponent has been that value cannot be created for shareholders if they are not created for stakeholders. For instance, an organisation is required to provide goods and services which customers are interested in purchasing; jobs attractive to employees; good relationship with suppliers, and be socially responsible to the community in which they operate (Sustentare-SAM. 2010).

### iii. Enlightened Shareholder Value Model

A middle-of-the-ground model evolved as the enlightened shareholder model, to harmonise the benefits and limitations of the shareholder and stakeholder value model (Jo and Harjoto, 2012). The model rests on the maximisation of the shareholder value still, except with a proviso that the company managers/board need to understand the expectations of the legitimate stakeholders and act accordingly within those expectations in order to create value for the shareholders (Jensen, 2001). Not totally far from the stakeholders' argument is the importance of having the following ingredients for success – satisfied customers, satisfied suppliers, motivated employees, and supportive community. Companies engaged in strategic dialogue and relationship with stakeholders are thus required to build sustainability in their business models. This is in line with the dynamics of the contemporary business world.

### iv. Sustainable Value Model

Nations come together to proffer solutions to challenges considered threatening the world or citizens (for instance the earth summit on climate change, globalisation and free market initiatives) or respond to the changing human and social values, as new concepts such as responsible consumerism, environmental justice and equity are emerging. These global developments impose a responsibility on organisations to be compliant, and in most cases the issues of transparency, and inclusiveness become paramount. The hitherto narrow view of corporate governance as the management of relationship between owners and managers can therefore no longer hold, so also the obsession with the financial performance dimension alone.

A responsive organisation therefore needs to perform against the ‘triple bottom line’, which has led to the emergence of a new concept of a sustainable value model based on a sustainable company (Vitols and Kluge, 2011).

### 2.1.7 Legislative and Institutional Framework

The framework for mandatory implementation of corporate governance in Nigeria is enshrined in three (3) acts. These are the Companies and Allied Matters Act (CAMA) of 1990, Investment and Securities Act (2007), and the Banks and Other Financial Institutions Act (BOFIA).

#### i. Companies and Allied Matters Act (CAMA)

The company law (CAMA) governs the incorporation of companies and incidental matters, registration of business names and the incorporation of trustees of certain committees, bodies and associations. Some relevant areas of guidance for public corporations relating to corporate governance are stated as follows:

a. **Provision of financial statement**: The Act requires the directors of every company to prepare financial statements reflecting a true and fair view of the operations of the company during the financial year. These financial statements must be presented to the shareholders at the Annual General Meeting, and it must at a minimum, provide information on accounts, directors’ report, information about emoluments of directors, disclosure of relevant information about subsidiaries among others. In addition, it provides for a time frame of at least twenty-one (21) days within which the statements must reach the shareholders (CAMA, 1990).

b. **Audit and Auditor**s: The Act provides for the appointment/re-appointment of auditor(s) by companies at the Annual General Meeting (AGM). The appointed auditors are responsible for auditing the financial statements prepared by the directors and presenting same at the company’s AGM. There are prohibitive laws precluding the appointment of officers or servants of the company as auditors. Audit committee members are also required to examine the report of the auditors and make appropriate recommendations to the AGM.

c. **Minority Shareholders**: The Act makes provision for the notice of company's general meeting to all shareholders irrespective of their holdings. To further protect the minority shareholders, the act provides for one-member one-vote in the event of a poll being taken. Equally, any member of the company may approach the courts of law to seek redress if his or her name is omitted from the company register without sufficient cause.

d. **Register of members**: Each company is mandated to keep a register of members/shareholders, showing details with regards to shareholding. Shareholders having at least 10% of the unrestricted voting rights in the company are required to be listed in a register of interest in shares

e. **Voting and Proxy Rights** – The Act under Section 116 (1a) provides for one-share-one-vote in Nigerian companies. In addition, the act provides for proxy representation at a general meeting. The proxy is conferred with the same attendance, voting, and speaking rights as the shareholder that appointed the proxy.

f. **Meetings** – The Act provides for both statutory and general meetings in companies. Statutory meetings are held within six (6) months of the incorporation of the company. General meetings can either be annual or extraordinary type. The annual meeting is mandatory and it is held to deliberate the issues discussed in earlier paragraphs. The extraordinary meeting can be convened by the Board of Directors/Director or requisitioned by any member holding 10% or more of the company's shares at the requisition date. The general meeting also provides an avenue for members to appoint and remove directors, and approve their remuneration.

g. **Directors' Fiduciary Duties** – Section 279 of the Act lays the general premise of the directors' fiduciary duty. It states that "a director of a company stands in a fiduciary directors' relationship towards the company and shall observe the utmost good faith towards the company in any transaction with it or on its behalf". The Act provides in

Section 279 (4) that the directors owe a duty of care to the shareholders, employees and other stakeholders. It further provides in Sections 280 and 281 that directors are prohibited from having a conflict of duties and interest CAMA (1990).

**ii. Investment Securities Act**

The Investment Securities Act (ISA), established the Securities and Exchange Commission, a regulatory body with the responsibility of overseeing the capital market and securities investment. The Act oversees the functions of the capital market, infrastructures and regulatory system of investment and securities business. It makes specific provisions for investors protection fund; mergers, take-overs and acquisitions; offer, sale and trade in securities; corporate responsibility of public companies; and registration and regulation of securities players. It also provides for the responsibility of the board with regards to the integrity of the financial reporting and control, a breach of which attracts prosecution. Overall, the Act seeks to achieve the reduction of systemic risk, and promotion of a fair and efficient market (ISA, 2007).

**iii. Banks and Other Financial Institutions Act**

The Banks and Other Financial Institutions Act (BOFIA) was enacted to regulate the banking industry. The responsibility for regulation and supervision lies with the Central Bank of Nigeria, which also has the powers to enact appropriate regulations in furtherance of the BOFIA's objectives. The BOFIA contains specific provisions on establishment of banks, duties of banks, books and records of account, supervision, and other miscellaneous matters, which are explained as follows:

a. **Establishment of banks**: This aspect of the Act deals with application, issuance and revocation of license; entry of foreign banks, mergers and acquisitions, minimum share capital requirement; and maintenance of accounting ratios on capital, cash reserves and liquidity.
b. **Duties of banks**: The maintenance of reserve fund, restriction on dividend and certain banking activities, disclosure of directors' interests; and prohibition of employment of certain persons and interlocking directorship.

c. **Books and records of account**: Maintenance of proper books, monthly returns, publication of annual accounts and consolidated statements; and the appointment, power and report of approved auditor.
d. **Supervision**: Entails the power of director of banking supervision and other examiners, routine bank examination and report; special examination; failing banks; significantly under-capitalised banks; restriction on use of certain names; general restriction on advertisement for deposits; trade union; and disqualification of certain members from bank management.
e. **Other miscellaneous matters**: Covers offences by banks, their servants and agents, directors and managers; priority of local deposit liabilities; and the application of CAMA and NDIC Act (BOFIA, 2002).

Notwithstanding these provisions protecting shareholders and other stakeholders, only a few reported cases of redress of breaches have been brought to the law courts as noted by Oserogho (2003), and this is partly due to the high cost of litigation, the unduly long process in adjudicating and corruption in the judiciary.

### 2.1.8 Status of Corporate Governance in Nigeria

Corporate governance structure and behaviour is largely determined by the laws operating in a country (Morrison, 2004), hence Nigeria's history of corporate governance cannot be divorced from the influence of Britain as the erstwhile colonial masters. Prior to independence, Britons had a stronghold economically through principal ownership of large corporations in the country (Adegbite and Amaeshi, 2010), and were able to influence the corporate governance system through the provisions of the company legislation patterned after the Anglo-Saxon model. Long after the end of colonial rule however, Nigeria still retains the inherited legal system of corporate governance. The question however remains if the British corporate laws are complementary, reflective and applicable to the local business environment (Adegbite and Nakajima, 2011). Paradoxically, the agency theory and the stakeholder theory have been largely influential in the corporate governance system in Nigeria. Yakasai (2001) traced the prominence of the stakeholder orientation to the societal concerns raised against multinational corporations (MNCs) considered too imperialistic and powerful to be held accountable, especially considering their activities in the Niger Delta region and the spate of

environmental degradation and pollution caused by the oil and gas companies. The equally significant investment of government in the oil sector also elevated other stakeholders as critical to the success of the firm beyond just the shareholders. The agency orientation follows the argument about man's self-centeredness and the problem of trust. This is considered an apparent challenge in Nigeria due to corporate executives expropriating company's resources for personal gains at the expense of the investors (Adegbite and Nakajima, 2011), endemic culture of corruption and bribery, ethnic tensions and rivalries, poorly functioning markets, and lack of adequate infrastructure (Ahunwan, 2002).

Corporate governance regulatory consciousness in Nigeria was triggered in 2002 with the inauguration of a committee mandated by the Securities and Exchange Commission (SEC) in Nigeria to develop a corporate governance guideline for public companies. This initiative heralded the SEC Code for Corporate Governance in 2003, which though was made voluntary, but nevertheless treated as a prerequisite for being listed on the Nigerian stock exchange. Other advancements in legislation include those covering the pension - National Pension Commission (PENCOM), and insurance industries - National Insurance Commission (NAICOM). In the banking industry, the Code of Corporate Governance for Banks in Nigeria – was issued by Central Bank of Nigeria (CBN) in 2006 and later updated in 2014. Following the introduction of these codes especially the SEC Code of 2003, the country has witnessed a gradual but consistent improvement in corporate governance practices (Onuoha, Ogbuji, Ameh and Oba, 2013). Notably, the banking sector has been at the forefront of ensuring the entrenchment of good corporate governance practices. This may be attributable to various cases of bank distresses leading to substantial loss of depositors' funds.

However, despite the commendable strides achieved in Nigeria with respect to corporate governance, there are implementation concerns which have limited the attainment of global best practice in corporate governance in the country (Okike, Adegbite, Nakpodia and Adegbite, 2015). These issues and challenges, for analysis purposes, can be categorised into three (3) factors namely operators, regulators and stakeholders. At the regulatory level, attempts have been made through the different codes in existence to prescribe appropriate levels of best practice for corporations. However, the ability of companies to imbibe corporate governance

seems to be linked to the following questions: Which would be more desirable between a mandatory and voluntary compliance with corporate governance practices? How can managers be convinced of the benefits of disclosure, transparency, avoiding conflicts of interests etc. especially in a secretive business environment like Nigeria? Adegbite (2012) emphasised that human capacity and these complexities are major challenges to the effective monitoring of compliance and enforcement in relation to corporate governance.

Corporate governance challenges in Nigerian banks can be categorised based on the pre-consolidation and post-consolidation periods.

**i. Pre-consolidation period**

Fifty-nine (59) banks were closed between 1994 and 2006 (CBN, 2008). The introduction of code of corporate governance was predicated on the weaknesses noted in corporate governance practices by banks. These weaknesses can be grouped into four (4) factors: board, structure and processes, shareholders, and stakeholders. At the board level, there were instances of board squabbles, ineffective oversight, fraud, and power struggle for entrenchment of board positions (Adeyemi, 2005). Weak structure and processes led to override of internal controls, poor risk management practices, and non-compliance with laws and regulations. The shareholders were generally passive, and depositors as stakeholders wielded strong influence in maximizing their values through ~~a~~ pressure on banks for high interest rates.

**ii. Post-consolidation period**

After the consolidation exercise, the emerging banks (especially resulting from mergers and acquisitions) grappled with conflicts of culture and control, inadequate capacity by board and management to run a much bigger bank, poor risk management, expropriation, and infrastructural challenges –in the area of integrated IT and accounting systems among others (Agusto, 2006). A consequence of the banking consolidation exercise is the reduction in number of banks from eighty-nine (89) to twenty-five (25) immediately after the consolidation exercise (Sanusi, 2012). This creates an oligopolistic market with the dominance of a few large players considered too big to fail. Ahunwan (2002) stated the failure of oligopolistic firms to effectively subject management to pressure, which defeats one of the corporate governance

mechanisms propounded under the agency theory i.e. the concept of product market as a disciplinary measure against managers promoting their own interests.

The introduction of corporate governance codes also led to several changes such as separation of CEO and chairman's roles, defined tenure for CEOs, and limit of government's equity holding in banks to 10%. Other changes include a requirement for prior approval of CBN for equity holding of 10% and above by any investor, strict rules on board composition, and requirement for board performance appraisal. In addition, the codes prescribe for independent directors, and removal of any director from the board who has a non-performing loan aged one year among others. Nevertheless, by 2009 the Nigerian banking industry witnessed another crisis largely attributed to corporate governance failure. Eight (8) banks' CEOs were sacked by CBN in 2009 (Ojeka, Iyoha and Ikpefan, 2014). Around this time, the apex bank injected ₦620billion into problem banks to forestall further collapse. Twenty-four (24) banks that declared total profit of ₦658 billion in 2008 recorded total loss of ₦1.37 trillion in 2009, as a result of improper and inappropriate accounting for their non-performing loans (NDIC, 2009).

### 2.1.9 Nigeria's Financial System

The financial system is made up of the following actors and participants: CBN, NDIC, AMCON, SEC, NAICOM, PENCOM, the Federal Mortgage Bank of Nigeria (FMBN), deposit money banks, merchant banks, non-interest bank, discount houses, microfinance banks, finance companies, primary mortgage banks, bureaux-de-change (BDCs), private credit bureaux, mobile money operators, Nigeria Stock Exchange (NSE), commodity exchange, mortgage refinance company, and development finance institutions (CBN, 2015).

### 2.1.10 Nigeria's Banking Industry

The history of banking in Nigeria started with the establishment of the Bank of British West Africa (which later became First Bank of Nigeria) and commencement of its business in 1892. Several other indigenous banks emerged in the 1930s-40s, but failed a few years later, leading to the need for regulation. The Central Bank of Nigeria birthed in 1959 through the CBN act of 1958 (Ali, Ekpe and Aigba, 2016). Between then and the late 80s, there were a few banks (about 29) providing commercial and merchant banking (Enendu, Abba and Fagge et al.,

2013). The liberalization policy – a component of the structural adjustment program of the government in 1986 heralded the proliferation of banks and other financial institutions which peaked at around 120 banks by 1994. By 2004, there were eighty-nine (89) banks in Nigeria (Soludo, 2004), mostly private and family-owned. On the regulatory front, the government enacted two (2) pieces of legislation, the Banks and Other Financial Institutions (BOFI) and the CBN Acts of 1991, complemented by the prudential regulations and the set-up of the Nigerian Deposit Insurance Corporation to insure banks' deposit liabilities. Reforms such as mandatory minimum capital requirement, interest rate deregulation, relaxed industry entry, and universal banking were introduced. Minimum capital requirement was increased from ₦500million to ₦2 billion in 2002, and ₦25 billion after a new wave of reforms in 2004. The 2004 reform mandated banks to increase their capital base to a minimum of ₦25 billion on or before 31 December 2005. This led to series of action steps by banks, such as sourcing funds from the capital markets, private placement, and mergers and acquisitions, effectively shrinking the number of banks from 89 to 25. Notwithstanding these initiatives, there was a crisis in the industry around 2009, which coincided with the financial crisis happening globally at that time.

Banks engaged in too risky investments, and as the effect of the global crisis impacted the capital market leading to a bubble burst, most banks were negatively affected. The CBN intervened decisively to prevent the collapse of the affected banks through several initiatives – reduction of mandatory financial ratios, injection of cash, and implementation of corporate governance. Other policies introduced include the mandatory adoption of 31 December as the financial year-ends for banks, on-site CBN examiners in banks, and sanctions where appropriate. These reforms have yielded results. For instance, improvements in the payment system through electronic channels leaves only about 9% of currency outside the banking system; the ratio of credit to the private sector to GDP (CP/GDP) rose from 14% in 2003 to 60% in 2010 signifying the huge leap in contribution to the real sector (Enendu, Abba and Fagge et al., 2013). A summary of important developments and regulations in the banking industry include the following:

i. 1952 - Banking Ordinance
ii. 1988 - Nigerian Deposit Insurance Corporation (NDIC) created

iii. 1990 – CBN introduced prudential guidelines for banks
iv. 1991 - Banks and Other Financial Institutions Act (BOFIA) enacted
v. 1994 - Failed Banks' decree promulgated
vi. 1997 - CBN and NDIC Acts amended
vii. 2003 - Money Laundering Act enacted
viii. 2005 - Banking Consolidation exercise
ix. 2006 - Code of Corporate Governance for Banks established by CBN
x. 2012 – Sustainable Banking Principles established by CBN

### 2.1.10.1 Central Bank of Nigeria

The CBN is the apex regulatory authority of banks and other financial institutions in Nigeria. It was established by the CBN Act of 1958 (severally amended over the years), but commenced operations in 1959. Some of their roles include:

i. Acting as banker to other banks
ii. Acting as banker to the government
iii. Lender of last resort
iv. Issuer of legal tender
v. Achieving financial system stability
vi. Debt management and debt financing
vii. Maintenance of external reserves
viii. Consumer protection
ix. Administering BOFIA (1991) etc.

The CBN through its regulation and supervision function, conducts onsite examination and offsite surveillance of activities by the banks. It is also a party to international regulations such as the core principles of the Basel Committee on banking supervision.

### 2.1.10.2 Banking Reforms in Nigeria

The banking sector has witnessed several reforms in the wake of global financial crises and its attendant effects which has led to bank losses and failures in Nigeria. These reforms are summarised below:

**2004-2008**

i. Banking consolidation programme through mergers and acquisitions in 2004, which raised minimum capital from ₦2billion to ₦25billion. The aim was to strengthen the banking sector through bigger balance sheet size in order to secure the safety of depositors' money, elicit active developmental roles in the economy, and become key players in the continent and globally (Soludo, 2004). This led to a reduction of number of banks from eighty-nine (89) to twenty-five (25) in 2005.
ii. Introduction of corporate governance codes
iii. Phased withdrawal of public sector funds from banks
iv. Automation of rendition of returns by banks
v. Changes in regulatory framework to be risk-focused, zero-tolerant on financial reporting infractions, enforcement of corporate governance principles and other laws.
vi. Key interventions in the real sector

**2009-2015**

Despite the reforms introduced pre-2009, another round of financial crisis engulfed the banking industry. Twenty-Four (24) Nigerian deposit money banks that had recorded N658billion profit in 2008, posted N1.37 trillion loss in 2009 leading to the sack of eight (8) bank CEOs (NDIC, 2009). This was because the banks had previously under-reported the extent of their non-performing loans, and were mandated to do so after CBN's examination, showing the ephemeral nature of financial performance. The following reforms were subsequently introduced:

i. Collaboration with Securities and Exchange Commission (SEC) and the Nigerian Stock Exchange (NSE) to reduce cost of transactions in bond issues borrowing
ii. Establishment of Asset Management Corporation of Nigeria (AMCON) in 2010 to take-over non-performing loans of banks.
iii. Adoption of International Financial Reporting Standards (IFRS) in 2010.
iv. Customer protection initiatives
v. Reviewed the Universal Banking model with replacement by new classification of banking licences as deposit money banks, merchant bank, specialised banks, and development finance institutions.

vi. Introduction of non-interest banking
vii. Adoption of cash-less policy initiatives (Sanusi, 2012).

### 2.1.11 Nigeria Deposit Insurance Company (NDIC)

The NDIC ("corporation") was set up by an act of parliament in 2006 to primarily insure all deposit liabilities of licensed banks and such other deposit taking financial institutions. In furtherance of this objective, NDIC is also responsible for providing assistance to insured institutions in the interest of depositors, and guaranteeing payments to depositors in case of imminent or actual financial difficulties. The corporation is funded by insurance premium on customers' deposits paid by the licensed banks, investment income, and approved borrowings. Insurance premium is computed as 15/16 (fifteenth-sixteenth) of 1(one) percent per annum, and it is wholly borne by the licensed banks (i.e. not recoverable from depositors). In the event of the revocation of the operating licence of a bank, a depositor shall receive from the corporation a maximum amount of ₦200,000. For this purpose, all accounts held by the depositor in the same right and capacity shall be consolidated as 1 (one) account. A depositor is however still entitled to liquidation dividends that may accrue once the assets of the failed institution has been realized. The corporation has regulatory powers over the licensed banks through supervision and on-site examination of records (NDIC, 2006).

### 2.1.12 Governance in the Banking Industry

Corporate governance has been studied severally in relation to firm performance, the banking industry offers a different prospect because of its unique and differentiating characteristics discussed below. As Haan and Vlahu (2016); and Laeven (2012) argued, focusing on shareholder value only and ignoring regulatory distortions has limited applicability in research on corporate governance of banks.

#### i. Agency Governance Mechanisms Effect

Agency mechanisms such as concentrated ownership, managerial incentives, large creditors and debt-holders, and market discipline among others propounded by the agency theory are more effective in non-financial industry than the banking industry (Laeven, 2012). Most countries have regulations restricting significant ownership of banks, or at the least the

presence of a legislation requiring approval by the regulatory authority. This therefore leaves banks with diffused ownership structure almost as a fait accompli. In a diffused ownership structure, the shareholders are not able to monitor the managers effectively because of their diffused status, and the lack of skills, and finance to affect such effective monitoring. While incentives such as cash, bonus, stock ownership and long-term incentives may encourage managers in non-banking industry to push harder for increased value, the equivalent opportunity for the bank manager is to take increased banking risks in order to improve earnings. Whilst this is in line with the shareholders' objective, the manager may not be favourably disposed to this objective, which puts them in conflict with owners due to their views on risk-taking (Laeven and Levine, 2007), or lack of human capital skills and private benefits of control.

Large creditors or debt-holders play a critical role in the corporate governance mechanisms of non-banking industry. Their interests are generally covered by the contracts rights executed with the debtors, and they can therefore trigger clauses applicable to breaches without requiring a collective meeting of other creditors. Their debts are also generally short-tenured, which ensures borrowers come for more funds at regular short intervals (Shleifer and Vishny, 1997). Bank depositors on the other hand do not enjoy this privilege. In the first instance, they are dispersed and therefore not able to monitor the activities of the bank managers strictly. Moreover, the information asymmetry in banks is much deeper than in other industries due to the complex nature of transactions, but more importantly depositors lack the power or influence to renegotiate their debts. Market discipline mechanisms such as, a market for managers to replace poor performing managers, and activities such as hostile takeovers in the banking industry do not achieve the same effectiveness as in other industries. While firms in the non-banking industry can be targets for hostile take-over, the banking regulations (at least in Nigeria) prevent such practices. Mergers and acquisitions plans must necessarily be reviewed and approved by the banking regulatory institution.

**ii. Diverse Objectives**

Regulators have different objectives from the shareholders. Shareholders are concerned about value maximisation primarily and the regulators are more focused on achieving financial

stability. This objective drives the regulators in directing bank boards to align the shareholders' interests with the regulators' and this may be achieved through restrictive policies on the activities of the banks and their boards. These restrictions impact one of the corporate governance mechanisms in literature – the market for corporate control (Renee, 2010). The banking industry is critical to the economic development of a nation, and a bank's failure may have ~~a~~ systemic effect and grave implications because of their distinctive roles in the payment system and financial intermediation. Hence, there is a higher degree of regulation by government agencies compared to non-banking firms (Flannery, 1998). The bank therefore owes accountability not only to its shareholders but also to the regulatory authorities.

**iii. Capital structure of banks**

The capital structure of deposit money banks distinguishes them from other firms. In the first instance, they carry a low level of equity capital compared to debt, which makes them more leveraged. By transforming illiquid assets into liquid liabilities, the banks generate liquidity for the economy (Macey and O'Hara, 2003) through this financial intermediary function. Since their liabilities are mainly in form of demand deposits while loans have longer maturities, this raises an unintended consequence in banks being exposed to potential coordination failures, referred to as 'bank run' (Diamond and Dybvig, 1983, Ferrarini, 2017).

**iv. Safety Nets**

Regulatory bodies such as the CBN provide safety nets in form of being lender of last resort, as well as the creation of deposit insurance scheme to prevent bank runs. Ironically, deposit insurance schemes creates a moral hazard by energising managers and owners of insured banks to engage in excessive risk-taking whilst luring depositors into lull on the need to monitor the managers (Macey and O'Hara, 2003, Ferrarini, 2017). This is more so knowing that managers can transfer some of their losses to innocent third parties.

**v. Multiple Stakeholders**

In non-banking firms, creditors or debt holders could protect their interests by writing contracts that prevent managers from taking excessive risks, or otherwise guarantee their own fixed claims. The deposit insurance scheme takes away the depositors' incentive to control excessive

risk-taking, thereby rendering the consequence of management's risky investment strategies to be of no effect. This therefore raises two important points. First, the importance of two other critical stakeholders in the deposit money banks – the depositors and the regulators, and as argued by Macey and O'Hara (2003) the need to expand the bank directors' duty of care beyond the minimum required for non-banking firms.

**vi. Other Factors**

When banks undertake risky transactions in order to maximise owners' returns, the shareholders take the returns without extending to the significant providers of funds (depositors), but when it goes sour the depositors may end up losing all their deposits. Therefore, 'a major part of the losses is externalised to stakeholders, while gains are fully internalised by shareholders' (Ferrarini, 2017), which goes against Sheifer and Vishny (1997)'s assertion that there is a need for suppliers of finance to corporations, to assure themselves of getting a return on their investment. Banking transactions are also more opaque, complex and voluminous than in non-banking industry, thereby creating more information asymmetries for depositors and consequently limiting the impact of market discipline (Tarraf, 2011; Ferrarini, 2017).

Empirical literature on bank governance that generally adopted the same measures applied in non-banking firms, such as board size, independent directors, and pay-for-performance metric (Mishra and Nielsen, 2000), gave little or no consideration to factors such as regulation, banks' transaction types, and organisational structure, which add a different breadth to the understanding of what can be termed effective governance (Renee, 2010). The foregoing analysis demonstrates the peculiarity of banks' corporate governance and the need for a design that aligns the manager with the interests of debt-holders and depositors (Acharya, Carpenter, Gabaix *et al.*, 2009) among others.

### 2.1.13 Banking Supervision – Emergence of BASEL Capital Accord

In 1974, the international banking sector experienced a shock arising from currency exchange risk failure. A transaction involving several banks resulted in the release of Deutsche Marks (German currency) to Bankhaus Herstatt (a Bank in Germany) for funding of the transactions,

in expected exchange for US Dollars. Unfortunately, due to time-zone differences between Europe and America, the US Dollars could not be released before Bankhaus Herstatt failed and its operational license suspended, thus leading to a huge loss on the part of the counterparty banks (~~BIS~~ Bank for International Settlements, 2004). To mitigate this form of cross-continent risks, some countries came together to form a committee under the Bank for International Settlements (BIS) platform, which later metamorphosed into the BASEL committee. The committee has since released several guidelines to standardize banking practices across the world referred to as BASEL I, II, and III, each being a build-up on the previous Basel capital accord to mitigate unaddressed banking risks.

i. **Basel I:**

This was targeted at mitigating credit default risks by providing guidelines on minimum capital requirements for major banks, which was set at 8% of banks' risk-weighted assets. It was introduced in 1988 to strengthen the stability of international banking system, and set up a fair and a consistent international banking system in order to decrease competitive inequality among international banks (Banks for International Settlements - BIS, 2016).

ii. **Basel II:**

It sought to address the narrow focus of Basel I on financial risk metrics by proposing a comprehensive risk management process. Introduced in 2004, the committee responded to the changing financial risks landscape resulting from innovative but questionable banking products by providing guidelines among others for risk management (differentiating between operational, credit and market risks), capital adequacy, and disclosure requirements by banks. The three main pillars of Basel II are minimum capital requirements (at 8% of assets), supervisory review process (by the country's banking regulator), and market discipline (through disclosure of risk).

iii. **Basel III:**

Introduced first as interim measures in 2009, and later updated and endorsed in 2010, these guidelines were regarded as the committee's response to the inadequacies of the BASEL I and II to prevent the financial crisis that erupted in 2008 as a result of systemic risks – the devastating impact a single bank could have on individual countries and world financial markets. The guidelines address funding, capital, leverage, and liquidity. The implications of

implementing the Basel I, II, and III accord include the following: tighter controls and increased costs on banks' risk behaviour, and a comprehensive signal to prevent failures (BIS, 2016). The foregoing analysis underscores the importance of regulation and supervision in corporate governance of banks.

The next sections provide a conceptual framework of how the constructs (corporate governance, and sustainability) and their associated variable measures were tested.

### 2.1.14 Framework for Corporate Governance

A major challenge in corporate governance studies is the lack of consensus on the effect of corporate governance on performance. Empirical studies have adopted the use of different methods to determine the causative relationship between corporate governance and performance, with differing results. Some other studies develop corporate governance index (Brown and Caylor, 2006; Klapper and Love, 2003; Uwuigbe, 2011) drawn from several sources such as the national codes in operation in the country of study, OECD corporate governance principles, BASEL principles on corporate governance, or international corporate governance rating agencies (such as CLSA, GMI, ISS) among others. Some of the explanations for mixed results include the selection of variables, the context in which the study was performed, the theoretical framework adopted, and the model in operation in the country of study. Studies such as Ho (2005), and Walls, Berrone, and Phan (2012) have opined that using a one-dimensional method may yield misleading results because it may not explain the overall corporate governance effect on performance, while Aguilera, Desender and de Castro (2012); and Al-Baidhani (2014) have suggested the use of corporate governance 'bundles' as a solution. Corporate governance bundles approach entails combining a set of internal, external, and other mechanisms to determine the effect of corporate governance in performance. Corporate governance bundles provides opportunity to determine the "complementarity or substitutability" of the mechanisms.

Corporate governance is viewed as not only concerned about solving agency problems that seek the interest of only the shareholders (as advocated by the agency theorists such as Jensen and Meckling, 1976) but taking into consideration the needs of all critical stakeholders (as

advanced by the stakeholder theorists such as Freeman (2001), and Donald and Preston (1995). Nigeria is classified as a developing country operating the anglo-saxon model borrowed from UK, without the supporting infrastructures such as legal system, market discipline, and protection of shareholders among others. In view of the foregoing, this study views corporate governance construct from two major concerns – solving the agency problems through: the *internal governance controls*, and *agency mechanisms*, and protecting other stakeholders through: *ethical leadership*, *regulation*, and *corporate governance practices disclosure*. Each concept is further explored below.

### 2.1.14.1 Internal Governance Controls

Internal governance controls are internal self-managed mechanisms and activities put in place to ensure proper corporate governance in organisations. For this study, they consist of the board structure, management structure, and ownership structure. They are discussed in more details below:

#### 2.1.14.1.1 Board Structure

The board of directors is considered a very critical mechanism in corporate governance because of its role in monitoring, advising and directing the organisation. The board largely seeks to align firm's interests and shareholders' values (Guo, Smallman, and Radford, 2013). The board of directors delegates day-to-day operation to management, but reviews their activities on periodic basis. It exercises oversight on strategy formulation, risk management, capital acquisition, talent management and engagement with key external stakeholders. As an advisor, the board provides opinions and directions to managers for key strategic business decisions. To play their role effectively, Jensen (1993) stipulated a separation of the CEO-Chairman role/function, a modest number of directors about eight, inside ownership of shares, free access to information, and restriction of board membership to only the CEO as the management representative. To achieve its objectives, the board carries out its function through different structural arrangements such as board size, board composition, independence, committee composition and membership, effectiveness, and diversity of the board members.

### i. Board Size

Board size is expected to influence performance through the decision-making process, even though there is an argument about which size is better between small and large board. Large boards are believed to increase the wealth of experience and resources available to the organisation, and thereby able to advise and monitor the performance of the executive management (Upadhyay and Sriram, 2011). They are also able to break into committees required to review information provided by management for corporate decisions. The opportunity to scrutinize management information partly helps in solving information asymmetry problem associated with principal-agent relationship, since information transparency is enhanced. This becomes important as banking transactions are usually voluminous and complex, especially where they have subsidiaries and, or several branches spread across different countries. Pathan and Skully (2010) found that bigger and more diversified banks have larger and more independent boards. Proponents of a small-sized board hinge their argument on its cost-effectiveness in decision-making as an advantage, and also point out several disadvantages of large-sized boards. For instance, they point to the 'free-rider' problem (Aebi, Sabato and Schmid, 2012) associated with the passivity of some board members (Mehran, Morrison, and Shapiro, 2011; Uwuigbe and Fakile, 2012), ineffective decision-making due to "group think" (Janis, 1983), or 'social loafing' syndrome (Bainbridge, 2002) that makes members 'yes-men'. In addition, there is a tendency for pluralistic ignorance (Westphal and Bednar, 2005) as the syndrome that causes members to bottle-up alternative views because no one is airing such dissenting view.

Board members are also social beings interacting towards achieving the best results for the corporation they monitor and advise. The context of their interaction therefore has a bearing on their decision-making effectiveness, and therefore their ability to pursue the same goals in unity or at cross-purposes. Equally important is a board with the right mix of experience, diversity, and representation to carry out its responsibilities effectively at committee and full board level. As Dorger (2011) averred, it is imperative to consider the firm size, depth, and level of complexity of issues facing the corporation to determine the optimal board size. With regards to Nigerian banks, the CBN code on corporate governance (CBN, 2014) provides for a range of five (5) to twenty (20) members.

### ii. Board Composition

Board composition is important to performance as a mechanism to monitor and control management, thus maximizing shareholders' wealth and minimizing agency costs (Fama and Jensen, 1983; Jensen and Meckling, 1976). A board is composed of executive and non-executive members, of which some of the latter are designated as independent directors. While executive members represent management, non-executive members are outsiders expected to filter behavioural tendencies and decisions considered against the interest of the shareholders, thereby mitigating agency problems and helping to bolster firm performance (Subrahmanyam, Rarigan, and Rosensteing, 1997; and Sanda, Garba and Mikailu, 2008). Executive directors are prone to being conflicted because of their dual responsibility, to the CEO and the shareholders, and therefore may not be as effective in monitoring the CEO as much as the outside directors. CBN provides that "the Board shall consist of executive and non-executive directors, and the number of non-executive directors shall be more than that of executive directors''.

### iii. Independence

Independence of directors, and the board is considered imperative in corporate governance. There is however a debate on what independence truly means, and how it may influence performance. According to the Official Journal European Union (2005), a director should be considered independent "only if s/he is free of any business, family or other relationship, with the company, its controlling shareholder or the management of either, that creates a conflict of interest such as to impair his judgement". CBN defines independent director as a director who does not represent any shareholder interest and holds no special business interest with the bank. In Nigerian practice however, directors are nominated based on recommendations of existing members, and are required to represent certain bloc of interest – be it shareholders, regulators, institutional investors, or ethnic group etc. While some non-executive directors have business relationship and financial ties to the firms outside the sitting allowances and emoluments, some others (independent non-executive directors) are truly independent of such ties (Vafeas, Waegelin, and Papamichael, 2003). The former would therefore likely be less effective at monitoring the management, than the latter. It may be argued that outsider directors that are truly independent may need to own the firm's shares (Subrahmanyam *et al.*, 1997; and Hambrick and Jackson, 2000) in order to align the shareholders and managers' interests. Yet,

holding large shares may encourage moral hazard, in which the directors expropriate resources or act to serve their own personal interests at the expense of other shareholders.

Independence is believed to be an enabler of firm performance because it facilitates a non-biased decision-making process (Zubaidah, Nurmala and Kamaruuzaman, 2009), and decreases related-party transactions (Dahya, Dimitrov and McConnell, 2008). Pathan (2009) opined that independent directors are expected to be more concerned about protecting their own reputation and would therefore not allow actions that may jeopardise the interest of the shareholders. Independence becomes important especially in a banking industry where depositors and debt-holders control the largest percentage of deposits and liabilities, and yet have no representation on the board. Independent directors would therefore be expected to focus more on proper monitoring rather than short-term objective of share price movement (Devriese, Dewatripont, Heremans, and Nguyen, 2004). As outsiders, they are more likely to protect the interest of the shareholders in monitoring management (Adams and Mehran, 2012), feel less obligated to management, and in addition offer fresh and varied ideas at board meetings.

Determining a mechanism for measuring independence may be tricky, but some of the measures in use include frequency of replacement of CEOs based on poor performance (Udueni, 1998; Liang and Li, 1999); involvement of directors in other boards (Shivdasani, 1993); ratio of outside directors (non-executive directors) to executive directors on board committees (Hayes, Mehran and Schaefer, 2004), and the number of outside directors appointed during the tenure of the CEO (Ghosh and Sirmans, 2003). In other instances, board independence is determined by gender or ethnic diversity of members, relationship or alignment of non-executive directors with a major shareholder, or an associated company owned by either the same principal or major supplier/creditor (in firms in which they are non-executive directors). CBN (2014) only provides that the Board of banks shall have at least two (2) Non-Executive Directors as Independent Directors. Members of the Board shall be qualified persons of proven integrity and shall be knowledgeable in business and financial matter.

### iv. Committee Composition and Membership

The board of directors carries out its functions mostly through its various committees. Board committees in Nigerian banks typically include legally required committees such as statutory audit, risk management, nomination, governance, remuneration, and audit committees. Banks are however at liberty to set up other committees deemed relevant to their business objectives, for instance information technology and strategy, credit and finance, human resources etc. The board chairman is excluded from sitting on any of the committees, whilst non-executive directors chair the various committees supposedly for independence. Sun and Liu (2014) established a relationship between the effectiveness of the audit committee and risk-taking.

### v. Board Diversity

Board diversity covers different aspect of the board structure such as gender, age, education, experience and ethnicity mix. It is expected that a diverse board offers several benefits such as increased creativity, diverse views, rich experience, improved access to information, robust decision-making process, and a positive signal to employees and the society as a firm that values diversity. It also serves as a checkmate against the domination of the board by a significant group. As a corporate governance tool, board diversity reduces propensity for ‘groupthink’, enhances thorough scrutiny of management performance, and improves output of the board deliberations. Olaoti (2016) found ethnicity of board of directors to have a positive and significant impact on Nigerian deposit money banks’ financial performance.

### vi. Board Effectiveness

Effective board functioning measures how “board members cooperate to exchange information, evaluate the merits of competing alternatives, and reach well-reasoned decisions” (Forbes and Milliken, 1999). Effectiveness is measured by various means – experience, ‘busyness’, expertise, quality, and board committee functions among others. The post-consolidation era of Nigerian banks led to the emergence of big banks with sizable capital base, which in turn has created the opportunities for them to execute oftentimes opaque and complex banking transactions. To monitor the executive board, the non-executive members need to have a deep knowledge of banking transactions, finance, financial investment and risk management,

as their jobs are now more difficult (Mehran, Morrison, and Shapiro, 2011; Minton, Taillard and Williamson, 2010).

Results on influence of financial expertise on performance are mixed. Aebi et al. (2012) and Minton et al. (2010) found that financial expertise is negatively related to stock market performance and changes in overall firm value, while Fernandes and Fich (2009) reported a significant positive relationship between financial expertise and stock performance. Minton et al. (2010) opined that when there is no crisis, external financial experts on the board are more prone to higher risk-taking and performance, hence the larger stock losses suffered during the financial crisis.

#### 2.1.14.2 Management Structure and Incentives

The Chief Executive Officer (CEO) embodies the headship of executive management, and also the agent representing the shareholders in what is called the principal-agent relationship in corporate governance studies. His or her interests are assumed by agency theorists to be mostly at variance with the owners' interests, and in order to maximise the shareholders' values it is recommended that offering managerial incentives (that guarantee the CEO's tenure, remuneration, and share ownership) and recognising a prerequisite of diversity features (such as gender, education, experience, age, and quality) would align the agent's interest with the principal's (Jensen and Meckling, 1976).

#### 2.1.14.3 Ownership Structure

Ownership structure is defined by the ''distribution of equity with regard to votes and capital and also by the identity of equity owners'' (Mathiesen, 2002). The ownership structure in a firm has implications for performance. For instance, the ownership type determines dividend policy; managers' incentives; firm's efficiency; independence; corporate strategy and performance; and attitude to expropriation of assets (Wahl, n.d.). Monks and Minow (2008) identified elements of ownership to include the right of the owner to use a property as s/he wishes; right to transfer property rights to others on own terms; and right to regulate anyone else's use. Ownership in literature is more commonly viewed from a few dimensions – ownership concentration, ownership structure, or ownership type. Concentrated Ownership

refers to the case where large equity ownership is held by few owners (controlling or significant shareholders) compared to the outstanding shares of the firm, while diffused ownership represents ownership of the firm by several small shareholders. Large equity ownership is expected to serve as an incentive for shareholders to exercise greater managerial monitoring with a view to maximising shareholders' interests. In some instances, any single shareholder having five percent (5%) or more of the voting rights of a company is considered a significant shareholder. Public/listed companies are required to disclose information of such significant shareholding in their annual reports. It is however different from 'proxy five percent (5%)' where some shareholders come together to reach 5% stake in order to force a motion at a board meeting. Other forms of ownership categorisation are Top 1, Top 3, Top 5 largest shareholders, management share ownership, block-holder, institutional investors, public ownership, private ownership, foreign ownership, and insider owners (Ozili and Uadiale, 2017; Rahman and Reja, 2015; and Wahl, n.d). Institutional investors are businesses holding and managing assets either for themselves or for their customers. They may be holding companies, pension funds, banks, mutual funds, trust funds, insurance company, or investment companies. They tend to buy and sell large blocks of a company's shares within a short time frame, and are sometimes represented on the board. Their role in corporate governance and performance is important, because they are able to monitor the board and bring valuable advice and expertise according to La Porta *et al*. (2000), thus also protecting the interests of outsider owners. Fung and Tsai (2012) established a positive effect of institutional ownership on firm performance.

Ownership concentration is important in corporate governance studies because countries with weak investor protection tend to promote ownership concentration. Concentrated ownership has the benefits of having well-informed shareholders, who can also exercise their voting rights more appropriately in their interests. On the downside, they may be motivated to amass private benefits to themselves against the interests of the minority shareholders (Shleifer and Vishny, 1997; Johnson *et al*., 2000, Oman, Fries and Buiter, 2003, and Mork and Yeung, 2003). They may also be motivated to take high and excessive risks since shareholders benefit on the successful risky transactions, whereas depositors and other fund lenders to the banks share the costs of failure such as loss of their deposits. It is believed that a diffused structure is costlier for the firm because of the difficulties of monitoring the managers (John and Senbet, 1998).

Diffuse ownership also suffers other problems – lack of monitoring expertise and free-riding. Caprio, Laeven, and Levine (2007) opined that the regulatory environment and legal investor protection status for shareholders of a country may strongly influence the ownership structure. Weak legal protection and weak regulations lead to lower diffused ownership structure and vice-versa.

Bank consolidation led to the emergence of a new ownership structure and control in the Nigerian banking industry. It forced a change in mind-set from owning one hundred percent (100%) of barely nothing (by way of family banks) to having a bite of one percent (1%) of something, and dealing with the loss of status symbol of being referred to as chairmen or directors (Soludo, 2004). There are mixed results on the relationship between ownership structure and firm performance, while it remains unresolved if concentrated ownership reduces agency cost and therefore enhances firm value. Literature accounts for some of these inconsistencies on the measures of performance adopted (accounting versus market-based measures), the corporate governance model in place (Bhasa, 2004) in the country of study, development status of the country (Vinten, 2002; and Sarre, 2003), contextual differences (Udayasankar and Das, 2007) and the theoretical lens of study (Turnbull, 1997).

### 2.1.14.4 Ethical Leadership

Brown, Trevino, and Harrison (2005) view ethical leadership as ‘‘the demonstration of normatively appropriate conduct through personal actions and interpersonal relationships, and the promotion of such conduct to followers through two-way communication, reinforcement, and decision making’’. At the root of the corporate governance problem – i.e. dealing with separation of owners and managers, and the information asymmetry – is the question of ethics and personal values. An ethical person – be it the shareholder, manager, employee, customer, director, or other stakeholder would be expected to take actions judged to be appropriate, acceptable and responsible towards society and the relevant stakeholders. These values include humanity, justice, responsibility, and moderation (Eisenbeiss, 2012). An ethical manager for instance would not be involved in insider trading, conflicted interests or other questionable practices. Similarly, the board should neither be seen supporting the firm’s operational strategy knowing it is damaging to the environment or destructive to other stakeholders, nor using the

information at its disposal to gain preferential treatment over the shareholders. There is however a debate about the mutual exclusivity of business ethics and firm economic performance (Groom, 2011), which was examined in a study on CEO ethical leadership and firm performance by Eisenbeiss, Knippenberg, and Fahrbach (2015). Akers (1989) holds the view that no society will successfully compete when people/organisations are dishonest, stab each other in the back, and have no trust, neither will government intervention in form of voluminous regulations to force firms to act honestly promote long-term competition. While ethical organisations enjoy good reputation, attractiveness, and employee morale, unethical organisations suffer high and unexplained consequences such as regulatory sanctions, poor image in the community, low employee productivity, and loss of customer patronage. News about corporate scandals, bank failures, expropriation of firm's resources, and financial reporting fraud still dominate the business clime despite the institution of corporate governance codes and practices in organisations. The question then arises: what may possibly be the unanswered root of the corporate governance weaknesses?

Jones and Millar (2010) reported that "subordinate acquiescence, and CEO dishonesty can be understood as constituting drivers of financial accounting misreporting". This explains some of the corporate governance challenges experienced at Enron, Parmalat etc. Corporate scandals, frauds, and immoral practices elevate the awareness of public interests in organisations, and calls to question the ethical orientation of the leadership. Unethical behaviour thus affects organisations in a negative way, with a potential for decreasing their market values. Arjoon (2005) distinguished between legal and ethical mechanisms in corporate governance. The former refers to "doing the right thing", while the latter refers to "doing things right", concluding that legal mechanisms are insufficient to build trust. Ethical leadership therefore plays important role in any organisation. Leadership at management or board level is faced with several challenges requiring making choices with attendant consequences. To transform an organisation into an effective and high-performing entity, leaders need a followership built on trust and commitment. The leader's thoughts, actions, values, and attitudes must support consistent rules and practices in every business situation in order to motivate ethical behaviour in the followership (Martinez, Ruiz, and Ruiz, 2011), thereby contributing to an ethical organisation. The critical importance of the CEO is underscored by

the Upper echelons theory (Hambrick and Mason 1984) which postulates that organisational outcomes are reflections of CEOs' characteristics, values, and (leadership) behaviours. Thus, CEOs with strong personalities and ethic-centric decision-making values cascade such values to others thereby creating an organisational ethical culture.

While a judgment on what is ethical or not may be subjective and varies among individuals and within cultures, there is at least a consensus on ethical values to consist of the principles of fairness, honesty, respect, integrity, openness, and responsibility (Casson, 2013). Although the ethical leadership of the CEO is critical, it needs to be backed up by organisational ethical program to have a significant influence on firm performance (Mayer, Kuenzi, Greenbaum, Bardes and Salvador, 2009). Ethical programs consist of documented ethical codes and policies, training, reward and sanction systems among others. Ethical leadership measures attributes of the CEO's personal ethics such as people orientation, integrity, fairness, responsibility and moderation; organisational ethical programs, and ethical culture (Kalshoven, Hartog and De Hoogh, 2011; and Eisenbeiss, Knippenberg, and Fahrbach, 2015).

The ethical dimension of corporate governance practice is important especially in developing countries where corruption, mismanagement and unethical practices remain a major concern (Boadu, 2013). There seems to be a culture of disdain for ethics wherein employees are able to explain away their non-action in reporting unethical practices. They rationalise such deviance in one of four ways: 'it's a standard practice', 'it's not a big deal', 'it's not my responsibility', or 'I want to be loyal' (Gentile, 2010). Ogechukwu (2013) identified some of the unethical practices in Nigerian banks to include tampering with customers' accounts, foreign exchange malpractices, frauds and forgeries among others. Enofe, Ekpulu, Onobun, and Onyeokweni (2015) attribute bank customers' dissatisfaction to unauthorised/spurious charges, insider-related dealings, and deceptive marketing activities, which affect the perception and patronage of the public towards banking services (Al-Faki, 2006). In the light of ethical lapses being a reflection of systematic weaknesses in governance, Ferrarini (2017) recommended future research on governance of financial institutions to extend to the role and impact of ethics in order to understand the 'informal constraints' influencing corporate behaviour and also to complement their regulation and supervision.

#### 2.1.14.5 Regulation

Regulation in banking is generally defined as the control over the creation, operation, and liquidation of banks (CentralBanksGuide, 2015). It goes hand in hand with supervision, which is the implementation and monitoring of the regulation and rules. Fulbet (2008) categorised the objectives of regulation into "preventing systemic risk, providing protection for investors, enhancing efficiency, and improving the social welfare". The global financial crisis in 2008 was largely attributed to the failure of corporate governance, hence regulation and supervision have been enhanced both as a complement to the corporate governance of financial institutions and as a substitute (Ferrarini, 2017). Intervention in form of regulation is considered justified to correct market imperfections, achieve stability, and an efficient and equitable use of resources. The need for regulation stems from the nature of activities and transactions, and the critical role played by banks. They support the national economy by providing external financing for individuals and companies. Regulation is therefore considered as an external governance mechanism complementary to internally self-managed controls.

In its simplest form, a deposit money bank collects short term funds from depositors and gives out term loans to borrowing customers. The bank's income comes from the interest rate spread between the deposit and loan activity. This transaction dynamic however creates a problem of its own. On one hand, a mismatch between the loan and deposit tenor may result in 'bank run' if all or a majority of the depositors approach the bank to withdraw their funds at the same time. On the other hand, the banks have to do a balancing act managing the conflicting interests of the shareholders and the debt holders (depositors). While the shareholders are risk takers, the debt-holders are risk averse, and therefore the agency theory objective of maximizing shareholders' value is not suitable for banks (Mullineux, 2006).

Considering all these factors, governments (through the regulators) have a responsibility to prevent bank failures, and have therefore always come up with legislations or regulations to protect the industry, protect the customers from exploitation, achieve social objectives such as financial stability (Llewellyn, 1999), financial inclusion and financial innovation. Regulation of banks is carried out through several means such as capital requirement, restrictions on entry/exit into/from banking, restrictions on bank activities, transparency and disclosure

requirements, price controls, provision of deposit insurance systems; and supervision. These regulatory mechanisms are discussed below:

**i. Capital Requirements**

Banks are required to maintain a minimum level of capital considered sufficient to serve as a cushion against potential losses resulting from bank failure, and/or minimize probability of default (Bartholdy, Boyle, and Stover, 2003). Opinions are varied both from empirical and theoretical perspective as to the effect of regulation and supervision on banks' efficiency and risk appetite (Behr, Schmidt and Xie, 2010).

**ii. Activity Restrictions**

This is a regulatory mechanism through which the regulator (CBN in Nigeria's case) prescribes an indication of the extent to which deposit money banks may or may not engage in non-lending services such as owning shares in non-financial firms, securities, real estate investment, insurance underwriting and selling, underwriting, brokering and dealing in securities and all aspects of the mutual fund industry. It is argued that engagement in these non-lending activities increases the incentives for managers to engage in excessive risk-taking (Laeven and Levine, 2009). A counter-argument however suggests that universal banking provides more opportunities for banks to expand their operations thereby improving their revenue, and diversifying their risks (MacDonald and Kock, 2006). With the reforms by CBN, the Nigerian banking industry disengaged from Universal banking orientation to a more structured banking system, in which deposit money banks were required to focus on core banking and transfer (where feasible) the services to other subsidiaries in the holding companies or cease their provision. Activity restrictions may affect a bank's efficiency by not allowing banks to enjoy the benefits of economies of scale or being competitive (Barth, Caprio and Levine, 2003).

**iii. Entry/Exit Restrictions**

Government regulations on entry and exit restrictions are generally aimed at reducing competition, protecting domestic banks, and reducing foreign exchange risk and capital flight

risk. Entry restrictions can be in form of minimum capital requirement, or restriction of foreign investment or ownership (Fulbert, 2008). Nevertheless, banks entry restrictions have their benefits, especially in the case of foreign banks. They enhance competition, and sometimes implement best practices from their home countries.

**iv. Deposit Insurance Corporation**

These are set up to insure potential losses by depositors in the event of a bank failure, bankruptcy, or regulatory-induced closure. The Nigerian version is called the NDIC, which also operates as a regulator in the banking industry. Opinions are mixed about its effect. Studies (such as Bartholdy, Boyle and Stover 2003; Fulbert, 2008; Leaven and Levine, 2009; and Chu, 2011) see deposit insurance scheme as counter-productive because it has a potential for increased moral hazard incentives, excessive risk-taking and managerial opportunism. This is because managers know that depositors' losses resulting from bank failures would be redeemed by the deposit insurance corporation. Depositors also have a weak incentive to monitor the banks, thus negating the market discipline mechanism assumed under the agency theory.

**v. Disclosure Requirements**

In order to promote transparency, regulators require operators to adopt prescribed financial accounting standards and provide a range of information to enable the public make an informed decision about the banks. This is quite important in the banking sector considering the level of information asymmetry that is attributable to the volume, complexity and opaqueness of daily banking transactions.

**vi. Supervision**

CBN and NDIC carry out supervision function through on-site and off-site examination of banks' records. The objective is to ensure compliance with regulations, discourage operators from engaging in excessive risk-taking, and assuring the quality of information provided by banks on their level of risk-taking (Barth, Caprio, and Levine, 2004; Fulbert, 2008). Supervision by regulators helps the monitoring role of the principal-agent discourse by mitigating against corrupt practices inherent in lending. It also enhances banks' financial

intermediation role (Beck, Demirguc-Kunt and Levine, 2006). Other authors such as Becker (1983), and Shleifer and Vishny (1989) however advance an argument likening the supervisors' roles to those of large shareholders who may use their powers for private benefits to expropriate the banks' resources and therefore bring to bear a negative influence on performance.

While regulation and supervision is mostly attributed to the financial stability objective, it also has a role to play in financial innovation and social responsibility. The financial crisis of 2008 circa was partly attributed to the 'innovative' products developed for the mortgage sector such as securitisation, which was not properly understood by the practitioners, neither was there a robust regulatory framework to manage the risks. The regulation and supervision of financial activity was blamed for its weakness in managing the financial innovations of credit transfer on financial system stability' (Baicu, n.d). The introduction of specialised product/instrument such as collateralised debt obligations, credit default swaps increased the financial crisis risk' (Llewellyn, 2010). In another vein, economic regulations restricting the activities of banks, entry and exit conditions, price controls or setting a high standard of compliance serve as incentive for banks to innovate (Stewart, 2010). Likewise, social regulations are enacted to compel banks to behave in a socially responsible manner, such as those enshrined in CBN's principles of sustainable banking.

### 2.1.14.6 Agency Mechanisms

Agency mechanisms are specific corporate governance mechanisms prescribed by the agency theorists to solve agency problems between the principal (business owners) and the agent (managers). Agency problems and conflicts arise because of discrepancy in interests. While owners want to maximise the firm's market value, managers want to maximise their own individual interest at the expense of the owners. Some of the ways by which managers pursue their personal interests at the expense of the owners include: theft of the company's assets, excessive managerial compensation, manipulation of earnings, consumption of perks, and empire building (Mueller, 2006). In order to manage these problems and conflicts, certain costs called agency costs must be borne by the owners. They include managerial incentives, monitoring costs, and residual loss, all aimed at aligning the interests of the managers to those

of the shareholders (Jensen and Meckling, 1976). For this study, the managerial incentives considered include: CEO tenure, CEO remuneration, and Insider ownership, whilst monitoring costs include Board meetings, and the diversity mechanisms of Board ethnicity, Board gender, and CEO age. Each mechanism is discussed in further details below.

**i. CEO Remuneration**

To align the interests of managers with those of shareholders, executive compensation has been used to motivate the CEOs to maximize the shareholders' wealth or value. It is expected that managers hoping to receive some form of compensation based on performance will be highly motivated to achieve and or exceed the set targets. Components of CEO remuneration generally include basic pay, direct ownership of shares, bonuses, and stock options. Other benefits include golden parachutes, retirement plans, life insurance, club membership, cost of passage, or incentive plans which could be made up of cash bonuses, executive stock options, and restricted stock etc. Studies have shown mixed results on the causal relationship between remuneration and performance. While some studies showed that firm performance is dependent on the CEO's compensation (Jensen and Murphy, 1990, Kurawa and Saidu, 2014), others (Bebchuk and Grinstein, 2005; Anjum, 2010; Aduda, 2011; Sigler, 2011 Lin, Kuo and Wang, 2013; and Hussain, Obaid and Khan, 2014) found the firm size to be the most important determinant of the CEO's compensation. The latter argument assumes that CEOs are overpaid since their pay is not proportionately linked to their performance. In response, it is contended that large firms are complex to run and therefore need experienced and skilled managers, thus providing a justification for the high pay. The downside to the performance-related pay strategy however is that managers may be pushed to the point of undertaking excessive-risk transactions (Bebchuk, Cohen, and Spamann, 2010; Beltralti and Stulz, 2012; and DeYoung, Peng, and Yan, 2013). Allen and Gale (2000) posited that the motivation for excessive risk-taking is high because the gain from good performance far outweighs the penalties for poor performance.

Zalewska (2016) argued that the use of remuneration to reduce agency problem is sub-optimal for the banking sector because of the conflict of interests between the shareholders and other stakeholders, and therefore suggests that regulators should play a role in balancing "short-term

performance with the long-term needs of society". Sigler (2011) in his review of various forms of executive compensation among US firms noted that each compensation component has a merit and demerit. For instance, cash bonus may encourage manipulation of accounting records; stock ownership may not be a strong incentive since the CEOs have little or no impact on daily share price as dictated by the market, whilst long-term incentive such as restricted cash may not tie the CEO's loyalty to the firm's performance. Consequently, firms should employ a mixture of different compensation components to allow the "shortcoming of one component to be offset by the strength of another". Perhaps in order to allay the fears of financial instability which may result from excessive risk-taking contingent on performance-based compensation, the revised corporate governance codes of CBN (2014) provides that:

> *Where stock options are adopted as part of executive remuneration or compensation, the Board shall ensure that they are not priced at a discount except with the authorization of the relevant regulatory agencies…. Share options shall be tied to performance and subject to the approval of the shareholders at AGMs…. Share options shall not be exercisable until one year after the expiration of the tenure of the Director.*

**ii. CEO Tenure**

CEO tenure is an agency governance measure to protect the interest of the shareholders. There are different arguments on the expected influence of tenure on firm performance. On one hand, the longer an executive director stays in a position, the more of firm and industry knowledge s/he is expected to acquire (Baysinger and Hoskisson, 1990), which should lead to an improvement in the firm performance. However, others argue that hiring a new CEO may improve the firm performance (Huson, Malatesta, and Parrino, 2004). While a performing CEO is rewarded with retention in office through renewal of his/her contract, poor performers are prone to being sacked by the Board. It is also argued that a guaranteed tenure provides job security to the CEO, as well as the benefit of being able to see the outcome of implemented strategies (Kyereboah-Coleman, 2007). On the contrary, long tenure tends to promote hegemony, which may be counter-productive to the shareholders' interest. The key objective should therefore be to determine the optimal tenure of the CEO that maximizes firm performance. CBN provides that the CEO's tenure shall be in accordance with the terms of engagement with the bank but subject to a maximum period of ten (10) years. Such tenure may be broken down into periods not exceeding five (5) years at a time.

**iii. CEO Age**

Age is assumed to correspond to experience, as the older an executive gets, the more business experience he or she gains which stands as a competitive edge. It is also assumed that young executives may not have sufficient experience to navigate the rough terrain of businesses. On the other hand, it may be argued that younger executives are more likely to be energetic, and risk-takers focusing on immediate goals in order to build a reputation (Hirshleifer, 1993), while Bertrand and Schoar (2003) suggest that older executives are more conservative in their work. There is no provision for age restriction in CBN's code of corporate governance.

**iv. Insider Ownership**

Jensen and Meckling (1976) posit that insider owners are motivated to pursue their own interests, and this is in turn aligned to enhance firm value, hence the level of insider ownership should have a linear relationship with a firm's value and its performance. Insider owners thus have the benefit of lowering the agency costs between managers and shareholders. Aebi *et al*. (2012) opine that it provides incentives to bank CEOs to maximize bank value and limit the bank's risk exposure. Morck, Shleifer, and Vishny (1988) argue that insiders are motivated to take ownership stake for two reasons – to help in aligning managers' interests with those of shareholders (Cornett, McNutt and Tehranian, 2010), as well as to protect their own jobs even in the face of inefficient performance. Empirical findings are however mixed. While Westman (2011) found a positive relationship between insider owners (managers and directors) and bank performance in a study of European countries, Demsetz (1983) found inverse relationship between firm performance and increasing insider ownership. This was supported in another study by Iturralde, Maseda and Arosa (2011) who found the optimal insider ownership level to be between 0-35 percent for improved firm performance, beyond which performance begins to decline due to the entrenchment risk.

Reforms in the banking sector have led to change in the ownership structure of Nigerian banks. Before the banking consolidation exercise, which required banks to increase their capital base from the previously held minimum of ₦1 billion to a new floor level of ₦25 billion, most banks were private, family-owned. The reform necessitated seeking capital injection from external sources, which thus led to a dilution of ownership and more importantly the reduction in the

stake of the founding owners. Some banks also had to adopt mergers and acquisition strategies to meet up with the new capital base requirement. According to Soludo cited in Imeokparia (2013), the consolidation in the banking sector 'drastically altered the ownership structure of Nigerian banks, making it more widespread and diversified' thus transforming from concentrated to diffused ownership structure. Nevertheless, the hitherto founders/owners of the banks before consolidation still found a way to hold stakes in the banks through the proportion of shares held by the bank's board, which is referred to as insider ownership. CBN (2014) provides that an equity holding of 5% and above by any investor shall be subject to its prior approval. Where such shares are acquired through the capital market, the bank shall apply for a no objection letter from the CBN immediately after the acquisition.

**v. Diversity – Board Ethnicity and Gender**

The study of board diversity is receiving increased attention in developing countries. Board diversity focuses on different dimensions such as composition based on ethnicity, gender, or nationality. The agency thesis holds that a diverse board will serve as a mechanism to prevent managerial opportunism, as diversity encourages diverse opinions. Studies on gender tend to show a relationship with firm performance (Carter, Simkins and Simpson, 2003; Campbell and Minguez-Vera, 2010). Female executives are assumed to be more methodical and restrained, thus more conservative and risk-averse (Byrnes, Miller, and Schafer, 1999; Martin, Nishikawa, and Williams, 2009) and better players in decision-making tasks. Due to the implicit bias about women, they need to prove themselves more than men to attain executive posts, and therefore tend to show more responsibilities and capabilities in their high office (Eagly and Carli, 2003). In addition, women are credited with communication and group problem-solving skills (Dallas, 2002; Schubert, 2006) and cooperative leadership style (Eagly and Carli, 2003). With the aggressive expansion of Nigerian banks to other continents, diversity has become imperative.

**vi. Board Meetings**

It is expected that boards need to meet periodically to carry out their oversight function. Too many meetings make the process routine and seen by management as interference by the board on their day-to-day function, while too few meetings keep the board in the dark about happenings in the organisation. Whilst holding several meetings can improve the board's

monitoring and oversight function, it may also lead to increase in agency costs through the associated meeting expenses or diversion of bank's material, time and human resources to non-productive venture. Coleman (2008) and Ntim and Osei (2011) established a positive relationship between frequency of meetings and firm performance, while Fernandes and Fitch (2009), and Aebi et al. (2012) did not find significant effects on the relationship between board 'busyness' and performance. CBN provides that bank Boards shall meet at least once a quarter, and to qualify for re-election, a director must have attended at least two-thirds of all Board and Board Committee meetings"

#### 2.1.14.7 Governance Practices Disclosure

Disclosure is considered a key corporate governance mechanism for ensuring transparency and accountability of organisations (Okike, 2007). For agency theorists, 'disclosure' helps in aligning the managers and owners' interests as information asymmetry is lessened, whilst stakeholder theorists view disclosure as a governance principle of being accountable to all of the firm's stakeholders (Isukul and Chizea, 2017). Prospective investors consider governance practices an important criterion influencing their investment decision, and the issue of transparency is even more important for banks in the light of loss of public confidence resulting from the global financial crisis that occurred in the industry. The level of disclosure practices in developing countries is considered weak because of its underdeveloped market, culture, and weak regulatory mechanisms (Haniffa and Cooke, 2002). Disclosures can be voluntary or mandatory (Uyar, 2011). While they are mandatory in countries such as Nigeria, UK, and US, they are voluntary in India, and Asian countries (Bhasin, 2010). 'Disclosure' must meet the criteria of timeliness, adequacy, relevance, availability, comparability, and comprehension (Brennan and Solomon, 2008; Adelopo, 2011). Most literature on disclosure have laid emphasis on accounting disclosure (Oluwagbemiga, 2014) and found determinants of disclosure to include ownership type, firm size, strong financial performance, and intention to raise capital on the stock exchange

### 2.1.15 Framework for Sustainability

'*What gets measured, gets managed*' (Dillenburg, Green, and Erekson, 2003).

The justification for sustainability as a firm performance measure derives its legitimacy from the ultimate objective of a corporation not only to create value for shareholders but to also create economic, social and environmental value (Galbreath, 2012). This new approach to performance measurement moves beyond the traditional profit-maximisation objective, to an expansive goal encompassing social and environmental responsibility (Stubbs and Cocklin, 2008). Building on WBCSD (2003) definition of sustainability, Bansal and DesJardine (2014) define business sustainability as the ability of firms to meet their "short-term financial needs without compromising their (or 'others') ability to meet their future needs". This has been translated by Passet (1979) to refer to the environment, society and the economy. In its application to the banking industry, it is sometimes couched as 'sustainable banking', which refers to *"*a philosophy that underpins everything about banking, a value system that says a bank's commercial activities must not only benefit its staff and shareholders, but also its customers and the wider economy, while at the same time preventing, or at least minimizing, any undue effects on society and the natural environment*"* (Imeson and Sim, 2013; Bouma Jeucken, and Klinkers, 2001). This is a similar view to CBN (2012)'s sustainable banking definition as an "approach that recognizes the role of banks in driving long-term economic development in Nigeria that is not only economically viable, but also environmentally responsible and socially relevant".

Under the CBN's guidelines, Nigerian banks are required to adopt and implement the sustainable banking principles, which are categorised into nine (9) pillars – Business activities environmental and social risk management; business operations environmental and social footprint; human rights; women's economic empowerment; financial inclusion; environmental and social governance; capacity building; collaborative partnerships; and reporting (CBN, 2012). According to CBN,

> *sustainable banking will enhance banks' "cost and liabilities reduction, access to capital and attraction of foreign investors and partners, financial and non-financial performance, brands and reputations, operational efficiencies, talent attraction and retention, client relationship and trust, growth prospects through new markets, and financial innovation" (CBN, 2013 pp3).*

Jones, Hillier and Comfort (2017) believe that

> *while the majority of the leading financial services companies publicly emphasise their commitment to sustainability, the dominant interpretation of sustainability within the industry is built around business imperatives, efficiency and cost savings rather than any social and environmental concern.*

In view of the foregoing, sustainability construct in this study is investigated along three main categories – economic sustainability, social sustainability and environmental sustainability. In specific terms, this study conceptualizes economic sustainability to encompass *financial performance*, and *financial innovation*; while social and environmental sustainability is grouped together and analysed as *corporate social performance* and *corporate reputation*. The final consideration relates to the integration or implementation of the three dimensions under *sustainability performance reporting*. Details are discussed below.

### 2.1.15.1 Corporate Social Performance

Corporate social performance is "a business organization's configuration of principles of social responsibility, processes of social responsiveness, and policies, programs, and observable outcomes as they relate to the firm's societal relationships" (Wood 1991: 693). Hopkins (2002) defines social responsibility as 'being concerned with treating the stakeholders of the firm ethically or in a responsible manner'. The European Commission (2011) on the other hand defines it as the 'the responsibility of enterprises for their impacts on society'. This entails putting a 'process in place to integrate social, and environmental concerns into their business operations and core strategy in close cooperation with their stakeholders'. The changing dynamics of the business world is putting pressure on organisations to go beyond satisfying their shareholders by considering the interests of other stakeholders in corporate governance practices, which has dove-tailed into the study of corporate social performance of firms measured by social performance and environmental performance. Corporate social performance therefore is the outcome of a firm's social responsibility practices. It is believed that a socially responsible firm not only conforms to the legal or regulatory requirements, but gives consideration to its stakeholders in order to truly maximize the shareholders' wealth. The concept of corporate social performance is further discussed below under social performance, and environmental performance.

#### 2.1.15.1.1 Social Performance

Social performance defines the context in which organisations are expected to consider the interest of society by taking responsibility of the impact of their activities on the various stakeholders such as customers, employees, and the community among others. Social performance therefore represents “a business organisation’s configuration of principles of social responsibility, processes of social responsiveness, and policies, programs, and observable outcomes as they relate to the firm’s societal relationships” (Wood 1991). It could also be referred to as the outcome of a firm’s corporate social responsibility operations which go beyond legal or regulatory requirements (McWilliams and Siegel 2000). It entails among others, programmes and policies aimed at employee well-being, philanthropy, employment creation, customer satisfaction, product responsibility, community development, diversity, and human rights.

There are two (2) schools of thought on social performance according to Waddock and Graves (1994). The slack resource theory (Gompers, Ishii and Metrick, 2003) believes that social performance activities are only carried out with the excess funds available from improved financial performance, while the good management theory (Brammer and Pavelin, 2006) states that social performance comes first before successful financial performance. Firms engaged in social performance build a good reputation in the minds of stakeholders, and are in return rewarded with improved financial performance through market mechanism. The importance of social performance is more critical to institutional investors who demand that firms must have high levels of social performance as a pre-condition for their investments (McKinsey, 2002).

In pursuit of social performance, firms recognise that employees want a secured and safe job environment, training and career opportunities, as well as good remuneration. In fact, their livelihoods are more tied to the fortunes of their organisations compared to institutional investors who are able to diversify their risks (Vitols and Kruge, 2011). By meeting these needs, a firm also increases its value through engagement of quality employees and low labour turnover rate. The local communities and government as secondary stakeholders expect firms

to maintain a safe operational environment, contribute to provision of social amenities, and promote ethical values (Agle *et al*., 1999; Johnson and Greening, 1999).
Studies on social performance have generally examined domains in the Social Performance index (Waddock and Graves, 1997; Rogosic, 2014), which include employee relations, diversity promotion, consumer relations, community relations, human rights and product responsibility (Arora and Petrova, 2009). The CBN's sustainable banking principles identify areas for social performance to include diversity, human rights, ethics, employment and labour laws, community development, and product responsibility.

**2.1.15.1.2 Environmental Performance**

Environmental performance refers to "the outcome of a firm's strategic activities that manage (or not) its impact on the natural environment" (Walls, Phan, and Berrone, 2011). It also implies measures that provide the protection of environmental factors – air, water, soil, ecosystems. Environmental performance covers programmes and policies to control a firm's negative effect on the environment (referred to technically as environmental footprint) such as property, energy, water, waste, carbon-di-oxide ($CO_2$) emission, and promoting financing of Socially Responsible Investment (SRI) and avoiding investment activities that are environmentally-harmful.

While efforts at protecting the environment may be argued to amount to incurring higher expenses for banks, and consequently minimising the shareholders' value in the short term, the upside is the promotion of environmental efficiency, otherwise known as eco-efficiency, which in the medium to long term is value-maximising. In the main, eco-efficiency comprises of initiatives aimed at expansive use of renewable energy, minimisation of toxic materials and increased recycling. Whilst deposit money banks are not considered to be in the 'dirty industry' such as oil and gas and manufacturing, where environmental issues are prevalent due to their operations, they have roles to play in shaping environmental performance by declining loans to clients involved in environmentally-harmful business activities. Banks can also develop programmes, strategies, policies and procedures to actively reduce their own environmental impact through reduction in water consumption, energy use, paper usage, waste management, and travels, which translate to less costs, and therefore better performance and responsibility.

According to Berrone and Gomez-Mejia (2009), good environmental performance gives a firm intangible benefits such as good social legitimacy, and stakeholder satisfaction with potential for enhancing its market value, while negative performances in form of environmental crises affect the market value of companies (Klassen and McLaughlin, 1996; and Dowell, Hart, and Yeung, 2000). The board of directors has an important role in achieving a good environmental performance through its monitoring of decision-making, and resource provision roles (Hillman and Dalziel, 2003; and Villiers, Naiker and Staden, 2011). Granted that managers and board may be conflicted about pursuing high-level environmental performance because of the costs involved, which is considered a profit-reduction action, there are characteristics of the board that may encourage the firm to pursue environmental strengths and mitigate environmental concerns. Independent directors are assumed to be altruistic, concerned about doing what is right and protecting their own reputation, therefore are expected to consider the long-term financial and social interests of the firm by promoting social responsibility. Similarly, managers and executive directors with significant shareholding are amenable to environmental-initiatives investment in order to maximize the firm's value in the long-term.

A board composed of law experts, interlocking and multiple directorships, large board size, and longer director tenure (Kassinis and Vafeas, 2002; Kor and Sundaramurthy, 2009; Villiers, Naiker and Staden, 2011) is also a plus for environmental performance. Firms adopt strong environmental performance due to one or combination of factors such as the managers' strategic position (Aragon-Correa, 1998), sensitivity to stakeholder pressures (Henriques and Sadorsky, 1999), ethical values (Bansal and Roth, 2000), or availability of economic incentives (Berrone and Gomez-Mejia, 2009).

In measuring environmental performance of organisations, two (2) common approaches are adopted in literature. These are assessing the processes, or assessing the outcomes. The processes approach focuses on the efforts accomplished by the top management to influence the environmental operational performance (outcomes) of the business; as well as the policies, programs, and resources mobilized (Henri and Journeault, 2008; Brunklaus, Malmqvist and Baumann, 2009). The outcomes approach looks at quantitative indicators, measuring outcomes

such as energy consumption, material inputs, waste and emissions etc. The outcomes approach is perceived to be objective and rigorous, and empirical studies are more prevalent in manufacturing companies (Cole, Eliotta, and Stoblib, 2008; Hermann, Kroeze, and Jawjit, 2007). This approach is however not suitable for the banking sector because of several reasons. First, there is no evidence that Nigerian banks currently keep data on measures such as carbon emission, water consumption, energy consumption or paper consumption. Second, even if data are readily available on those measures, they only measure the internal impact on environmental performance, which is insignificant compared to external impact resulting from the activities banks finance. Third, can banks be reasonably and justly held accountable for environmental impact of their customers' activities? Finally, any attempt to conduct environmental impact assessment in banks would be time-consuming, costly, and methodologically challenging (Allet, 2011). In developed economies, studies make use of environmental indicators developed by reputable agencies such as Kinder, Lydenberg, and Domini's (KLD) dataset, which are not readily available for Nigerian firms.

#### 2.1.15.2 Corporate Reputation

Corporate reputation is a sum-total of the perception of an organisation by several parties both within and outside the organisation, from different perspectives (Fombrun, Gardberg and Sever, 2000; Wartick, 2002), which must be aligned, especially by the key stakeholders - employees and customers (Hatch and Schultz, 2001). Chun (2005) consequently views corporate reputation as the aggregation of how a firm sees itself ('identity') and how others see it ('image'). Barnett, Jermier and Lafferty (2006) however hold a different opinion on the interpretation of identity and image, preferring to view 'identity' as the underlying 'core' or basic character of the firm, and 'image' as "observers ' general impressions of a corporation's distinct collection of symbols, whether that observer is internal or external to the firm".

Corporate reputation is a mechanism to gain competitive advantage necessary for survival and sustainability (Ljubojevic and Ljubojevic, 2008). It is often described as the most important intangible asset (Institute of Directors, 1999), which requires internal programs support. A firm with a positive reputation finds it easier to attract good employees, customers and enhance loyalty, hence it boosts a firm's image among stakeholders (Wilderman and Buxel, 2005). The

public defines a firm with a good reputation by several indices such as the quality of its products, financial performance, social responsibility, ethical culture, and image, and the CEO's reputation has an important role to play in promoting the firm's reputation (Kitchen and Laurence, 2003). Rating agencies such as Fortune 2000, and the Financial Times use indicators such as financial soundness, social responsibility, management and products quality, and efficiency to measure a firm's reputation, and equally a set of criteria such as strategy, business globalisation, financial and corporate culture to determine the CEO's reputation (Chun, 2005).

Building a strong organisational ethical culture complemented with an ethical CEO enhance the reputation rating of firms. Reputations of leaders are considered inseparable from their firms (Graffin, Pfarrer and Hill, 2012), and there are different measures by which such evaluation is done. For instance, Love, Lim, and Bednar (2017) posit that reputation agencies and the public consider media coverage of the CEO, firm performance, social responsibility, industry awards and recognitions, and whether the CEO is seen as a 'corporate saviour' (recruited from outside the firm) or promoted internally. Corporate reputation therefore reflects an appraisal of a firm's competencies and ethical predisposition (Berens and van Riel, 2004).
Watson (2007). According to CBN, a robust and transparent governance built on ethical relationship with stakeholders such as employees, business partners, and clients helps banks to meet their business objectives and critical to their reputation as a credible, responsible institution. For the purpose of this thesis, corporate reputation is studied from the 'identity' perspective – that is how employees and managers view their firm's reputation.

#### 2.1.15.3 Financial Innovation

Financial innovation according to the European Central Bank (ECB, 2003) entails innovation to a product, organisation or a service improvement with a view to reducing cost or risk by the bank or the financial industry as a whole. It also connotes an outcome that results in new financial products, financial markets, improved process efficiency, and delivery channels among others. A similar definition offered by Frame and White (2002) views financial innovation as "something new that reduces costs, reduces risks or provides an improved product/service/instrument that better satisfies participants' demands…" within a financial

system. Whilst innovation in a manufacturing industry may be measured by the number of products, patents obtained, research and development expenditure, the banking industry is characteristically different.

Studies such as Domeher, Frimpong and Appiah (2014), Cherotich, Sang, Shisia, and Mutung'u (2015) have looked at financial innovation in the financial services/banking industry in order to identify what constitutes innovation. Financial innovation may come in form of tweaks to existing systems or products, new financial products and services, new organisational forms or a radical innovation driven by disruptive technological advancement in ICT, such as the introduction of ATMs, mobile internet, cloud computing, and risk management software etc. (Woo, 2017). Vargas (2009) identified three sources of innovation, process innovation, organisational innovation, and product innovation. Arnaboldi and Rossignoli (2015) identified six (6) major categories of innovation in the financial services industry, which are, product, delivery channel, ICT, operating systems, organisational structure, and group organisational model.

Financial innovation is largely driven by cost reduction objective, development in information technology, firm size, firm market power, unstable macroeconomic conditions, competition and financial regulations' (Baicu, n.d., Frame and White, 2002). It promotes efficient allocation of resources, customer satisfaction, and economic growth, all targeted towards sustainability. CBN (2012) requires banks to 'drive innovation for improved financial system accessibility and usage by developing innovative products and services', yet there is a relative dearth of empirical studies that test hypotheses or otherwise provide a quantitative analysis of financial innovation (Frame and White, 2002). Plausible explanations for this include traditional linkage of innovation to research and development common in non-banking industry, little or no patenting rights in finance industry, and lack of precise financial innovation data useful for information.

### 2.1.15.4 Financial Performance

Financial performance refers to ''conclusions drawn from financial analysis of a firm, via the selection, evaluation, and interpretation of financial data, along with other pertinent

information, to assist in investment and financial decision-making'' (King'ang'ai, Kigabo, Kihonge, and Kibachia, 2016). The financial industry crisis in 2008/9 brought out the need for a more detailed view of what constitutes financial performance of banks. New accounting norms (such as IFRS, GAAP) were introduced to ensure a stricter but commonly applied principles to the treatment of financial reporting. In addition, the financial statement is of use to several stakeholders, not just the shareholders. Prospective investors are interested in appraising a bank's ability to earn future growth and remain sustainable, government is interested in tax income, customers are interested in the safety of their deposits, and regulators are interested in financial stability and so on and so forth. This therefore justifies the need for financial performance study to be as broad as possible. The uniqueness and peculiarities of the banking industry relative to non-banking industries, also makes the need for adoption of a distinct approach to understanding their financial performance (Macey and O'hara, 2003).

Financial performance of banks is broadly categorised into financial (accounting ratios) and market measures. Typical market measures include Tobin's Q, Price Earnings ratio (P/E ratio), Earnings per Share, Dividend Per Share. Accounting ratios are however further categorised into other groups, such as: (i). Profitability ratios – focus is on profit of the banks, and typical indicators include ROA, ROE, and net interest margin. (ii). Liquidity ratios – important that banks are able to meet their financial commitments and avoid distress. Typical measures include loan-to-deposit ratio, and loan to asset ratio. (iii). Risk and solvency ratios – useful to determine the potential to go into bankruptcy or financial distress. Typical ratios include non-performing loan ratio, debt-to-equity ratio, debt-to-asset ratio, and Z-score. (iv). Efficiency ratios – determine the rate at which a bank converts assets to income-yielding opportunity, the use of little resources to achieve greater value. Typical ratios include cost-to-income ratio. Most studies on corporate governance adopt ROA, ROE, Tobin's Q in the study of financial performance, with only a few (such as Akpan and Riman, 2012, Nyor and Mejabi, 2013, Islam, 2014) recognising the peculiarity of the banking sector and the need for other measures such as non-performing loan, and net interest margin. A preponderance of studies tends to use financial performance, with accounting-based metrics (Combs, Crook, and Shook, 2005; Carton and Hofer, 2006; and Richard et al., 2009), and, or market-based metrics such as Tobin's Q, Earnings per Share, Dividend Yield (Brown and Caylor, 2006; Bhagat and Bolton,

2008; and Mnasri, 2015) among others to measure performance. This study adopted ROA, ROE, TBQ, NIM, and NPL.

#### 2.1.15.5 Sustainability Performance Reporting

Global Reporting Initiative (2011) defines Sustainability Performance Reporting as "the practice of measuring, disclosing, and being accountable to internal and external stakeholders for organizational performance towards the goal of sustainable development.", whilst WBCSD, (2003) similarly defines it as public reports published by companies for the benefits of the internal and external stakeholders with a picture of the corporate position and activities on economic, environmental and social dimensions. Sustainability reporting provides a foundation for improving performance, "preserving and enhancing value of firm through various strategic benefits" such as – reputation protection and enhancement, committed employees, stakeholder recognition, access to capital and license to operate (Warren and Thomsen, 2012). Sustainability reporting is used in literature interchangeably and synonymously with terms such as sustainable performance, corporate social responsibility reporting, sustainable development, sustainable growth, and triple bottom line reporting (Roca and Searcy, 2012, Elkington, 1998). Common to all the terms is the integration of the balance of three pillars representing economic, social and environmental pillars (Kocmanová, Hřebíček, and Dočekalová, 2011).

The need for sustainability is encapsulated in the principles of accountability, legitimacy, stakeholder relations, and information asymmetry (Aggarwal, 2013). Organisations are under pressure to be accountable, while the quest for legitimacy constrains an organisation from breaking the law, or taking actions that would lead to boycott by stakeholders. In addition, firms need to maintain good relations with their stakeholders, failure of which may lead to negative press and poor reputation. Agency problem is mostly concerned about resolving information asymmetry problem, which arises because the agent knows more about the company's performance and policies than the principal and other stakeholders, for which sustainability reporting is an antidote. Different stakeholders with differing interests are concerned about how to evaluate the performance of an organisation, which cannot be largely explained by the traditional financial metrics. In understanding how a firm's value is thus

created, destroyed or sustained, it is imperative to consider both tangible and intangible measures. A firm's value must therefore be linked to tangible measures such as physical net assets, and intangible measures such as innovation, customer and employee relations, supplier relations, and reputation (Warren and Thomsen, 2012).

Publication of sustainability reports by companies started in the late 1980s (Wiedmann, Lenzen, and Barrett, 2009), while Nigerian banks started publication of sustainability reports in their annual reports from 2013 financial year-end. Some of the common methods for measuring and reporting sustainable performance in literature include:

i. Analysts' ratings by agencies such as Global Reporting Initiative (GRI) – The GRI index measures economic, social and environmental information in organizations through several indicators grouped along six dimensions covering: Economic, Society, Environment, Labour, Product Responsibility, and Human Rights, after which a sustainability report is generated for participating organisations.
ii. Self-constructed Index – Involves developing sustainability indices by extracting sustainability indicators from annual reports using content analysis.
iii. Hybrid method – combines the first two approaches by identifying appropriate indices from the rating agencies, and combining with extracts from companies' annual reports and websites to develop a sustainability index.

Indicators obtained in approach (ii) and (iii) are scored either by binary coding, weighted scoring, or descriptive reporting. Binary coding (as used in Ameer and Othman, 2012; and Backstrom and Karlsson, 2015) scores a company 1 if an item is disclosed in the annual report, and 0 if not. Weighted scoring method (as used in Rebai-Bouricha, 2014) assigns weights based on importance of the evaluated item, and descriptive reporting as used by Kumar (2013); and Weber and Oni (2015). Measuring sustainability is not without its limitations as pointed out by Mayer (2008), and this includes the data definition, methodological appropriateness, and standardisation, yet more than eighty percent of top two-hundred-and-fifty biggest multinationals follow the GRI method (Oliveros, 2011).

### 2.1.16 Corporate Governance Dimensions and Sustainability: A Conceptual Perspective

Debate continues to rage on the link between corporate governance and sustainability. Friedman (1970); and Brown, Helland, and Smith (2006) opined that incorporating social and environmental policies in business practices erodes the value due to the shareholders. It is their contention that this amounts to another form of agency cost, for which only the managers enjoy private benefits in form of good reputation while the shareholders suffer as a result of the additional costs to business operations. Jensen (2001) alluded to this argument by stating that firms that adopt social and environmental practices models will either be eliminated by competitors or survive by eating into their own value.

Porter and Kramer (2011) however logically argued that meeting the needs of other stakeholders such as customers, and employees among others, creates value for shareholders. Conversely, there is a value destruction for non-sustainable practices which would lead to higher costs to the firms in form of low productivity from disgruntled employees, products and services boycott from dissatisfied customers, punitive penalty and fine payments to government, and business disruptions by a dissatisfied community.

The banking industry provides space for economic activities, but its operation also affects the social and environmental space. Literature is replete with cases of collapsed and failed banks resulting from poor corporate governance practices, which suggests logically that corporate governance at the least contributes to survival of businesses. What is however in contention is the business case for sustainability. Schaltegger and Ludeke-Freund (2012) posited that there are direct and indirect drivers of sustainability – cost and cost reduction, risk and risk reduction, revenue and profit margin, reputation and brand value, attractiveness as employer, and innovative capabilities. As banks pursue environmental and social performance initiatives, they are able to reduce their energy, water, material and waste costs. In a similar vein, supporting firms producing ‘green’ products and services create opportunity for additional revenue stream and profit, whilst promoting employee wellbeing, and innovation boosts a firm’s reputation and consequently its sustainability. In recognition of the importance of sustainability, the CBN introduced the ‘sustainable banking principles’ in 2013, which is an

"approach that recognizes the role of banks in driving long-term economic development in Nigeria that is not only economically viable, but also environmentally responsible and socially relevant" (CBN, 2012). This is the classical definition of sustainability, measured along the triple bottom-line dimensions of economic, social and environmental performance of a firm (Elkington, 1997; Nidasio, 2006; Rudden and Rudden, 2012) with a view to thriving into the future. Economic performance (sustainability) measures items conventionally reported in the financial statements and annual report e.g. profitability, growth, market value, financial innovation etc. Social performance (sustainability) encompasses the impact of companies on community development, employee well-being, customer satisfaction, corporate reputation, diversity, product responsibility, financial inclusion, ethics, and human rights among others.

Environmental performance (sustainability) covers programmes and policies to control a firm's negative footprint on the environment, promote positive environmental practices, and finance socially responsible investments. Eccles, Ioannou and Serafeim (2014) posit that firms engaged in sound corporate governance practices with consideration of all stakeholders in their business strategies gain economic benefits in terms of profitability, and stock market performance, but more importantly benefits considered as intangible values which give a firm competitive advantage such as brand value, employees' and customers' satisfaction, community development, reputation, and regulatory compliance.

Corporate governance contributes not only to corporate prosperity, but also to responsibility (Kocmanova, Hrebicek, and Docekalova, 2011). Sound corporate governance practices protect not only the shareholders, but also other stakeholders. For shareholders, prosperity is achieved through economic efficiency. As businesses continue to pursue growth, the need for financing becomes imperative, and the trend in modern business world is a prerequisite for firms seeking capital (through investors, banks, and international capital markets) to make a full disclosure of the state of their corporate governance and social responsibility practices in order to attract funds. Stakeholder relationships impact an organisation's objectives, hence are critical to the survival of an organisation. Effective stakeholder relationships are based on trust, which is best facilitated by corporate governance (Stuebs and Sun, 2015). Organisations such as the OECD, the UN Global Compact, the IFC (World Bank Group) among others "encourage firms to

integrate social aspects in their governance agenda and recognize that a company's environmental, social, and governance responsibilities are integral to its performance and long-term sustainability" (Walls, Berrone, and Phan, 2012). The management of a firm's economic, social and environmental performance therefore leads to "corporate sustainability, whose objectives include long-term growth, efficiency performance, and company competitiveness" (Kocmanova, and Docekalova, 2011).

The following sections create a linkage between the variables of corporate governance dimensions and those of sustainability adopted for this work.

#### 2.1.16.1 Internal Governance Controls and Corporate Social Performance

"Corporate governance is concerned with holding the balance between economic and social goals and … to align as nearly as possible the interests of individuals, corporations, and society" (Cadbury, 2003).

Harjoto and Jo (2011) in a classification of the link between internal governance control measures (of the board, management and ownership) and corporate social performance, reviewed four reasons for firms' engagement in social responsibility – (i). to build their own personal reputation as good global citizens (Barnea and Rubin, 2010); (ii) to rely on social and environmental activists to protect the CEO's job (Cespa and Cestone, 2007) ; (iii) to signal or differentiate a firm's products especially through advertising intensity in a highly competitive market (Fisman, Heal, and Nair, 2006); and (iv) to reduce conflict of interests between managers and stakeholders (Calton and Payne, 2003; Scherer, Palazzo and Baumann, 2006). Therefore, firms with effective corporate governance are more likely to engage in social responsibility to enhance stakeholders' relationship and therefore achieve higher firm value and performance.

The role of corporate governance (internal governance controls) is also important in environmental performance. On one hand it requires a capital outlay which may have long-term strategic implications, whilst on the other hand it requires alliances with stakeholders within and outside the organisation (Walls, Berrone, and Phan, 2012), thus calling for the

support of the owners, managers and the board. Nigerian banks are required by the sustainable banking principles to focus on areas of their direct and indirect impact on the environment. Directly, the banks are required to introduce initiatives to reduce their resources consumption, such as energy, water, and materials (paper). They are also required to reduce pollution prevention through effective waste management, reduction of carbon emission by minimising air pollution arising from use of generator sets and car exhaust pipe fumes. The most significant impact on the environment however comes from the banks' customers who engage in oil and gas, manufacturing, and other extractive industries. The banks' role is to ensure that they have a robust credit appraisal system that reviews the environmental impact assessment (EIA) of each project and declines loans and credit requests from customers that engage in harmful environmental practices (such as environmental pollution, and degradation). They are also required to integrate environmental awareness and considerations in their supply chain management. In addition, banks are expected to actively support customers engaged in 'green' products and services through socially responsible investment (SRI) financing. Organisations that pay attention to environmental considerations gain on cost initiatives, avoid recriminations, and earn a favourable reputation.

### 2.1.16.2 Ethical Leadership and Corporate Reputation

Ethical leadership ensures that corporate behaviour rubs positively on shareholders, stakeholders and the whole community, which is critical to long-term survival. Corporate reputation is an intangible asset that confers competitive advantage on firms. This is more so in modern business world where firms are faced with stakeholder pressure and increasing scrutiny from regulatory authorities/bodies. The relationship between ethical leadership and corporate reputation can be deduced from the CEO's relationship with different stakeholders. For example, ethical companies are able to attract employees fitting to the organisational values efficiently, who in turn support each other and work as a team thereby improving efficiency and productivity (Podsakoff, Whiting, Podsakoff and Blume, 2009). Customers and suppliers seek to form business partnership with reputable companies built on trust, and therefore develop loyal and reliable business partnership relationship with such organisations to reduce costs of acquisition and negotiation. The community acknowledges the social responsibility of such organisations thus enhancing its reputation and opportunities for more

customer patronage; while government supports ethical organisations with favourable policies to reduce cost of transactions and so on and so forth (Mihelic, Lipicnik and Tekavcic, 2010).

Corporate reputation enables a firm to attract and retain quality employees, investors, customers' loyalty, government recognition and strategic partnerships. A good reputation is also helpful for firms undergoing crisis, as they are offered a benefit of doubt by the society, and hence able to maintain their standing, whereas an unethical corporation may suffer a collapse from such crisis. The link between leadership and corporate reputation can be viewed from how the public or rating agencies form their perception of a leader's 'prominence and quality'. The media as a 'social arbiter' shines light on the CEO, thus giving the public the opportunity to evaluate both the positive and negative information disseminated, in order to form a perception. A net significant positive information therefore leads to a favourable evaluation of the CEO, and by implication the firm's reputation. Furthermore, as CEOs get industry awards and recognitions from credible institutions, they enhance the public perception of their quality and capabilities. CEOs recruited externally also have intrinsic reputational advantage in that they are seen as 'corporate saviours' (Love, Lim, and Bednar (2017). Other means of evaluation include the firm's performance, social and environmental performance, and philanthropy.

#### 2.1.16.3 Regulation and Financial Innovation

Financial innovation allows banks to serve their customers more effectively and efficiently, lower costs of operations, and gain competitive edge through leadership position in ICT, operating processes, innovative products and services among others. Regulation serves both as a mechanism to protect the customers and the financial industry, and as an enabler of financial innovation. Regulation is equally important to manage the risk of anti-money laundering and terrorist activities associated with the highly digitalised global payment system that facilitates billions of transactions per day. The lack of regulatory oversight was attributed to the financial crises of 2008-2009, when financial services firms developed risky products (such as collateralised debts, swaps, and special-purpose vehicles) and practices especially in the mortgage sector (Trichet, 2009). In response to the 2008/9 global financial crisis in the banking industry, Basel II plus compliance prescribed regulatory reforms to address the risks of

unregulated financial innovation (Oluwagbemi, Abah and Achimugu, 2011). The Dodd-Frank Act in US for instance produced a set of rules spanning eight hundred and forty nine (849) pages, leading to concerns about how restrictive and stifling the rules are in promoting creativity.

Whilst it may thus be argued that efforts at regulatory compliance add to banks' burden, it offers opportunity for savvy firms to move ahead of peers. Stewart (2010) provides a taxonomy of regulation benefits on innovation. Compliance burden triggers compliance innovation, or circumventive innovation which allows the firms to escape the regulatory constraints e.g. regulation on financial inclusion by CBN caused banks to develop new products and update their delivery channels to bring in more customers into the banking net.

Determinants of innovation include a firm's size, market share, and industry competition among others. Big firms have the ability to recover the cost of innovation, while smaller firms are able to innovate speedily by avoiding bureaucratic bottlenecks. Also, firms controlling a larger market share, possessing skilled employees, and older in age have better incentives to innovate (Lerner and Tufano, 2011). Other factors which may influence the degree of innovation include the level of education in the country, location of the firms, profitability, level of employment, and macro-economic factors. Ultimately, innovation is considered a competitive edge for sustainability of organisations, as it allows for improved customer satisfaction, cost and risk reduction. Regulation influences financial innovation as banks try to lower their costs of doing business by 'eluding the legal stipulations that affect their profitability' (Baicu, n.d.), comply with CBN's sustainable banking principle which requires financial inclusion, and respond to minimum capital requirement and competition. The regulators have a balancing role to promote financial innovation-driven growth whilst ensuring customers' protection and financial stability at the same time.

The CBN's principle of sustainable banking requires bank to develop strategies for embracing and speeding-up financial inclusion in order to drive economic growth. Hitherto, the Nigerian economy has largely been cash-based with a significant portion of currency outside the banking system. Financial inclusion is described as a 'process or situation which allows for ease of

access to, or availability of and usage of formal financial systems by members of the economy' (CBN, 2013). CBN anchored its financial system strategy on developing a cashless policy with key initiatives that include: development of varied financial products; enhancement of payment processes; development of credit system; and encouragement of a savings culture. In response, several financial innovation measures have been introduced - ATMs, point-of sale (POS), Mobile money, and internet banking. Banks have introduced information communication technology initiatives, upgraded their operating processes and systems, improved on their delivery channels among others. The regulator however has a role to promote consumer education, ensure the integrity of the innovation, and protect customers against exploitation and losses.

#### 2.1.16.4 Agency Mechanisms and Financial Performance

Agency mechanism is expected to lead to strong financial performance by addressing agency problems through minimal agency costs incurred by the owners. Agency costs enable the manager to engage in high risk investments, and high financial leverage with potentials for high returns. The risk management function provides a check on mitigating the high risks. In addition, managers' potential for expropriation of assets is eliminated or reduced to the barest minimum, which leads to improved operating performance. As banks develop strong governance practices, they build goodwill, which in turn allows investors and the general public/other stakeholders to develop greater trust and confidence in their future cash flow and growth prospects resulting in higher market valuation (Haque and Arun, 2016). Agency mechanisms therefore offer opportunities for efficient use of capital.

The banking institution is uniquely different from non-banking firms (Macey and O'hara, 2003) due to the nature of its capital structure. Capital is provided by both the shareholders and the creditors (notably depositors and debt-holders). Funds made available by the depositors are used for intermediation purposes and lent out to customers in need of funds. The efficient bank is that which can improve the information asymmetries between the depositor and lender while managing the risks. Banks therefore have to design their products and services to meet customers' needs. The intermediation requires skills to diversify credit and liquidity risks while avoiding the likelihood of bank-run by depositors who have lodged in their demandable funds

(Joseph and Loretta, 2013). This balancing act, which influences the bank performance requires making a choice of risk against an expected return – reduce risk to prevent bank distress or increase risk to achieve higher returns. The choice of risk therefore influences banks' transaction profile and dynamics (such as asset mix, asset quality, debt maturity, and risk management etc.) and in turn their cost and profitability. Black, Jang and Kim (2006) and Claessen (2006) posited that organisations with good governance practices benefit by having lower cost of funds and higher profitability, which ultimately enhances their market value. This is the agency view of using corporate governance mechanisms to prevent misappropriation and therefore maximise shareholders' value.

#### 2.1.16.5 Governance Practices Disclosure and Sustainability Performance Reporting

Gibson and O'Donovan (2007) established a relationship between governance practices disclosure and sustainability, visibly demonstrated by increasing annual report disclosures. Governance practices disclosure covers different aspects of corporate governance mechanisms and practices, which individually and collectively enhance sustainability performance reporting. Some of the studies supporting the relationship between governance practices disclosure and sustainability performing reporting include the work of Michelon and Parbonetti (2012) which found board composition correlates to quality of sustainability reporting. Rupley, Brown and Marshall (2012) opine that independent directors are generally more exposed, have deeper and wider interaction with several stakeholders, and are less obligated to management, which enhances their inclination towards a firm's reputation and sustainability reporting. Adams and Ferreira (2009) posited that board diversity especially in terms of women on the board show more passion for governance practices and sustainability disclosure, while Ong and Djajadikerta (2016) found that the presence of certain committees such as governance, audit, and sustainability allows for more dedicated resources by the firm towards improving their sustainability initiatives and performance.

## 2.2 Theoretical Literature

Corporate governance has been studied from different theoretical lenses, which include agency, stakeholder, steward, institutional, resource dependency theories among others. This section reviews the agency, stakeholder, and institutional theories, and concludes with a theoretical framework to underpin this study.

### 2.2.1 Agency Theory

The origin of agency theory can be traced to the work on separation of ownership and control of firms by Berle and Means (1932) in examining the issue of individual greed in the modern corporation, earlier alluded to by Smith (1776) who asserted that it would be asking too much of directors of corporations to exercise the same degree of care over other people's money as private company partners would over theirs. Jensen and Meckling (1976) identified the phenomenon as agency problem, and defined an agency relationship as a 'contract under which one or more persons (the principal(s)) engage another person (the agent) to perform some service on their behalf which involves delegating some decision-making authority to the agent'. This emphasises the separation of ownership from control (Fama and Jensen, 1983), following from which literature identifies three major assumptions that define the relationship between the principal and the agent: information asymmetry, bounded rationality, and potential for goal conflict (Gomez-Mejia and Wiseman, 2007), and they jointly account for moral hazard and adverse selection problems in the agency relationship.

Information asymmetry occurs wherein shareholders and managers are in possession of different sets of information (Cai, Liu, Qian and Yu, 2015). Agents, as managers of corporations are the custodians of information about the business operations because of their familiarity with their assigned roles and responsibilities. This gives them an advantage which can be exploited at the expense of the principals. Their access to information may allow them to use same for their private agenda or entrenchment (Milgrom and Roberts, 1992), and as information asymmetry increases, it becomes more difficult for principals to know if the agent is doing the right thing (Balkin, Markman, and Gomez-Mejia, 2000). The existence of information asymmetry therefore allows the agents (managers of a corporate entity) to pursue objectives that may be at variance with that of the owners (Ross, 1973; Fama, 1980). The

agency theory seeks to align the principal and the agent's interests through various direct and indirect mechanisms. This is to manage the information asymmetry and establish the limits of managers' discretionary power and the transparency of their actions. Such mechanisms include compensation schemes for managers, direct monitoring by the Board of Directors, strengthening shareholders' rights, legal protection against managers' tendency to expropriate, class action suits (Claessens, 2006) and prohibitions against insider dealing. The indirect means include corporate control, such as those provided by capital markets, threats of takeovers, and the labour market among others.

Potential for conflicts arise when, in the perception of a firm's owners, managers focus on their own interests or do not manage the firm in the best interests and a manner consistent with the wealth maximisation objectives of the owners, through an efficient, productive and dynamic allocation of resources (Maher, Maria, and Andersson, 1999). Agents cannot assume the entire cost or benefits associated with their action. In meeting the needs of the shareholders, agents also have their own objectives – such as maximising their own remuneration, preference for some investment projects, or increasing the firm's market share (Coase, 1937; Williamson 1975, 1985, Jensen and Meckling, 1976). To resolve this conflict, agency theory proposed that writing a complete contract (stating in clear terms ex ante) on utilisation of resources, distribution of benefits, expected business value within a time frame etc. may be a solution, except that complete contracts are impossible since it's not practicable to predict all possibilities. Therefore, incomplete contracts with "residual rights of control" was seen as the practicable solution. Thus, 'governance structures can be seen as a mechanism to make decisions that were not specified in the original contract' (Hart, 1995).

It is also expected that the agent and principal will exercise mutual mistrust, which the agency theory recommends to be resolved by incurring an agency cost. Agency cost is the sum of the "monitoring expenditures" by the principal, the "bonding expenditures" by the agent, and the "residual loss". As described by Jensen and Meckling (1976), monitoring costs are incurred by the principal to mitigate the agent's deviant tendencies or manipulative behaviour. Bonding costs are incurred by agents to ensure that they will not take actions to harm the principal's interests or provide compensation in the event of such actions. Residual loss is a potential cost

that occurs when both monitoring costs and bonding costs fail to control the divergent behaviour of the manager. It is an additional agency cost incurred as a result of the reduction in the principal's welfare arising from this divergence between agent's action and principal's interest (Jensen and Meckling, 1976). It may be argued that the principal has the right to sanction an errant agent, the cost of doing so may be expensive (Jensen and Meckling, 1976). Therefore, corporate governance represents a mechanism towards enhancing the long-term success of an organisation (Solomon, 2013). It demands that the management of corporations account for the welfare of stakeholders such as customers, employees, investors, suppliers, and communities, while the board of directors are expected to be responsible for the overall governance of the corporations (Shleifer and Vishny, 1997). The agency theory constructs relevant to this study include, internal governance controls, agency mechanisms, financial innovation, regulation, and financial performance. A summary of tenets of the agencytheory is depicted below:

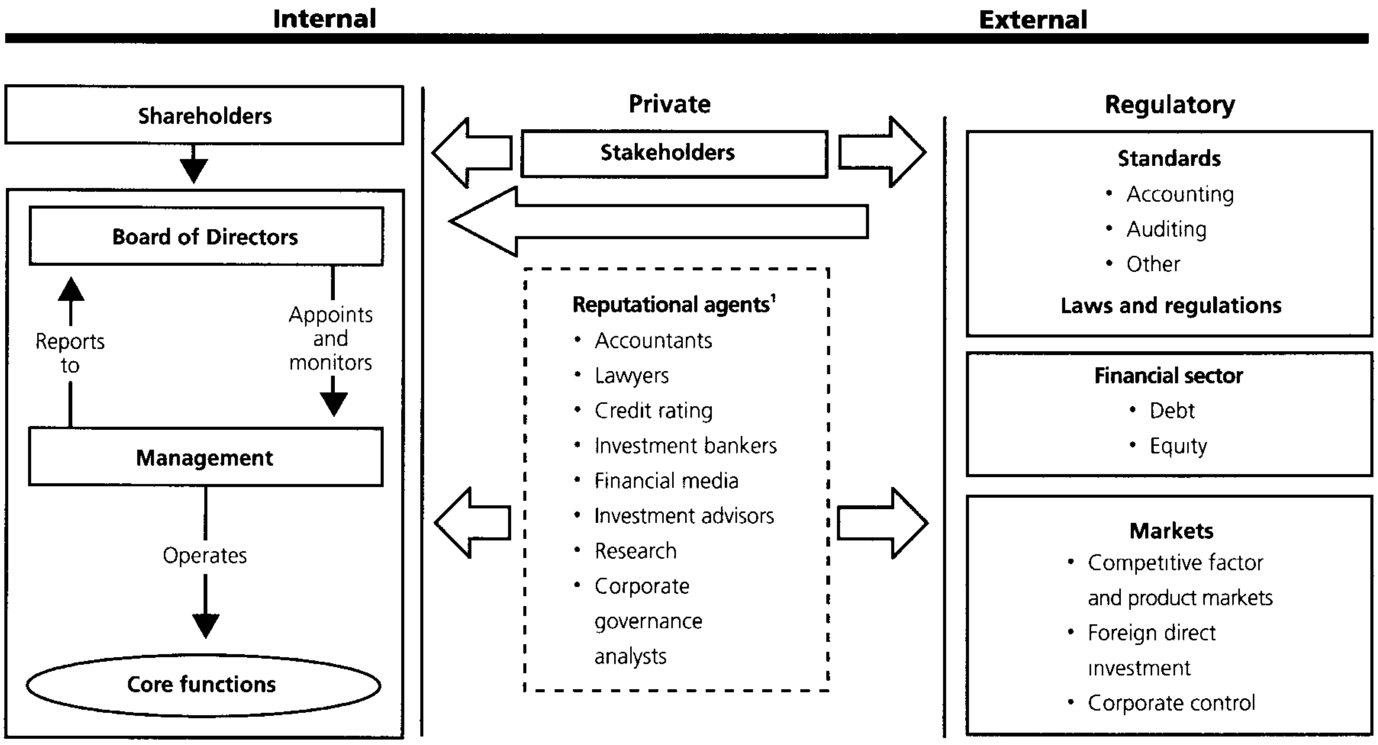


**Fig 2.1 Corporate Governance Framework – Agency Theory**

**Source. Adapted from Muir and .Saba (1995)**

### 2.2.2 The Stakeholder theory

The stakeholder theory builds on the limitations of the agency theory by seeing the organisation as having a bigger role and vision, especially in the society. It therefore seeks to enlarge the interested parties beyond the shareholders by accommodating other involved parties such as employees, suppliers, customers, environmentalists, government and the society among others. Freeman (1984) defined a stakeholder as "any group or individual who can affect or is affected by the achievement of a corporation's purpose". The stakeholder model views all legitimate stakeholders as working towards receiving benefits, and thus no stakeholder is more important than the other.

The stakeholder theory surmises that the firm has responsibilities towards the potential of all stakeholders, which promotes the duty of the firm beyond the economic objective towards the ethical objective. As a corollary, the directors have responsibilities to the stakeholders (shareholders and non-shareholders) and must seek to balance their interests, even if divergent or conflicting. The survival or prosperity of a company cannot rely principally on the contributions of the shareholders, rather it requires the input of other stakeholders, especially the primary stakeholders, which have been agreed in literature to include employees, suppliers, customers, and the community. Consequently, the consideration of other stakeholders' interests will not only increase the stakeholders' value, but also the social wealth in the final analysis. The social wealth is earned from the loyalty of stakeholders, enhanced reputation of the firm, and extra reward paid by the stakeholders. Stakeholders therefore play important role in sustainability of firms, through resources and help beyond just being irritants (Clemens and Bakstran, 2010). Consequently, stakeholders are about the business, and the business is about the stakeholders (Freeman, 2001).

Stakeholder theory preaches fairness to all stakeholders and not just the shareholders. Thus, balancing economics with ethics, moreover it's the collective participation of the stakeholders as a team that can guarantee profit and sustainability. It is the postulation of the stakeholder theory that value creation for stakeholders consequently translates to value creation for the shareholder as one of the stakeholders. To make profit, an organisation needs to have satisfied customers (for return business), satisfied employees and managers (for higher productivity),

satisfied community (to avoid disruptive activities to the business) and so forth. The desirable company therefore, is one which is able to forge teamwork between the various stakeholders. This teamwork is promoted through innovation in product quality (involving collaboration with customers), and enhanced corporate reputation (resulting from collaboration with the community). Shareholders would enjoy greater wealth and an enduring business when interests of other stakeholders, trust, and development of long-term relationships are embedded in business practices (OECD, 2014).

To situate the stakeholder theory within the context of its nature and purpose, it has been classified into three theses - descriptive, instrumental, and normative (Donaldson and Preston, 1995). The descriptive thesis views the corporation as an assemblage of cooperative and competitive interests with inherent value. The instrumental thesis identifies connection between stakeholder management and the firm's objective, while the normative serves as guide to managers in their decision-making about identifying legitimate stakeholders and their interests (Asher, Mahoney and Mahoney, 2005). Stakeholder theory is widely used in management studies – corporate social responsibility, organisational behaviour (Brenner and Cochran, 1991; Clarkson, 1991; and Wang and Dewhirst, 1992) and public management amongst others. The instrumental and normative perspectives are the more relevant aspects of the theory related to corporate governance, with Jones (1995) and Hendry (2001) using the theses to link corporate governance and ethics.

Notwithstanding the appeal and logic of the stakeholders' theory, it has often been criticized for its ambiguity, impracticability of balancing conflicting interests (Donaldson and Preston, 1995), and non-standardized variables to measure stakeholders' interests or benefits, enforcement of stakeholders' rights among others. Freeman's definition of a stakeholder has been described as too simplistic as it opens up avenue for inclusion of any and every party to be a claimant, as stakeholders have individual agenda in relation to the company and hence differing set of goals (Fitzgerald and Storbeck, 2003; John and Senbet 1998; and Maher and Andersson, 2000). Common in literature however are two (2) important categories – primary and secondary stakeholders. In the former category are shareholders, employees, suppliers and customers, who all have a direct exchange relationship with the firm (Clarkson, 1995). In the

latter category are governments, trade associations, communities and political groups among others. They have indirect relationships with the firm, but are clearly affected by its actions, mainly in terms of the social or environmental consequences (Donaldson and Preston, 1995).

Unfortunately, the theory does not provide as much a cast-in-concrete protection for stakeholders as the agency theory provides for shareholders, yet it remains one of the most important theories used in the study of corporate governance. Based on OECD principles of corporate governance, and the work of Jones (1995) and Hendry (2001), the stakeholders' theory constructs relevant to this study are ethical leadership, corporate reputation, sustainability, and governance practices disclosure. A summary of tenets of the stakeholders' theory is depicted below:

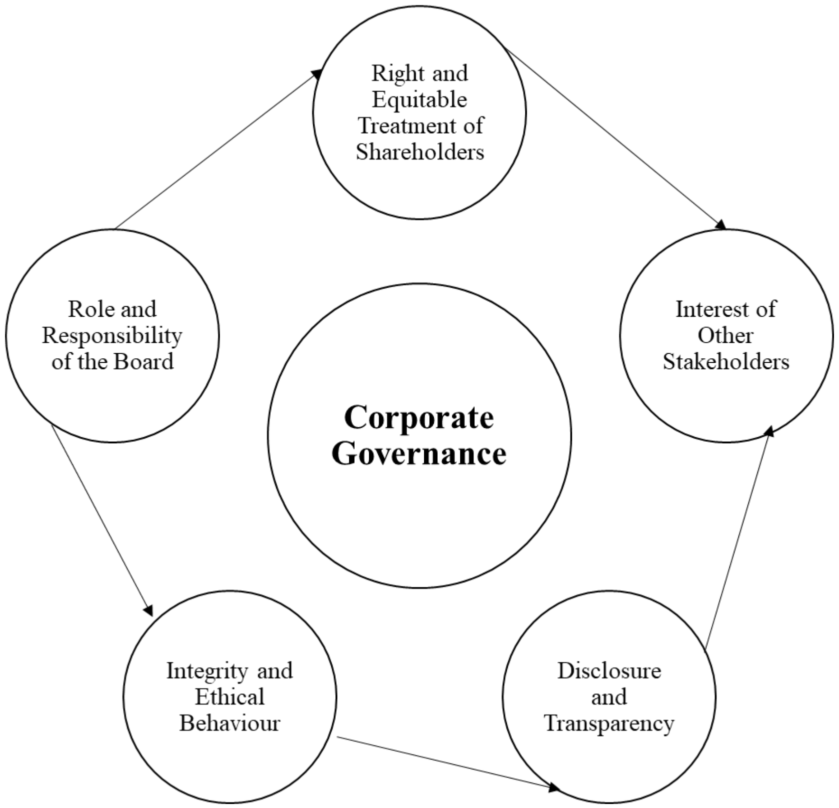


**Fig 2.2 Corporate Governance Framework – Stakeholders Theory Adopted from OECD (2004)**

### 2.2.3 Institutional Theory

Institutional theory has gone through several developments and revision since the work of Selznick, cited in Bukhari (2014), resulting in the dispensation of early and old institutionalism, new institutionalism, and neo-institutionalism. Institutional theory focuses on critical aspects of social structure. It considers how authoritative guidelines for social behaviour are established (Scott, 2005). Different components of institutional theory explain the creation, diffusion, adoption, and adaptation of social structure over time; and how they wane and become abandoned (Oliver, 1992; Gilmore and Sillince, 2014). Institutional theory views the state as key in influencing individual firms, the industry, and stakeholders' relationships. This is referred to as the legitimacy creation process, which is critical for all kinds of firms (Yamak and Süer, 2005).

The institutional theory attempts to answer the question: why are organisations so similar? DiMaggio and Powell (1983) posited that organisations achieve isomorphism by adopting similar practices and structures to fit in to their institutional environment. This can easily be seen from structures such as organisational charts, practices such as corporate governance and Corporate Social Responsibility, strategic decisions such as mergers and acquisitions, and downsizing. The theory further posits that organisations are rooted in their institutional environments, and exist in two worlds concurrently – the economic world where they must grapple with factors of production such as capital and labour, and a social world concerned more with symbolic factors such as legitimacy and status. The institutional environment is made up of institutions which consist of explicit rules and regulations, shared social cognitions, and social norms, all of which constrain and shape organisations (Suddaby, 2013).

The institutional theory is built on six (6) key assumptions - infusion of value, diffusion, rational myths, loose coupling, legitimacy, and isomorphism (Suddaby, 2013). Infusion of value considers how structures such as functional titles in organisations, families, church etc. acquire value beyond their intended function, and this sometimes bring about unintended consequences leading to purposive action. Diffusion considers how adoption of new practices, innovation, structures etc. are more often based on subjective/social reasons rather than objective/technical ones for as long as they are in accordance with societal expectations and

values. Thus, an adopter of innovative practice will subject a new practice to traditional beliefs and accept innovation only after establishing a consistency in what is communicated as the reasons for the innovation. Rational myths explain how organisations rely on perpetuated/conventional assumptions about what constitutes appropriate behaviour as defined by myths of economic rationality rather than economic rationality, thus surviving by complying with their institutional environment. Organisations adopt practices sometimes not because of conviction, but because they want to follow the crowd or conform to social pressure, therefore they need to separate their key productive functions from other functions adopted as a result of institutional pressures. Loose coupling occurs when organisations separate formal adoption of a practice from its implementation. Organisations achieve the appearance of legitimacy when they follow rational myths. They are therefore more likely to survive and be successful by having more access to resources precluded from organisations considered not legitimate (Suddaby, 2013).

***Isomorphism***

DiMaggio and Powell (1983) defined institutional isomorphism as “a constraining process that forces one unit in a population to resemble other units that face the same set of environmental conditions”. Organisations largely adopt structures, behaviours and practices found in other leading organisations in order to conform to institutional environment. This brings about similarity in identity (isomorphism) over time as they face and respond to similar institutional pressures. Isomorphism in organisations involves three processes: Coercive isomorphism – largely from rules or laws by the state; normative isomorphism – largely from pressures of a profession; and mimetic isomorphism – copying perceived successful or legitimate organisations even when the success principles are not obvious (Suddaby, 2013).

Scott (1994) built on institutional isomorphism by identifying a similar concept called institutional pillars, which are regulative, normative and cultural-cognitive, representing legal, social and cultural institutions. In summary, the institutional theory approach to corporate governance postulates adopting the isomorphism lens, and institutions could be the culture, religion, market, family, regulations, political, or legal. It is a theory also widely used in organisational behaviour, economics, political science and sociology. A summary of tenets of the institutional theory is depicted below:

| | Internal Control | External Control |
|---|---|---|
| **Institutions** | | |
| | | |
| **Formal** | Board of Directors, Management, Shareholders (ownership) | Regulations, Laws, Industry self-regulation, Reputational agents (financial analysts, accountants, Investment bankers/advisors, Rating agencies, Lawyers etc) |
| | | |
| **Informal** | Religious beliefs, cultures, ethics, firm specific norms and values, managerial ethos, codes of conduct | Industry self-regulation<br>Reputation<br>Societal norms and values<br>Traditions and values |

**Fig 2.3 Corporate Governance Framework – Institutional Theory**

**Adapted from DiMaggio and Powell (1983); Meyer and Rowan (1977); La Porta et al., (2008)**

### 2.2.4 Theoretical Framework

The agency and stakeholders' theories are adopted as the theoretical underpinnings of this study. Agency theory was developed and revised by many scholars including Berle and Means (1932), Jensen and Meckling (1976), Fama and Jensen, 1983), while the stakeholders' theory was developed by Freeman (1984); Donaldson and Preston (1995), with contributions by OECD (2004), BASEL (2015) among others. The two theories are widely used in the field of management to study organisational behaviour, corporate social performance and corporate governance among others. The agency theory indicates that governance structures need to be put in place to prevent managerial opportunism, and align managers' behaviour to the owners' interests. In addition to managing the conflict of interest, information asymmetry involving the managers versus owners and stakeholders increases the perceived risk of a firm by the investors. The market responds to this by under-valuing a firm's shares or requesting for increased returns. These challenges can however be managed by adequate public disclosure, which is a pillar of sustainability performance reporting (Warren and Thomsen, 2012). The stakeholder theory postulates that the firm has responsibilities and accountabilities towards a broad range of stakeholders, which promotes the duty of the firm beyond the economic objective towards the ethical objective, and that it's the collective participation of the stakeholders as a team that can guarantee profit and sustainability. It further holds that by

ignoring stakeholder interests, a firm's reputation is at risk, and similarly its financial performance.

Applied to this study, the agency theory holds that it is expected that agency mechanisms (Shleifer and Vishny, 1997; Berle and Means, 1932) will influence financial performance. This is because managers will not be able to expropriate assets or resources; and will also make investment and risk-taking decisions in a manner consistent with the wealth maximisation objectives of the owners, through an efficient, productive and dynamic allocation of resources (Jensen and Meckling, 1976).

Regulation will influence financial innovation because, while it may place a compliance burden on banks, non-compliance may lead to grave consequences such as a heavy penalty, picketing or shutdown, unless they innovate. On the other hand, regulation that reduces information asymmetry (between the bank and its customers) about a product or service will aid innovation. Financial innovation has positive effect on information asymmetry and reduces the cost of transaction for banks. Financial innovation allows banks to assess effectively the risk of a potential customer, whilst monitoring existing customers. This reduces the problem of adverse selection and moral hazard advanced in agency theory, but also raises the need for mitigating the associated innovation risks (Pinto and Sobreira, 2010). Innovation-growth view expects financial innovations to help reduce agency costs, facilitate risk sharing and ultimately improve allocative efficiency and economic growth (Beck, Chen, Lin and Song, 2012). Regulation has a role to prevent banks from exploiting customers' and investors' misunderstandings of financial markets (Henderson and Pearson, 2011). Social regulation promotes social responsibility, for instance 'financial inclusion' (which is largely achieved through financial innovation) as enshrined in CBN's principles of sustainable banking. It's also the driving force behind diversity, ethics, product responsibility, and human rights among others. Social regulation therefore seeks to correct a market externality by imposing requirements on banks to protect the welfare of society or the environment (Stewart, 2010).

The stakeholders' theory holds as follows: that it is expected that ethical leadership (which prevents abuse of power or inappropriate actions by managers according to Mercier, cited in

Dessain, Meier and Salas, 2008) would influence the corporate reputation of the firm because it promotes a company's worth and confers competitive advantage and benefits such as attraction of employees, customers, and investors, while allowing firms to charge premium prices (Deephouse 2000; Fombrun 1996). Corporate governance practices disclosure will influence sustainability performance reporting because key stakeholders want businesses to be responsible, accountable and transparent, which can be achieved through sustainability reporting. Sustainability performance reporting thus helps to mitigate risk, protect corporate brand, and secure a competitive position (WBCSD, 2003); and finally, that internal governance controls will influence corporate social performance because aligning decisions solely to the interests of shareholders is counterproductive since it does not guarantee the sustainable development of the organisation, which can only result from the convergence of all stakeholders' interests (Donaldson and Preston, 1995).

With the above statements, the formula for sustainability is explained thus:
If agency mechanisms are put in place, shareholders' values will be maximised, through returns from efficient use of resources. The ethical consideration of stakeholders and the influence of regulation support social responsibility, growth of companies' capacity to innovate, and preservation of their corporate reputations and valuations especially with respect to their image. By committing itself to sustainable development, "a bank is not doing so just for legal compliance or response to pressure, but also to develop a capacity to generate value for all stakeholders in an equitable and responsible way" (Dessain, Meier and Salas, 2008).

## 2.3 Empirical Framework

This section discusses previous empirical findings identified in literature within the context of corporate governance dimensions and sustainability. The review considers findings in literature from the developed countries, developing countries, and Nigeria. The presentation format is in tabular form, arranged on the basis of the hypotheses formulated in the study. Each study reviewed is tabulated with information on the methodology, sample description, source of data, key findings/conclusions, and the researcher's remarks/observed limitations (if any). A summary of gaps identified generally, and specifically to each study variable is presented at the end of table 2.1 **Table 2.1: Review of Empirical Findings** below.

| | INTERNAL GOVERNANCE CONTROLS AND CORPORATE SOCIAL PERFORMANCE (CSP) | | | |
|---|---|---|---|---|
| | | | | |
| S.No | Author(s) & Study Scope | Methodology/Sample Description/Da | Key Findings and Conclusions | Remarks and Limitations |
| **1** | Arani (2016) - Iranian firms | Descriptive with correlation. Ex-post facto. Panel data Generalised Regression method. {128 companies. Electronic archives and resources such as Rahavard Noving software and Tehran Stock Exchange Codal [illegible] | significant relationship between institutional ownership and CSP; positive significant influence of board size, and proportion of non-executive directors on CSP | Secondary data. Focus on Social Responsibility Disclosure. The positive -Extracted raw data from documents and websites into MsExcel before inputting into Eviews 7 for analysis |
| **2** | Ali (2013) - Pakistan | Literature review {Review of 35 research articles on CG and Social Responsibility} | Key CG mechanisms - board gender, independence, directors' tenure, and board size. Findings: More female board members are more conscious about CSR, large board size may not be supportive in CSR strategy, independent directors influence CSR strategy positively, CSR improves | Literature review, not empirical. Study refers further work on effect of board composition and board structure on CSR. |
| **3** | Desender and Epure (2013) - 27 countries comparative study | Ex-post facto. Panel data regression {1116 firms - Data from Thomson Reuters ASSET4, Datastream, worldscope, and national data variables from multiple sources - world bank, Hofstede} | A positive relationship between foreign ownership concentration, board independence and CSP, while formal and informal institutions significantly moderate the relationship between ownership concentration and CSP | International panel data analysis/cross-country comparative study, which is quite different from the focus of this study. Also, the use of secondary data from Datastream and ASSET4 versus extracting raw data from the annual reports of banks. CSP scores provided by Thomson Reuters ASSET4 are an imperfect measure, while the study sample mostly includes firms from developed markets. |
| **4** | Arora and Petrova (2009) | {382 firms - List of firms from S&P 500, Soocial performance from KLD Domini 400 Universe, Financial information from CDA/ Spectrum Thomson Financial's 13F database, corporate governance data from the RiskMetrics database, and financial performance from Compustat}. | The interaction effect between commitment-based employee relations and environment show a positive relationship with financial performance; and corporate governance systems moderate the relationship between various social performance domains and financial performance. | The study established the importance of studying the interdependence among governance mechanisms. Study was based on secondary data. |
| **5** | Fauzi, Mahoney, and Rahman (2007) - Indonesian firms | Secondary data collected from Company Annual Reports for firms registered on the Jakarta stock exchange. {324 firms} | No relationship between institutional ownership and CSP, but a good financial performance leads to an increase in CSP | Study was limited to only one governance variable (institutional ownership), and one year annual report. The study however recommends combining content analysis with qualitative approach. |

| | | | | |
|---|---|---|---|---|
| **6** | Walls, Berrone, and Phan (2012) - US firms | Exploratory, fact-based research design. Random Effects Least Square panel regression. | board, management, and ownership mechanisms of corporate governance are relevant to environmental performance | Exploratory research. Study sample was based on dirty/polluting industries where environmental impact is directly significant. Study recommends use of surveys, case studies and other means of investigation. Study was also limited to environmental performance, hence not generalisable to CSP. Positives: Separation of environmental strengths and concerns in analysis to see the true environmental performance. There was detailed study on interaction effects between corporate governance and environmental strengths and concerns |
| **7** | Villiers, Naiker and Staden (2011) -US firms | KLD Index ratings {1216 firms - social perfomance data from KLD database, financial data from compustat files, board governance data from the Corporate Library's Board Analyst database} | evidence of strong environmental performance in firms characterized by the following: larger boards, more legal experts on the board, interlocking directors, higher concentration of independent directors, and lower concentration of directors appointed after the CEO took office. | Positives: Multiple-theoretical perspective, cross-section of industries, large sample size and sample period, complete set of board characteristics rather than narrow set. The study tested relationships and not causation, and also did not establish determining factors (compliance versus strategic) for environmental performance |
| **8** | Berrone and Gomez-Mejia (2009) - US firms | fixed-effects estimation models {469 companies - ExecuComp database, Compustat database, the Investor Responsibility Research Center (IRRC) database, and proxy statements reported to the Securities and Exchange Commission (SEC)}. | environmental performance is an important non-financial determinant of CEOs'-pay in dirty industries (i.e. polluting industries). | Data source not common in developing countries. Quantitative research without the benefits of follow-up interview with officers in sampled firms. Study considered a reverse causation between environmental performance and executive compensation suggesting the latter is the outcome contrary to most studies. Performance measures used are not readily available to the CEOs studied. |
| **9** | Wu and Shen (2013) - 22 countries comparative study | Secondary data. Heckman two-step regression {162 banks - Ethical Investment Research Service (EIRIS) databank and Bankscope database} | Study considered three motives behiind CSR (Strategic, altruism, and greenwashing). CSR positively associates with Financial Performance in terms of return on assets, return on equity, net interest income, and non-interest income. In contrast, CSR negatively associates with non-performing loans. Strategic choice is the primary motive of | CSP was treated as explanatory and financial performance as outcome, which is different from this current study. Study could not establish the individual effect of social/environmental/governance practices on financial performance because an aggregated index was utilised. Positive: Focus on the banking industry. |
| **10** | Xi'an, Xi'an, Fan, and Luo (2017) - China | Secondary data. Panel regression {606 listed firms. CSR reporting quality evaluation scores obtained from CSR rating agency (Rankins Inc)} | Government mandatory regulation leads to an overall improvement in CSR reporting quality. The positive effect is greater when firms are larger and have better financial performance, but less when firms are controlled by government | Dummy variables was used to measure government regulation (the independent variable). |
| **11** | Wong and Wong (2015) - 22 countries comparative study | Secondary data collected from recent websites, annual reports, CSR reports and sustainability reports of banks. {3 banks. Secondary (Annual reports)} | Selected banks channelled their efforts on environment, people, and community. Stakeholder pressure makes social responsibility inevitable/unavoidable for banks. | Case study of 3 banks + reliance on secondary data for analysis. Study only focused on 1-year review (2013). Study recommends use of primary data (questionnaire, interview) for future research. |

| | ETHICAL LEADERSHIP AND CORPORATE REPUTATION | | | |
|---|---|---|---|---|
| | | | | |
| S.No | Author (s) | Methodology | Key Findings and Conclusions | Remarks and Limitations |
| 1 | Love, Lim and Bednar (2017) -US | Secondary data. Panel data regression. {372 firms. Data from Fortune magazine's annual "Most Admired Company", ExecuComp database, Financial World's annual "CEO of the Year" competition} | While CEOs can substantially influence their firm's reputation, only a few of them make a dramatic difference. Industry awards and recognitions do not lead to superior firm performance as they place burden on the CEOs leading to higher performance expectation. Firms are seen in part as a reflection of their leaders. | |
| 2 | Eisenbeiss, Knippenberg, and Fahrbach (2015) - German firms | Web-based survey, Likert scale for different variables ranging from four points to ten points likert scale. {32 firms, 145 questionnaire survey participants, performance data for the organizations via the Electronic Federal Index of the German Justice Ministry} | CEO ethical leadership correlated positively with organizational ethical culture. The organizational ethical culture was positively intercorrelated with firm performance. Significant interaction effect between organizational ethical culture and organizational ethics program on firm performance. A significant conditional indirect (i.e., mediated by ethical culture) effect of CEO ethical leadership on firm performance when there was a strong ethics program. | Financial performance drawn from employees and only validated with objective performance data (where available). Study could not establish causal relationships. Cross-industry study loses the benefit of homogeneity, as industry characteristics may mask the results. |
| 3 | Mihelic, Lipicnik, and Tekavcic (2010) -Slovenia | Literature review / Conceptual Review. {Definition of ethical leadership, and description of ethical leader's personality} | | Not empirical |
| 4 | Watson, T. (2007) -US | {4 case studies} | poor management, unethical practices, a lack of engagement with customers and other stakeholders, indifferent or aggressive performances by CEOs and lack of preparedness for crisis communication severely or terminally affected the organisations | No quantitative hypothesis testing. Case studies were based on published sources and financial data, thereby limiting their generalisability |
| 5 | Boadu (2013) - Ghana | grounded theory approaches of three primary research methods: survey questionnaires, interviews and focus group discussion. 28 Semi-structured interviews - Top management staff interview} | National cultural practices influence institutional environment, which in turn influences corporate governance. The culture of giving and receiving gifts has influence on provision of social responsibility, and unethical implications for corporate governance' | |
| 6 | Ogechukwu (2013) - Nigeria | Theoretical | | Author provides a sundry list of unethical practices in the Nigerian banking sector. Not empirical. |
| 7 | Yidawi (2005) - Nigeria | Survey, Chi-square, frequency distribution and percentages. {160 bank employees} | frauds and forgeries are on the increase; most of the frauds are due to insiders or collaborators and this has materially affected the reputation of banks; frauds and mismanagement are responsible for systemic distress | Small sample size, convenience sampling technique, non-causal analysis |

| | | | | |
|---|---|---|---|---|
| **8** | Neves and Story (2013) - Portugal | Survey {18 firms -224 subordinate-supervisor dyads} | Ethical leadership significantly related to affective commitment to the organisation; strength of the relationship between the supervisor's ethical conduct and employees' commitment to the organization varied according to the supervisor's reputation for performance; if leaders are not perceived as ethical, employees show the lowest levels of affective commitment to the organization | No causality inference; ethical leadership as explanatory variable to organisational deviance; focus on supervisor's reputation rather than corporate reputation. |
| **9** | Enofe, Ekpulu, Onobun, and Onyeokweni (2015) - Nigeria | Questionnaire Survey {100 employees -Convenience sampling} | A weak negative insignificant relationship between unauthorized tampering with customers account and financial performance; a weak positive significant relationship between insiders' related credit and financial performance in the Nigerian banking sector.Unethical | Measures/Proxies of ethical behaviour (opinion on insider related credit, and unauthorised tampering – in relation to financial performance)?.Convenience sampling technique, small sample size, financial performance measured by respondents' opinions. |
| **10** | Gberevbie (2011) - Nigeria | Secondary data | Unethical practices have consequences - prosecution, embarrassment, and negative reputation. Ethical leadership more likely to lead to organisational development, and the contrary hurts stakeholders -employees, shareholders, government etc | Secondary data. Descriptive, not inferential. Not empirical. |

**Source: Author's Analysis (2017)**

| | REGULATION AND FINANCIAL INNOVATION | | | |
|---|---|---|---|---|
| | | | | |
| S.No | **Author (s)** | **Methodology** | **Key Findings and Conclusions** | **Remarks and Limitations** |
| **1** | Stewart (2010) - USA | Lterature Review | Constraints to innovation include higher uncertainty and larger differences in the expected profitability of innovation investments. Enablers on the other hand include flexible regulations, incentives-based regulation and performance standards | Not empirical |
| **2** | Perihan and Kienpin (2017) -UAE | Panel data (Generalised Least Square) random effect. Construction of governance index and innovation index. {25 banks. Financial data from | gender and education do not significantly affect innovativeness of banks, having more experienced and independent board members enhances the innovation | Study only linked one internal governance control variable to innovation without considering the interdependence of other variables. Focus was also on the influence of oil price drop in UAE on innovation |
| **3** | O'connor and Rafferty (2012) - USA | Secondary data -Content Analysis, Panel regression {1719 firms. Data from the Investor Responsibility Resource Center (IRRC) to measure corporate governance, and other firm-level data from the Compustat database} | corporate governance has only a modest effect, at most, on the level of research and development expenditures | Proxy for innovation was R&D expenditures. Sampled firms excluded financial institutions. |
| **4** | Racic, Cvijanovic and Aralica (2007) - Croatia | Exploratory research | degree of innovative activities in an economy is dependent on internal (firm) and external (institutional) characteristics. There is however a complementarity between the system of corporate governance, financial | Not empirical |
| **5** | Arnaboldi and Rossignoli (2015) -Europe and USA | Secondary data -Content Analysis, Panel regression {81 firms} | Innovating banks are charcterised by: larger market share, cost-efficient but less profitable, lower firm age, and higher risk | Explanatory variables were bank specific characteristics, efficiency, profitability, risk, loan quality and investment in loans. Study did not consider regulation. |
| **6** | Cherotich, Sang, Shisia, and Mutung'u, (2015) -Kenya | Descriptive study. Secondary data {Census - 44 banks. Data for financial innovation obtained from Central Bank of Kenya (CBK)'s annual statistics, while that of financial performance was obtained from CBK's annual bank supervision reports and banks' annual financial statements} | financial innovativeness of commercial banks had a positive and significant effect on financial performance | Financial innovation was treated as the explanatory variable, and financial performance as the outcome. |
| **7** | Frame & White (2002) | Literature review {24 studies on innovation using thematic Classification} | regulation spurs financial innovation, adoption and diffusion by banks is related to firm size, whilst welfare effects of financial innovation appear to be generally positive | Not empirical |
| **8** | Pinto & Sobreira (2010) - Brazil | Conceptual | regulation has a role not to prevent innovation but to assure the market and the economy of the capabilities and | Not empirical |

| | | | | |
|---|---|---|---|---|
| **9** | Leaven and Levine (2009) - 48 countries - comparative study | World Bank Survey, Index construction, Regression {279 banks, Data from worldbank dataset} | the existence of a deposit insurance system increases banking risk; whilst stricter capital requirements and activity restrictions lead to higher bank risk | Regulation as an explanatory variable for banks' risk-taking, not financial innovation |
| **10** | Barth, Caprio and Levine (2004) -107 countries - | World Bank Survey, Index construction, Regression {Data from | no statistically significant relationship between regulation and performance | Regulation as an explanatory variable for banks' performance, not financial innovation |
| **11** | Pasiouras (2008) - 95 countries - comparative study | Panel data {715 banks, Data from worldbank dataset} | regulations promote technical efficiency | Regulation as an explanatory variable for banks' technical efficiency, not financial innovation |
| **12** | Pasiouras, Tama and Zopounidis (2009) - 75 countries - comparative | Panel data{615 banks, Data from worldbank dataset} | supervisory power and market discipline increase profit and cost efficiency, while capital requirements and activity restrictions provided mixed results. | Regulation as an explanatory variable for banks' efficiency, not financial innovation |
| **13** | Chortareas, Girardone and Ventouri (2010) - 11 selected EU countries | Data Envelopment Analysis{190 banks consisting of 1,382 observations. Data sourced from BankScope and Worldbank database } | regulation and supervision minimize operational efficiencies of the banks under study | Regulation as an explanatory variable for banks' efficiency, and performance, not financial innovation |
| **14** | Kremmling (2011) - 56 countries - comparative study | Panel data{515 banks, Data from worldbank dataset} | capital requirements and activity restrictions have a negative influence on loan loss provisions during the financial crisis. Deposit insurance systems improved net interest income during the crisis, even though it also increased loan loss provision | Regulation as an explanatory variable for banks' performance in a post-financial crisis era, not financial innovation |
| **15** | Alam (2012) - 56 countries - comparative study | Data Envelopment Analysis{235 banks, Data from worldbank dataset} | | Regulation as an explanatory variable for banks' efficiency and risk, not financial innovation |
| **16** | Ugwuanyi (2015) - Nigerian banks | Ex-post facto, Panel data{13 banks, Data from worldbank dataset} | positive relationship between increase in capital requirement and other variables such as bank risk, profitability, size, spread, and capital adequacy ratio. Regulation pressure however showed a negative correlation with capital adequacy and risk-taking appetite, and a non-significant effect on capital adequacy and risk-taking appetite | Regulation as an explanatory variable for banks' risk-taking, not financial innovation |

**Source: Author's Analysis (2017)**

| | AGENCY MECHANISMS AND FINANCIAL | | | |
|---|---|---|---|---|
| | | | | |
| S.No | **Author (s)** | **Methodology** | **Key Findings and Conclusions** | **Remarks and Limitations** |
| **1** | Juras and Hinson (2008) - Germany | Panel data -OLS regression{360 banks - 1440 bank year observations. Sheshunoff database used for annual financial data, board information from proxy statements} | traditional mechanisms such as board size, ownership or percentage of outside directors appear to have less impact on banks as a regulated industry. Board effectiveness and impact may be tied to the intangibles of board interaction, trust, experience, interpersonal relationships with senior management and ability to provide an effective counterpoint to a strong CEO | The use of a single industry allows study to focus upon the measures rather than potential variations across industries. Performance measures used - Dependent Variabales (ROA, ROE, NIM, Efficiency ratio), Control Variables (Loan charge off:Total Loans ratio, Capital Ratio, and Total Assets) |
| **2** | Irshad, Hashmi, Kausar, and Nazir (2015) - Pakistani firms | Panel data{100 randomly selected non-financial firms. Data from annual reports}. | Board effectiveness (measured by frequency of meetings), has no significant influence on performance. Ownership concentration has a negative and significant relationship with Return on Asset (ROA). | Study on non-financial firms. IV (Ownership, Board Size, Independence, Meeting frequency,CEO Duality) Control Variables (Firm size -total assets, leverage, age) DV (ROA & Marginal Q) |
| **3** | Ntim and Osei (2011) - South African firms | | positive relationship between frequency of meetings and firm performance | |
| **4** | Fernandes and Fitch (2009) | | No significant effects on the relationship between board 'busyness' and performance | |
| **5** | Aebi, Sabato, and Schmid (2012) -North America | Descriptive statistics, panel data regression{573 banks. COMPUSTAT Bank database; hand-collect corporate governance variables from the banks' annual report and proxy statement forms in the SEC's EDGAR database, and company websites. Other data sources include IRRC Governance Legacy database, IRRC Directors Legacy database, Standard & Poor"s ExecuComp data-base, and Thomson Financial"s CDA/Spectrum database}. | No significant effects on the relationship between board 'busyness' and performance. Standard governance measures in non-financial firms may fall short in describing the relevant governance structure of banks. | Bank performance measures used: Dependent Variables (Buy-and-hold returns; ROE, and ROA), Control Variables (Market-to-book ratio, Total Assets, Tier1 capital ratio, Depos-its/assets, Loans/assets, Income diversity) |
| **6** | Kajola (2008) - Nigerian firms | Ex-post facto. Panel data -OLS regression{20 non-financial firms. Data from annual reports. Non-probability, and stratified random sampling} | Positive significant relationship between the CEO duality and Profit Margin; and between board size and ROE | Non-financial firms. 4 explanatory variables all from the Board mechanism. |
| **7** | Ujunwa (2012)- Nigerian firms | Generalised Least Square (GLS) Fixed-Effects and Random-Effects models{122 quoted firms. Data from NSE Factbook, annual reports and financial statements}. | board gender has negative significant effect on financial performance, while ethnic diversity was negatively related to firm performance but not significant | Combined sample of 35 industries. Use of dummy variable to measure board ethnicity can be improved upon. |

| | | | | |
|---|---|---|---|---|
| **8** | Sigler (2011) - US firms | Descriptive and regression analysis.{280 listed firms. Forbes magazine and Standard & Poor's Compustat data} | found a positive and significant relationship between the CEO pay and ROE | US studies may not be generalisable to developing countries |
| **9** | Lin, Kuo, and Wang (2013) - US firms | Panel data. Regression Analysis{903 listed firms. Data from ExecuComp database}. | found tenure, size of the firm, and leverage ratio to be significantly positively associated with CEO's compensation. CEOs with older age are associated with higher cash compensation. There is a substitution effect between CEO compensation and the level of CEO ownership and that larger firms give higher pay to their CEOs | Sample period limited to four years. US studies may not be generalised to developing countries. |
| **10** | Peni (2014) - US firms | Panel data. Fixed-effect regression{305 firms -1525 firm-year observations. CEO data handpicked from AuditAnalytics database, annual reports, press releases. Financial data from Thomson Reuters Worldscope} | firms with female CEOs/Chairs may outperform firms led by males. There was also a positive relationship between CEO's age and ROA; whilst 'executive busyness' seems to increase Tobin's Q and ROA as measures of firm performance | Study excluded financal institutions, and used US firms limiting the generalisability of the results. Endogeneity tests was not conducted in the study |
| **11** | Dezso and Ross (2008) | | found female participation below the CEO level improves Tobin's Q, but no relationship between female CEOs and firm performance | |
| **12** | Akpan and Amran (2014) - Nigeria | Secondary data. Multiple regression{90 firms} | found board gender not having effect on performance, rationaled by insignificant number of female members on board, concluding perhaps it was a mere window dressing exercise to have females on the board | Sample size comprised of firms in 11 industries. Corporate governance variables concentrated only on the board mechanism. The mesurement of Board age, and board independence as independent variables can be improved upon. |
| **13** | Sanda, Garba and Mikailu (2008) - Nigerian firms | Descriptive and regression analysis (OLS and Fixed-effect).{89 firms for regression, 205 firms for descriptive analysis. Data obtained from Nigeria Stock Exchange (NSE) Factbook, and annual reports}. | no relationship between CEO tenure and firm performance | Cross-industry sample |
| **14** | Manawaduge and Zoysa (2013) - Sri-Lankan firms | regressions using pooled data{157 listed firms. Data obtained from 3 main sources: OSIRIS database (OSIRIS), CSE's Data Library - for share price information , and the firms' annual reports} | found a significant positive relationship between ownership concentration and performance (ROA and ROE) | Non-financial firms. Tests of results (R-squared, adjusted R-squared) were low. The use of pooled data regression analysis suffers the drawback of not taking the heterogeneity of the sampled firms into context. Fixed-effect or Random effect (based on Hausman Test result) more appropriate. |

| | | | | |
|---|---|---|---|---|
| **15** | Zeitun and Gary (2007) - Jordanian firms | Panel data regression{59 publicly listed companies}. | found a significant positive relationship between ownership concentration and firm performance (ROA & ROE) | Excluded banks. |
| **16** | Wu and Cui (2002) - China | | found a positive relationship between ownership concentration and accounting measures (ROA and ROE), but a negative relationship with respect to the market value measures (price-earnings ratio, and market-to-book-value ratio) | |
| **17** | Shah, Butt and Saeed (2011) - Pakistani firms | Descriptive statistics, non-parametric test, cluster analysis - square Euclidean Distance, chi square{67 firms}. | reported negative and significant association between ownership concentration and firm performance | Study excluded financial firms. Data non-normal |
| **18** | Tsegba and Herbert (2014) - Nigerian firms | Panel data regression{70 firms} | Negative and significant association between ownership concentration and firm performance; a negative and significant relationship between firm performance and insider ownership. | Study excluded financial firms. Statistics tests (R-squared) were very low |
| **19** | Abor and Biekpe (2007) - Ghanaian firms | Regression analysis | inside ownership has significant positive impact on profitability | |
| **20** | Mnasri (2015) - Tunisian banks | Panel data regression{10 banks. Annual reports, stock information (Board of the financial market)} | found a negative and significant impact of block-holder ownership on bank performance | Focus on internal governance mechanisms (ownership and board structure) only. Performance measures used: Dependent Variables (ROA, Tobins Q), Control Variables (Equity-to-Asset ratio (E/A), and Total Assets). Test for robustness (Interest Margin, Intermediation margin) |
| **21** | Bektas and Kaymak (2009) - Turkish banks | | suggest that ownership concentration and type is irrelevant to bank performance | |
| **22** | Al-Amarneh (2014) - Jordanian banks | Panel data regression{13 banks} | ownership concentration has positive and significant value on performance, but institutional ownership and foreign ownership do not affect the bank performance | Study used 3 explanatory variables from the Board Mechanism only. Bank performance measures used: Dependent Variables (ROA, Operating Efficiency Ratio), Control Variables (Total Assets, and Non-performing Loan ratio) |
| **23** | Akpan and Riman (2012) - Nigerian firms | Descriptive, Multiple regression analysis{11 banks. Data obtained from the annual reports} | 'large' banks exhibited poor loan management ability, whilst 'smaller' banks were afraid of giving large loans that might not be easily redeemable due to the cost of loan recoveries. Other findings include: a negative relationship between the board size and Non-Performing loans (NPL), and a positive relationship between the number of shareholders and Non-performing loan. | Data covered 2005-2008. The banking sector has undergone further consolidation and reforms. Semi-log linear OLS regression method was adopted, which does not take into consideration the individual heterogeneity of the banks. ROA, ROE & NPL were proxies for performance, while Board size and number of shareholders were proxies for corporate governance |
| **24** | Nyor and Mejabi (2013) - Nigerian banks | Ex-post facto. Multiple regression. {14 banks. NSE Factbook, Annual reports} | Corporate governance (proxied by board size, composition, audit committee, and CEO duality) showed no statistical significance on non-performing loans. | Explanatory variables focus on the board mechanism alone. Test statistics (R-squared) low, meaning a poor model fit. Bank performance measure -Non-performing Loan |
| **25** | Islam, 2014 - Bangladesh bank | Ex-post facto. Descriptive financial ratio analysis, paired t- test{1 bank} | | Use of 1 bank, t-test, limitation with the model used, and result not generalisable. Bank performance measures used: ROA, ROE, Cost-to-income, Net loans-to-total asset. Loans-to-deposit ratio, non- |

| | | | | |
|---|---|---|---|---|
| **25** | Islam, 2014 - Bangladesh bank | Ex-post facto. Descriptive financial ratio analysis, paired t- test{1 bank} | | Use of 1 bank, t-test, limitation with the model used, and result not generalisable. Bank performance measures used: ROA, ROE, Cost-to-income, Net loans-to-total asset, Loans-to-deposit ratio, non-performing loan ratio) |

**Source: Author's Analysis (2017)**

| | **<u>CORPORATE GOVERNANCE PRACTICES DISCLOSURE AND SUSTAINABILITY REPORTING</u>** | | | |
|---|---|---|---|---|
| **S.No** | **Author (s)** | **Methodology** | **Key Findings and Conclusions** | **Remarks and Limitations** |
| **1** | Isikuk and Chizea (2017) - Nigerian & South African firms | Unweighted composite index{10 banks (5 from each country), content analysis of annual reports} | Nigerian banks, on average, tend to disclose more corporate governance information and have more voluminous annual reports than South African banks. They are also mandatory-induced. The emergence of information communication technology and availability of Internet technology are responsible for enhancing corporate governance disclosure. Disclosure relating to social, environmental, and ethical reporting or corporate social responsibility is a routine year-end collation exercise. | Study adopted 1-year (2013) review of governance practices disclosure. Study did not carry out a relational or causal investigation – Purely descriptive. |
| **2** | Bhasin (2010) - India | Exploratory case study -combining literature review and Interviews{one (1) company} | Company complied with all the mandatory disclosure requirements of Clause 49, but only a partial compliance with some of the important non-mandatory requirements stipulated under Clause 49 | Non-availability of inside information, and limited scope for discussion with the key officials of the company. Findings based on the study of just one Indian company (RIL),for year 2008-2009, which may limit the generalizability of the results. Study focused on one avenue of company disclosure, the corporate annual reports. Study did not carry out a relational or causal investigation – Purely descriptive, using a case study of one (1) firm, which is non-banking. |
| **3** | Janggu, Darus, Zain and Sawani (2014) - Malaysia | Secondary data, content analysis, index construction{Stratified random sampling technique- 100 listed firms. Data collected using content analysis of the annual reports. PLS - Structural Equation Modelling Analysis}. | Board size, professional board members, board designation as 'Datuk' were the predictors of sustainability reporting ranked by strength in descending order. | Study limited to the Board Mechanism as the explanatory variable ignoring the complementarity or substitutability of other mechanisms (internal and external). |

| | | | | |
|---|---|---|---|---|
| **4** | Ong and Djajadikerta (2016) - Australia | Composite Index developed from content analysis{Sustainability disclosures in annual report and stand-alone sustainability reports} | significant positive correlations between corporate governance disclosure (independence, multiple directorships, female members, sustainability committee) and sustainability reporting | Study used 1-year data of Australian resources industry, and also examined a limited number of attributes of board diversity to proxy corporate governance mechanism, thereby making the findings less generalisable to conditions that differ from the study. In addition, data was not normally distributed hence the use of non-parametric tests. |
| **5** | Aggarwal (2013) | Literature review/Meta-analysis/qualitative and descriptive research approach in literature review{30 studies} | 12 showed positive relationship, 2-negative relationship, 7 - no significant relationship, and 9 - mixed results | No hypothesis testing. Study recommends sustainability research in developing countries |
| **6** | Rebai, Azaiez and Saidane (2012) - Tunisia | Mathematical Model construction{5 French banks} | All 5 banks evaluated failed to achieve 100% 'sustainable bank' status for lacking in one or more performance areas on stakeholders' utility. | Sustainable Banking Performance Evaluation Framework was based on Marginal Utility Functions, while the rating of stakeholders' preferences was based on authors' opinions rather than sourced from stakeholders. |
| **7** | Kumar (2013) - Intercontinental study | qualitative and explorative approach, employing a mix of content analysis and investigative techniques; and grounded theory{80 multinational firms} | Sustainability has emerged as an important area of soft law self-regulation for corporations significant inconsistencies and inadequacies among company reports undermine the comparability and usefulness of this information ; data consistency was the biggest challenge in applying the value and measurement approach; inconsistencies and gaps in the data reporting method used by the sampled companies making comparison difficult | Not empirical with quantitative hypothesis testing |
| **8** | Deloitte (2017) - Nigerian banks | Online survey complemented by face to face interviews, and telephone conference calls{18 banks} | topmost triggers for sustainable banking efforts by Nigerian banks: reputational benefits, regulatory requirements, operational benefits, and employee attraction and retention | No hypothesis testing |
| **9** | Weber and Oni (2015) - China, Bangladesh, Nigeria | Literature review, Index{10 DMBs in Nigeria. Data from annual reports, environmental reports, corporate social responsibility reports and sustainability reports of selected banks} | Mandatory guidelines have an impact on the sustainability performance of banks. Reporting mostly concentrated on financial inclusion, women's economic empowerment, environmental and social footprint of business operations | Not empirical with quantitative hypothesis testing. A binary score was assigned as score for disclosure of effort on the nine sustainable banking principles (NSBP) of CBN, and a bar chart plotted to compare year on year compliance with NSBP by banks |

**Source: Author's Analysis (2017)**

### 2.3.1 Gaps in Literature Reviewed

This section discusses the gaps identified in Table 2.1 above. The discussion first explains the general gaps before commenting on specific gaps related to each study variable.

A preponderance of studies above examined relationship/influence between/of corporate governance and financial performance, leaving gaps for study on relationship between corporate governance and sustainability (which combines financial and non-financial measures). Sustainability is considered the litmus test for today's businesses, even though the construct is at infancy stage of study in literature, with most studies being in developed countries, and fewer with empirical test of hypothesis in quantitative design. Sustainability has demonstrated its potential to foster the balanced development of three dimensions: economic, social and environmental, but only the first dimension is receiving increased attention in the business world (Liu and Zhuang 2013, Chardine-Baumann and Botta-Genoulaz 2014). This is also buttressed by Othman (2014) who suggested research into corporate governance; and sustainability (economic, social and environmental performance).

Most of the studies above treated corporate governance as one-dimensional construct from the internal governance mechanisms perspective, focusing on variable(s) from one of board structure, management structure, or ownership structure), whilst ignoring the complementarity and substitutability of internal and external governance mechanisms.

Most of the studies have adopted strictly quantitative research design methodology (Uwuigbe and Fakile, 2012; Ujunwa, 2012; Kajola, 2008) using panel data analysis of firm observations; a short time-frame of study (between 2 and 4 years) as the dataset, and ignoring the benefits of a mixed-method approach. Albassam (2014); McNulty et al., (2013); and Zattoni *et al.,* (2013) encourage researchers to employ the use of mixed-methods in exploring corporate governance behaviour in their analyses

From the literature review, studies that extended the discourse on corporate governance utilized observable data from developed countries where data are more abundant (Haan and Vlahu, 2016). In addition, some of their results were generalised without taking into consideration: the differences in social context (according to Claesens and Fan, 2002); Nam and Nam, 2004); and Zeitun and Gary, 2007); weaknesses of institutions in the developing countries; and other exogenous variables that may

distort the applicability of such findings in Nigeria such as religious, political and social systems. This observation cuts across the different mechanisms considered in this study – board, ownership, management. A questionnaire survey and semi-structured interview provides opportunities for respondents to reflect the local context in the study findings.

Several studies conducted in Nigeria especially on corporate governance focused on either non-banking firms (Kajola, 2008), or a mixture of banking firms and firms in other industries (Kyereboah-Coleman, 2007). Adams and Maher (2003), and Subrahmanyam, Rangan and Rosenstein (1997) agree that the governance structure of banking firms is significantly different from that of nonfinancial firms and the empirical findings of nonfinancial firms cannot be generalised to banks. Studies which focused on the banking industry in Nigeria (such as Akpan and Riman, 2012; Uwuigbe, 2011; Ahmad and Mansur, 2012; and Ikpefan, 2013) have not only reported mixed findings but excluded a key feature which distinguishes banking firms from non-banking firms – regulation. This key feature makes the traditional corporate governance approach, which promotes the exclusive primacy of the shareholders without considering the interests of other stakeholders insufficient. This study seeks to adopt multiple theories to investigate different dimensions of corporate governance and sustainability.

#### 2.3.1.1 Internal Governance Controls and Corporate Social Performance

Under the conceptualisation of internal governance controls, this study looks at board structure, management structure, and ownership structure. Corporate social performance was measured by social performance (towards employees, customers, community) and environmental performance. The empirical findings in table 2.1 above are based on studies that touched on the variables considered in this study. From the empirical review, all the studies identified were in developed countries or emerging markets, which may not be generalised to Nigeria. The studies also adopted secondary data, sourcing data from independent rating agencies such as KLD, IRRC, Datastream, ASSET4 and Bankscope. KLD ratings and its likes are based on extensive research by independent analysts and are used for investment decisions and advice, which appropriates as the main strengths of the ratings (Harrison and Freeman, 1999), they are also widely used by institutional money managers and by academics from a variety of fields (Cho and Patten, 2007; Mahoney and Roberts, 2007). A developing country such as Nigeria does not have equivalent independent agencies with detailed and accurate data about the variables used in the studies. Except for Wong and Wong (2015) and Wu and Shen (2013), other studies did not include banks. There was also the inconsistency in the use of CSR/CSP as an explanatory variable or outcome

variable, See Berrone and Gomez-Mejia (2009); Wu and Shen (2013) versus other highlighted studies. In addition, none of the studies combined board structure, management structure, and ownership structure as explanatory variables, thus neglecting the interdependence of the different mechanisms (as suggested by Arora and Petrova, 2009), nor adopted survey (as recommended by Walls, Berrone, and Phan, 2012, and Wong and Wong, 2015). Businesses focus only on community (philanthropy), ignoring employees, customers, and product responsibility. With the implementation of CBN's principles of sustainable banking, there is a need to understand the motive of banks in embracing social and environmental responsibility. This study seeks to fill the gap by examining how banks have used their internal governance controls to motivate their organisation towards corporate social performance.

#### 2.3.1.2 Ethical Leadership and Corporate Reputation

Nearly all the studies were not empirical and without hypotheses formulated for testing. The exception being the works of Love, Lim and Bednar (2017), and Eisenbeiss, Knippenberg, and Fahrbach (2015), which were conducted in developed countries. The local studies by Ogechukwu (2013), Enofe, Ekpulu, Onobun, and Onyeokweni (2015), Gberevbie (2011), and Yidawi (2005) did not attempt a causal analysis. Neves and Story (2013) also did not establish a causality inference, but more importantly ethical leadership was studied as an explanatory variable to organisational deviance; whilst focus was on supervisor's reputation rather than corporate reputation. There is therefore a dearth of studies on ethical leadership and corporate reputation. This study seeks to fill the gap by finding out the role of ethical leadership as a dimension of corporate governance in influencing corporate reputation in selected deposit money banks in Nigeria.

#### 2.3.1.3 Regulation and Financial Innovation

Most of the local and foreign studies under 'regulation' (item 9-16 in table 2.1) linked the outcome to bank risk-taking, efficiency, or performance. Similarly, most of the studies under financial innovation were not empirically based, whilst the few (such as O'connor and Rafferty, 2012; Perihan and Kienpin, 2017) were conducted in developed countries. Pinto and Sobreira (2010)'s study was conceptual, Frame and White's study was based on literature review. Only Stewart (2010), and Racic, Cvijanovic and Aralica (2007) considered regulation and financial innovation, but both were not empirically-based. This study therefore seeks to fill the gap by determining the role of regulation in enabling financial innovation in selected deposit money banks in Nigeria

#### 2.3.1.4 Agency Mechanisms and Financial Performance

There is a preponderance of literature on agency mechanisms and financial performance as identified by study 1-25 in table 2.1. Some of the common trends in the various studies include: secondary data domination, using panel data regression analysis; reliance on established databases such as Compustat, IRRC, Thomson Reuters Worldscope for studies in developed countries, deliberate exclusion of financial institutions or combination of financial institutions and firms from other industries, and a focus on only one or two corporate governance mechanisms as explanatory variables. Most local studies rely on Nigerian Stock Exchange (NSE) Fact book, which has limited information on featured firms. In addition, they are 'tertiary' source of data. This therefore restricts local researches on study variables to a few corporate governance measures such as board size, CEO duality, Audit Committee, Board composition and independence. Independent review of banks' annual reports and other sources of secondary data such as bank websites offers richer insight into information and more variables for measuring corporate governance practices of banks. This study seeks to fill the gap by extracting variables from the annual reports and other source documents to cover sparsely-tested variables in corporate governance literature such as board ethnicity, board gender, CEO age, CEO tenure, CEO remuneration, Total Board and Committee Meetings, Non-Performing Loans, TBQ, and Net Interest Margin.

The few studies on banks (such as Mnasri, 2015; Al-Amarneh, 2014; Aebi *et al.*, 2012) adopted traditional financial performance measures such as ROA, ROE as dependent variables without considering the risk/default measure represented by non-performing loan (except Akpan and Riman, 2012; Nyor and Mejabi, 2013; Islam, 2014). Nigerian deposit money banks are grappling with issues threatening their survival such as deteriorating non-performing loans, and operational inefficiencies - factors that may affect the financial soundness of a bank, yet only few studies considered the need for a portfolio of measures (ROA, ROE, TBQ, NIM, and NPL) which covers key bank performance categories of profitability, market, and risk/default. In addition, the researcher did not find any study combining interdependence of agency mechanisms from board structure (ethnicity, gender, and meetings), management structure (CEO age, tenure, compensation), and ownership structure (insider ownership). All the studies also adopted mono-method research design, which is quantitative design. This study bridges the gap by examining complementarity of agency mechanisms, as well as a portfolio of bank financial performance measures which include non-performing loans, and net interest margin.

#### 2.3.1.5 Corporate Governance Practices Disclosure and Sustainability Performance Reporting

Most of the studies (Weber and Oni, 2015; Kumar, 2013; and Aggarwal, 2013) were not empirically based. While Isikuk and Chizea (2017) observed the relationship between governance practices disclosure and sustainability reporting in five banks each from Nigeria and South Africa, the study was limited to only one-year review, and without a causative analysis. On the other hand, Bhasin (2010) and Ong and Djajadikerta (2016) focused on only one company in their studies. Common to all the studies is the selective approach to the explanatory variable (governance practices disclosure), with most of the studies focused on only a few dimensions of corporate governance mechanisms. In addition, only Rebai, Azaiez and Saidane (2012) developed a performance evaluation framework for measuring sustainability performance, albeit from an economic perspective, using marginal utility functions for several stakeholders.

In Eccles *et al.*, (2014)'s study on sustainability practices and performance, they relied on readily available sustainable practices ratings of companies provided by Thomson Reuters ASSET4 database, which is not available in developing countries such as Nigeria. In addition, the quasi-experiment methodology adopted lacked random assignment of treatment in a laboratory setting thus putting up the issue of causality between sustainable practices and performance up for debate; it also excluded financial institutions in their selected companies. The works of Rebai-Bouricha (2014) and Kumar (2013) on sustainable banking/performance, present stand-alone constructs, without a link to corporate governance. This study seeks to bridge the gap by empirically testing the relationship between governance practices and sustainability reporting, whilst developing an integrated sustainability report to measure the economic, social, and environmental performance of deposit money banks. In selecting indices for economic sustainability, this study considered financial innovation, and financial performance (which included financial performance measures relevant and peculiar to the Nigerian banking industry, such as non-performing loan ratio, capital adequacy ratio, earnings growth, leverage, loans-to-deposit ratio, and cost-to-income ratio, return on assets, return on equity, net interest margin and tobin's Q).

# CHAPTER THREE

## METHODOLOGY

### 3.0 Preamble

This section entails the research procedures employed in this study. These include the research design, population, sample size and techniques, sample frame, data collection methods, research instruments, constructs measurement, data analysis, and ethical considerations.

### 3.1 Research Design

The descriptive and inferential research design was adopted for this study to achieve in-depth understanding of the phenomena under investigation (Denzin and Lincoln, 1994; Maxwell, 2005), as well as to establish causal relationships between the independent and dependent variables i.e. corporate governance dimensions and sustainability.

As part of the research design, this study adopted an Ex-post facto approach which is suitable for investigating possible cause and effect relationships. This is useful in investigating the dynamics agency mechanisms, bank characteristics and financial performance in Nigerian banks (Simon and Goes, 2013; Kerlinger and Rint, 1986; Cohen, Manion and Morison, 2000). This method has been adopted severally in corporate governance studies (Yermack, 1996; Garba, Sanda, and Mikailu, 2005; Kajola, 2008).

The study also adopted the mixed-methods approach which combines both quantitative and qualitative data collection techniques and analysis procedures (survey, composite index, and semi-structured interview). This method allowed for research questions to be better answered and it afforded the researcher an opportunity to better evaluate the extent to which the findings are reliable (Tashakkori and Teddlie, 2003). It also enabled the researcher to provide better opportunities for the research questions to be answered, overcome weakness of a mono-method, and strengthen the validity of findings (Bryman, 1998), whilst allowing for the exhibition of greater confidence in findings (Webb, Campbell, Schwartz, and Sechrest, 1966). A convergent parallel mixed-method was utilized, which

involved simultaneously collecting, merging and using both quantitative and qualitative data, whilst the integration of findings was done at the interpretation level. The researcher started with the quantitative approach because there is a good pre-knowledge of the phenomenon to be studied.

## 3.2 Population of the Study

The target population consists of infinite employees in all the twenty-two (22) Deposit Money Banks in Nigeria as at 31 December, 2016, while the study population comprised fourteen thousand one hundred and forty-seven (14,147) estimated employees working in the Lagos branches of the eight (8) Deposit Money Banks (depicted in Table 3.1b below). The selected banks' annual reports provide total population bankwide for each of the eight banks, but the study population of employees in Lagos branches was extrapolated based on the total number of Lagos branches as a percentage of the bankwide (nationwide) branches. The selection of eight banks was based on adoption of inclusion criteria (Verhoef and Hilsden, 2004), which comprises of: (i). being a publicly listed bank, (ii). availability of complete eleven-year annual reports from 2006 to 2016, and (iii) a corresponding eleven-year stock market and financial accounting information among other requirements. The banks also contribute seventy-two percent (72%) of the total deposit base of DMBs as at 2016 financial year-end (CBN, 2016). The criteria help in meeting the requirements for a panel data analysis, which favours including only firms with several consecutive years of data (Yermack, 1996; Cheng et al., 2008, Ntim, 2009). Table 3.1a and 3.1b show the study population:

**Table: 3.1a: Selected Banks' Staff Strength and Branch Network**

| Bank | Total Bank Employees | Total Branches Nationwide # | Total Lagos Branches # |
|---|---|---|---|
| Access Bank | 4,104 | 312 | 105 |
| Diamond Bank | 4,950 | 273 | 93 |
| FCMB | 4,160 | 204 | 59 |
| Fidelity Bank | 3,420 | 240 | 59 |
| First Bank | 9,249 | 569 | 127 |
| GT Bank | 5,206 | 211 | 81 |
| UBA | 9,296 | 441 | 106 |
| Zenith Bank | 7,120 | 377 | 145 |
| **TOTAL** | **47,505** | **2,627** | **775** |

**Source: Annual Report (2016)**

**Table 3.1b: Study Population – Lagos Branches**

| Bank | Total Bank Employees (A) | Nationwide Branches (B) | Total Lagos Branches # (C) | Lagos Branches to All Branches (%) ( D ) =C/B | Estimated Lagos Branches Employees (E ) = D * A |
|---|---|---|---|---|---|
| Access Bank | 4,104 | 312 | 105 | 34 | 1381 |
| Diamond Bank | 4,950 | 273 | 93 | 34 | 1686 |
| FCMB | 4,160 | 204 | 59 | 29 | 1203 |
| Fidelity Bank | 3,420 | 240 | 59 | 25 | 841 |
| First Bank | 9,249 | 569 | 127 | 22 | 2064 |
| GT Bank | 5,206 | 211 | 81 | 38 | 1999 |
| UBA | 9,296 | 441 | 106 | 24 | 2234 |
| Zenith Bank | 7,120 | 377 | 145 | 38 | 2738 |
| **TOTAL** | **47,505** | **2,627** | **775** | | **14,147** |

**Source: Author's Computation (2017)**

Study population for employees in Lagos branches was adopted because Lagos State is the study scope. The banks were not willing to provide a list of their employees in Lagos state, hence the need to extrapolate based on available information in the annual reports.

## 3.3 Sample Size Determination

For the survey method, the sample size was determined based on a sample size calculator formula, which when read for a study population of fourteen thousand one hundred and forty-seven (14, 147) employees at 95% confidence level and a 3.5% Margin of error, gives a sample size of seven hundred and seventy-one (771). The sample size calculator was adopted because the "ever increasing demand for research has created a need for an efficient method of determining the sample size needed to be representative of a given population" (Krejcie and Morgan, 1970). It is also a commonly used method in organizational research (Bartlett, Kotrlik, and Higgins, 2001), hence, this study adopted a sample size of seven hundred and seventy-one (771) employees.

For the semi-structured interview, the selection of respondents was guided by recommendations of Haniffa and Hudaib, 2007 to focus on quality of data through the careful selection of interviewees, rather than focusing on the number of interviewees. Morse (2000) recommended between eight (8) and twelve (12) participants as sufficient sample size especially for a mixed-method approach where the objective is to supplement the result of the quantitative method. Guest, Bunce, and Johnson (2006); and

Iszatt-White (2011) both recommended ten (10). This study considered board members, and senior management staff in the sampled banks appropriate to provide relevant data for this study. A selection of fifteen (15) respondents was adopted for this study.

## 3.4 Sample Frame

The sample frame for this study was used to define the population of interest (Retzer, 2003). From the list of twenty-two (22) deposit money banks in Nigeria (CBN, 2016) as provided on CBN's website, the following banks - Access Bank, Diamond Bank, FCMB, Fidelity Bank, First Bank, GT Bank, UBA, and Zenith Bank were selected for this study. The sample frame for this study also covered the executive directors, the management and non-management cadres as obtained from the banks' annual reports.

## 3.5 Sampling Technique

This study adopted a multi-stage sampling technique, which involved proportional-to-size sampling technique, stratified sampling and purposive sampling technique. Multi-stage sampling technique allows for data collection costs savings, flexibility, and feasibility (Shimizu, 2005). The first stage (proportional-to-size sampling technique) involved allotting sample size to each bank based on their population in proportion to the total population. The stratified sampling involved breaking into strata, head-office employees along five (5) broad specialised functional areas – Executive management, Controls (Financial Control, Internal control and compliance, risk management), Business development (treasury, retail and corporate banking), Corporate Development (investor relations, corporate affairs, legal/secretarial), and Operations and Technology, as well as branch employees along two (2) broad areas - domestic operations and business development (marketing). This enhanced the identification of sub-groups within the study population and also created a sample which adequately represented the sub-groups (Yount, 2006). The third stage involved purposive sampling. Key informant technique was employed to ascertain the employees in the different strata. Table 3.2 below shows the distribution of copies of questionnaires. Each bank got allocation from the sample size (771) based on the percentage of its total staff strength relative to the aggregate for selected banks (47488). For instance, Access Bank with a staff strength of four thousand one hundred and four (4104) represents nine percent (9%) of the total staff strength in selected banks (47488), which when multiplied by the total sample size (771) translates approximately to sixty-seven (67) copies of questionnaires:

**Table 3.2 Allocation of copies of questionnaire**

| Bank | TOTAL | % | Total Copies |
|---|---|---|---|
| Access Bank | 4104 | 9 | 67 |
| Diamond Bank | 4950 | 10 | 80 |
| FCMB | 4143 | 9 | 67 |
| Fidelity | 3420 | 7 | 56 |
| First Bank | 9249 | 19 | 150 |
| GT Bank | 5206 | 11 | 85 |
| UBA | 9296 | 20 | 151 |
| Zenith Bank | 7120 | 15 | 116 |
| **TOTAL** | **47,488** | **100** | **771** |

**Source: Author's Computation (2017)**

## 3.6 Method and Sources of Data Collection

This study collected data from two sources – primary sources and secondary sources.

i. Primary sources: The primary source of data for this study came from responses of employees extracted from the administered questionnaire as well as the semi-structured interview conducted. Responses from the questionnaire were used to address objective 1-3, which are: To determine the influence of internal governance controls in enhancing corporate social performance; to determine the effect of ethical leadership in influencing corporate reputation; and to determine the role of regulation in promoting financial innovation. While the justification for including employees across all categories in a survey testing ethical leadership, regulation, financial innovation and corporate social performance may be self-explanatory, the practice in most studies (Manawaduge, 2012; Wepukhuku, 2016) is to restrict opinions on corporate governance practices to only board members and top management. This study adopts a different methodology because of the inherent flaws in current practice. Experience in developing countries such as Nigeria shows that top management staff in most cases may not be disposed to revealing frank, honest but negative information about their firms. Seeking opinions from employees across all levels provide a better opportunity to avoid a 'groupthink' syndrome (Janis, 1983). Employees are a critical bloc of stakeholders in any organisation, and there is a growing recognition that human capital is a source of competitive advantage. Concenrns that employees are not involved in board meetings and therefore may not be knowledgeable is not backed by evidence. Employees observe governance practices in their

organisations, and get feedback formally or through grapevine on board processes and operations. As a critical stakeholder, firms need to have the buy-in of employees to see the importance of corporate governance rather than just a shove-down-the-throat approach that comes with seeing it from a rule-enforcement mindset. By taking part in the survey, employees get enlightened on their role, and that of other stakeholders.

Questionnaires are most suitable for descriptive or explanatory research; work best with standardized questions that can be similarly interpreted by respondents (Robson, 2002), and normally require less skill and sensitivity to administer (Jankowicz, 2005), economical for large data collection, authoritative in general, easy to explain and understand, more control on the research process, and a popular method in strategic management studies (Solomon, Lin, Norton and Solomon, 2003). Semi-structured interview helps to explore the views, experiences, beliefs and/or motivations of respondents on social phenomena (Boyce and Neale, 2006).

ii. Secondary sources: The secondary source of data was used to address objective 4-6 of this study. For objective 4 (to determine the influence of agency mechanisms on financial performance) and objective 5 (to determine the moderating effect of bank characteristics on the influence of agency mechanisms on financial performance), this study extracted the measuring data for the variables of interest through a content analysis of the sampled banks’ financial statements and annual reports, banks’ websites, banks’ publications, stock market price reports, and industry statistics and reports. This is consistent with other studies (Hashim and Devi, 2010; Sanda, Mikailu, and Garba, 2005). For objective 6 (to determine the effect of corporate governance practices disclosure in enhancing sustainability performance reporting), this study adopted the use of a composite index for both the independent variable (corporate governance practices disclosure), and the dependent variable (sustainability performance reporting). This is consistent with other studies (Ameer and Othman, 2012, Ntim, 2011, Uwuigbe, 2011). Procedures adopted for the construction of the index and measurement are in section 3.7.1 below. Secondary data offered the researcher the benefits of time and money savings (Ghauri and Gronhaug 2005), provided a source that can be testable and checked relatively easily by others (Denscombe 2007), and allowed for access to quality data without obtrusion.

## 3.7 Measurement of the Research Variables

The focus of this study was to assess the degree to which corporate governance dimensions impact sustainability of Nigerian deposit money banks. Therefore, the constructs employed in this study were corporate governance dimensions, and sustainability. The items used to measure the variables were derived from a review of literature. Some of the literature on corporate governance dimensions and tested variables include:

i. Internal Governance Controls and Agency Mechanisms:

   Board structure - Varshney, Kaul and Vasal (2012); Jensen and Meckling (1976); Adams and Mehran (2012); Sun and Liu (2014); Peni (2014);

   Management Structure - Kyereboah-Coleman (2007); Lin, Kuo and Wang (2013); Schoar (2003), Campbell and Minguez-Vera (2010); Bhagat and Bolton (2008);

   Ownership Structure - Mork and Yeung (2003); Iturralde, Maseda and Arosa (2011); Fung and Tsai (2012);

ii. Regulation - Laeven and Levine (2009); Chu (2011);

iii. Ethical Leadership – Brown, Trevino, and Harrison (2005), Eisenbeiss, Knippenberg, and Fahrbach (2015);

iv. Corporate Governance Practices Disclosure OECD (2004); CBN (2009), Ntim (2011), Uwuigbe (2011).

For the moderating variable:

v. Bank characteristics - Hu and Izumida (2008); Boone, Field, Karpoff and Raheja (2007); Penman and Penman (2007).

Studies and literature on Sustainability and the measured variables include:

vi. Financial performance – Combs, Crook, and Shook (2005); Brown and Caylor (2006); Bhagat and Bolton (2008); Mnasri (2015);

vii. Corporate Social Performance - Waddock and Graves (1997); Arora and Petrova (2009), Berrone and Gomez-Mejia (2009); Walls, Phan, and Berrone (2011),

viii. Corporate reputation -Ljubojevic and Ljubojevic (2008), Chun, R (2005), Feldman, Bahamonde, and Bellido (2014), Walker and Dyck (2014);

ix. Financial innovation – Arnaboldi and Rossignoli (2015), Cherotich, Sang, Shisia, and Mutungu (2015), Stewart (2010), Ekpu (2015)

x. Sustainability – Bradford, Earp, Showalter and Williams (2017), Stankevicienea and Nikonorova (2013), Rebai, Azaiez, and Saidane (2012), Fauzi, Svensson and Rahman (2010), Roy, Sarker and Parvez (2015), Sebbatu (2008), Jaimes-Valdez and Jacobo-Hernandez (2016).

The aforementioned variables were included as appropriate in the questionnaire, panel data, content analysis, and constructed composite index depending on the research question.hypothesis.

Table 3.3 below shows the variables, sub-variables and measurement, data source and reference to the data source:

**Table 3.3 – Measurement of Research Variables**

| | **VARIABLE** | **SUB-VARIABLES MEASUREMENT** | **DATA SOURCE** | **REFERENCE** |
|---|---|---|---|---|
| | **DEPENDENT VARIABLES:** | | | |
| i | CORPORATE SOCIAL PERFORMANCE | | | |
| | | Social Performance - Employee | Questionnaire | Q: A1i (1-5) |
| | | Social Performance - Customer | Questionnaire | Q: A1ii (1-5) |
| | | Social Performance - Community Development | Questionnaire | Q: A1iii (1-5) |
| | | Environmental Performance | Questionnaire | Q: A2 (1-7) |
| | | | | |
| ii | CORPORATE REPUTATION | | | |
| | | Products and services quality | Questionnaire | Q: F 1 |
| | | Financial soundness | Questionnaire | Q: F 2 |
| | | Global competitiveness | Questionnaire | Q: F 3 |
| | | | | |
| iii | FINANCIAL INNOVATION | | | |
| | | Innovative products and services | Questionnaire | Q: C 1 |
| | | Information Communication Technology (ICT) | Questionnaire | Q: C 2 |
| | | operating systems and processes | Questionnaire | Q: C 3 |
| | | update of delivery channels | Questionnaire | Q: C 4 |
| | | | | |
| iv | FINANCIAL PERFORMANCE | | | |
| | | Return on Asset (ROA) | Annual Report | |
| | | Return on Equity (ROE) | Annual Report | |
| | | Non-Performing Loan (NPL) | Annual Report | |
| | | Net Interest Margin (NIM)<br>Tobin's Q | Annual Report<br>Banks' website + Daily Stock Market Price Reports (Historical) | |

| | VARIABLE | | SOURCE | REFERENCE |
|---|---|---|---|---|
| v | SUSTAINABILITY PERFORMANCE REPORTING | See further details below (3.7.1) | Index constructed by Researcher | Appendix "G" |
| | | | | |
| | **INDEPENDENT VARIABLES:** | | | |
| | | | | |
| i | INTERNAL GOVERNANCE CONTROLS | | | |
| | | Board of Directors | Questionnaire | Q: D 1 (1-7) |
| | | Management Structure | Questionnaire | Q: D 2 (1-6) |
| | | Ownership Structure | Questionnaire | Q: D 3 (1-5) |
| | | | | |
| ii | ETHICAL LEADERSHIP | | | |
| | | CEO's Personal Ethics | Questionnaire | Q: E (2, 3, 5, 6) |
| | | Ethical Programs | Questionnaire | Q: E (10, 11, 12) |
| | | Ethical Culture | Questionnaire | Q: E (1, 4, 7, 8, 9, 13) |
| | | | | |
| iii | REGULATION | | | |
| | | Quality of Supervision | Questionnaire | Q: B (1, 7, 9, 10) |
| | | Activity Restriction | Questionnaire | Q: E (3) |
| | | Deposit Insurance Scheme | Questionnaire | Q: E (6) |
| | | Entry/Exit Regulation | Questionnaire | Q: E (8) |
| | | Capital Requirement | Questionnaire | Q: E (4, 5, 11) |
| | | Disclosure | Questionnaire | Q: E (2) |
| | | | | |
| iv | AGENCY MECHANISMS | | | |
| | | Board Ethnicity | Annual Report | |
| | | Board Gender | Annual Report | |
| | | Insider Ownership | Annual Report | |
| | | CEO Age | Annual Report | |
| | | CEO Tenure | Annual Report | |
| | | CEO Remuneration | Annual Report | |
| | | Total Meetings | Annual Report | |
| | | | | |
| v | BANK CHARACTERISTICS | Bank Age, Size (Total Assets), Leverage | Annual Report | |
| | | | | |
| | | | | |
| vi | CORPORATE GOVERNANCE PRACTICES DISCLOSURE | See further details below (3.8.1) | Index constructed by Researcher | Appendix "F" |

**Source: Author's Computation (2017)**

### 3.7.1 Construction of Index – Corporate Governance and Sustainability Indices

Building an index allows for the use of a quantitative method to establish the relationship and influence between the independent and dependent variables, as quantitative disclosure provides the most verifiable evidence of performance.

Studies (Montiel and Delgado-Ceballos, 2014; Henri and Journeault (2008); and Brunklaus, Malmqvist and Baumann (2009) suggest means of collecting data to measure sustainability performance, through the use of sustainability index designed by rating agencies (such as KLD Index, Dow Jones Sustainability Index); or index and scales developed by the researcher using surveys or content analysis (Ameer and Othman, 2012). While the latter method has advantages, some of which include – cost savings, more relevance to the study context, transparency, and re-testability, the former has limitations such as the index suffering from applicability in other countries (Tilakasiri, 2013), inconsistency in data definition, methodological appropriateness, and standardisation (Mayer, 2008).

This study adopted four steps in constructing the index: Firstly, the researcher reviewed the provisions contained in the corporate governance codes as enacted by CBN and SEC, as well as adaptations from other studies (Black et al, 2003, Ntim, 2011, Uwuigbe, 2011) and extracted items for inclusion in the corporate governance practices disclosure index to measure the variable of interest. This process entailed ensuring face validity, unidimensionality, and the empirical relationships between the variables. Similar exercise was carried out by reviewing the CBN's sustainable banking principles, best practices (UNGC, 2013), adaptations from other studies found relevant to this study (Ameer and Othman, 2012), and extracts from literature for the sustainability reporting index. Secondly, the researcher categorised the items into similar sub-index in line with readings in literature. The third step involved scoring the index. The researcher is required to determine if scoring will be based on disclosure or performance. While studies (Herbohn, Walker, and Loo, 2014; Al-Tuwaijri, Christensen, and Hughes; 2004) showed evidence that a significant positive relationship exists between quality of disclosure and performance, the researcher needs to determine the quality of disclosure by selected banks to avoid 'greenwashing' (Baumgartner and Ebner, 2010). This study therefore adopted a scoring basis for non-disclosure, qualitative disclosure and quantitative disclosure as used in studies (Uwuigbe, 2011; Backstrom and Karlsson, 2015). Each bank was scored based on the level of disclosure on each evaluated item in the index (corporate governance practices index, and sustainability performance

index). Level of disclosure for corporate governance practices was extracted from their corporate governance report embedded in the annual reports, whilst disclosure on sustainability performance was extracted from a combination of sources such as (i). The sustainability report inserted in the annual report, (ii). Stand-alone sustainability reports (where available), and (iii). Additional information on sustainability performance on the websites (where available). A sample of scoring method is shown in table 3.4a (sample environmental performance scoring) and table 3.4b (sample financial performance scoring guide).

In choosing weights, the study was guided by the level of efforts required in fulfilling each item. For instance, if there is a requirement for an item to be disclosed, the weight of 1 is assigned, in which case a 1 is scored for disclosure and a 0 for non-disclosure. If a provision requires the establishment of a separate Board Nomination committee, and a Board Governance committee, a score of 1 is assigned out of a maximum 2 for a bank that has a combined Board Nomination and Governance committee. The weighting system for each measured item is disclosed in appendix “F” and “G”. The fourth and final step was the computation of the scores by individual banks. There are different approaches used in studies to compute the index scores. Some studies adopt a two-step process, first of which is to have the total score by each firm, and then a second step to determine whether to give weights to different sub-indices in the composite index. Assigning weights to different sub-indices may lead to bias or accord significance to some indices over others (Tilakasiri, 2013). Other studies argued that this assumption is not practical since some items have more importance than others. For the corporate governance practices disclosure index, this study adopted the unweighted approach for the total scores in order to avoid bias, because the measured items are quite straightforward – it’s either an item is disclosed or not, hence the decision is binary. The score of each firm is therefore calculated and expressed as a percentage of the total maximum score. For the sustainability reporting index however, the study adopted the weighted method. This is because the weight attached to economic/financial performance is adjudged by practitioners and stakeholders to be the most important, followed by social performance and environmental performance in that order. In addition, the financial performance was based on actual audited results, from which different ratios were computed (ROA, ROE, TBQ, NIM, Capital Adequacy, NPL, Liquidity proxied by Loan to Deposit ratio, Earnings growth, Leverage, and Cost-to-income ratio).

The study adopted a guide to determine the strength or weakness of each financial performance ratio indicator by the banks, with each item assigned a score of 3 or 5 based on the level of significance of the indicator. The total scores under economic/financial performance by each bank was thereafter weighted as fifty-percent (50%) of the total sustainability reporting index, social performance as thity-percent (30%), and environmental performance as twenty-percent (20%) to arrive at the one-hundred percent (100%) maximum score. The objects measured under social performance were categorised into: diversity, employment/labour, human rights, ethics, product responsibility, and community. Individual item score ranged from 0-3, where 0 means no evidence of practice, 1 means a line or word mention of the measured item, 2 means a general description in details, and 3 a general and specific description in details with figures or statistics. A sample of disclosure by four of the sampled banks on environmental sustainability (specifically energy saving initiative) is shown in table 3.4a below. The study also took into consideration instances of non-disclosure, which could be due to several reasons such as item being non-material, irrelevant, or a deliberate refusal to disclose. The researcher believes that firms are more likely to overstate what they do than under report, which is why the level of details in the disclosure is weighted.

**Table 3.4a: Sample Scoring Guide on Environmental Sustainability**

**ENVR005: Energy efficiency initiatives reporting:**

| | | | |
|---|---|---|---|
| **Bank A:** | | | |
| No mention of energy efficiency initiatives in annual report, website or stand-alone report | | | |
| | | | |
| **Bank B:** | | | |
| "….the bank monitors its carbon footprint. This is mainly done by actively tracking our energy consumption". | | | |
| | | | |
| **Bank C:** | | | |
| "We continue to improve efficiency and productivity by saving energy, switching to cleaner fuels and utilizing sustainably managed renewable energy sources to run our branches. Every day, we ensure that our branches and all head office locations close by 7pm while our alternate delivery channels continue to run on solar power". | | | |
| | | | |
| **Bank D:** | | | |
| We adopt energy efficiency practices in all our offices, through the use of LED energy saving bulbs, early branch closure, eco-friendly and energy-efficient equipment such as air-conditioners. Our records for the year are as follows: | | | |
| | | | |

| | Prior year | Current year | % Reduction |
|---|---|---|---|
| Total electricity purchased (kWh) | 4,132,144.72 | 2,856,362 | 30.9 |
| Total amount used per employee (kWh) | 3848.76 | 2,559 | 33.5 |
| Total Diesel Consumption (Ltr) | 2,002,025.30 | 1,856,392 | 7.27 |
| Total Diesel used per employee (ltr) | 1,873 | 1,717 | 8.33 |

**Source: Author's Computation (2017)**

Based on the level of disclosure above, Bank A scores 0, Bank B scores 1, Bank C scores 2, and Bank D scores 3.

Table 3.4b below shows the scoring guide on financial performance.

**Table 3.4b:** Scoring Guide on Financial Performance

| | |
|---|---|
| **SCORING GUIDE (Based on Rating agencies and Bankers Ratio Guide)** | **Maximum Score** |
| ROE: Negative = 0, Below 11% = 1, 11% and 20% = 2, Above 20% = 3 | 3 |
| ROA: Negative = 0, Below 1.6% = 1, 1.6% and 3% = 2, Above 3% = 3 | 3 |
| Earnings Growth: Negative = 0, Below 11% = 1, 11% and 20% = 2, Above 20% = 3 | 3 |
| Tobin's Q: Below 1 = 0, Btw 1 and 1.5 = 1, Above 1.5 = 3 | 3 |
| Cost-to-Income Ratio: 50% or Below = 5, 51 - 65% = 3, 66-75% = 1, Above 75% = 0 | 5 |
| Net Interest Margin: Negative = 0, Below 50% = 1, 50% and 60% = 2, Above 60% = 3 | 3 |
| Non-Performing Loans Ratio: Between 0 and 2% = 5, Btw 2 and 5% = 2, Above 5% = 0 | 5 |
| Loans-to-Deposit Ratio: Above 90% = 0, Between 80 and 89% = 1, 70-79% = 2, 50-69% = 3 | 3 |
| Leverage Ratio: Below 3% = 0, 3-5% = 3, Above 5% = 5 | 5 |
| Capital Adequacy Ratio: Between 0 and 15% = 0, >15 <= 20% = 3, Above 20% = 5 | 5 |
| | |
| TOTAL MAXIMUM SCORE ON FINANCIAL PERFORMANCE | **38** |

**Source: Author's Computation (2017)**

The scoring guide was based on recommendations in bankers' ratio guide, rating agencies guide, and discussions with deposit money bank executives. Scoring guide reflects a range as adjudged by banking and rating agency practitioners between 'poor' and 'excellent' performance.

### 3.7.1.1 Sustainability Reporting Index

The table below shows the sub-indices, the broad category of items measured, the maximum score obtainable in each category, and the weighted score of the major indices. The full details showing item by item measurement on the index is in appendix "F"

**Table: 3.5 Sustainability Reporting Index Measuring Scale (Streamlined Version)**

| Major Index | Major Sub-Index | Minor Sub-Index | Maximum Score | Weighted Score |
|---|---|---|---|---|
| **ECONOMIC** | Financial Ratios (10 items ) | ROA, ROE, TBQ, NIM, Capital Adequacy, NPLR, Loan to Deposit ratio, Earnings growth, Leverage, and Cost-to-income ratio (see Table 3.4b above) | 38 | 50% |
| | | | | |
| | Financial Inclusion (4 ) | SMEs, Literacy, delivery channels, and minority support | 8 | |
| **SOCIAL** | Diversity (11 ) | | 25 | 30% |
| | Human Rights (7) | | 7 | |
| | Ethics (90 | | 12 | |
| | Employment/Labor(10) | | 22 | |
| | Product Responsibility(5) | | 9 | |
| | Community (8) | | 16 | |
| **ENVIRONMENTAL** | Policies and Practices (17) | | | |
| | | Resources efficiency | 31 | 20% |
| | | Structures and Policies | | |
| | | Socially Responsible Investment | | |
| | | | | |
| **TOTAL** | | | | **100%** |

**Source: Author's Computation (2017)**

Table 3.5 shows the major items measured under sustainability performance reporting Index. For instance, Diversity (tested as a component of social performance) contained eleven (11) neasured items, yielding a maximum score of twenty-five (25)

### 3.7.1.2 Corporate Governance Practices Disclosure Index

The table below shows the sub-indices, the items measured, the maximum score obtainable in each category, and the weighted score of the major indices:

**Table: 3.6 Governance Practices Disclosure Index Measuring Scale (Streamlined Version)**

| SUB-INDICES | TOTAL ITEMS EVALUATED | MAX SCORE PER CATEGORY | % SCORE |
|---|---|---|---|
| Independence | 6 | 6 | |
| Committee | 8 | 13 | |
| Operations and processes | 23 | 24 | |
| Disclosure | 22 | 24 | |
| Risk Management | 18 | 33 | |
| Transparency and Integrity | 10 | 12 | |
| Shareholders rights | 16 | 16 | |
| **TOTAL** | | **128** | **100%** |

**Source: Author's Computation (2017).**

#### 3.7.1.3 Financial Performance

**Table 3.7: Measurement of financial performance**

| Variable | Measure | Use |
|---|---|---|
| ROA | Profit Before Tax / Total Assets | Hypothesis 4, 5 and 6 |
| ROE | Profit Before Tax / Total Equity | Hypothesis 4, 5 and 6 |
| TBQ | Equity Market Value / Equity Book Value | Hypothesis 4, 5 and 6 |
| NIM | $\frac{(\text{Interest Income} - \text{Interest Expenses})}{\text{Average Earning Assets}}$ | Hypothesis 4 |
| NPL | Non-Performing Loans | Hypothesis 4 |
| NPLR | Non-Performing Loans / Gross Loans | Hypothesis 6 |
| NIM (2) | $\frac{(\text{Interest Income} - \text{Interest Expenses})}{\text{Interest Income}}$ | Hypothesis 6 |
| Earnings growth | (Year 2 Earnings – Year 1 Earnings)/Year 1 Earnings | Hypothesis 6 |
| Cost-to-Income Ratio | Operating expenses before loan loss / Net operating income | Hypothesis 6 |
| Loan-to-Deposit Ratio | Net Total Loans and Advances / Total Deposits | Hypothesis 6 |
| Leverage Ratio | Total Assets / Shareholders funds | Hypothesis 5 and 6 |
| Capital Adequacy Ratio | Total Qualifying Capital / Risk-weighted assets | Hypothesis 6 |

**Source: Author's Computation (2017)**

Table 3.7 shows the formula used in computing the financial ratios

## 3.8 Design of Research Instrument

This study adopted questionnaire, semi-structured interview, and content analysis as research instruments.

### 3.8.1 The Questionnaire Survey

A set of structured questionnaires was used in gathering respondents' responses as basis of analysis in the study. The questionnaire was designed using the 5-point Likert scale. It was organized into three sections, A, B and C. The five-point Likert scale is simple to use, as a numerical value is attributed to the informant's opinion (Hussey and Hussey, 1997).

**Section A:** This section contained the demographic information of the respondents, that is, the biographical data with respect to gender, age, marital status, educational qualification, job function, job grade, and years spent in the organisation.

**Section B:** This section dealt with close-ended questions that relate to some of the research variables i.e. internal governance controls, regulation, ethical leadership, financial innovation, social responsibility (social performance and environmental performance), corporate reputation, and financial

performance. The five-point Likert scale had the following values: Strongly agree (5) Agree (4) Neutral (3) Disagree (2) Strongly Disagree (1), and in the case of financial performance Excellent (5) Above average (4) Average (3) Below Average (2) Very Poor (1).

**Section C:** This section contained open-ended questions requiring information on some of the study variables.

### 3.8.2 The Semi-Structured Interview

The semi-structured interview was based on a list of dimensions and questions in the study. It consisted of simple and short questions presented in open-ended format that permitted respondents to extensively share their personal opinion on the relationship between corporate governance and sustainability performance. Each interview lasted between 30 minutes and 90 minutes and was held at the respondent's office or as recommended by respondent. An interview guide steered the discussions around the main corporate governance and sustainability themes (See Appendix "B").

### 3.8.3 Content Analysis

Content analysis was used to provide measures for the agency mechanisms; bank characteristics, corporate governance practices disclosure index; and sustainability index. The contents for analysis and measure were derived from: sampled banks' financial statements and annual reports, websites, sustainability reports, corporate governance reports, stock-price online portal, industry statistics and reports, and regulatory bodies' websites (CBN/NDIC/SEC) etc.

## 3.9 Pre-distribution of Research Instruments –Pilot Testing

The researcher conducted a pilot study before the main data collection exercise commenced. Hoyle and Ingram (1991); Isaac and Michael (1995); and Hill (1998) suggested 10 to 30 participants for pilots in survey research. The pilot test for this study involved sixty (60) distributed questionnaires to randomly selected employees in deposit money banks excluded from the main study. This approach allowed for broader participation by the pilot respondents, which assisted the researcher in identifying potential problems that may be inherent in the research instrument. Forty-four (44) questionnaires were retrieved and found usable.

## 3.10 Validity of Research Instrument

The questionnaire used in this study addressed validity threats through face and content validity by experts' review of the questionnaire. Experts in this case refer to the researcher's supervisors, senior scholars in business management, accounting, banking, and statistics, as well as practitioners in the banking industry. They were provided with access to the measurement tool, and their feedback on the effectiveness of each question in measuring the constructs was considered and reflected in the final questionnaire.

This study also used Confirmatory Factor Analysis (CFA) to assess the scale validity and the fit of the measurement model. The convergent phase was adopted for the validation of the items. The study adopted three (3) conditions to assess convergent validity as shown in Table 3.6 below. The three conditions are, first, the CFA loadings indicate that all scale and measurement items are significant and exceed the minimum value criterion of 0.70. Second each construct Composite Reliability (CR) exceeds 0.80. Third, each construct's Average Variance Extracted Estimate (AVE) exceeds 0.50. From the results, conditions 2 and 3 were met fully, while six (6) out of the seventy-five (75) items under condition 1 were marginally below the set criterion. Thus, this implies that most of the conditions for convergent validity as suggested and recommended by Fornell and Larcker (1981) and Bagozzi and Yi (1988) are met.

For the secondary data/ content analysis, several studies (Kyereboah-Coleman, 2007; Uwuigbe and Fakile, 2012; Varshney, Kaul and Vasal, 2012) have adopted the use of index, content analysis methods in answering research questions on corporate governance and performance, and therefore supports the measurement validity of this method. Semi-structured interview was validated by triangulating data from different sources and a well-documented audit trail of materials and processes.

**Table 3.8. Validity Test**

| Measurement | Loading | Indicator Reliability | Error Variance | Compose Reliability | Ave. Variance Estimated |
|---|---|---|---|---|---|
| | ≥ 0.7 | | ≤ 0.5 | ≥ 0.8 | ≥ 0.5 |
| **A. SOCIAL PERFORMANCE** | | | | | |
| **Employees Relationship: In my bank ….** | | | | | |
| ER1 | 0.7499 | 0.5624 | 0.4376 | | |
| ER2 | 0.7019 | 0.4927 | 0.5073 | 0.8574 | 0.7396 |

| | | | | | |
|---|---|---|---|---|---|
| ER3 | 0.7176 | 0.5149 | 0.4851 | | |
| ER4 | 0.826 | 0.6823 | 0.3177 | | |
| ER5 | 0.6947 | 0.4826 | 0.5174 | | |
| **Customers Relationship** | | | | | |
| CR1 | 0.7675 | 0.5891 | 0.4109 | 0.8819 | 0.7743 |
| CR2 | 0.7132 | 0.5087 | 0.4913 | | |
| CR3 | 0.7878 | 0.6206 | 0.3794 | | |
| CR4 | 0.8002 | 0.6403 | 0.3597 | | |
| CR5 | 0.7994 | 0.639 | 0.361 | | |
| **Community and Society Relationship** | | | | | |
| CSR1 | 0.7918 | 0.6269 | 0.3731 | 0.8708 | 0.758 |
| CSR2 | 0.6876 | 0.4728 | 0.5272 | | |
| CSR3 | 0.7753 | 0.6011 | 0.3989 | | |
| CSR4 | 0.7667 | 0.5878 | 0.4122 | | |
| CSR5 | 0.7645 | 0.5845 | 0.4155 | | |
| **B. REGULATION** | | | | | |
| BRS1 | 0.7547 | 0.5696 | 0.4304 | 0.9301 | 0.7406 |
| BRS2 | 0.8622 | 0.7434 | 0.2566 | | |
| BRS3 | 0.7183 | 0.516 | 0.484 | | |
| BRS4 | 0.7649 | 0.5851 | 0.4149 | | |
| BRS5 | 0.7111 | 0.5057 | 0.4943 | | |
| BRS6 | 0.6846 | 0.4687 | 0.5313 | | |
| BRS7 | 0.7043 | 0.496 | 0.504 | | |
| BRS8 | 0.6967 | 0.4854 | 0.5146 | | |
| BRS9 | 0.7954 | 0.6327 | 0.3673 | | |
| BRS10 | 0.6969 | 0.4857 | 0.5143 | | |
| BRS11 | 0.7382 | 0.5449 | 0.4551 | | |
| **C. FINANCIAL INNOVATION** | | | | | |
| FI1 | 0.8675 | 0.7526 | 0.2474 | 0.8446 | 0.7602 |
| FI2 | 0.7222 | 0.5216 | 0.4784 | | |
| FI3 | 0.7398 | 0.5473 | 0.4527 | | |
| FI4 | 0.7002 | 0.4903 | 0.5097 | | |
| **D. ENVIRONMENTAL PERFORMANCE: My bank …** | | | | | |
| EP1 | 0.8738 | 0.7635 | 0.2365 | 0.9198 | 0.7684 |
| EP2 | 0.6883 | 0.4738 | 0.5262 | | |
| EP3 | 0.7638 | 0.5834 | 0.4166 | | |
| EP4 | 0.8174 | 0.6681 | 0.3319 | | |
| EP5 | 0.7285 | 0.5307 | 0.4693 | | |

| | | | | | |
|---|---|---|---|---|---|
| EP6 | 0.7784 | 0.6059 | 0.3941 | | |
| EP7 | 0.7798 | 0.6081 | 0.3919 | | |

**E. GOVERNANCE MECHANISMS**

| | | | | | |
|---|---|---|---|---|---|
| **Board of Directors** | | | | | |
| BoD1 | 0.7347 | 0.5398 | 0.4602 | 0.899 | 0.7257 |
| BoD2 | 0.7143 | 0.5102 | 0.4898 | | |
| BoD3 | 0.7261 | 0.5272 | 0.4728 | | |
| BoD4 | 0.7143 | 0.5102 | 0.4898 | | |
| BoD5 | 0.7311 | 0.5345 | 0.4655 | | |
| BoD6 | 0.7283 | 0.5304 | 0.4696 | | |
| BoD7 | 0.7299 | 0.5328 | 0.4672 | | |
| **Chief Executive Officer (CEO) / Executive Management** | | | | | |
| CEO1 | 0.7222 | 0.5216 | 0.4784 | 0.8745 | 0.7332 |
| CEO2 | 0.7155 | 0.5119 | 0.4881 | | |
| CEO3 | 0.733 | 0.5373 | 0.4627 | | |
| CEO4 | 0.7144 | 0.5104 | 0.4896 | | |
| CEO5 | 0.741 | 0.5491 | 0.4509 | | |
| CEO6 | 0.7714 | 0.5951 | 0.4049 | | |
| **Share Ownership Structure** | | | | | |
| SoS1 | 0.7347 | 0.5398 | 0.4602 | 0.841 | 0.7182 |
| SoS2 | 0.6683 | 0.4466 | 0.5534 | | |
| SoS3 | 0.7999 | 0.6398 | 0.3602 | | |
| SoS4 | 0.6269 | 0.393 | 0.607 | | |
| SoS5 | 0.7484 | 0.5601 | 0.4399 | | |
| **F. ETHICAL LEADERSHIP** | | | | | |
| ELC1 | 0.7777 | 0.6048 | 0.3952 | 0.9443 | 0.753 |
| ELC2 | 0.8522 | 0.7262 | 0.2738 | | |
| ELC3 | 0.7228 | 0.5224 | 0.4776 | | |
| ELC4 | 0.8442 | 0.7127 | 0.2873 | | |
| ELC5 | 0.6977 | 0.4868 | 0.5132 | | |
| ELC6 | 0.7849 | 0.6161 | 0.3839 | | |
| ELC7 | 0.7652 | 0.5855 | 0.4145 | | |
| ELC8 | 0.6859 | 0.4705 | 0.5295 | | |
| ELC9 | 0.7666 | 0.5877 | 0.4123 | | |
| ELC10 | 0.6854 | 0.4698 | 0.5302 | | |
| ELC11 | 0.7643 | 0.5842 | 0.4158 | | |
| ELC12 | 0.6889 | 0.4746 | 0.5254 | | |

| | | | | | |
|---|---|---|---|---|---|
| ELC13 | 0.7281 | 0.5301 | 0.4699 | | |
| **G. CORPORATE REPUTATION** | | | | | |
| CR1 | 0.8817 | 0.7774 | 0.2226 | 0.9038 | 0.8707 |
| CR2 | 0.8526 | 0.7269 | 0.2731 | | |
| CR3 | 0.8775 | 0.77 | 0.23 | | |
| **H. FINANCIAL PERFORMANCE of my bank relative to industry-average** | | | | | |
| FP1 | 0.7345 | 0.5395 | 0.4605 | 0.8338 | 0.7464 |
| FP2 | 0.8043 | 0.6469 | 0.3531 | | |
| FP3 | 0.6961 | 0.4846 | 0.5154 | | |
| FP4 | 0.7466 | 0.5574 | 0.4426 | | |

NOTE: All loadings in Table ** are significant at $p < 0.0001$.

**Source: Author's Computation (2017)**

## 3.11 Reliability of Research Instrument

To measure reliability, this study adopted inter-item reliability method, which involved using PASW 23 (SPSS) to analyse the Cronbach Alpha internal consistency measurement of the items included in the questionnaire administered for pilot test. According to George and Mallery (2003), the rule of thumb that is generally acceptable is as follows:

**Table 3.9. Cronbach Alpha Statistics Interpretation**

| | |
|---|---|
| $\alpha \geq 0.9$ | Excellent (High- Stakes testing) |
| $0.7 \leq \alpha < 0.9$ | Good (Low- Stakes testing) |
| $0.6 \leq \alpha < 0.7$ | Acceptable |
| $0.5 \leq \alpha < 0.6$ | Poor |
| $\alpha < 0.5$ | Unacceptable |

**Source: George and Mallery (2003)**

Based on the test, the table below shows the result:

**Table 3.10: Reliability Statistics**

| Cronbach's Alpha | No of Items |
|---|---|
| .926 | 75 |

**Source: Author's Computation (2017)**

The result indicated a good internal consistency in the instrument used based on the Cronbach's alpha coefficient value reported at 0.926.

For the secondary data, reliability is a function of the source and data collection method, which is determined by the authority or reputation of the source (Dochartaigh, 2002). Annual reports and financial statements constitute the most significant pieces of secondary data in this study phase. They go through very rigorous process including certification by reputable auditors, review and certification by CBN, SEC, NDIC, and presentation at the Annual General Meetings. There are laws and regulations guiding financial reporting and the consequences of inaccurate or misleading financial reporting. Therefore, annual reports and financial statements especially by the big six audit firms are considered reliable and trustworthy source of data. This study adopted a detailed documentation of the steps and procedures followed in the semi-structured interview, cross-checking of the interview transcripts and consistent definition and meaning of codes and scored used (Gibbs, 2007).

## 3.12 Methods of Data Analysis

### 3.12.1 Quantitative Data

Descriptive and inferential methods of analysis was adopted for this research. The former was used to analyse the demographic characteristics of the respondents, while the latter was used to test the formulated hypotheses. Multiple regression was used for objective 1-3, and 6, while panel regression was used for objective 4 and 5. Panel data analysis is widely used in corporate governance literature (Kajola, 2008, Uwuigbe, 2011) for testing relationships and effects of corporate governance mechanisms on firm performance. It offers several benefits: allows for the combination of time series with cross-section; ability to reveal effects hidden in strictly cross-sections or time-series data; most appropriate for longitudinal analyses and data gathered at micro units such as individuals, firms and households (Baltagi, 1995). Panel data analysis was carried out using Eviews Version 9 software. Analysis included descriptive statistics – mean score, minimum, maximum, and standard deviation. The multiple regression analysis, which involved correlations was thereafter used to determine the relationship, and influence of the independent variables on the dependent variables. Multiple regression is used to predict the value of a variable (dependent) based on the value of two or more other variables (independent). It also models the relationship between two or more explanatory variables and a response variable, and accommodates continuous predictors and categorical predictors. It also involves one continuous outcome.

To use multiple regression, the study considered the prerequisite assumptions and tests on the data collected, which include: dependent variable is measured on a continuous scale (as either an interval or ratio variable), two or more independent variables, which can be either continuous (i.e., an interval or ratio variable) or categorical (i.e., an ordinal or nominal variable), independence of observations, a linear relationship between the dependent variable and individual independent variable as well as collective independent variables, homoscedasticity, and non-multicollinearity among others. The five-point likert-scale data used in the questionnaire (with values "strongly agree", "agree", "neutral", "disagree" and "strongly disagree) were equally spaced, hence the masured variables qualify as continuous data, of the interval type (Subedi, 2016; Sullivan and Artino, 2013; Boone and Boone, 2012; Lubke, Gitta, Muthen, and Bengt, 2004; Glass, Peckham, and Sanders, 1972).

Indexes are very useful in quantitative research because they "provide a researcher a way to create a composite measure that summarizes responses for multiple rank-ordered related questions or statements" (Crossman, 2017).

For objective 1-3, and 6, the data collected from the questionnaire, Corporate Governance Practices Index, and Sustainability Performance Index was subjected to relevant statistical analysis using IBM PASW (formerly SPSS) version 23. Descriptive statistics was used to determine the frequency distribution and percentage analysis, mean, minimum, maximum and standard deviation scores of both the dependent and independent variables. It was also used to analyse the demographic characteristics of the respondents. In addition, a Structural Equation Modelling was used to determine the goodness of fit of objectives 1-3 tested by questionnaire.

### 3.12.2 Qualitative Data

Semi-structured interviews were recorded using a Dictaphone. The recordings were transcribed and analysed to identify the data themes (Yin, 2011). The findings from the semi-structured interviews were methodologically triangulated (Bekhet and Zauszniewski, 2012) with content analysis of the CBN's code of corporate governance, CBN's Sustainable banking principles, OECD corporate governance principles, and the selected banks' records, such as annual reports, websites, and other available archival record. The study adopted a manual transcription.

## 3.13 Ethical Considerations

Ethical issues involved in research aim to ensure that the research is designed taking certain factors into consideration: the research population is not embarrassed, harmed or disadvantaged; participant's consent; participant's confidentiality; and misrepresenting results (Cooper, Schindler and Sun, 2006). This research adopted a deontological view which argues that the ends served by the research can never justify the use of unethical research methods. The cover letter for the survey clearly stated the study benefits and participants' rights and protections, anonymity of participants, intended purpose of the data, and a voluntary participation clause. In addition, the results from the survey are only reported in aggregate form in order to avoid the identification of individual responses from participants. For the interview, this study took into consideration and exercised due care in gaining accessibility to the proposed banks, data sources and participants; respondent's consent; and maintaining confidentiality of data during analysis and interpretation of the results (Albassam, 2014).

# CHAPTER FOUR

## DATA PRESENTATION AND ANALYSIS

### 4.0 Preamble

This chapter presents the results of analysis of data collected, tested hypotheses, as well as tables employed to illustrate the data gathered for this study. The data were analysed using the descriptive statistics based on the research objectives and the hypotheses formulated for the study. The hypotheses formulated were tested mainly through the use of multiple regression and Structural Equation Modelling.

### 4.1 Data Presentation

The data obtained for this study were presented using tables specifying relevant information on the copies of questionnaire distributed and retrieved from the respondents. A total of seven hundred and seventy-one (771) copies of questionnaire were randomly administered to the employees of selected banks in Lagos state. Five hundred and seventy-three (573) copies of the questionnaire were retrieved and found usable for analysis, which amounted to 74.3% response rate (see Table 4.1).

**Table 4.1: Breakdown (in Aggregate) of Questionnaire Distribution and Retrieval**

| S/N | Name of Banks | Bank Code | Total Distributed | Total Retrieved | % Retrieved | Total Not Retrieved | % Not Retrieved |
|---|---|---|---|---|---|---|---|
| 1 | Access | BK # 1 | 67 | 37 | 55.2 | 30 | 44.8 |
| 2 | Diamond | BK # 2 | 80 | 77 | 96.2 | 3 | 3.8 |
| 3 | FCMB | BK # 3 | 67 | 62 | 91.2 | 6 | 8.8 |
| 4 | Fidelity | BK # 4 | 56 | 43 | 76.8 | 13 | 23.2 |
| 5 | First Bank | BK # 5 | 150 | 103 | 68.7 | 47 | 31.3 |
| 6 | GT Bank | BK # 6 | 84 | 66 | 78.6 | 18 | 21.4 |
| 7 | UBA | BK # 7 | 151 | 100 | 66.2 | 51 | 33.8 |
| 8 | Zenith | BK # 8 | 116 | 85 | 73.3 | 31 | 26.7 |
| **Total** | | | **771** | **573** | **74.3** | **198** | **25.7** |

**Source: Author's Computation (2017)**

Table 4.1 above shows the breakdown (in aggregate) of questionnaire distribution and retrieval. The information presented in the table spread across the eight (8) deposit money banks used in this research work. Total copies of administered questionnaire not retrieved was 198 representing 25.7 percent. This response rate was considered good as 573 copies of the questionnaire were found usable for this study.

## 4.2 Demographic Characteristics of Respondents

This section describes the demographic characteristics of the respondents. Important variables considered were gender, age, marital status, highest education level, job function, job position, and years spent in the current bank as presented in Table 4.2 below:

**Table 4.2: Demographic Characteristics of Respondents**

| Bio-Data Info | Items | BK#1 | BK#2 | BK#3 | BK#4 | BK#5 | BK#6 | BK#7 | BK#8 | Total | Total |
|---|---|---|---|---|---|---|---|---|---|---|---|
| | | % | % | % | % | % | % | % | % | freq | % |
| **Gender** | Male | 26 | 50 | 35 | 26 | 65 | 34 | 61 | 50 | 347 | 60.56 |
| | | *(70.3)* | *(64.9)* | *(56.5)* | *(60.5)* | *(63.1)* | *(51.5)* | *(61.0)* | *(58.8)* | | |
| | Female | 11 | 27 | 27 | 17 | 38 | 32 | 39 | 35 | 226 | 39.44 |
| | | *(29.7)* | *(35.1)* | *(43.5)* | *(39.5)* | *(36.9)* | *(48.5)* | *(39.0)* | *(41.2)* | | |
| | **Total** | **37** | **77** | **62** | **43** | **103** | **66** | **100** | **85** | **573** | **100.00** |
| **Age Grade** | 18-24 | 0 | 1 | 2 | 1 | 2 | 1 | 4 | 3 | 14 | 2.44 |
| | | *0.0* | *(1.3)* | *(3.2)* | *(2.3)* | *(1.9)* | *(1.5)* | *(4.0)* | *(3.5)* | | |
| | 25-34 | 8 | 30 | 32 | 10 | 43 | 26 | 61 | 34 | 244 | 42.58 |
| | | *(21.6)* | *(39.0)* | *(51.6)* | *(23.3)* | *(41.7)* | *(39.4)* | *(61.0)* | *(40.0)* | | |
| | 35-44 | 19 | 36 | 21 | 28 | 47 | 34 | 35 | 38 | 258 | 45.03 |
| | | *(51.4)* | *(46.8)* | *(33.9)* | *(65.1)* | *(45.6)* | *(51.5)* | *(35.0)* | *(44.7)* | | |
| | 45-54 | 10 | 10 | 7 | 4 | 11 | 5 | 0 | 10 | 57 | 9.95 |
| | | *(27.0)* | *(13.0)* | *(11.3)* | *(9.3)* | *(10.7)* | *(7.6)* | *0.0* | *(11.8)* | | |
| | **Total** | **37** | **77** | **62** | **43** | **103** | **66** | **100** | **85** | **573** | **100.00** |
| **Marital Status** | Single | 3 | 15 | 18 | 6 | 21 | 23 | 38 | 16 | 140 | 24.43 |
| | | *(8.1)* | *(19.5)* | *(29.0)* | *(14.0)* | *(20.4)* | *(34.8)* | *(38.0)* | *(18.8)* | | |
| | Married | 34 | 61 | 42 | 37 | 82 | 42 | 61 | 69 | 428 | 74.69 |
| | | *(91.9)* | *(79.2)* | *(67.7)* | *(86.0)* | *(79.6)* | *(63.6)* | *(61.0)* | *(81.2)* | | |
| | Divorced | 0 | 1 | 2 | 0 | 0 | 0 | 1 | 0 | 4 | 0.70 |
| | | *0.0* | *(1.3)* | *(3.2)* | *0.0* | *0.0* | *0.0* | *(1.0)* | *0.0* | | |
| | Separated | 0 | 0 | 0 | 0 | 0 | 1 | 0 | 0 | 1 | 0.17 |
| | | *0.0* | *0.0* | *0.0* | *0.0* | *0.0* | *(1.5)* | *0.0* | *0.0* | | |
| | **Total** | **37** | **77** | **62** | **43** | **103** | **66** | **100** | **85** | **573** | **100.00** |

**Table 4.2: Demographic Characteristics of Respondents *Contd***

| Bio-Data Info | Items | BK#1 | BK#2 | BK#3 | BK#4 | BK#5 | BK#6 | BK#7 | BK#8 | Total | Total |
|---|---|---|---|---|---|---|---|---|---|---|---|
| | | % | % | % | % | % | % | % | % | freq | % |
| **Highest Education** | HND/BSc | 18 | 30 | 30 | 24 | 49 | 30 | 62 | 35 | 278 | 48.52 |
| | | *(48.6)* | *(39.0)* | *(48.4)* | *(55.8)* | *(47.6)* | *(45.5)* | *(62.0)* | *(41.2)* | | |
| | MSc/MBA/MEd | 18 | 46 | 28 | 18 | 52 | 31 | 36 | 48 | 277 | 48.34 |
| | | *(48.6)* | *(59.7)* | *(45.2)* | *(41.9)* | *(50.5)* | *(47.0)* | *(36.0)* | *(56.5)* | | |
| | Doctorate | 1 | 1 | 2 | 0 | 2 | 3 | 1 | 1 | 11 | 1.92 |
| | | *(2.7)* | *(1.3)* | *(3.2)* | *0.0* | *(1.9)* | *(4.5)* | *(1.0)* | *(1.2)* | | |
| | Others | 0 | 0 | 2 | 1 | 0 | 2 | 1 | 1 | 7 | 1.22 |
| | | *0.0* | *0.0* | *(3.2)* | *(2.3)* | *0.0* | *(3.0)* | *(1.0)* | *(1.2)* | | |
| | **Total** | **37** | **77** | **62** | **43** | **103** | **66** | **100** | **85** | **573** | **100.00** |
| **Job function** | Business Dev | 6 | 7 | 15 | 1 | 19 | 25 | 19 | 6 | 98 | 17.10 |
| | | *(16.2)* | *(9.1)* | *(24.2)* | *(2.3)* | *(18.4)* | *(37.9)* | *(19.0)* | *(7.1)* | | |
| | Ops and Tech | 4 | 5 | 20 | 8 | 37 | 15 | 26 | 29 | 144 | 25.13 |
| | | *(10.8)* | *(6.5)* | *(32.3)* | *(18.6)* | *(35.9)* | *(22.7)* | *(26.0)* | *(34.1)* | | |
| | Risk Mgt | 20 | 39 | 18 | 22 | 30 | 16 | 42 | 48 | 235 | 41.01 |
| | | *(54.1)* | *(50.6)* | *(29.0)* | *(51.2)* | *(29.1)* | *(24.2)* | *(42.0)* | *(56.5)* | | |
| | Corporate Dev | 4 | 13 | 4 | 12 | 8 | 4 | 6 | 1 | 52 | 9.08 |
| | | *(10.8)* | *(16.9)* | *(6.5)* | *(27.9)* | *(7.8)* | *(6.1)* | *(6.0)* | *(1.2)* | | |
| | Others | 3 | 13 | 5 | 0 | 9 | 6 | 7 | 1 | 44 | 7.68 |
| | | *(8.1)* | *(16.9)* | *(8.1)* | *0.0* | *(8.7)* | *(9.1)* | *(7.0)* | *(1.2)* | | |
| | **Total** | **37** | **77** | **62** | **43** | **103** | **66** | **100** | **85** | **573** | **100.00** |
| **Job Position** | Trainee | 0 | 8 | 12 | 1 | 24 | 10 | 4 | 8 | 67 | 11.69 |
| | | *0.0* | *(10.4)* | *(19.4)* | *(2.3)* | *(23.3)* | *(15.2)* | *(4.0)* | *(9.4)* | | |
| | Banking Officer | 17 | 29 | 31 | 24 | 60 | 30 | 39 | 47 | 277 | 48.34 |
| | | *(45.9)* | *(37.7)* | *(50.0)* | *(55.8)* | *(58.3)* | *(45.5)* | *(39.0)* | *(55.3)* | | |
| | Mgt | 17 | 34 | 7 | 9 | 17 | 15 | 53 | 27 | 179 | 31.24 |
| | | *(45.9)* | *(44.2)* | *(11.3)* | *(20.9)* | *(16.5)* | *(22.7)* | *(53.0)* | *(31.8)* | | |
| | Senior Mgt | 3 | 6 | 12 | 9 | 1 | 10 | 4 | 3 | 48 | 8.38 |
| | | *(8.1)* | *(7.8)* | *(19.4)* | *(20.9)* | *(1.0)* | *(15.2)* | *(4.0)* | *(3.5)* | | |
| | Executive Mgt | 0 | 0 | 0 | 0 | 1 | 1 | 0 | 0 | 2 | 0.35 |
| | | *0.0* | *0.0* | *0.0* | *0.0* | *(1.0)* | *(1.5)* | *0.0* | *0.0* | | |
| | **Total** | **37** | **77** | **62** | **43** | **103** | **66** | **100** | **85** | **573** | **100.00** |

**Table 4.2: Demographic Characteristics of Respondents *Contd***

| Bio-Data Info | Items | BK#1 | BK#2 | BK#3 | BK#4 | BK#5 | BK#6 | BK#7 | BK#8 | Total | Total |
|---|---|---|---|---|---|---|---|---|---|---|---|
| | | % | % | % | % | % | % | % | % | freq | % |
| **Years Spent** | Less than 3 years | 0 | 7 | 11 | 2 | 15 | 6 | 15 | 6 | 62 | 10.82 |
| | | *0.0* | *(9.1)* | *(17.7)* | *(4.7)* | *(14.6)* | *(9.1)* | *(15.0)* | *(7.1)* | | |
| | 3-5 years | 9 | 18 | 20 | 6 | 25 | 17 | 33 | 23 | 151 | 26.35 |
| | | *(24.3)* | *(23.4)* | *(32.3)* | *(14.0)* | *(24.3)* | *(25.8)* | *(33.0)* | *(27.1)* | | |
| | 6-10 years | 17 | 32 | 17 | 25 | 45 | 25 | 39 | 43 | 243 | 42.41 |
| | | *(45.9)* | *(41.6)* | *(27.4)* | *(58.1)* | *(43.7)* | *(37.9)* | *(39.0)* | *(50.6)* | | |
| | 10 years and above | 11 | 20 | 14 | 10 | 18 | 18 | 13 | 13 | 117 | 20.42 |
| | | *(29.7)* | *(26.0)* | *(22.6)* | *(23.3)* | *(17.5)* | *(27.3)* | *(13.0)* | *(15.3)* | | |
| | **Total** | **37** | **77** | **62** | **43** | **103** | **66** | **100** | **85** | **573** | **100.00** |

**Source: Author's Computation (2017)**

**Table 4.2.1 Code for sampled banks**

| | | | |
|---|---|---|---|
| BK#1 - Access Bank | BK#3 - FCMB | BK#5 - First Bank | BK#7 - UBA |
| BK#2 - Diamond Bank | BK#4 - Fidelity Bank | BK#6 - GTBank | BK#8 - Zenith Bank |

**Source: Author's Computation (2017)**

Table 4.2 shows the demographic characteristics of respondents in the eight deposit money banks used for this research study. The method of presentation of the results show on the first row the absolute numbers of respondents in each demography sub category for individual bank, and the total for the eight banks. A percentage of the total sub-category relative the total for all sub-categories is thereafter determined. The second row shows distribution of each sub-category expressed in percentage relative to the total for all sub-categories within individual bank. For instance, using 'Years Spent', the first row shows BK#5 having 15 respondents under the 'less than 3 years' sub category, while all banks have 62 respondents under the sub-category (representing 10.82%). Within BK#5, the 'less than 3 years' sub-category represents 14.6% of the total respondents (103). A further interpretation of the table is shown below:

**Gender**:

A total of 347 male respondents (representing 60.56%) and 226 female respondents (representing 39.44%) were sampled in the study. Gender distribution analysis of each bank within the total sample in percentage terms, shows BK#5 had the highest male representation at 18.73%, while BK#1 and BK#4 had the least representation at 7.49% each. In the female category, BK#7 had the highest proportion at 17.26%, with BK#1 with the least at 4.87%. Despite the disparity between the number of

male and female respondents, the distribution shows that there was a good representation of the male and female gender (approximately 60% to 40%), thus ensuring that the data obtained reflects strong and unbiased views of diverse genders.

**Age**:

The age distribution of respondents was categorised based on the following clusters: 18-24, 25-34, 35-44, 45-54, and above 54 years. The frequency and percentages of age category are 14 (2.44%), 244 (42.58%), 258 (45.03%), and 57 (9.95%) respectively, with none of the respondents being above 54 years old. The age distribution of the respondents shows that all the respondents were adults, whose ages ranged from 18 to 54 years, thus they were deemed capable of expressing their preferences, choices and making reasonable decisions. In addition, the bulk of respondents (87.6%) came from the 25-35, and 35-45 group (i.e. 42.6% and 45% respectively), signifying a very active population of employees with knowledge and experience of issues about the banking industry.

**Marital Status**:

The marital status distribution of respondents was categorised into: single, married, divorced, and separated. The frequency and percentages are 140 (24.43%), 428 (74.69%), 4 (0.70%), and 1 (0.17%) respectively. In all the banks under study, the married category had more representation than any other marital status category. The distribution shows a representation of all categories, albeit a preponderance of married respondents (74.69%) to other clusters, which is not unusual considering the industry under study.

**Highest Education**:

The highest education status distribution of respondents was categorised into: HND/BSc, MSc/MBA/M.Ed, Doctorate, and Others. The frequency and percentages are 278 (48.52%), 277 (48.34%), 11 (1.92%), and 7 (1.22%) respectively. In all the banks under study, there was representation across all levels, except BK#4, and BK#1,2,5 in the "Doctorate and "Others" category respectively. The distribution shows approximately equal representation at first and second-degree level. The results reflect a good representation of educational level suitable for understanding the purpose of the study as well as the questions contained in the questionnaire.

**Job Function**:

The Job Function distribution of respondents was categorised along the following broad clusters: Business development (to include roles such as marketing, treasury among others), operations and technology (covers all domestic and international operations services, as well as information

technology support groups), risk management (covers internal audits/controls, compliance, credit and other risks management among others), corporate development (covers functions such as strategic planning, financial control, corporate affairs, human capital management, administration, business transformation groups among others), and the Others (to specify non-assigned functions). In all the banks under study, three (BK#3, #5, and #6) had more respondents in other functions than Risk Management. The distribution covers the major categories of functions in the banking industry. While Risk Management function plays statutorily-assigned role in corporate governance, the representation of other key functions provides a balance for diversity of opinions.

**Job Position**:

The job position status was categorised into Trainee (representing new intakes and up to the third level entry in the bank), banking officers (officers and senior banking officers), management (assistant managers, deputy managers, managers, senior managers and principal managers), senior management (assistant general managers, deputy general managers, and general managers), and executive management (executive directors). A total of 229 employees occupy management level and above (representing 40%), while the remaining respondents totalling 344 (representing 60%) occupy levels below the management cadre. This is consistent with the pyramidal structure in organisations. The job position data structure is further enhanced by the years of experience employees had stayed on the job in their current place of employment.

**Years spent on the job**:

A total of 360 employees had spent 6 years and above on the job (representing 63%), while the remaining respondents totalling 213 (representing 37%) had spent less than 6 years in their current place of employment. It should be noted that employees move across the industry fairly regularly and therefore the years spent by each respondent in their current employment is at least equal to, or less than total years spent in the banking industry. The data structure therefore complements the job position status analysis (in Table 4.2).

## 4.3 Descriptive Statistics on Classification of Research Variables

This study adopted descriptive statistics in order to show the level of mean scores and standard deviation. The mean scores were obtained based on a five (5) point Likert scale. This ranges from "strongly agree" represented with 5 points to "strongly disagree" represented with 1 point, or alternatively as depicted in the study section on financial performance, "excellent" represented by 5

points to "very poor" represented by 1. The analysis showed the degree of responses by compiling the mean scores of each item using Statistical Package for Social Sciences (SPSS/PASW) software, V 23.

### 4.3.1 Descriptive Statistics of responses on Independent Variables

#### 4.3.1.1 Descriptive Statistics of responses on Internal Governance Controls

The following tables show the descriptive statistics of responses from the eight banks included in this research study on Internal Governance Controls. Internal governance controls was further broken into three (3) categories: board structure, management structure and ownership structure. The measurement items (extracted from the questionnaire) for each category are disclosed in tables below, while the full questionnaire is in appendix "A". The relevance of showing the descriptive statistics is to identify the degree of importance that respondents place on each of the corporate governance mechanism variables. Analysis of the mean score and standard deviation for each measured item on individual bank basis, as well as the total sample Likert-response rate in frequency and percentage is shown in the tables below:

**Table 4.3a: Descriptive Statistics Internal Governance Controls – Board Structure**

| | **BANK CODE** | | | | | | | | **LIKERT RESPONSE (freqand%)** | | | | | **STAT** | |
|---|---|---|---|---|---|---|---|---|---|---|---|---|---|---|---|
| **Board of Directors** | **#1** | **#2** | **#3** | **#4** | **#5** | **#6** | **#7** | **#8** | **1** | **2** | **3** | **4** | **5** | **M** | **SD** |
| Board size | 3.60 | 3.38 | 3.29 | 3.65 | 3.04 | 3.12 | 3.17 | 3.58 | 11 | 110 | 207 | 183 | 62 | 3.31 | 0.96 |
| | 1.07 | 1.00 | 0.98 | 0.72 | 0.73 | 1.13 | 1.14 | 0.71 | *1.9* | *19.2* | *36.1* | *31.9* | *10.8* | | |
| Board composition | 3.84 | 3.88 | 3.57 | 3.93 | 3.57 | 3.56 | 3.89 | 3.84 | 1 | 42 | 152 | 282 | 96 | 3.75 | 0.82 |
| | 0.80 | 0.84 | 0.78 | 0.70 | 0.75 | 0.93 | 0.85 | 0.81 | *0.2* | *7.3* | *26.5* | *49.2* | *16.8* | | |
| Board independence | 4.24 | 4.13 | 3.73 | 4.09 | 4.12 | 3.89 | 4.17 | 4.06 | | 10 | 84 | 342 | 137 | 4.06 | 0.67 |
| | 0.64 | 0.68 | 0.75 | 0.53 | 0.45 | 0.70 | 0.70 | 0.78 | | *1.7* | *14.7* | *59.7* | *23.9* | | |
| Board meetings | 4.08 | 3.69 | 3.52 | 4.07 | 3.87 | 3.35 | 3.88 | 3.68 | 2 | 47 | 143 | 281 | 100 | 3.75 | 0.85 |
| | 0.68 | 0.80 | 0.74 | 0.63 | 0.80 | 0.87 | 0.98 | 0.86 | *0.3* | *8.2* | *25.0* | *49.0* | *17.5* | | |
| Board committees | 4.22 | 4.05 | 4.05 | 4.16 | 3.99 | 3.77 | 4.27 | 4.14 | 1 | 4 | 64 | 384 | 120 | 4.08 | 0.60 |
| | 0.53 | 0.63 | 0.64 | 0.49 | 0.48 | 0.80 | 0.60 | 0.47 | *0.2* | *0.7* | *11.2* | *67.0* | *20.9* | | |
| Diversity | 4.30 | 4.22 | 4.08 | 4.21 | 3.96 | 3.99 | 4.34 | 4.12 | | 4 | 84 | 312 | 173 | 4.14 | 0.68 |
| | 0.57 | 0.66 | 0.78 | 0.47 | 0.67 | 0.64 | 0.71 | 0.66 | | *0.7* | *14.7* | *54.5* | *30.2* | | |
| Strategy | 4.46 | 4.38 | 4.21 | 4.21 | 4.17 | 4.18 | 4.52 | 4.42 | | 2 | 49 | 284 | 238 | 4.32 | 0.64 |
| | 0.56 | 0.59 | 0.73 | 0.52 | 0.66 | 0.70 | 0.63 | 0.59 | | *0.3* | *8.6* | *49.6* | *41.5* | | |
| **MEAN** | **4.10** | **3.96** | **3.78** | **4.05** | **3.82** | **3.69** | **4.03** | **3.98** | | | | | | **3.92** | **0.47** |
| **STD DEV** | **0.43** | **0.44** | **0.53** | **0.32** | **0.41** | **0.49** | **0.48** | **0.46** | | | | | | | |

**Source: Author's Computation (2017)**

Table 4.3 (a) above shows the descriptive statistics of responses on board structure as an internal governance control mechanism. From responses obtained, the most agreeable statement (91%) is the

statement that "The Board plays a key role in setting the bank's strategy" (M=4.32, SD=0.64). This is followed (88%) by "Board committees have strong effect in shaping the bank's performance" (M=4.08, SD=0.60). About a fifth of respondents (21%) mostly disagree with the statement "The Board size is adequate to positively influence the bank's performance" (M=3.31, SD=0.96). Across the banks, there is a general positive disposition towards the importance of the board of directors as a governance mechanism. As shown above, BK#1 ranks highest with a mean score of 4.10 (SD=0.43), while BK#6 ranks the least with a mean score of 3.69 (SD=0.49). In specific terms, they rank the importance of the board structure as: size, diversity, committee, and independence in descending order. Overall, the findings indicate that the respondents agreed with all items, with an overall mean of 3.92 (SD=0.47). Mean scores for the measured items ranged between 3.31 and 4.32, suggesting that majority of the participants agreed, albeit to varying degrees.

**Table 4.3b: Descriptive Statistics Internal Governance Controls – Management Structure**

| | **BANK CODE** | | | | | | | | **LIKERT RESPONSE (freqand%)** | | | | | **STAT** | |
|---|---|---|---|---|---|---|---|---|---|---|---|---|---|---|---|
| **Management** | **#1** | **#2** | **#3** | **#4** | **#5** | **#6** | **#7** | **#8** | **1** | **2** | **3** | **4** | **5** | **M** | **SD** |
| Internal Promotion | 3.92 | 3.64 | 3.27 | 4.14 | 3.73 | 3.61 | 3.84 | 3.71 | 4 | 43 | 173 | 247 | 106 | 3.71 | 0.88 |
| | 0.98 | 0.99 | 0.71 | 0.60 | 0.73 | 0.99 | 0.96 | 0.78 | *0.7* | *7.5* | *30.2* | *43.1* | *18.5* | | |
| Tenure | 3.51 | 3.34 | 3.03 | 3.88 | 3.12 | 3.18 | 3.31 | 3.49 | 4 | 89 | 253 | 175 | 52 | 3.32 | 0.87 |
| | 0.99 | 0.94 | 0.68 | 0.63 | 0.63 | 0.84 | 0.93 | 1.00 | *0.7* | *15.5* | *44.2* | *30.5* | *9.1* | | |
| Remuneration | 4.03 | 3.70 | 3.39 | 3.91 | 3.47 | 3.38 | 3.61 | 3.60 | 2 | 39 | 222 | 237 | 73 | 3.59 | 0.81 |
| | 0.87 | 0.69 | 0.82 | 0.57 | 0.68 | 0.82 | 0.90 | 0.89 | *0.3* | *6.8* | *38.7* | *41.4* | *12.7* | | |
| Diversity | 3.81 | 3.75 | 3.63 | 3.95 | 3.33 | 3.47 | 3.71 | 3.73 | 12 | 58 | 137 | 284 | 82 | 3.64 | 0.92 |
| | 0.85 | 0.88 | 0.98 | 0.62 | 0.83 | 0.90 | 0.94 | 1.07 | *2.1* | *10.1* | *23.9* | *49.6* | *14.3* | | |
| CEO's reputation | 4.27 | 4.23 | 3.84 | 4.23 | 4.09 | 3.68 | 4.15 | 4.06 | 2 | 19 | 85 | 302 | 165 | 4.06 | 0.77 |
| | 0.56 | 0.58 | 0.98 | 0.53 | 0.60 | 0.83 | 0.96 | 0.73 | *0.3* | *3.3* | *14.8* | *52.7* | *28.8* | | |
| Incentive-based compensation | 3.78 | 3.56 | 3.45 | 4.09 | 3.39 | 3.52 | 3.62 | 3.71 | 3 | 45 | 211 | 234 | 80 | 3.60 | 0.84 |
| | 0.75 | 0.68 | 0.84 | 0.61 | 0.58 | 0.95 | 1.08 | 0.87 | *0.5* | *7.9* | *36.8* | *40.8* | *14.0* | | |
| **MEAN** | **3.89** | **3.70** | **3.44** | **4.03** | **3.52** | **3.47** | **3.71** | **3.72** | | | | | | **3.65** | **0.58** |
| **STD DEV** | **0.48** | **0.47** | **0.55** | **0.42** | **0.33** | **0.58** | **0.78** | **0.65** | | | | | | | |

**Source: Author's Computation (2017)**

Table 4.3b above shows the descriptive statistics of responses on the role of management structure as a governance mechanism. From responses obtained, the most agreeable statement (82%) is the statement that "The CEO's reputation in the society has impact on performance" (M=4.06, SD=0.77). This is followed (88%) by "CEO's diversity (age, gender, ethnicity) has significant impact on performance" (M=3.64, SD=0.92). About a sixth of respondents (16%) mostly disagree with the

statement “A longer tenure in office by the CEO increases the likelihood of improved bank performance” (M=3.32, SD=0.87). Across the banks, there is a general positive disposition towards the importance of management as a governance mechanism. It is however relatively weaker than the respondents’ perception of the board structure, as only about half expressed strong opinion in favour of management structure. As shown above, BK#4 ranks highest with a mean score of 4.03 (SD=0.42), while BK#3 ranks the least with a mean score of 3.44 (SD=0.55). In specific terms, respondents rank the importance of the management as: reputation, diversity, internal promotion, and incentive-based compensation in descending order. Overall, the findings indicate that the respondents agreed with all items, with an overall mean of 3.65 (SD=0.58). All mean scores for the items ranged between 3.32 and 4.06, therefore, the majority of participants agreed, albeit to varying degrees.

**Table 4.3c: Descriptive Statistics Internal Governance Controls – Ownership Structure**

| | BANK CODE | | | | | | | | LIKERT RESPONSE (freqand%) | | | | | STAT | |
|---|---|---|---|---|---|---|---|---|---|---|---|---|---|---|---|
| **Ownership** | **#1** | **#2** | **#3** | **#4** | **#5** | **#6** | **#7** | **#8** | **1** | **2** | **3** | **4** | **5** | **M** | **SD** |
| Institutional investors | 3.95 | 3.90 | 3.87 | 4.07 | 4.12 | 3.74 | 4.11 | 4.24 | 1 | 9 | 98 | 335 | 130 | 4.02 | 0.69 |
| | 0.85 | 0.62 | 0.74 | 0.51 | 0.70 | 0.79 | 0.62 | 0.61 | *0.2* | *1.6* | *17.1* | *58.5* | *22.7* | | |
| Insider ownership | 4.05 | 3.78 | 3.55 | 4.12 | 3.63 | 3.58 | 3.92 | 3.98 | 1 | 14 | 171 | 299 | 88 | 3.80 | 0.73 |
| | 0.66 | 0.70 | 0.80 | 0.66 | 0.64 | 0.77 | 0.72 | 0.67 | *0.2* | *2.4* | *29.8* | *52.2* | *15.4* | | |
| Diffused ownership | 2.97 | 3.47 | 3.37 | 3.88 | 3.44 | 3.39 | 3.33 | 3.51 | 9 | 77 | 208 | 220 | 59 | 3.42 | 0.90 |
| | 1.01 | 0.79 | 0.98 | 0.66 | 0.76 | 0.88 | 1.17 | 0.70 | *1.6* | *13.4* | *36.3* | *38.4* | *10.3* | | |
| Concentrated ownership | 3.87 | 3.64 | 3.57 | 3.93 | 3.72 | 3.61 | 3.82 | 3.74 | 4 | 24 | 180 | 283 | 82 | 3.72 | 0.78 |
| | 0.71 | 0.63 | 0.74 | 0.55 | 0.76 | 0.74 | 1.00 | 0.82 | *0.7* | *4.2* | *31.4* | *49.4* | *14.3* | | |
| Employees Share Own. | 4.00 | 3.52 | 3.71 | 3.88 | 3.58 | 3.64 | 3.82 | 3.52 | 5 | 34 | 174 | 289 | 71 | 3.68 | 0.80 |
| | 0.94 | 0.81 | 0.73 | 0.63 | 0.57 | 0.92 | 0.80 | 0.92 | *0.9* | *5.9* | *30.4* | *50.4* | *12.4* | | |
| **MEAN** | **3.77** | **3.66** | **3.61** | **3.98** | **3.70** | **3.59** | **3.80** | **3.80** | | | | | | **3.73** | **0.51** |
| **STD DEV** | **0.54** | **0.43** | **0.61** | **0.38** | **0.33** | **0.60** | **0.62** | **0.42** | | | | | | | |

**Source: Author’s Computation (2017)**

Table 4.3c above shows the descriptive statistics of responses on the role of ownership structure as a governance mechanism. From responses obtained, the most agreeable statement (81%) is the statement that “Institutional investors provide effective monitoring on the Board” (M=4.02, SD=0.69). This is followed (68%) by “Directors’ share ownership is most helpful to improve stakeholders’ interests” (M=3.80, SD=0.73). About a sixth of respondents (15%) mostly disagree with the statement “Diffused ownership (i.e. many with few shares) encourages little or no monitoring of management” (M=3.42, SD=0.90). Across the banks, there is a general positive disposition towards the importance of ownership as an internal governance control mechanism. It is however relatively weaker than the

respondents' perception of the board structure, as only about two-thirds expressed strong opinion in favour of ownership. As shown above, BK#4 ranks highest with a mean score of 3.98 (SD=0.38), while BK#6 ranks the least with a mean score of 3.59 (SD=0.60). In specific terms, they rank the importance of the ownership as: institutional investors, directors' share ownership, and concentrated ownership in descending order. Overall, the findings indicate that the respondents agreed with all items, with an overall mean of 3.73 (SD=0.51). All mean scores for the items ranged between 3.42 and 4.02, thus the majority of participants agreed, albeit to varying degrees.

### 4.3.1.2 Descriptive Statistics of Responses on Regulation

The following tables show the descriptive statistics of responses from the eight banks included in this research study on regulation variables. The relevance of showing the descriptive statistics is to identify the degree of importance that respondents place on each of the regulation measures. The measure test is indicated in the table below, while the full questionnaire is in appendix "A". Analysis of the mean score and standard deviation for each measured item on individual bank basis, as well as the total sample Likert-response rate in frequency and percentage is shown in the tables below:

**Table: 4.4: Descriptive Statistics Regulation**

| | BANK CODE | | | | | | | | LIKERT RESPONSE (freqand%) | | | | | STAT | |
|---|---|---|---|---|---|---|---|---|---|---|---|---|---|---|---|
| | **#1** | **#2** | **#3** | **#4** | **#5** | **#6** | **#7** | **#8** | **1** | **2** | **3** | **4** | **5** | **M** | **SD** |
| Regulators' ethical values | 4.22 | 4.09 | 4.00 | 4.30 | 3.88 | 4.33 | 4.35 | 4.14 | 1 | 7 | 82 | 299 | 184 | 4.15 | 0.71 |
| | 0.58 | 0.78 | 0.54 | 0.51 | 0.68 | 0.71 | 0.81 | 0.71 | *0.2* | *1.2* | *14.3* | *52.2* | *32.1* | | |
| Disclosure | 4.22 | 4.27 | 3.94 | 4.42 | 4.23 | 4.47 | 4.14 | 4.35 | | 20 | 30 | 311 | 212 | 4.25 | 0.71 |
| | 0.79 | 0.79 | 0.65 | 0.55 | 0.88 | 0.56 | 0.65 | 0.55 | | *3.5* | *5.2* | *54.3* | *37.0* | | |
| Activity restrictions | 3.62 | 3.83 | 3.65 | 4.12 | 3.56 | 3.73 | 3.74 | 3.87 | 6 | 57 | 131 | 260 | 119 | 3.75 | 0.93 |
| | 1.14 | 0.85 | 0.93 | 0.63 | 1.03 | 0.99 | 0.93 | 0.83 | *1.0* | *9.9* | *22.9* | *45.4* | *20.8* | | |
| Capital requirement on efficiency. | 4.32 | 4.10 | 3.97 | 4.26 | 3.96 | 4.14 | 3.55 | 4.27 | 4 | 24 | 83 | 307 | 155 | 4.02 | 0.80 |
| | 0.82 | 0.64 | 0.81 | 0.58 | 0.48 | 0.76 | 1.14 | 0.64 | *0.7* | *4.2* | *14.5* | *53.6* | *27.1* | | |
| Capital requirement on risk appetite. | 3.81 | 3.81 | 3.60 | 3.98 | 3.77 | 3.71 | 3.58 | 3.95 | 1 | 44 | 147 | 280 | 101 | 3.76 | 0.84 |
| | 0.78 | 0.80 | 0.90 | 0.60 | 0.81 | 0.92 | 0.97 | 0.71 | *0.2* | *7.7* | *25.7* | *48.9* | *17.6* | | |
| Deposit Insurance Scheme | 3.51 | 3.56 | 3.60 | 3.93 | 3.31 | 3.62 | 3.94 | 3.66 | 5 | 92 | 103 | 282 | 91 | 3.63 | 0.96 |
| | 1.04 | 0.97 | 0.88 | 0.86 | 0.99 | 0.96 | 0.94 | 0.91 | *0.9* | *16.1* | *18.0* | *49.2* | *15.9* | | |
| CBN's Disciplining power | 4.46 | 4.38 | 4.29 | 4.37 | 4.33 | 4.46 | 4.54 | 4.21 | 1 | 2 | 23 | 301 | 246 | 4.38 | 0.60 |
| | 0.56 | 0.56 | 0.64 | 0.54 | 0.47 | 0.68 | 0.61 | 0.66 | *0.2* | *0.3* | *4.0* | *52.5* | *42.9* | | |
| Entry/Exit restrictions | 4.22 | 4.03 | 3.92 | 4.23 | 3.67 | 3.99 | 4.24 | 4.14 | 7 | 19 | 85 | 302 | 160 | 4.03 | 0.82 |
| | 0.75 | 0.84 | 0.84 | 0.61 | 0.79 | 0.79 | 0.94 | 0.66 | *1.2* | *3.3* | *14.8* | *52.7* | *27.9* | | |
| Pricing of banks' products | 4.11 | 4.22 | 3.92 | 4.21 | 4.01 | 4.24 | 4.44 | 4.17 | 2 | 10 | 59 | 317 | 185 | 4.17 | 0.71 |
| | 0.81 | 0.74 | 0.78 | 0.56 | 0.51 | 0.70 | 0.70 | 0.75 | *0.3* | *1.7* | *10.3* | *55.3* | *32.3* | | |

| | #1 | #2 | #3 | #4 | #5 | #6 | #7 | #8 | 1 | 2 | 3 | 4 | 5 | M | SD |
|---|---|---|---|---|---|---|---|---|---|---|---|---|---|---|---|
| Regulators' skills level | 3.95 | 3.81 | 3.58 | 4.07 | 3.65 | 3.80 | 3.61 | 3.95 | 4 | 19 | 156 | 320 | 74 | 3.77 | 0.74 |
| | 0.62 | 0.71 | 0.67 | 0.46 | 0.57 | 0.79 | 1.01 | 0.63 | *0.7* | *3.3* | *27.2* | *55.8* | *12.9* | | |
| CBN's regulations against bank failures. | 4.24 | 3.95 | 3.71 | 4.19 | 4.26 | 3.99 | 4.40 | 4.08 | 3 | 7 | 74 | 324 | 165 | 4.12 | 0.71 |
| | 0.55 | 0.69 | 0.61 | 0.45 | 0.48 | 0.77 | 0.88 | 0.73 | *0.5* | *1.2* | *12.9* | *56.5* | *28.8* | | |
| **MEAN** | **4.06** | **4.00** | **3.83** | **4.19** | **3.88** | **4.04** | **4.05** | **4.07** | | | | | | **4.00** | **0.40** |
| **STD DEV** | **0.42** | **0.38** | **0.41** | **0.34** | **0.22** | **0.41** | **0.50** | **0.38** | | | | | | | |

**Source: Author's Computation (2017)**

Table 4.4 above shows the descriptive statistics of responses on the role of regulation in corporate governance. From responses obtained, the most agreeable statement (95%) is the statement that "The supervisory and regulatory power of CBN to discipline bank management, owners, and auditors is vital to the banking system" (M=4.38, SD=0.60). This is followed (91%) by "Disclosure of bank's performance as published in the annual report adequately provides stakeholders with transparent information for their decision-making" (M=4.25, SD=0.71). About a sixth of respondents (17%) mostly disagree with the statement "The Deposit Insurance Scheme of NDIC to protect depositors' funds has minimal impact on banks' risk-taking" (M=3.63, SD=0.96). Across the banks, there is a general positive disposition towards the importance of regulation in corporate governance. Respondents overwhelmingly gave a strong favourable opinion on seven of the eleven measured items, while the remaining four items had approximately 67% agreement. As shown above, BK#4 ranks highest with a mean score of 4.19 (SD=0.34), while BK#3 ranks the least with a mean score of 3.83 (SD=0.41). In specific terms, they rank the importance of regulation as: disciplining power of regulators, disclosure, regulations, and capital requirements in descending order. Overall, the findings indicate that the respondents agreed with all items, with an overall mean of 4.00 (SD=0.40). All mean scores for the items ranged between 3.63 and 4.38, thus the majority of participants agreed, albeit to varying degrees.

### 4.3.1.3 Descriptive Statistics of Responses on Ethical leadership

The following tables show the descriptive statistics of responses from the eight banks included in this research study on ethical leadership variables, which is measured from three dimensions - the CEO's ethical values, the organisation's ethical work program, and organisation's ethical culture. The relevance of showing the descriptive statistics is to identify the degree of importance that respondents place on each of the ethical leadership measures. The measure test is indicated in the table below, while the full questionnaire is in appendix "A". Analysis of the mean score and standard deviation for each

measured item on individual bank basis, as well as the total sample Likert-response rate in frequency and percentage is shown in the tables below:

**Table 4.5a: Descriptive Statistics Ethical leadership – CEO's Personal Ethics**

| | BANK CODE | | | | | | | | LIKERT RESPONSE (freqand%) | | | | | STAT | |
|---|---|---|---|---|---|---|---|---|---|---|---|---|---|---|---|
| **ELC (CEO's personal ethics)** | **#1** | **#2** | **#3** | **#4** | **#5** | **#6** | **#7** | **#8** | **1** | **2** | **3** | **4** | **5** | **M** | **SD** |
| Empathy | 3.92 | 3.74 | 3.82 | 4.32 | 4.03 | 3.55 | 3.98 | 3.31 | 8 | 34 | 132 | 283 | 116 | 3.81 | 0.87 |
| | 0.76 | 1.04 | 0.76 | 0.57 | 0.80 | 0.93 | 0.59 | 0.99 | *1.4* | *5.9* | *23.0* | *49.4* | *20.2* | | |
| Fairness | 3.95 | 3.68 | 3.40 | 4.26 | 3.77 | 3.73 | 3.30 | 3.34 | 2 | 66 | 169 | 250 | 86 | 3.61 | 0.89 |
| | 0.62 | 0.82 | 0.80 | 0.73 | 0.95 | 0.90 | 0.88 | 0.84 | *0.3* | *11.5* | *29.5* | *43.6* | *15.0* | | |
| Long-term focus | 4.11 | 3.77 | 3.60 | 3.88 | 4.11 | 4.17 | 3.84 | 3.65 | | 15 | 149 | 298 | 111 | 3.88 | 0.74 |
| | 0.61 | 0.76 | 0.76 | 0.66 | 0.83 | 0.71 | 0.68 | 0.59 | | *2.6* | *26.0* | *52.0* | *19.4* | | |
| Integrity | 4.19 | 3.77 | 3.81 | 4.28 | 3.72 | 4.12 | 3.75 | 3.25 | 17 | 19 | 120 | 329 | 88 | 3.79 | 0.85 |
| | 0.52 | 0.97 | 0.77 | 0.55 | 0.55 | 0.67 | 0.80 | 1.14 | *3.0* | *3.3* | *20.9* | *57.4* | *15.4* | | |
| Tone at the top | 4.27 | 3.84 | 3.66 | 4.37 | 3.50 | 4.15 | 3.80 | 4.05 | 4 | 16 | 124 | 326 | 103 | 3.89 | 0.75 |
| | 0.56 | 0.71 | 0.77 | 0.49 | 0.73 | 0.71 | 0.82 | 0.60 | *0.7* | *2.8* | *21.6* | *56.9* | *18.0* | | |
| **MEAN** | **4.09** | **3.76** | **3.66** | **4.22** | **3.82** | **3.94** | **3.73** | **3.52** | | | | | | **3.80** | **0.63** |
| **STD DEV** | **0.42** | **0.65** | **0.56** | **0.39** | **0.63** | **0.63** | **0.62** | **0.67** | | | | | | | |

**Source: Author's Computation (2017)**

Table 4.5a above shows the descriptive statistics of responses on the role of the CEO's ethical values in corporate governance. From responses obtained, the most agreeable statement (75%) is the statement that "The top management sets a good example in terms of ethical behaviour" (M=3.89, SD=0.75). This is followed (73%) by "The CEO can be trusted to do the things he/she says" (M=3.79, SD=0.85). About a tenth of respondents (12%) mostly disagree with the statement "The CEO rewards performance in a fair manner" (M=3.61, SD=0.89). Across the banks, there is a fairly positive disposition towards the importance of the CEO's personal ethical values in corporate governance. Respondents gave a moderately favourable opinion on four of the five measured items, while the remaining item had approximately 60% agreement. As shown above, BK#4 ranks highest with a mean score of 4.19 (SD=0.34), while BK#3 ranks the least with a mean score of 3.83 (SD=0.41). In specific terms, they rank the importance of the CEO's personal ethics as follows: tone at the top, integrity, and long-term focus in descending order. Overall, the findings indicate that the respondents agreed with all items, with an overall mean of 3.80 (SD=0.63). All mean scores for the items ranged between 3.61 and 3.89, thus the majority of participants agreed, albeit to varying degrees.

**Table 4.5b: Descriptive Statistics Ethical leadership – Ethical Programs**

| | BANK CODE | | | | | | | | LIKERT RESPONSE (freqand%) | | | | | STAT | |
|---|---|---|---|---|---|---|---|---|---|---|---|---|---|---|---|
| **Ethical Programmes** | **#1** | **#2** | **#3** | **#4** | **#5** | **#6** | **#7** | **#8** | **1** | **2** | **3** | **4** | **5** | **M** | **SD** |
| Codes of ethics | 4.49 | 4.26 | 4.23 | 4.32 | 4.37 | 4.58 | 4.46 | 4.39 | 2 | 1 | 27 | 287 | 256 | 4.39 | 0.62 |
| | 0.51 | 0.52 | 0.64 | 0.52 | 0.64 | 0.53 | 0.66 | 0.73 | *0.3* | *0.2* | *4.7* | *50.1* | *44.7* | | |
| Ethical officer | 4.27 | 3.62 | 3.73 | 4.19 | 3.80 | 4.14 | 3.90 | 4.05 | 3 | 30 | 117 | 283 | 140 | 3.92 | 0.84 |
| | 0.73 | 0.92 | 0.81 | 0.73 | 0.89 | 0.84 | 0.80 | 0.71 | *0.5* | *5.2* | *20.4* | *49.4* | *24.4* | | |
| Ethical training programmes | 4.46 | 4.00 | 4.03 | 4.28 | 4.06 | 4.26 | 3.95 | 4.26 | 1 | 20 | 44 | 350 | 158 | 4.12 | 0.70 |
| | 0.51 | 0.71 | 0.72 | 0.50 | 0.67 | 0.73 | 0.88 | 0.52 | *0.2* | *3.5* | *7.7* | *61.1* | *27.6* | | |
| **MEAN** | **4.41** | **3.96** | **3.99** | **4.26** | **4.07** | **4.32** | **4.10** | **4.23** | | | | | | **4.14** | **0.56** |
| **STD DEV** | **0.41** | **0.56** | **0.56** | **0.52** | **0.54** | **0.58** | **0.60** | **0.46** | | | | | | | |

**Source: Author's Computation (2017)**

Table 4.5b above shows the descriptive statistics of responses on the role of the organisation's ethical work programmes in corporate governance. From responses obtained, the most agreeable statement (95%) is the statement that "The bank has documented codes and practices of ethics known officially to all staff (M=4.39, SD=0.62). This is followed at (89%) by "My bank promotes ethical awareness through training programmes for staff" (M=4.12, SD=0.70). Only a minor representation of respondents (6%) mostly disagree with the statement "A designated ethical officer monitors and reports on staff unethical behaviours" (M=3.92, SD=0.84). Across the banks, there is an overwhelming positive disposition towards the importance of the organisation's ethical work programmes in corporate governance. Respondents gave strong favourable opinion on the three measured items. As shown above, BK#1 ranks highest with a mean score of 4.41 (SD=0.41), while BK#2 ranks the least with a mean score of 3.96 (SD=0.56). Overall, the findings indicate that the respondents agreed with all items, with an overall mean of 4.14 (SD=0.56). All mean scores for the items ranged between 3.92 and 4.39, thus the majority of participants agreed, albeit to varying degrees.

**Table 4.5c: Descriptive Statistics Ethical leadership –Ethical Culture**

| | BANK CODE | | | | | | | | LIKERT RESPONSE (freqand%) | | | | | STAT | |
|---|---|---|---|---|---|---|---|---|---|---|---|---|---|---|---|
| **Organisational Ethical Culture** | **#1** | **#2** | **#3** | **#4** | **#5** | **#6** | **#7** | **#8** | **1** | **2** | **3** | **4** | **5** | **M** | **SD** |
| Moral Principles | 3.78 | 3.74 | 3.74 | 4.16 | 4.01 | 3.74 | 4.03 | 3.42 | 17 | 35 | 121 | 257 | 143 | 3.83 | 0.97 |
| | 0.89 | 0.97 | 0.85 | 0.62 | 0.87 | 0.92 | 0.92 | 1.29 | *3.0* | *6.1* | *21.1* | *44.9* | *25.0* | | |
| Dealing with Conflict of interest | 3.92 | 3.68 | 3.48 | 3.93 | 3.88 | 3.41 | 3.36 | 3.78 | 24 | 52 | 92 | 334 | 71 | 3.66 | 0.95 |
| | 0.92 | 0.98 | 0.95 | 0.77 | 0.65 | 1.05 | 1.25 | 0.68 | *4.2* | *9.1* | *16.1* | *58.3* | *12.4* | | |
| Remedial fairness | 4.24 | 3.95 | 3.81 | 4.40 | 3.76 | 4.23 | 4.09 | 3.78 | 5 | 9 | 97 | 342 | 120 | 3.98 | 0.72 |
| | 0.60 | 0.72 | 0.72 | 0.50 | 0.62 | 0.63 | 0.70 | 0.88 | *0.9* | *1.6* | *16.9* | *59.7* | *20.9* | | |
| Reward | 4.03 | 3.53 | 3.32 | 4.26 | 3.60 | 3.77 | 3.54 | 3.75 | 7 | 54 | 155 | 262 | 95 | 3.67 | 0.90 |
| | 0.96 | 1.00 | 0.81 | 0.66 | 0.80 | 0.93 | 0.92 | 0.89 | *1.2* | *9.4* | *27.1* | *45.7* | *16.6* | | |
| Authorisation Procedure | 3.89 | 3.51 | 3.55 | 4.02 | 3.89 | 3.67 | 3.53 | 4.05 | 7 | 66 | 111 | 270 | 119 | 3.75 | 0.95 |
| | 0.97 | 0.97 | 0.86 | 0.71 | 0.83 | 1.16 | 1.09 | 0.77 | *1.2* | *11.5* | *19.4* | *47.1* | *20.8* | | |
| **MEAN** | **3.97** | **3.68** | **3.58** | **4.15** | **3.83** | **3.76** | **3.71** | **3.76** | | | | | | **3.78** | **0.50** |
| **STD DEV** | **0.58** | **0.52** | **0.47** | **0.34** | **0.40** | **0.60** | **0.51** | **0.46** | | | | | | | |

**Source: Author's Computation (2017)**

Table 4.5c above shows the descriptive statistics of responses on the role of the organisation's ethical culture in corporate governance. From responses obtained, the most agreeable statement (81%) is the statement that "Unethical conduct reported is disciplined fairly" (M=3.98, SD=0.72). This is followed at (70%) by "In my bank, employees are never asked to do things that conflict with their moral principles" (M=3.83, SD=0.97). About a seventh of respondents (13%) mostly disagree with the statement "The bank makes it clear to employees how to deal with conflicts of interests" (M=3.66, SD=0.95). Across the banks, there is a fairly positive disposition towards the importance of the organisation's ethical culture in corporate governance. Respondents gave a favourably strong opinion on one of the five measured items, while the remaining items had approximately 67% agreement. As shown above, BK#4 ranks highest with a mean score of 4.15 (SD=0.34), while BK#3 ranks the least with a mean score of 3.58 (SD=0.47). Overall, the findings indicate that the respondents agreed with all items, with an overall mean of 3.78 (SD=0.50). All mean scores for the items ranged between 3.66 and 3.98, thus the majority of participants agreed, albeit to varying degrees.

### 4.3.2 Descriptive Statistics of responses on Dependent Variables

#### 4.3.2.1 Descriptive Statistics of responses on Corporate Social Performance

The following tables show the descriptive statistics of responses from the eight banks included in this research study on corporate social performance variables, which is measured from the social, and environmental performance dimensions. Social performance is further dimensioned from the banks' performance towards employees, customers, and the community. The relevance of showing the descriptive statistics is to identify the degree of importance that respondents place on each of the social responsibility variables. The measure test is indicated in the table below, while the full questionnaire is in appendix "A". Analysis of the mean score and standard deviation for each measured item on individual bank basis, as well as the total sample Likert-response rate in frequency and percentage is shown in the tables below:

**Table 4.6a: Descriptive Statistics Corporate Social Performance –Social Performance (Employees)**

| | BANK CODE | | | | | | | | LIKERT RESPONSE (freqand%) | | | | | STAT | |
|---|---|---|---|---|---|---|---|---|---|---|---|---|---|---|---|
| **Employees** | **#1** | **#2** | **#3** | **#4** | **#5** | **#6** | **#7** | **#8** | **1** | **2** | **3** | **4** | **5** | **M** | **SD** |
| Training and development | 4.51 | 4.14 | 4.31 | 4.33 | 3.95 | 4.44 | 3.86 | 4.32 | 1 | 26 | 55 | 281 | 210 | 4.17 | 0.79 |
| | 0.51 | 0.82 | 0.69 | 0.61 | 0.85 | 0.75 | 0.99 | 0.52 | 0.2 | 4.5 | 9.6 | 49.0 | 36.6 | | |
| Remuneration | 4.05 | 3.84 | 3.36 | 3.95 | 3.47 | 4.00 | 3.56 | 3.68 | 12 | 58 | 114 | 301 | 88 | 3.69 | 0.92 |
| | 0.74 | 0.84 | 0.99 | 0.69 | 0.95 | 0.84 | 0.97 | 0.93 | 2.1 | 10.1 | 19.9 | 52.5 | 15.4 | | |
| Staff turnover | 3.54 | 3.16 | 2.92 | 3.77 | 3.18 | 3.64 | 2.73 | 3.69 | 24 | 120 | 164 | 210 | 55 | 3.27 | 1.03 |
| | 0.93 | 0.93 | 0.91 | 0.68 | 0.81 | 1.00 | 1.24 | 0.96 | 4.2 | 20.9 | 28.6 | 36.6 | 9.6 | | |
| Job satisfaction | 3.65 | 3.31 | 3.19 | 3.86 | 3.43 | 3.55 | 2.97 | 3.53 | 20 | 107 | 147 | 232 | 67 | 3.38 | 1.03 |
| | 0.89 | 0.96 | 1.02 | 0.80 | 0.97 | 1.01 | 1.14 | 1.02 | 3.5 | 18.7 | 25.7 | 40.5 | 11.7 | | |
| Career advancement | 3.81 | 3.34 | 3.52 | 3.44 | 3.34 | 3.73 | 3.01 | 3.51 | 25 | 101 | 136 | 237 | 74 | 3.41 | 1.06 |
| | 0.85 | 1.03 | 0.86 | 0.93 | 1.17 | 0.92 | 1.18 | 1.02 | 4.4 | 17.6 | 23.7 | 41.4 | 12.9 | | |
| **MEAN** | **3.98** | **3.68** | **3.59** | **3.94** | **3.57** | **3.95** | **3.42** | **3.82** | | | | | | **3.70** | **0.67** |
| **STD DEV** | **0.51** | **0.63** | **0.57** | **0.49** | **0.63** | **0.66** | **0.79** | **0.64** | | | | | | | |

**Source: Author's Computation (2017)**

Table 4.6a above shows the descriptive statistics of responses on the role of social performance towards employees in an organisation's sustainability. From responses obtained, the most agreeable statement (86%) is the statement that "Training and development is highly valued and promoted" (M=4.17, SD=0.79). This is followed at (73%) by "Employees are remunerated and rewarded fairly" (M=3.69, SD=0.92). A quarter of respondents (25%) mostly disagree with the statement "Staff turnover is minimised in my bank" (M=3.27, SD=1.03). Across the banks, there is a weak positive disposition

towards their organisation's performance as it relates to employees. Respondents gave a favourably strong opinion on one of the five measured items, while the remaining items had approximately 55% agreement. As shown above, BK#1 ranks highest with a mean score of 3.98 (SD=0.51), while BK#7 ranks the least with a mean score of 3.42 (SD=0.64). In specific terms, they rank the importance of their organisation's social performance towards employees as: training and development, fair remuneration and career advancement in descending order. Nevertheless, the findings indicate that the respondents agreed with all items, with an overall mean of 3.70 (SD=0.67). All mean scores for the items ranged between 3.27 and 4.17, thus the majority of participants agreed, albeit to varying degrees.

**Table 4.6b: Descriptive Statistics Corporate Social Performance – Social Performance (Customers)**

| | **BANK CODE** | | | | | | | | **LIKERT RESPONSE (freqand%)** | | | | | **STAT** | |
|---|---|---|---|---|---|---|---|---|---|---|---|---|---|---|---|
| **Customers** | **#1** | **#2** | **#3** | **#4** | **#5** | **#6** | **#7** | **#8** | **1** | **2** | **3** | **4** | **5** | **M** | **SD** |
| Consumer rights protection | 4.30 | 4.27 | 4.26 | 4.30 | 4.05 | 4.33 | 4.39 | 4.20 | 0 | 2 | 36 | 351 | 184 | 4.25 | 0.58 |
| | 0.52 | 0.64 | 0.60 | 0.60 | 0.38 | 0.66 | 0.53 | 0.65 | 0.0 | 0.3 | 6.3 | 61.3 | 32.1 | | |
| Service delivery | 4.11 | 4.26 | 3.98 | 4.19 | 3.97 | 4.36 | 3.90 | 4.22 | 1 | 10 | 59 | 360 | 143 | 4.11 | 0.65 |
| | 0.57 | 0.64 | 0.80 | 0.55 | 0.43 | 0.57 | 0.75 | 0.70 | 0.2 | 1.7 | 10.3 | 62.8 | 25.0 | | |
| Conducive environment | 4.19 | 4.36 | 4.29 | 4.19 | 4.16 | 4.41 | 4.16 | 4.15 | 0 | 4 | 20 | 388 | 161 | 4.23 | 0.54 |
| | 0.46 | 0.56 | 0.58 | 0.50 | 0.36 | 0.55 | 0.53 | 0.66 | 0.0 | 0.7 | 3.5 | 67.7 | 28.1 | | |
| Innovative products and services | 4.43 | 4.49 | 4.26 | 4.30 | 4.42 | 4.62 | 4.15 | 4.22 | 0 | 7 | 21 | 309 | 236 | 4.35 | 0.61 |
| | 0.56 | 0.60 | 0.68 | 0.56 | 0.50 | 0.58 | 0.56 | 0.73 | 0.0 | 1.2 | 3.7 | 53.9 | 41.2 | | |
| customers' word-of-mouth marketing | 3.68 | 4.12 | 3.77 | 4.07 | 3.70 | 4.18 | 3.57 | 3.91 | 1 | 11 | 144 | 332 | 85 | 3.85 | 0.69 |
| | 0.67 | 0.61 | 0.61 | 0.63 | 0.52 | 0.61 | 0.81 | 0.72 | 0.2 | 1.9 | 25.1 | 57.9 | 14.8 | | |
| **MEAN** | **4.10** | **4.31** | **4.08** | **4.19** | **4.06** | **4.39** | **3.95** | **4.13** | | | | | | **4.14** | **0.49** |
| **STD DEV** | **0.40** | **0.48** | **0.52** | **0.44** | **0.26** | **0.43** | **0.53** | **0.59** | | | | | | | |

**Source: Author's Computation (2017)**

Table 4.6b above shows the descriptive statistics of responses on the role of the social performance towards customers in attaining sustainability. From responses obtained, the most agreeable statement (96%) is the statement that "My bank provides a conducive environment for customers' transactions" (M=4.23, SD=0.54). This is closely followed (at 95%) by "My bank continuously introduces innovative products and services to meet customers' expectations" (M=4.35, SD=0.61). There was a negligible expression of disagreement with any of the statements. Across the banks, there is an overwhelming positive disposition towards the performance of their banks towards customers. Respondents gave a significantly favourable opinion on four of the five measured items, while the remaining item had approximately 73% agreement. As shown above, BK#6 ranks highest with a mean score of 4.39

(SD=0.43), while BK#7 ranks the least with a mean score of 3.95 (SD=0.53). In specific terms, they rank the importance of social performance towards customers as: conducive environment, innovative products and services, consumer rights' protection, and timely service delivery in descending order. Overall, the findings indicate that the respondents agreed with all items, with an overall mean of 4.14 (SD=0.49). All mean scores for the items ranged between 3.85 and 4.35, thus the majority of participants agreed, albeit to varying degrees.

**Table 4.6c: Descriptive Statistics Corporate Social Performance – Social Performance (Community)**

| | BANK CODE | | | | | | | | LIKERT RESPONSE (freqand%) | | | | | STAT | |
|---|---|---|---|---|---|---|---|---|---|---|---|---|---|---|---|
| **Community** | **#1** | **#2** | **#3** | **#4** | **#5** | **#6** | **#7** | **#8** | **1** | **2** | **3** | **4** | **5** | **M** | **SD** |
| Philanthropy | 4.60 | 4.14 | 3.86 | 4.16 | 4.37 | 4.09 | 3.96 | 3.74 | 0 | 9 | 101 | 295 | 168 | 4.09 | 0.73 |
| | 0.55 | 0.76 | 0.77 | 0.69 | 0.54 | 0.72 | 0.72 | 0.71 | 0.0 | 1.6 | 17.6 | 51.5 | 29.3 | | |
| Staff volunteerism | 4.68 | 3.91 | 3.66 | 4.07 | 4.06 | 4.49 | 3.59 | 3.59 | 3 | 13 | 128 | 304 | 125 | 3.93 | 0.76 |
| | 0.58 | 0.69 | 0.68 | 0.67 | 0.56 | 0.56 | 0.84 | 0.71 | 0.5 | 2.3 | 22.3 | 53.1 | 21.8 | | |
| Local community meetings | 4.11 | 3.68 | 3.65 | 3.95 | 3.47 | 3.71 | 3.81 | 3.53 | 0 | 23 | 191 | 300 | 59 | 3.69 | 0.71 |
| | 0.61 | 0.77 | 0.81 | 0.58 | 0.54 | 0.76 | 0.63 | 0.77 | 0.0 | 4.0 | 33.3 | 52.4 | 10.3 | | |
| Marginalised groups | 3.78 | 3.46 | 3.63 | 3.81 | 3.09 | 3.68 | 3.67 | 3.49 | 3 | 45 | 231 | 236 | 58 | 3.53 | 0.80 |
| | 0.71 | 0.77 | 0.81 | 0.66 | 0.63 | 0.75 | 0.73 | 1.01 | 0.5 | 7.9 | 40.3 | 41.2 | 10.1 | | |
| Bribery and corruption | 4.70 | 4.25 | 3.90 | 4.26 | 4.07 | 4.32 | 4.52 | 3.86 | 1 | 8 | 87 | 253 | 224 | 4.21 | 0.76 |
| | 0.52 | 0.57 | 0.67 | 0.66 | 0.89 | 0.81 | 0.52 | 0.85 | 0.2 | 1.4 | 15.2 | 44.2 | 39.1 | | |
| **MEAN** | **4.37** | **3.89** | **3.74** | **4.05** | **3.81** | **4.06** | **3.91** | **3.64** | | | | | | **3.89** | **0.51** |
| **STD DEV** | **0.38** | **0.51** | **0.49** | **0.47** | **0.38** | **0.40** | **0.48** | **0.61** | | | | | | | |

**Source: Author's Computation (2017)**

Table 4.6c above shows the descriptive statistics of responses on the role of social performance towards the community in an organisation's sustainability. From responses obtained, the most agreeable statement (83%) is the statement that "My bank has a policy statement on bribery and corruption" (M=4.21, SD=0.76). This is followed closely at (81%) by "My bank supports philanthropic activities financially on a regular basis" (M=4.09, SD=0.73). About a tenth of respondents (8%) mostly disagree with the statement "My bank executes initiatives for the marginalised groups in the society regularly" (M=3.53, SD=0.80). Across the banks, there is a moderately positive disposition towards their organisation's performance as it relates to the community. Respondents gave a favourably strong opinion on two of the five measured items, while the remaining items had approximately 63% agreement. As shown above, BK#1 ranks highest with a mean score of 4.37 (SD=0.38), while BK#8 ranks the least with a mean score of 3.64 (SD=0.61). In specific terms, they rank the importance of

their organisation's social performance towards the community as: anti-bribery, philanthropy, and employee volunteer service in descending order. Overall, the findings indicate that the respondents agreed with all items, with an overall mean of 3.89 (SD=0.51). All mean scores for the items ranged between 3.53 and 4.21, thus majority of participants agreed, albeit to varying degrees.

**Table 4.6d: Descriptive Statistics Corporate Social Performance –Environmental Performance**

| | BANK CODE | | | | | | | | LIKERT RESPONSE (freqand%) | | | | | STAT | |
|---|---|---|---|---|---|---|---|---|---|---|---|---|---|---|---|
| **Environmental** | **#1** | **#2** | **#3** | **#4** | **#5** | **#6** | **#7** | **#8** | **1** | **2** | **3** | **4** | **5** | **M** | **SD** |
| Resource consumption reduction | 4.14 | 4.35 | 4.27 | 4.40 | 3.81 | 4.02 | 3.78 | 4.21 | 2 | 22 | 95 | 266 | 188 | 4.08 | 0.82 |
| | 0.67 | 0.72 | 0.83 | 0.62 | 0.86 | 0.85 | 0.92 | 0.64 | *0.3* | *3.8* | *16.6* | *46.4* | *32.8* | | |
| Environmental impact assessment | 4.22 | 4.07 | 4.11 | 4.23 | 3.90 | 3.88 | 3.66 | 4.26 | 2 | 14 | 119 | 285 | 153 | 4.00 | 0.78 |
| | 0.58 | 0.86 | 0.85 | 0.53 | 0.57 | 0.83 | 0.93 | 0.62 | *0.3* | *2.4* | *20.8* | *49.7* | *26.7* | | |
| Social responsible investment | 4.19 | 4.09 | 3.90 | 4.37 | 3.92 | 3.44 | 3.65 | 3.89 | 1 | 15 | 142 | 305 | 110 | 3.89 | 0.74 |
| | 0.74 | 0.67 | 0.72 | 0.54 | 0.39 | 0.84 | 0.88 | 0.71 | *0.2* | *2.6* | *24.8* | *53.2* | *19.2* | | |
| Awareness and training | 4.32 | 3.82 | 4.11 | 4.21 | 3.54 | 3.68 | 3.77 | 3.98 | 1 | 26 | 133 | 304 | 109 | 3.86 | 0.78 |
| | 0.58 | 0.82 | 0.79 | 0.51 | 0.57 | 0.77 | 0.90 | 0.76 | *0.2* | *4.5* | *23.2* | *53.1* | *19.0* | | |
| Pollution prevention | 3.68 | 3.57 | 3.84 | 3.77 | 2.90 | 3.36 | 3.63 | 3.99 | 7 | 47 | 215 | 232 | 72 | 3.55 | 0.86 |
| | 0.88 | 0.92 | 0.77 | 0.68 | 0.72 | 0.84 | 0.88 | 0.57 | *1.2* | *8.2* | *37.5* | *40.5* | *12.6* | | |
| Waste management | 3.84 | 3.60 | 4.00 | 4.07 | 3.24 | 3.56 | 3.78 | 3.92 | 7 | 33 | 172 | 272 | 89 | 3.70 | 0.84 |
| | 0.90 | 0.89 | 0.72 | 0.55 | 0.82 | 0.84 | 0.91 | 0.64 | *1.2* | *5.8* | *30.0* | *47.5* | *15.5* | | |
| New environmental technologies | 3.95 | 4.05 | 3.77 | 4.19 | 3.76 | 3.73 | 4.00 | 4.18 | | 17 | 118 | 318 | 120 | 3.94 | 0.73 |
| | 0.94 | 0.72 | 0.71 | 0.39 | 0.57 | 0.69 | 0.94 | 0.56 | | *3.0* | *20.6* | *55.5* | *20.9* | | |
| **MEAN** | **4.05** | **3.94** | **4.00** | **4.18** | **3.58** | **3.67** | **3.75** | **4.06** | | | | | | **3.86** | **0.58** |
| **STD DEV** | **0.56** | **0.55** | **0.54** | **0.31** | **0.44** | **0.56** | **0.75** | **0.41** | | | | | | | |

**Source: Author's Computation (2017)**

Table 4.6d above shows the descriptive statistics of responses on the role of environmental performance in an organisation's sustainability. From responses obtained, the most agreeable statement (79%) is the statement that "My bank continuously introduces initiatives to reduce resource consumption (water, energy, paper)" (M=4.08, SD=0.82). This is followed closely and jointly at (76%) by "my bank carries out environmental impact assessment on each credit application" (M=4.00, SD=0.78), and "my bank embraces new environmental technologies and processes" (M=3.94, SD=0.73). About a tenth of respondents (9%) mostly disagree with the statement "My bank invests in pollution prevention system" (M=3.55, SD=0.86). Across the banks, there is a moderately positive disposition towards their organisation's performance as it relates to the environment. Respondents' opinions were quite measured on five of the seven tested items, while the remaining items had approximately 58%

agreement. As shown above, BK#4 ranks highest with a mean score of 4.18 (SD=0.31), while BK#5 ranks the least with a mean score of 3.58 (SD=0.44). In specific terms, they rank the importance of their organisation's environmental performance as: resource efficiency, environmental impact assessment, new environmental technologies and processes, and awareness and training program in descending order. Overall, the findings indicate that the respondents agreed with all items, with an overall mean of 3.86 (SD=0.58). All mean scores for the items ranged between 3.55 and 4.08, thus the majority of participants agreed, albeit to varying degrees.

#### 4.3.2.2 Descriptive Statistics of responses on Financial Innovation

The following table shows the descriptive statistics of responses from the eight banks included in this research study on financial innovation variables, which is measured by four (4) items – products and services, Information Communication Technology (ICT), operating systems and processes, and delivery channels. Analysis of the mean score and standard deviation for each measured item on individual bank basis, as well as the total sample Likert-response rate in frequency and percentage is shown in the tables below:

**Table 4.7: Descriptive Statistics Financial Innovation**

| | **BANK CODE** | | | | | | | | **LIKERT RESPONSE (freqand%)** | | | | | **STAT** | |
|---|---|---|---|---|---|---|---|---|---|---|---|---|---|---|---|
| **Financial Innovation** | **#1** | **#2** | **#3** | **#4** | **#5** | **#6** | **#7** | **#8** | **1** | **2** | **3** | **4** | **5** | **M** | **SD** |
| Products and services | 4.27 | 4.20 | 4.13 | 4.23 | 4.24 | 4.29 | 4.45 | 4.13 | | 17 | 33 | 313 | 210 | 4.25 | 0.69 |
| | 0.56 | 0.74 | 0.69 | 0.53 | 0.47 | 0.70 | 0.76 | 0.87 | | *3.0* | *5.8* | *54.6* | *36.6* | | |
| ICT | 4.35 | 4.27 | 4.23 | 4.23 | 4.23 | 4.32 | 4.35 | 4.15 | 4 | 1 | 33 | 337 | 198 | 4.26 | 0.64 |
| | 0.54 | 0.58 | 0.58 | 0.57 | 0.49 | 0.64 | 0.89 | 0.59 | *0.7* | *0.2* | *5.8* | *58.8* | *34.6* | | |
| Operating systems and processes | 4.19 | 3.96 | 3.92 | 4.12 | 4.02 | 3.94 | 4.24 | 4.22 | 5 | 8 | 51 | 382 | 127 | 4.08 | 0.66 |
| | 0.66 | 0.79 | 0.58 | 0.50 | 0.40 | 0.76 | 0.75 | 0.68 | *0.9* | *1.4* | *8.9* | *66.7* | *22.2* | | |
| Delivery channels | 4.11 | 3.91 | 3.68 | 4.07 | 3.95 | 3.85 | 4.09 | 4.13 | 7 | 13 | 93 | 335 | 125 | 3.97 | 0.76 |
| | 0.74 | 0.81 | 0.67 | 0.59 | 0.69 | 0.81 | 0.84 | 0.75 | *1.2* | *2.3* | *16.2* | *58.5* | *21.8* | | |
| **MEAN** | **4.23** | **4.08** | **3.99** | **4.16** | **4.11** | **4.10** | **4.28** | **4.16** | | | | | | **4.14** | **0.51** |
| **STD DEV** | **0.54** | **0.51** | **0.43** | **0.43** | **0.38** | **0.57** | **0.63** | **0.52** | | | | | | | |

**Source: Author's Computation (2017)**

Table 4.7 above shows the descriptive statistics of responses on the role of financial innovation in an organisation's sustainability. From responses obtained, the most agreeable statement (79%) is the statement that "My bank outperforms peers in information communication technology (ICT)" (M=4.26, SD=0.69). This is followed closely at (91%) by "My bank develops innovative products and services"

(M=4.26, SD=0.64). Across the banks, there is a positively strong disposition towards their organisation's financial innovation. As shown in Table 4.6, BK#7 ranks highest with a mean score of 4.28 (SD=0.63), while BK#3 ranks the least with a mean score of 3.99 (SD=0.43). In specific terms, they rank the importance of their organisation's financial innovation practices as: ICT, products and services, operating systems and processes, and delivery channel in descending order. Overall, the findings indicate that the respondents agreed with all items, with an overall mean of 4.14 (SD=0.51). All mean scores for the items ranged between 3.97 and 4.26, thus the majority of participants agreed, albeit to varying degrees.

#### 4.3.2.3 Descriptive Statistics of responses on Corporate Reputation

The following table shows the descriptive statistics of responses from the eight banks included in this research study on corporate reputation variables, which is measured by three (3) items – product quality, financial soundness, and global competitiveness. Analysis of the mean score and standard deviation for each measured item on individual bank basis, as well as the total sample Likert-response rate in frequency and percentage is shown in the tables below:

**Table 4.8: Descriptive Statistics Corporate Reputation**

| | BANK CODE | | | | | | | | LIKERT RESPONSE (freqand%) | | | | | STAT | |
|---|---|---|---|---|---|---|---|---|---|---|---|---|---|---|---|
| **Corporate Reputation** | **#1** | **#2** | **#3** | **#4** | **#5** | **#6** | **#7** | **#8** | **1** | **2** | **3** | **4** | **5** | **M** | **SD** |
| Product quality | 4.32 | 4.13 | 4.15 | 4.12 | 4.11 | 4.24 | 4.26 | 4.02 | 5 | 9 | 58 | 319 | 182 | 4.16 | 0.73 |
| | 0.53 | 0.71 | 0.72 | 0.85 | 0.61 | 0.82 | 0.81 | 0.72 | *0.9* | *1.6* | *10.1* | *55.7* | *31.8* | | |
| Financial soundness | 4.27 | 4.04 | 4.08 | 4.30 | 4.11 | 4.24 | 4.24 | 3.95 | 4 | 8 | 69 | 317 | 175 | 4.14 | 0.73 |
| | 0.73 | 0.70 | 0.69 | 0.60 | 0.64 | 0.91 | 0.67 | 0.82 | *0.7* | *1.4* | *12.0* | *55.3* | *30.5* | | |
| Global competitiveness | 4.14 | 3.65 | 3.66 | 3.65 | 3.65 | 3.96 | 3.98 | 4.05 | 14 | 36 | 111 | 264 | 148 | 3.87 | 0.95 |
| | 0.79 | 0.89 | 0.83 | 1.02 | 1.10 | 1.01 | 0.93 | 0.90 | *2.4* | *6.3* | *19.4* | *46.1* | *25.8* | | |
| **MEAN** | **4.24** | **3.94** | **3.96** | **4.16** | **3.95** | **4.15** | **4.16** | **4.01** | | | | | | **4.05** | **0.65** |
| **STD DEV** | **0.59** | **0.59** | **0.59** | **0.66** | **0.64** | **0.78** | **0.65** | **0.64** | | | | | | | |

**Source: Author's Computation (2017)**

Table 4.8 above shows the descriptive statistics of responses on the role of corporate reputation in an organisation's sustainability. From responses obtained, the most agreeable statement (87%) is the statement that "My bank is highly regarded for the quality of its products and services" (M=4.16, SD=0.73). This is followed closely at (86%) by "My bank has strong record of financial soundness" (M=4.14, SD=0.73), while the last item measured" My bank has global competitiveness (M=3.87,

SD=0.95) had 72% agreement. Across the banks, there is a positively strong disposition towards their organisation's corporate reputation. As shown above, BK#1 ranks highest with a mean score of 4.24 (SD=0.59), while BK#2 ranks the least with a mean score of 3.94 (SD=0.59). Overall, the findings indicate that the respondents agreed with all items, with an overall mean of 4.05 (SD=0.65). All mean scores for the items ranged between 3.87 and 4.16, thus the majority of participants agreed, albeit to varying degrees.

#### 4.3.2.4 Descriptive Statistics of responses on Financial Performance

The following table shows the descriptive statistics of responses from the eight banks included in this research study on financial performance variables, which is measured by four (4) items – profitability, revenue growth, share price, and market share. Analysis of the mean score and standard deviation for each measured item on individual bank basis, as well as the total sample Likert-response rate in frequency and percentage is shown in the tables below:

**Table 4.9: Descriptive Statistics Financial Performance**

| | BANK CODE | | | | | | | | LIKERT RESPONSE (freqand%) | | | | | STAT | |
|---|---|---|---|---|---|---|---|---|---|---|---|---|---|---|---|
| **Financial Performance** | **#1** | **#2** | **#3** | **#4** | **#5** | **#6** | **#7** | **#8** | **1** | **2** | **3** | **4** | **5** | **M** | **SD** |
| Profitability | 4.16 | 3.25 | 3.32 | 4.19 | 3.14 | 4.55 | 3.93 | 4.60 | 26 | 50 | 107 | 200 | 190 | 3.83 | 1.12 |
| | 0.99 | 1.05 | 0.99 | 0.82 | 1.01 | 0.98 | 1.05 | 0.74 | *4.5* | *8.7* | *18.7* | *34.9* | *33.2* | | |
| Revenue growth (Turnover) | 4.05 | 4.01 | 3.21 | 4.35 | 3.71 | 4.42 | 3.89 | 4.39 | 15 | 25 | 100 | 249 | 184 | 3.98 | 0.95 |
| | 1.13 | 0.77 | 0.94 | 0.69 | 0.93 | 0.86 | 0.90 | 0.79 | *2.6* | *4.4* | *17.5* | *43.5* | *32.1* | | |
| Share Price | 3.70 | 3.21 | 3.26 | 4.00 | 2.72 | 4.44 | 3.62 | 4.28 | 26 | 56 | 181 | 174 | 136 | 3.59 | 1.09 |
| | 1.13 | 1.13 | 0.90 | 0.85 | 0.82 | 0.86 | 0.96 | 0.81 | *4.5* | *9.8* | *31.6* | *30.4* | *23.7* | | |
| Market Share | 3.81 | 3.48 | 3.42 | 4.16 | 2.81 | 4.35 | 3.75 | 4.27 | 20 | 53 | 140 | 232 | 128 | 3.69 | 1.03 |
| | 0.91 | 0.91 | 0.78 | 0.84 | 1.02 | 0.83 | 0.91 | 0.81 | *3.5* | *9.2* | *24.4* | *40.5* | *22.3* | | |
| **MEAN** | **4.09** | **3.71** | **3.63** | **4.17** | **3.52** | **4.29** | **3.98** | **4.20** | | | | | | **3.91** | **0.62** |
| **STD DEV** | **0.57** | **0.55** | **0.56** | **0.52** | **0.53** | **0.68** | **0.53** | **0.50** | | | | | | | |

**Source: Author's Computation (2017)**

Table 4.9 above shows the descriptive statistics of responses on the role of financial performance in an organisation's sustainability. From responses obtained, the most agreeable statement (76%) is the statement that "My bank's revenue growth (Turnover) has improved compared to peers" (M=3.98, SD=0.95). About a seventh of respondents (13%) expressed outright disagreement with the statements measuring their organisation's financial performance with the other three measures. As shown above, BK#6 ranks highest with a mean score of 4.29 (SD=0.68), while BK#5 ranks the least with a mean

score of 3.52 (SD=0.53). In specific terms, they rank the financial performance of their organisation as: revenue growth, profitability, market share, and share price in descending order. Overall, the findings indicate that the respondents agreed with all items, with an overall mean of 3.91 (SD=0.62). All mean scores for the items ranged between 3.59 and 3.98, thus the majority of participants agreed, albeit to varying degrees.

## 4.4 Test of Hypotheses

This section tests hypothesis 1-6. Hypothesis 1-3 was tested by questionnaire, while hypothesis 4-5 was tested by secondary data (content analysis), and hypothesis 6 by secondary data (index). The section also includes thematic analysis from the semi-structured interview.

### 4.4.1 Test of Hypothesis One

Hypothesis one was formulated based on the following premise of research question and research objective

**Research Objective 1:**

To assess the effects of internal governance controls on corporate social performance in selected deposit-money banks in Nigeria

**Research Question 1:**

To what extent do internal governance controls influence corporate social performance of deposit-money banks in Nigeria?

Based on the above research question and objective, hypothesis one was stated in both the null and alternate forms as below:

**Research Hypothesis One**

$H_0$: Internal governance controls do not have significant effect on corporate social performance in selected deposit-money banks in Nigeria.

$H_a$: Internal governance controls have significant effect on corporate social performance in selected deposit-money banks in Nigeria.

Hypothesis One was statistically tested using correlation and regression analysis to (i) identify whether or not there is a relationship, and (ii) examine the degree of the relationship, between the independent

(that is, internal governance controls) and dependent variables (corporate social performance); (iii) to analyse the significant effect of the variables under study.

**Step 1 and 2:**

To identify whether or not there is a relationship, and to examine the degree of the relationship among internal governance controls, and corporate social performance using Pearson Product-Moment Correlation Coefficient. The correlation analysis is represented by the symbol *r* and it reflects the degree of linear relationship between two variables.

**Decision Rule:**

Using the guide that Evans (1996) suggests for the absolute value of *r*:

.00-.19 - "very weak" ; .20-.39 – "weak" , .40-.59 – "moderate", .60-.79 – "strong",.80-1.0 – "very strong".

### 4.4.1.1 Internal Governance Controls and Corporate Social Performance

To test "$H_0$ - Internal governance controls do not have significant effect on corporate social performance in selected deposit-money banks in Nigeria.

#### 4.4.1.1.1 Correlation Coefficient

The test of hypothesis one (1) starts with a correlation matrix.

**Table 4.10: Correlation Matrix Internal Governance Controls and Corporate Social Performance (CSP)**

| | | Corp_Soc_Perf | Int_gov_Cont |
|---|---|---|---|
| Corp_Soc_Perf | Pearson Correlation | 1 | .479** |
| | Sig. (2-tailed) | | .000 |
| | N | 573 | 573 |
| Int_Gov_Cont | Pearson Correlation | .479** | 1 |
| | Sig. (2-tailed) | .000 | |
| | N | 573 | 573 |

**. Correlation is significant at the 0.01 level (2-tailed).

**Source: Author's Computation (2017)**

In table 4.10 above, the relationship between internal governance controls and corporate social performance was investigated using Pearson product-moment correlation coefficient. There was a moderate, positive correlation between internal governance controls and corporate social performance, $r = .479$, $n = 573$, $p < .001$.

**4.4.1.1.2 Regression Analysis between Internal Governance Controls and Corporate Social Performance (CSP)**

Having determined the strength and direction of relationship between internal governance controls and corporate social performance in Table 4.10 above, a multiple regression analysis was conducted to determine the significant contribution of the three measures of internal governance controls to corporate social performance based on the combined eight banks sampled. The results of this analysis revealed how much of the corporate social performance can be explained by internal governance controls, which was also further deconstructed to determine the specific contribution of board structure, management, and ownership structure. Preliminary analyses were conducted to ensure no violation of the assumptions of normality, linearity, multi-collinearity and homoscedasticity.

The model tested is:

Corporate Social Performance = *f* (internal governance controls),

Where, internal governance controls is represented by:

i. Board structure
ii. Management structure
iii. Ownership structure

The results are as shown below:

**Table 4.11: Model Summary –Internal Governance Controls and CSP**

| Model | R | R Square | Adjusted R Square | Std. Error of the Estimate |
|---|---|---|---|---|
| 1 | .498[a] | .248 | .244 | .36930 |

a Predictors: (Constant), Ownership_Structure, Board_Structure, Mgt_Structure

b. Dependent Variable: corporate social performance

**Source: Author's Computation (2017)**

**Interpretation of Results:**

The results from the model summary table 4.11 above revealed the extent to which the variance in the dependent variable (corporate social performance) is explained by the independent variable (internal governance controls). In this case the R-squared is .248, which expressed as a percentage equals 24.8%. This connotes that 24.8% of the variance in corporate social performance can be predicted from the variables Board, Mgt., and Ownership structure. The adjusted R-squared which identifies the percentage of variance in the dependent variable that is explained by the independent variables shows .244 (that is 24.4%) variability of the independent variable (internal governance controls components:

board, management, and ownership) while the standard error of the estimate indicates .36930 which signifies error term.

**Table 4.12: ANOVA –Internal Governance Controls and CSP**

| Model | | Sum of Squares | df | Mean Square | F | Sig. |
|---|---|---|---|---|---|---|
| 1 | Regression | 25.545 | 3 | 8.515 | 62.436 | .000[b] |
| | Residual | 77.601 | 569 | .136 | | |
| | Total | 103.146 | 572 | | | |

b. Predictors: (Constant), Board_Structure, Ownership_Structure, Management_Structure

**Source: Author's Computation (2017)**

Table 4.12 above assesses the statistical significance of the three measures of internal governance controls (board, mgt, and ownership) on corporate social performance. This analysis tests the null hypothesis that multiple R in the population equals 0. The rule is that, a model reaches statistical significance when Sig. = .000; this in other word means that p<.0005.

Therefore, from table 4.12, internal governance controls are statistically significant to corporate social performance, where Sig. = .000 {F (3, 569) = 62.436}.

**Decision Rule:** Reject the Null hypothesis, when the significance value is below 0.05. Do not reject hypothesis, when significance value is greater than 0.05.

**Interpretation of Result:** The ANOVA table shows that the F value is 62.436 at .000[b] Significance level. The implication is that internal governance controls has a significant effect on corporate social performance.

**Decision:** Reject the null hypothesis. Therefore, there is a significant effect of internal governance controls on corporate social performance.

**Table 4.13: Coefficients –Internal Governance Controls and CSP**

| Model | | Unstandardized Coefficients | | Standardized Coefficients | | | Correlations | | |
|---|---|---|---|---|---|---|---|---|---|
| | | B | Std. Error | Beta | t | Sig. | Zero-order | Partial | Part |
| 1 | (Constant) | 2.102 | .146 | | 14.413 | .000 | | | |
| | Board_str | .212 | .039 | .234 | 5.368 | .000 | .413 | .220 | .195 |
| | Mgt_Str | .225 | .034 | .310 | 6.589 | .000 | .453 | .266 | .240 |
| | Own_str | .031 | .037 | .037 | .833 | .405 | .301 | .035 | .030 |

a. Dependent Variable: corporate social performance

**Source: Author's Computation (2017)**

**Interpretation of Result:**

The coefficient table 4.13 above shows the simple model that expresses the extent to which internal

governance controls has an effect on corporate social performance and which of the variables included in the model contributed to the prediction of the dependent variable. The study is interested in comparing the contribution of each independent variable; therefore, beta values are used for the comparison.

Preliminary analyses were performed to ensure no violation of the assumptions of normality, linearity and homoscedasticity. Multicollinearity tests (tolerance and VIF) were also performed and found within range, that is tolerance tests showing individual values above 0.20 (Menard, 1995) and VIF with individual value below 10 (Myers, 1990). There was a moderate positive relationship between board structure; management structure; and corporate social performance, while ownership structure had a weak relationship, $r = .413$, $n = 573$, $p < .001$; $r = .453$, $n = 573$, $p < .001$; and $r = .301$, $n = 573$, $p > .005$ respectively. The part correlation column tells us how much of the total variance in social responsibility is uniquely explained by the three measures of internal governance controls. Board structure makes less 3.8 % (.195*.195), Management structure less 5.76% (.240*.240), and ownership structure less 0.09% (.030*.030) unique contribution to explain corporate social performance.

The model revealed that management structure had the most statistical significance in predicting corporate social performance, recording the highest beta value of (*beta* = .310, with a $T_{val}$ higher than 1.96, *Sig*. .000 $p < .05$), followed by board structure with a beta value of (*beta* = .234, with a $T_{val}$ higher than 1.96, *Sig*. .000 p < .05). On the other hand, there is no statistical significant effect of ownership structure on corporate social performance, even though for every unit increase in ownership_Structure, there is a 0.037-unit increase in the predicted corporate social performance , holding all other variables constant, with a beta value (*beta* = .037, with a $T_{val}$ lower than 1.96, *Sig*. .405 p > .05.

The regression equation from the above table is:
Corporate Social Performance Predicted = 2.102+ .212*boardstr +.225*Mgt str +.031*own_str.

This by interpretation means that for every unit increase in Board_Structure, a .212-unit increase in corporate social performance is predicted, holding all other variables constant. Similarly, for every unit increase in Management_Structure, there is a .225-unit increase in the predicted corporate social performance, holding all other variables constant, while for every one (1) unit increase in the Ownership_Structure, we expect an approximately .031-point increase in corporate social performance. This means that management structure makes the strongest unique contribution to explaining corporate social performance.

In order to probe the results, a further analysis (appendix "H") was carried out to determine the specific component(s) of the board, management, and ownership structures contributing to corporate social performance (i.e. social performance and environmental performance). The disaggregated results show that board size has positive significance on both social and environmental performance, while board committees has significance on environmental performance only. Other components of board structure were not significant at 5% level. CEO Tenure, and Incentive-based compensation have a positive significant influence on social, and environmental performance, while CEO remuneration has positive significant effect only on social performance. Other components of management structure were not significant at 5% level. Directors share ownership, and concentrated ownership have significant influence on environmental performance, while employee share ownership has influence on social performance. Other components of ownership structure were not significant at 5% level.

**Decision:**

The significance level below 0.01 implies a statistical confidence of above 99%. This implies that the internal governance control has a significant effect on corporate social performance. Thus, the null hypothesis ($H_{01}$) was rejected; while the alternative hypothesis ($Ha_1$) which says that internal governance control has a significant effect on corporate social performance is accepted.

### 4.4.2 Test of Hypothesis Two

Hypothesis two was formulated based on the following premise of research question and research objective:

**Research Objective 2:**

To examine the extent to which ethical leadership influence corporate reputation

**Research Question 2:**

What effect does ethical leadership have on banks' corporate reputation?

Based on the above research question and objective, hypothesis two was stated in both the null and alternate forms as below:

**Research Hypothesis Two**

$H_0$: Ethical leadership does not have significant effect on corporate reputation in selected deposit-money banks in Nigeria.

$H_a$: Ethical leadership has significant effect on corporate reputation in selected deposit-money banks in Nigeria.

Hypothesis Two was statistically tested using correlation and regression analysis to (i) identify whether or not there is a relationship, and (ii) examine the degree of the relationship, between the independent variable (ethical leadership) and dependent variable (Corporate reputation); (iii) to analyse the significant effect of the variables under study.

**Step 1 and 2:**

To identify whether or not there is a relationship, and to examine the degree of the relationship, between Ethical leadership and Corporate reputation using Pearson Product-Moment Correlation Coefficient. The correlation analysis is represented by the symbol *r* and it reflects the degree of linear relationship between two variables.

**Decision Rule:**

Using the guide that Evans (1996) suggests for the absolute value of *r*:

.00-.19 - “very weak” ; .20-.39 – “weak” , .40-.59 – “moderate”, .60-.79 – “strong”,

.80-1.0 – “very strong”.

## 4.4.2.1 Ethical leadership and Corporate Reputation

For ‘Ethical leadership does not have significant effect on corporate reputation in selected deposit-money banks in Nigeria’.

### 4.4.2.1.1 Correlation Coefficient

The test of hypothesis two (2) starts with a correlation matrix. The correlation analysis was done based on all the sampled banks combined. Below is the result:

**Table 4.14: Correlation Coefficient - Ethical leadership and Corporate Reputation**

| | | Eth_Lead_Cul | Corp_Rep |
|---|---|---|---|
| Eth_Lead_Cul | Pearson Correlation | 1 | .468** |
| | Sig. (2-tailed) | | .000 |
| | N | 573 | 573 |
| Corp_Rep | Pearson Correlation | .468** | 1 |
| | Sig. (2-tailed) | .000 | |
| | N | 573 | 573 |

**. Correlation is significant at the 0.01 level (2-tailed).

**Source: Author’s Computation (2017)**

In table 4.14 above, the relationship between ethical leadership and corporate reputation was investigated using Pearson product-moment correlation coefficient. There was a moderate, positive correlation between ethical leadership and corporate reputation, $r = .468$, $n = 573$, $p < .001$

#### 4.4.2.1.2 Regression Analysis between Ethical leadership and Corporate Reputation

Having determined the strength and direction of relationship between ethical leadership and corporate reputation, a multiple regression analysis was conducted to determine the significant contribution of the three measures of ethical leadership to corporate reputation based on the combined eight banks sampled. The regression analysis, specifically, regression and analysis of variance were employed to test the hypothesis. This was used to examine the predictive capabilities of ethical leadership on corporate reputation among the sampled banks in Nigeria.

The results of this analysis revealed how much of the corporate reputation can be explained by ethical leadership, which was also further decomposed to determine the specific contribution of CEO's ethics, Organisational ethical program, and ethical culture. Preliminary analyses were conducted to ensure no violation of the assumptions of normality, linearity, multi-collinearity and homoscedasticity.

The model tested is:

Corporate reputation = *f* (Ethical leadership),

Where, Ethical leadership is represented by:

i. CEO's Ethics
ii. Organisational Ethical Culture
iii. Ethical Program

The results are as shown below:

**Table 4.15: Model Summary - Ethical leadership and Corporate Reputation**

| Model | R | R Square | Adjusted R Square | Std. Error of the Estimate |
|---|---|---|---|---|
| 1 | .581[a] | .337 | .334 | .53110 |

**Source:** Author's Computation (2017)

**Interpretation of Results:**

The results from the model summary table 4.15 above revealed the extent to which the variance in the dependent variable (Corporate reputation) is explained by the independent variable (ethical leadership). In this case the R square is .337, which expressed as a percentage equals 33.7%. This connotes that 33.7% of the variance in in corporate reputation can be predicted from the variables CEO's ethics, Organisational ethical program, and ethical culture. The adjusted R-squared which identifies the percentage of variance in the dependent variable that is explained by the independent variables shows .334 (that is 33.4%) variability of the independent variable (ethical leadership dimensions: CEO's

ethics, Organisational ethical program, and ethical culture) while the standard error of the estimate indicates .53110 which signifies error term.

**Table 4.16: ANOVA - Ethical leadership and Corporate Reputation**

| Model | | Sum of Squares | df | Mean Square | F | Sig. |
|---|---|---|---|---|---|---|
| 1 | Regression | 81.639 | 3 | 27.213 | 96.477 | .000[b] |
| | Residual | 160.497 | 569 | .282 | | |
| | Total | 242.137 | 572 | | | |

a. Dependent Variable: Corporate Reputation

b. Predictors: (Constant), Eth_Culture, Ethical_Prog, CEO_Ethics

Table 4.16 above assesses the statistical significance of the three measures of ethical leadership (CEO's ethics, Organisational ethical program, and ethical culture) on corporate reputation. This analysis tests the null hypothesis that multiple R in the population equals 0. The rule is that, a model reaches statistical significance when Sig. = .000; this in other word means that p<.0005. Therefore, from table 4.31, ethical leadership are statistically significant to Corporate Reputation, where Sig. = .000 {F (3, 569) = 96.477}.

**Decision Rule:** Reject the Null hypothesis, when the significance value is below 0.05. Do not reject hypothesis, when significance value is greater than 0.05.

**Interpretation of Result:** The ANOVA table shows that the F value is 96.477 at .000[b] Significance level. The implication is that ethical leadership (CEO's ethics, Organisational ethical program, and ethical culture) has a significant effect on corporate reputation.

**Decision:** Reject the null hypothesis. Therefore, there is a significant effect of ethical leadership on corporate reputation.

**Table 4.17: Coefficients - Ethical leadership and Corporate Reputation**

| Model | | Unstandardized Coefficients | | Standardized Coefficients | t | Sig. | Correlations | | |
|---|---|---|---|---|---|---|---|---|---|
| | | B | Std. Error | Beta | | | Zero-order | Partial | Part |
| 1 | (Constant) | .918 | .203 | | 4.528 | .000 | | | |
| | CEO_Ethics | .026 | .047 | .026 | .564 | .573 | .230 | .024 | .019 |
| | Eth_Cul | .132 | .061 | .102 | 2.149 | .032 | .324 | .090 | .073 |
| | Eth_Prog | .613 | .043 | .523 | 14.095 | .000 | .570 | .509 | .481 |

a. Dependent Variable: Corporate Reputation

**Source: Author's Computation (2017)**

**Interpretation of Result:**

The coefficient table 4.17 above shows the simple model that expresses the extent to which ethical leadership has an effect on corporate reputation and which of the variables included in the model contributed to the prediction of the dependent variable. The study is interested in comparing the contribution of each independent variable; therefore, beta values are used for the comparison.

Preliminary analyses were performed to ensure no violation of the assumptions of normality, linearity and homoscedasticity. Multicollinearity tests (tolerance and VIF) were also performed and found within range, that is tolerance tests showing individual values above 0.20 (Menard, 1995) and VIF with individual value below 10 (Myers, 1990). There was a weak positive relationship between CEO's ethics; ethical culture and corporate reputation, $r = .230$, $n = 573$, $p < .005$; $r = .324$, $n = 573$, $p < .005$ respectively, and a moderate relationship between ethical programs and corporate reputation, $r = .570$, $n = 573$, $p < .005$. The part correlation column tells us how much of the total variance in corporate reputation is uniquely explained by the three measures of ethical leadership. CEO ethics makes less 0.04 % (.019*.019), Ethical_prog less 0.53% (.073*.073), and ethical_culture less 23.14% (.481*.481) unique contribution to explain corporate reputation.

The model also revealed that ethical program had the most statistical significance in predicting corporate reputation, recording the highest beta value of (*beta* = .523, with a $T_{val}$ higher than 1.96, *Sig.* .000 $p < .05$), followed by ethical culture with a beta value of (*beta* = .102, $T_{val}$ higher than 1.96, *Sig.* .032 p < .05). CEO's ethical value however does not make a significant contribution in explaining corporate reputation, as it shows a beta value (*beta* = .047, $T_{val}$ lower than 1.96, *Sig*. .573 p > .05).

The regression equation from the above table is:

Corporate reputation Predicted = .918+ .026*CEO_xter + .613*Ethical_Prog +.132*Eth_Cul.

This by interpretation means that for every unit increase in CEO's ethics, a .026-unit increase in Corporate reputation is predicted, holding all other variables constant. On the other hand, for every unit increase in ethical program, there is a .613-unit increase in the predicted Corporate reputation, holding all other variables constant, while for every one (1) unit increase in the ethical culture, we expect an approximately .132-point increase in Corporate reputation. This means that ethical program makes the strongest unique contribution to explaining Corporate reputation.

**Decision:**

The significance level below 0.01 implies a statistical confidence of above 99%. This implies that the ethical leadership has an effect on Corporate reputation. Thus, the null hypothesis ($H_{01}$) was rejected; while the alternative hypothesis ($Ha_1$) which says that ethical leadership has a significant effect on Corporate reputation is accepted.

### 4.4.3 Test of Hypothesis Three

Hypothesis three was formulated based on the following premise of research question and research objective:

**Research Objective 3:**

To evaluate the role of regulation on financial innovation.

**Research Question 3:**

To what degree does regulation influence banks' financial innovation?

Based on the above research question and objective, hypothesis three was stated in both the null and alternate forms as below:

**Research Hypothesis Three**

$H_0$: Regulation does not have significant effect on financial innovation in selected deposit-money banks in Nigeria.

Ha: Regulation has significant effect on financial innovation in selected deposit-money banks in Nigeria

Hypothesis Three was statistically tested using correlation and regression analysis to (i) identify whether or not there is a relationship, and (ii) examine the degree of the relationship, between the independent variable (Regulation ) and dependent variable (financial innovation); (iii) to analyse the significant effect of the variables under study.

**Step 1 and 2:**

To identify whether or not there is a relationship, and to examine the degree of the relationship, between Regulation and financial innovation using Pearson Product-Moment Correlation Coefficient. The correlation analysis is represented by the symbol *r* and it reflects the degree of linear relationship between two variables.

**Decision Rule:**

Using the guide that Evans (1996) suggests for the absolute value of *r*:

.00-.19 - "very weak" ; .20-.39 – "weak" , .40-.59 – "moderate", .60-.79 – "strong",

.80-1.0 – "very strong".

### 4.4.3.1 Regulation and Financial Innovation

To test for "$H_0$ – Regulation does not have significant effect on financial innovation in selected deposit-money banks in Nigeria.

#### 4.4.3.1.1 Correlation Coefficient

The study ran a correlation analysis on all the sampled banks combined. Result is shown below:

**Table 4.18: Correlation Coefficient - Regulation and Financial Innovation**

| | | Bkr_Reg_Sup | Fin_Inno |
|---|---|---|---|
| Bkr_Reg_Sup | Pearson Correlation | 1 | .500** |
| | Sig. (2-tailed) | | .000 |
| | N | 573 | 573 |
| Fin_Inno | Pearson Correlation | .500** | 1 |
| | Sig. (2-tailed) | .000 | |
| | N | 573 | 573 |

**. Correlation is significant at the 0.01 level (2-tailed).

**Source: Author's Computation (2017).**

In table 4.18 above, the relationship between regulation and financial innovation was investigated using Pearson product-moment correlation coefficient. There was a moderate, positive correlation between the two variables, $r = .500$, $n = 573$, $p < .001$.

#### 4.4.3.1.2 Regression Analysis between Regulation and Financial Innovation

Having determined the strength and direction of relationship between Regulation and Financial Innovation, a multiple regression analysis was conducted to determine the significant contribution of the six measures of Regulation to Financial Innovation based on the combined eight banks sampled. The results of this analysis revealed how much of the financial innovation can be explained by regulation, which was also further decomposed to determine the specific contribution of disclosure, activity restriction, deposit insurance, entry/exit regulation, supervision, and capital requirements. Preliminary analyses were conducted to ensure no violation of the assumptions of normality, linearity, multi-collinearity and homoscedasticity.

The model tested is:

Financial Innovation = *f* (Regulation),

Where, Regulation is represented by:

i. Supervision quality
ii. Capital requirements
iii. Deposit insurance
iv. Entry/Exit barriers
v. Disclosure
vi. Activity restriction

The results are as shown below:

**Table 4.19: Model Summary- Regulation and Financial Innovation**

| Model | R | R Square | Adjusted R Square | Std. Error of the Estimate |
|---|---|---|---|---|
| 1 | .584[a] | .341 | .334 | .41866 |

a. Predictors: (Constant), Cap Req, Discl., Entry-exit, DepoIns, Act. Restr., Superv qual.

b. Dependent Variable: Financial Innovation

**Interpretation of Results:**

The results from the model summary table 4.19 above revealed the extent to which the variance in the dependent variable (Financial innovation) is explained by the independent variable (regulation). In this case the R square is .341, which expressed as a percentage equals 34.1%. This connotes that 34.1% of the variance in financial innovation can be predicted from the variables - disclosure, activity restriction, deposit insurance, entry/exit regulation, supervision, and capital requirements. The adjusted R-squared which identifies the percentage of variance in the dependent variable that is explained by the independent variables shows .334 (that is 33.4%) variability of the independent variable (regulation dimensions: disclosure, activity restriction, deposit insurance, entry/exit regulation, supervision, and capital requirements) while the standard error of the estimate indicates .41866 which signifies error term.

**Table 4.20: ANOVA- Regulation and Financial Innovation**

| Model | | Sum of Squares | df | Mean Square | F | Sig. |
|---|---|---|---|---|---|---|
| 1 | Regression | 51.345 | 6 | 8.558 | 48.824 | .000[b] |
| | Residual | 99.204 | 566 | .175 | | |
| | Total | 150.550 | 572 | | | |

a. Dependent Variable: Financial_Innovation

b. Predictors: (Constant), Cap Req, Discl, Entry-exit, DepoIns, act- rest, Qual Superv

Table 4.20 above assesses the statistical significance of the six measures of regulation (disclosure, activity restriction, deposit insurance, entry/exit regulation, supervision, and capital requirements) on financial innovation. This analysis tests the null hypothesis that multiple R in the population equals 0. The rule is that, a model reaches statistical significance when Sig. = .000; this in other word means that p<.0005. Therefore, from table 4.20, Regulation is statistically significant to Financial innovation, where Sig. = .000 {F (6, 566) = 48.824}.

**Decision Rule:** Reject the Null hypothesis, when the significance value is below 0.05. Do not reject hypothesis, when significance value is greater than 0.05.

**Interpretation of Result:** The ANOVA table shows that the F value is 48.824 at .000[b] Significance level. The implication is that regulation (activity restriction, supervision, and capital requirements at 5%, and disclosure at 10%) has a significant effect on financial innovation.

**Decision:** Reject the null hypothesis. Therefore, there is a significant effect of regulation on financial innovation.

**Table 4.21: Coefficients- Regulation and Financial Innovation**

| Model | | Unstandardized Coefficients | | Standardize Coefficients | t | Sig. | Correlation |
|---|---|---|---|---|---|---|---|
| | | B | Std. Error | Beta | | | |
| 1 | (Constant) | 1.316 | 0.181 | | 7.249 | 0.000 | |
| | Sup_Qual | 0.485 | 0.054 | 0.420 | 8.987 | 0.000 | 0.546 |
| | cap_req | 0.218 | 0.039 | 0.234 | 5.521 | 0.000 | 0.416 |
| | Depo_Ins | -0.013 | 0.020 | -0.025 | -.678 | 0.498 | 0.079 |
| | Entry_Exit | 0.014 | 0.024 | 0.022 | .561 | 0.575 | 0.269 |
| | Discl | 0.047 | 0.028 | 0.065 | 1.705 | 0.089 | 0.268 |
| | Activity_rest | -0.065 | 0.021 | -0.118 | -3.155 | 0.002 | 0.059 |

a. Dependent Variable: Financial Innovation

**Source: Author’s Computation (2017)**

**Interpretation of Result:**

The coefficient table 4.21 above shows the simple model that expresses the extent to which regulation has an effect on financial innovation, and which of the variables included in the model contributed to the prediction of the dependent variable. The study is interested in comparing the contribution of each independent variable; therefore, beta values are used for the comparison.

Preliminary analyses were performed to ensure no violation of the assumptions of normality, linearity and homoscedasticity. Multicollinearity tests (tolerance and VIF) were also performed and found within range, that is tolerance tests showing individual values above 0.20 (Menard, 1995) and VIF with individual value below 10 (Myers, 1990). There was a weak positive relationship between disclosure, activity restriction, deposit insurance, entry/exit regulation and financial innovation, $r = .268$, $n = 573$, $p < .005$; $r = .059$, $n = 573$, $p > .005$, $r = .079$, $n = 573$, $p > .005$, $r = .269$, $n = 573$, $p < .005$ respectively, and a moderate relationship between supervision, and capital requirements and financial innovation, $r = .546$, $n = 573$, $p < .005$, and $r = .416$, $n = 573$, $p < .005$ respectively.

The part correlation column tells us how much of the total variance in financial innovation is uniquely explained by the six measures of regulation. Sup_Qual makes less 1.25%, cap_req makes less 2.31%, Depo_Ins less 0.11%, Entry_Exit less 0.14%, Discl less 3.76%, and Activity_rest less 2.43% unique contribution to explain financial innovation.

The model also revealed that quality of supervision had the most statistical significance in predicting financial innovation, recording the highest beta value of (*beta* = .420, with a $T_{val}$ higher than 1.96, *Sig.* .000 $p < .05$), followed by capital requirements with a beta value of (*beta* = .234, $T_{val}$ higher than 1.96, *Sig*. .000 $p < .05$), and activity restriction albeit a negative influence with a beta value of (*beta* = -.118, $T_{val}$ higher than 1.96, *Sig*. .002 $p < .05$). Disclosure, deposit insurance, and entry and exit regulations however do not make a significant contribution in explaining financial innovation, as they show beta values (*beta* = .065, $T_{val}$ lower than 1.96, *Sig*. .573 $p > .05$, *beta* = -.025, $T_{val}$ lower than 1.96, *Sig*. .573 $p > .05$, *beta* = .022, $T_{val}$ lower than 1.96, *Sig*. .573 $p > .05$) respectively.

The regression equation from the above table is:

Financial Innovation Predicted = 1.316+ .047*Discl - .065*Act Rest -.013*DepoIns +.014*Ent-Exit +.485Qual Sup +.218*Cap Req.

This by interpretation means that for every unit increase in Discl, ent-exit, qualsup, and cap req, a .047, .014, .485, and .218 unit increase respectively in financial innovation is predicted, holding all other variables constant. On the other hand, for every unit increase in act-rest, and depoins, there is a .065, and .013 unit decrease in the predicted financial innovation, holding all other variables constant. This means that quality of supervision makes the strongest unique contribution to explaining financial innovation.

**Decision:**

The significance level below 0.01 implies a statistical confidence of above 99%. This implies that regulation has an effect on financial innovation. Thus, the null hypothesis ($H_0$) was rejected; while the alternative hypothesis (Ha) which says that regulation has a significant effect on financial innovation is accepted.

### 4.4.3.2 Modelling the Regression Effect Using Structural Equation Modelling

A structural equation modelling, as recommended by Anderson and Gerbing (1998) was applied to assess construct validity, and model fits for objectives one (1) to three (3) which were tested using the questionnaire.

Bentler and Wu (2002); Bentler and Bonett (1980); and Kaplan (2000) argued that different indicators of goodness-of-fit are usually adopted in various research concepts. Further, the higher the number of the indices of indicators, the acceptable range of a good fit should be: Normed Fit Index (NFI) =>.90; and Comparative Fit Index (CFI) acceptable value =>.90. Other informative indices that measure the close association between the model and the data include Root Mean Squared Error of Approximation (RMSEA); Goodness of fit (GFI); etc. This is shown in Table 4.24

**Table 4.22: Model fit index – Hypothesis 1-3**

| Model-Fit Index | Score | Recommended Cut-off Values |
|---|---|---|
| **Normed Fit Index (NFI)** | .920 | => .90 |
| **Comparative Fit Index (CFI)** | .911 | = > .90 |
| **Root Mean Squared Error of Approximation (RMSEA)** | .046 | .05 or less = good |
| **Goodness of Fit (GFI)** | .986 | = > .90 |

**Interpretation:**

*As shown in Table 4.22, all the model-fit indices exceeded the respective common acceptance levels suggested by previous research, demonstrating that the measurement model exhibited a good and satisfactory fit with the data collected.*

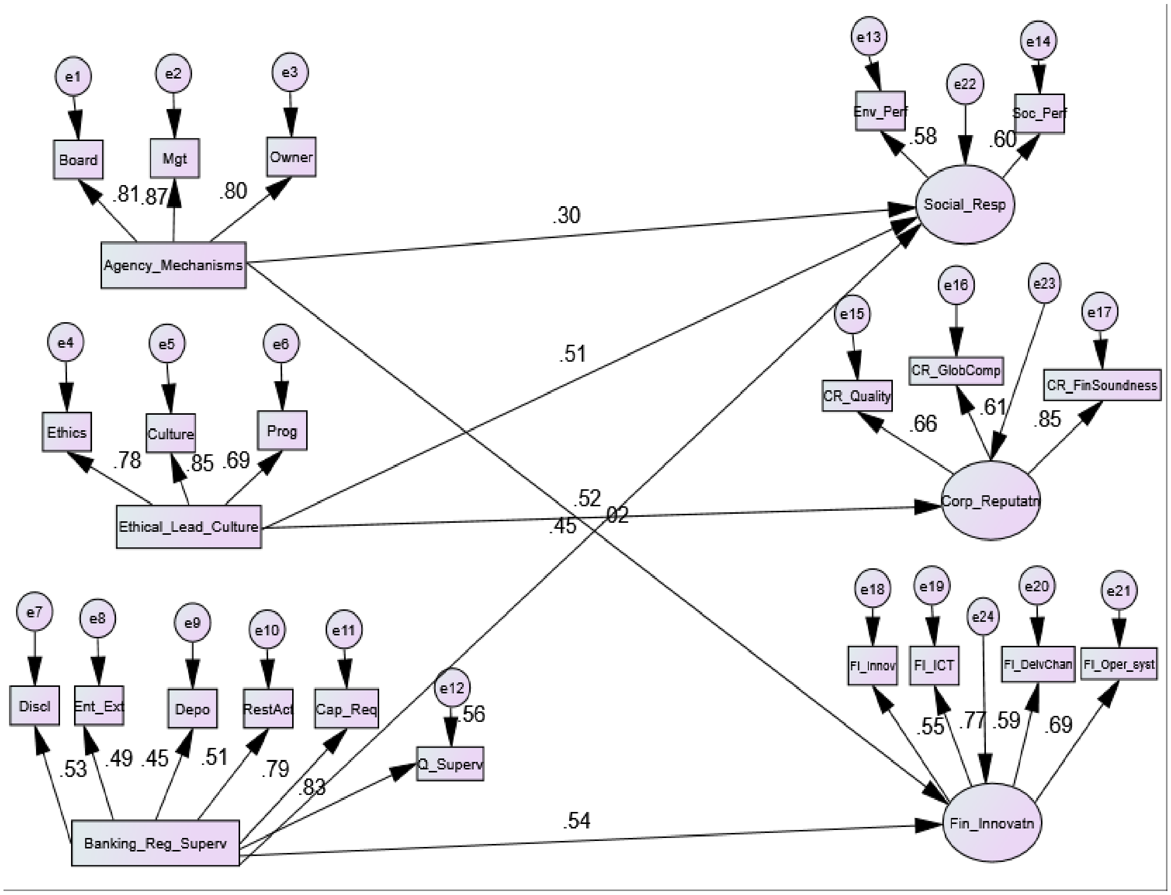


**Fig. 4.1: Model fit index –Hypothesis 1-3**

**Table 4.23: Standardized Regression Weights**

| | | | Estimate |
|---|---|---|---|
| Social_Resp | <--- | Agency_Mechanisms | .298 |
| Fin_Innovatn | <--- | Agency_Mechanisms | .016 |
| Social_Resp | <--- | Ethical_Lead_Culture | .515 |
| Corp_Reputatn | <--- | Ethical_Lead_Culture | .522 |
| Fin_Innovatn | <--- | Banking_Reg_Superv | .544 |
| Social_Resp | <--- | Banking_Reg_Superv | .449 |
| Board | <--- | Agency_Mechanisms | .815 |
| Mgt | <--- | Agency_Mechanisms | .867 |
| Owner | <--- | Agency_Mechanisms | .798 |
| Ethics | <--- | Ethical_Lead_Culture | .777 |
| Culture | <--- | Ethical_Lead_Culture | .851 |

| | | | Estimate |
|---|---|---|---|
| Prog | <--- | Ethical_Lead_Culture | .689 |
| Discl | <--- | Banking_Reg_Superv | .528 |
| Depo | <--- | Banking_Reg_Superv | .446 |
| RestAct | <--- | Banking_Reg_Superv | .515 |
| Cap_Req | <--- | Banking_Reg_Superv | .793 |
| Env_Perf | <--- | Social_Resp | .584 |
| Soc_Perf | <--- | Social_Resp | .597 |
| CR_Quality | <--- | Corp_Reputatn | .662 |
| CR_GlobComp | <--- | Corp_Reputatn | .610 |
| CR_FinSoundness | <--- | Corp_Reputatn | .853 |
| FI_Innov | <--- | Fin_Innovatn | .550 |
| FI_ICT | <--- | Fin_Innovatn | .766 |
| FI_DelvChan | <--- | Fin_Innovatn | .594 |
| Q_Superv | <--- | Banking_Reg_Superv | .828 |
| Q_Superv | <--- | e12 | .561 |
| FI_Oper_syst | <--- | Fin_Innovatn | .692 |
| Ent_Ext | <--- | Banking_Reg_Superv | .486 |

**Interpretation**

The regression weights in table 4.23 show that when regulation variables increase by one (1) unit, social responsibility and financial innovation will correspondingly increase by 45% and 54% respectively; when ethical leadership variables increase by one (1) unit, corporate reputation and social responsibility will correspondingly increase by 52% and 51% respectively; and when internal governance controls variables increase by one (1) unit, financial innovation and social responsibility will correspondingly increase by 2% and 30% respectively. Overall, all the standardised item loadings confirm that the items successfully contributed to each distinct factor tested in the model.

### 4.4.4 Test of Hypothesis Four

This section provides results obtained using secondary data on the research objective, "Agency Mechanisms and Financial Performance". Since the study is made up of time series and cross-sectional observations from the period 2006 to 2016, it is essential to carry out a panel regression analysis involving consistent fixed effect estimates, efficient random effect estimates and Hausman test. Thus, to determine between the consistent fixed effect and consistent random effect, the Hausman test was carried out. The Hausman test compares two estimators, the fixed effects model and the random effects model. Under the null hypothesis given by the test, both are consistent, but the random effects model

is a more efficient estimator. Under the alternative, the fixed effects model is consistent, but the random effects model becomes inconsistent. If the P-value of the Chi-square results in the Hausman test being insignificant, then it is safe to use random effects. If on the other hand the result reveals a significant P-value, the appropriate model to use is the fixed effects (Hausman, 1978; Hausman and Taylor, 1981).

The result of the estimated model presents the relationship between the variables included in the model. Econometric analysis has been employed to determine the nature of the relationship existing between banks' financial performance proxied by- Return On Assets (ROA), Return On Equity (ROE), Tobin's Q (TBQ), Net Interest Margin (NIM), and Non-performing Loan (NPL) -as the dependent variable, and the independent variable being agency mechanisms, proxied by CEO Tenure (CEOT), CEO Age (CEOA), CEO Remuneration (CEOR), Board Gender (BGDR), Board Ethnicity (BETHN), Board intensity represented by Total Meetings (TM), and Insider Ownership (INO) as the explanatory variables. Given the unbalanced nature of this panel study which consists of eight observations over eleven years, the study makes use of seven parameter estimates (which cover the board, management, and ownership dimensions of agency mechanisms) as the maximum allowed. Several variables representing different dimensions of Agency mechanisms were tested to determine the best model for this hypothesis, but were dropped in the final analysis for not passing the necessary tests to fit into the model. The dropped variables include: Board size, independence, composition, CEO characteristics (Gender, Education etc), Board chair characteristics (age, tenure, remuneration etc).

This section is focused on the presentation, evaluation and analysis of regression results of the model presented as well as achieving the expected results of the hypothesis. Both descriptive and economic analysis are carried out in this chapter. The descriptive analysis majorly focuses on the unique statistical characteristics of the variables. The econometric analysis includes the pooled regression, Hausman test result and consistent fixed regression analysis.

Hypothesis four was formulated based on the following premise of research question and research objective:

**Research Objective 4:**

To ascertain the influence of agency mechanisms on financial performance of banks.

Based on the above research question and objective, hypothesis four was stated in both the null and alternate forms as below:

**Research Hypothesis Four**

$H_0$: Agency mechanisms do not have significant effect on financial performance.

$H_a$: Agency mechanisms have significant effect on financial performance

### 4.4.4.1 Agency Mechanisms on Financial Performance

The table below shows the descriptive statistics of the study variables on Agency mechanisms and financial performance. The relevance of showing the descriptive statistics is to identify the pattern of observations in the panel data used to test this hypothesis.

**Table 4.24: Descriptive Statistics Agency Mechanisms and Financial Performance**

| | Mean | Maximum | Minimum | Std. Dev. | # Obs |
|---|---|---|---|---|---|
| **Independent Variables** | | | | | |
| BETHN | 8.69 | 14.00 | 3.00 | 2.57 | 88 |
| BGDR | 11.67 | 33.33 | 0.00 | 10.63 | 88 |
| CEOA | 48.58 | 58.00 | 34.00 | 5.21 | 88 |
| CEOR | 66.56m | 205m | 6.5m | 44.88 | 88 |
| CEOT | 4.43 | 19.00 | 1.00 | 3.68 | 88 |
| INO | 1.32bn | 7.2bn | 47.7m | 1.34b | 88 |
| TM | 24.48 | 50.00 | 8.00 | 10.31 | 88 |
| | | | | | |
| | | | | | |
| **Dependent Variables** | | | | | |
| ROA | 2.13 | 5.94 | -2.26 | 1.52 | 88 |
| ROE | 14.73 | 36.56 | -20.89 | 11.05 | 88 |
| TBQ | 1.34 | 5.77 | 0.09 | 1.30 | 88 |
| NIM | 13.99 | 32.39 | 5.81 | 4.13 | 88 |
| NPL | $3.88e^{10}$ | $5.84e^{11}$ | $2.31e^{9}$ | $7.18e^{10}$ | 88 |

**Source: Author's Computation (2017)**

From table 4.24 above, descriptive statistics are provided per unit in absolute numbers for the variables: Board ethnicity (BETHN), CEO age (CEOA), CEO remuneration (CEOR), CEO tenure (CEOT), Insider Ownership (INO), Total Meeting (TM), and Non-Performing Loan (NPL). Values are expressed in percentages for board gender (BGDR), Return on Assets (ROA), Return on Equity (ROE), Tobin's' Q ratio (TBQ), and Net Interest Margin (NIM). The table shows eighty-eight observations, representing data for eleven years (2006-2016) for each of the eight sampled banks.

Table 4.25 below shows the source of data for the independent variables considered under hypothesis four (4). The dependent variables (ROA, ROE, TBQ, NIM, and NPL) were computed by the researcher

from the financial statements embedded in the banks' annual report. The formula for each dependent variable is in table 3.7

**Table 4.25: Source of data for agency mechanisms**

| Variables | Source | Reference Section | Remarks |
|---|---|---|---|
| Board Ethnicity | Annual Report | Board of Directors, "Directors, officers, and professional advisers" | Director's Bio data, Contacts with banks' Human Capital Management Department, and Internet Search. Total number of board members analysed into 3 ethnic groups -Yoruba, Ibo, and Hausa. Ethnic dominancy is determined by percentage of ethnic concentration. |
| CEO Age | Annual Report | Board of Directors' profile | Director's Bio data, Contacts with banks' Human Capital Management, and Internet Search. Researcher computes age from disclosed/obtained date of birth |
| CEO Remuneration | Annual Report | Directors' remuneration | CEO's compensation is in 'Notes to the account' |
| CEO Tenure | Annual Report | Board of Directors, Corporate Governance Report | Researcher computes tenure from date of CEO's assumption of office |
| Insider Ownership | Annual Report | Directors' report, "Directors and their interests" | Researcher computes total board share ownership from disclosure under "Directors' shareholding interests" |
| Total Meeting | Annual Report | Board of Directors, Corporate Governance Report, "Attendance of Board and Board Committee Meetings" | Researcher computes total meetings from disclosure of board meetings |
| Return on Asset | Annual Report | Financial Statement | Researcher's computation |
| Return on Equity | Annual Report | Financial Statement | Researcher's computation |
| Tobin's Q | Bank's website & Stock Reports | http://www.marketwatch.ng/stock; www.tradingeconomics.com ; www.capitalassets.com.ng<br><br>Investors' Relations -bank website | Researcher's computation |
| Net Interest Margin | Annual Report | Financial Statement | Researcher's computation |
| Non-Performing Loans | Annual Report | Financial Statement - Notes to the accounts - Loans and Advances | Researcher's computation from the banks' classification of non-performing loans |

**Source: Author's Computation (2017).**

To investigate the effects of agency mechanisms variables on financial performance of Nigerian deposit money banks, this study used the following dynamic panel regression model expressed in its general form as:

$FINPERF_{it} = \beta_0 it + \beta_1 lnBGDR_{it} + \beta_2 lnBETHN_{it} + \beta_3 lnCEOR_{it} + \beta_4 TM_{it} + \beta_5 lnCEOT_{it} + \beta_6 lnCEOA_{it} + \beta_7 lnINO_{it} + U_{it}$ (Eq. 1)

In this model, the dependent variable is bank's financial performance (FINPERF) measured by return on total assets (ROA), return on equity (ROE), Tobin's Q (Q), Net Interest Margin (NIM) and Non-Performing Loan (NPL). The independent variables consist of seven (7) agency mechanism variables,

namely Board gender (in logarithm) (LBGDR), Board Ethnic composition (in logarithm) (LBETHN), CEO's remuneration (in logarithm) (LCEOR), Board's intensity proxied by total meetings of the board and the committees (TM), CEO's tenure (in logarithm) (LCEOT), CEO's age (in logarithm) (LCEOA), and Insider Ownership (in logarithm) (LINO). $U_{it}$ is the random error term and subscripts i and t represent firm and time period, respectively.

**Analyses of Measures**

**Multicollinearity**

Table 4.26 below presents the correlation matrix and VIF (Variance Inflation Factor) for all the independent and dependent variables used in the analysis. According to the results, there is no multicollinearity among the variables since the inter-correlations among the explanatory variables are low. To check further, another diagnostic test for multicollinearity is used, with the variance inflation factor (VIF) calculated for independent variables as follows:

VIF ($\beta i$) = $1/(1-R^2)$, where $R^2$ is the squared multiple correlation coefficient between $X_i$ and the other independent variables. When $R^2$ is equal to zero, VIF has its minimal value of one (Maddala, 2001). Therefore, the closer the value of VIF to one (1), the degree of multicollinearity is lower. If one of the VIFs is greater than 10, then there is a multicollinearity problem (Gujarati, 1995). Based on the results in table 4.31b, the VIF values range from 1.306 to 1.903 (with average of 1.554), which is lower than 10, thus confirming that multicollinearity does not exist among the independent variables.

**Table 4.26: Correlation Matrix and Variance Inflation Factors**

| | LBETHN | LBGDR | LCEOA | LCEOR | LCEOT | LINO | TM | LROA | LROE | LNIM | LTBQ | LNPL |
|---|---|---|---|---|---|---|---|---|---|---|---|---|
| **LBETHN** | 1 | | | | | | | | | | | |
| | ----- | | | | | | | | | | | |
| **LBGDR** | 0.430 | 1 | | | | | | | | | | |
| | 3.431 | ----- | | | | | | | | | | |
| **LCEOA** | -0.172 | -0.422 | 1 | | | | | | | | | |
| | -1.258 | -3.360 | ----- | | | | | | | | | |
| **LCEOR** | 0.142 | 0.360 | 0.365 | 1 | | | | | | | | |
| | 1.036 | 2.779 | 2.830 | ----- | | | | | | | | |
| **LCEOT** | 0.115 | -0.218 | 0.298 | -0.065 | 1 | | | | | | | |
| | 0.835 | -1.609 | 2.248 | -0.468 | ----- | | | | | | | |
| **LINO** | -0.197 | -0.041 | -0.200 | -0.213 | -0.104 | 1 | | | | | | |
| | -1.445 | -0.295 | -1.473 | -1.569 | -0.756 | ----- | | | | | | |
| **TM** | 0.411 | 0.330 | -0.159 | 0.150 | -0.088 | 0.229 | 1 | | | | | |
| | 3.248 | 2.519 | -1.162 | 1.092 | -0.636 | 1.697 | ----- | | | | | |
| **LROA** | 0.360 | -0.023 | 0.280 | 0.169 | 0.224 | -0.464 | -0.347 | 1 | | | | |
| | 2.782 | -0.164 | 2.107 | 1.236 | 1.659 | -3.773 | -2.670 | ----- | | | | |
| **LROE** | 0.268 | 0.036 | 0.155 | 0.076 | 0.141 | -0.409 | -0.327 | 0.930 | 1 | | | |
| | 2.007 | 0.263 | 1.129 | 0.550 | 1.027 | -3.233 | -2.498 | 18.250 | ----- | | | |
| | | | | | | | | | | | | |

| | LBETHN | LBGDR | LCEOA | LCEOR | LCEOT | LINO | TM | LROA | LROE | LNIM | LTBQ | LNPL |
|---|---|---|---|---|---|---|---|---|---|---|---|---|
| **LNIM** | -0.135 | -0.290 | -0.092 | -0.543 | 0.199 | -0.208 | -0.536 | 0.236 | 0.315 | 1 | | |
| | -0.981 | -2.187 | -0.664 | -4.658 | 1.462 | -1.536 | -4.574 | 1.748 | 2.393 | ----- | | |
| **LTBQ** | 0.115 | -0.204 | -0.002 | -0.281 | 0.108 | -0.500 | -0.451 | 0.594 | 0.639 | 0.634 | 1 | |
| | 0.834 | -1.501 | -0.012 | -2.110 | 0.784 | -4.159 | -3.640 | 5.329 | 5.989 | 5.914 | ----- | |
| **LNPL** | -0.264 | 0.187 | -0.101 | 0.248 | -0.294 | 0.132 | 0.079 | -0.490 | -0.519 | -0.363 | -0.473 | 1 |
| | -1.977 | 1.374 | -0.735 | 1.845 | -2.217 | 0.958 | 0.575 | -4.052 | -4.376 | -2.809 | -3.873 | ----- |
| **VIF** | 1.612 | 1.903 | 1.721 | 1.684 | 1.198 | 1.306 | 1.455 | | | | | |

Correlation -Line1
t-Statistic-Line2

This study used panel data analysis technique because the panel data allows for the control of individual heterogeneity, which cannot be controlled by time series and cross section analysis, hence the results could be biased. Both the fixed effects model and the random effects model can be run in a panel data analysis (Baltagi, 1995). To determine which of these regressions should be used in this study, we performed the Hausman test for each model.

## Hausman Test

We analysed whether the equation advanced in each Model was explanatory for any of the five proposed financial performance measures (Appendix C). Hausman test examines whether the difference between the random effects regression and the fixed effects regression is zero. In other words, the null hypothesis says $H_0$: Random effect is preferred. Endogeneity was resolved in the models through the application of Hausman's test (Hausman, 1978; Hausman and Taylor, 1981). "The Hausman's test determines whether there is significant correlation between the unobserved bank-specific random effects and the regressors. If there is no such correlation, then the random effects model may be more powerful and parsimonious. If there is such a correlation, the random effects model would be inconsistently estimated and the fixed effects model would be the model of choice. In addition, it examines whether the adopted models for the hypotheses are appropriate without misspecification". The pooled regression results (OLS), and Hausman's test result suggesting the appropriate use of fixed effects model are contained in Appendix "D"

#### 4.4.4.1.1 Testing of Hypothesis 4(a)

To test the influence of agency mechanisms (proxied by board gender, board ethnicity, total meeting, CEO tenure, CEO age, and insider ownership) on Return on Asset (as a proxy for financial performance). The implicit form of the equation is expressed as:

ROA = f (BGDR, BETHN, TM, CEOT, CEOA, INO) …. Eq. (2)

The explicit form of the equation could be expressed as:

ROA = 0 +$\beta$1 BGDRit +$\beta$2 BETHNit + $\beta$3 TMit + $\beta$4 CEOTit+$\beta$5 CEOQit+ $\beta$6INOit+ Uit ………Eq. (3)

The log form of the equation therefore becomes:

ROA =$\beta$0 + $\beta X_1$ (LBGDR)it + $\beta X_2$ (LBETHN)it + $\beta X_3$ (TM)it + $\beta X_4$ (LCEOT)it + $\beta X_5$ (LCEOA)it + $\beta X_6$ (LINO)it + Uit - ……Eq. (4)

The result is presented below:

**Table 4.27a: Dependent Variable: LROA**

| Variable | Coefficient | Std. Error | t-Statistic | Prob. |
|---|---|---|---|---|
| C | -10.88656 | 6.204920 | -1.754505 | 0.0868 |
| LBETHN | 0.963083 | 0.417730 | 2.305518 | 0.0263 |
| LCEOT | -0.051011 | 0.123737 | -0.412249 | 0.6823 |
| LCEOA | 3.542714 | 1.672624 | 2.118058 | 0.0403 |
| LINO | -0.154484 | 0.085388 | -1.809197 | 0.0778 |
| LBGDR | -0.264949 | 0.197629 | -1.340638 | 0.1874 |
| TM | -0.016045 | 0.013455 | -1.192492 | 0.2399 |

| | | | |
|---|---|---|---|
| R-squared | 0.684390 | Durbin-Watson stat | 1.692498 |
| Adjusted R-squared | 0.592017 | | |
| F-statistic | 7.408938 | | |
| Prob(F-statistic) | 0.000001 | | |

**Source: Author's Computation (2017)**

This section reports the results of the regression analysis. Table 4.27a above includes an analysis of the return on asset (ROA) against the other independent variables. According to the results reported, it is concluded that the regression model fits the data and the whole model is statistically significant (R-squared = 0.68, P-Value = 0.00). The independent variables (LBETHN, LCEOT, LCEOA, LINO, LBGDR, and TM) can therefore be relied on to explain 68% of the variations in the ROA. The Adjusted R-Squared is 0.59 which means about 59% of ROA is explained by independent variables in the model after adjusting for error in data. In other words, in Nigerian deposit money banks, ROA is affected by independent variables in the model. F-statistic (7.41; p-value<0.01) and the Durbin Watson (1.69) individually suggests that the model is of good fit and free from serial auto-correlation effect.

As explained in the previous sections, board gender is used to measure the percentage of female directors on the board. Based on the results in Table 4.27a, board gender has a negative coefficient and

statistically insignificant. Similarly, board intensity, proxied by the total board and board committee meetings(TM), insider ownership (LINO) and CEO tenure (LCEOT) measured by their natural logarithm had negative coefficient and are statistically insignificant, which means they do not appear to influence return on asset (ROA) in Nigerian Deposit money banks. On the contrary, Board ethnic concentration (LBETHN) measured by its natural logarithm and CEO age (LCEOA) are positively correlated to ROA and statistically significant, which means that both LBETHN and LCEOA appear to influence return on asset (ROA) in Nigerian Deposit money banks in similar direction. Specifically, a percentage change in LBETHN and LCEOA results in 0.96 and 3.54 percentage change respectively in banks performance holding other variables at constant. However, in magnitude and direction it could be observed that variations in LCEOA appears to exhibit the highest effect on banks performance. Detailed analysis of the result further suggests that while LINO is significant at 10%, the degree of the responsiveness of the banks performance to the variations in LCEOA is elastic and statistically significant. Thus, all things being equal, a percentage change in LCEOA will result in a greater percentage change in banks performance. Therefore, it could be noted here that LBETHN and LCEOA account for the most significant effect on banks performance (ROA) and could be regarded to play the most significant role in determining the level of performance of the banks within the scope of the present study.

The predicted ROA result from the model is:

LROA = -10.88656 + 0.963083 *LBETHN -0.051011 *LCEOT + 3.542714 *LCEOA – 0.154484 *LINO -0.264949 *LBGDR -0.016045 *TM

**4.4.4.1.2 Testing of Hypothesis 4(b)**

To test the influence of agency mechanisms (proxied by board gender, board ethnicity, CEO remuneration, CEO tenure, CEO age, and insider ownership) on Return on Equity (as a proxy for financial performance).

The implicit form of the equation is expressed as:

ROE = f (BGDR, BETHN, CEOR, CEOT, CEOA, INO) Eq. (5)

The explicit form of the equation could be expressed as:

ROE = 0 + β1 (BGDR)it + β2 (BETHN)it + β3 (CEOR)it + β4 (CEOT)it + β5 (CEOA)it + β6 (INO)it + Uit Eq. (6)

The log form of the equation therefore becomes:

ROE =β0 + βX1 (LBGDR)i,t + βX2 (LBETHN)i,t + βX3 (CEOR)i,t + βX4 (LCEOT)i,t + βX5 (LCEOA)i,t + βX6 (LINO)i,t + Ui,t - Eq. (7)

The test result is shown below:

**Table 4.27b: Dependent Variable: LROE**

| Variable | Coefficient | Std. Error | t-Statistic | Prob. |
|---|---|---|---|---|
| C | -9.627859 | 6.849710 | -1.405586 | 0.1674 |
| LBETHN | 0.278775 | 0.413583 | 0.674047 | 0.5041 |
| LCEOT | -0.150373 | 0.132250 | -1.137033 | 0.2621 |
| LCEOA | 5.266132 | 2.077594 | 2.534726 | 0.0152 |
| LINO | -0.215735 | 0.091392 | -2.360536 | 0.0231 |
| LBGDR | -0.117252 | 0.238021 | -0.492610 | 0.6249 |
| LCEOR | -0.223202 | 0.124695 | -1.789986 | 0.0808 |

| | | | |
|---|---|---|---|
| R-squared | 0.622076 | Mean dependent var | 2.688720 |
| Adjusted R-squared | 0.511464 | S.D. dependent var | 0.715272 |
| F-statistic | 5.623958 | Durbin-Watson stat | 1.427204 |
| Prob(F-statistic) | 0.000014 | | |

**Source: Author's Computation (2017)**

Table 4.27b above includes an analysis of the return on equity (ROE) against the other independent variables. According to the results reported, it is concluded that the regression model fits the data and the whole model is statistically significant (R-squared = 0.62, P-Value = 0.01). The independent variables (LBETHN, LCEOT, LCEOA, LINO, LBGDR, and LCEOR) can therefore be relied on to explain 62% of the variations in the ROE. The Adjusted R-Squared is 0.51 which means about 51% of ROE is explained by independent variables in the model after adjusting for error in data. In other words, in Nigerian deposit money banks, ROE is affected by independent variables in the model. F-statistic (5.62; p-value<0.01) and the Durbin Watson (1.43) suggests that the model is of good fit. Based on the results in Table 4.27b, board gender (LBGDR), CEO remuneration (LCEOR), and CEO Tenure (LCEOT) have a negative coefficient and are statistically insignificant, which means they do not appear to influence return on equity (ROE) in Nigerian deposit money banks. On the contrary, CEO age (LCEOA) measured by its natural logarithm is positively correlated to ROE and statistically significant, while insider ownership (LINO) was also statistically significant but negatively correlated, which

means that both LINO and LCEOA appear to influence return on equity (ROE) in Nigerian Deposit money banks in opposite directions. Specifically, a percentage change in LCEOA, and LINO results in 5.27 and -0.22 percentage change in banks performance (ROE) respectively, holding other variables at constant. However, in magnitude and direction it could be observed that variations in LCEOA appears to exhibit the highest effect on banks performance. Detailed analysis of the result further suggests that while LCEOR is significant at 10%, the degree of the responsiveness of the banks performance to the variations in LCEOA is elastic and statistically significant. Thus, all things being equal, a percentage change in LCEOA will result in a greater percentage (5.27%) change in banks performance. Therefore, it could be noted that LINO and LCEOA account for the most significant effect on banks performance (ROE) albeit in opposite direction, and could be regarded to play the most significant role in determining the level of performance of the banks within the scope of the present study.

The predicted ROE result from the model is:

LROE = -9.627859+ 0.278775*LBETHN -0.150373*LCEOT + 5.266132*LCEOA -0.215735*LINO -0.117252*LBGDR -0.223202*LCEOR

**4.4.4.1.3 Testing of Hypothesis 4 (c)**

To test the influence of agency mechanisms (proxied by board gender, board ethnicity, CEO remuneration, CEO tenure, CEO age, and insider ownership) on TBQ (as a proxy for financial performance).

The implicit form of the equation is expressed as:

TBQ = f (BGDR, BETHN, CEOR, CEOT, CEOA, TM) Eq. (8)

The explicit form of the equation could be expressed as:

TBQ = $\beta$0 + $\beta$1 (BGDR)i,t + $\beta$2 (BETHN)i,t + $\beta$3 (CEOR)i,t + $\beta$4 (CEOT)i,t + $\beta$5 (CEOA)i,t + $\beta$6 (TM)i,t + Ui,t Eq. (9)

The log form of the equation therefore becomes:

TBQ =$\beta$0 + $\beta$X1 (LBGDR)i,t + $\beta$X2 (LBETHN)i,t + $\beta$X3 (CEOR)i,t + $\beta$X4 (LCEOT)i,t + $\beta$X5 (LCEOA)i,t + $\beta$X6 (TM)i,t + Ui,t - Eq. (10)

The result is shown below:

**Table 4.27c: Dependent Variable: LTBQ**

| Variable | Coefficient | Std. Error | t-Statistic | Prob. |
|---|---|---|---|---|
| C | 16.65798 | 6.217357 | 2.679271 | 0.0104 |
| LBGDR | -0.398601 | 0.203722 | -1.956592 | 0.0569 |
| LCEOT | 0.042860 | 0.125607 | 0.341223 | 0.7346 |
| LCEOA | -2.121786 | 1.721399 | -1.232594 | 0.2244 |
| LBETHN | 0.867090 | 0.419145 | 2.068714 | 0.0446 |
| LCEOR | -0.564132 | 0.116333 | -4.849269 | 0.0000 |
| TM | 0.024871 | 0.012726 | 1.954379 | 0.0572 |

| | | | |
|---|---|---|---|
| R-squared | 0.777925 | Durbin-Watson stat | 1.859544 |
| Adjusted R-squared | 0.715950 | | |
| F-statistic | 12.55233 | | |
| Prob(F-statistic) | 0.000000 | | |

**Source: Author's Computation (2017)**

This section reports the results of the regression analysis. Table 4.27c above includes an analysis of the Tobin's' Q (TBQ) against the other independent variables. According to the results reported, it is concluded that the regression model fits the data and the whole model is statistically significant (R-squared = 0.78, P-Value = 0.00). The independent variables (LBGDR, LCEOT, LCEOA, LBETHN, LCEOR, and TM) can therefore be relied on to explain 78% of the variations in the TBQ. The Adjusted R-Squared is 0.72 which means about 72% of TBQ is explained by independent variables in the model after adjusting for error (approximately 7.7%) in data. In other words, in Nigerian deposit money banks, TBQ is affected by independent variables in the model. F-statistic (12.55; p-value<0.01) and the Durbin Watson (1.86) individually suggests that the model is of good fit and free from serial auto-correlation effect. Based on the results in Table 4.27c, board gender (LBGDR), and CEO Age (LCEOA) have a negative coefficient and are statistically insignificant, while CEO tenure (LCEOT) and total meeting (TM) have positive coefficient but are also statistically insignificant, which means they all do not appear to influence Tobin's Q (TBQ) in Nigerian Deposit money banks. On the contrary, board ethnicity (LBETHN) measured by its natural logarithm is positively correlated to TBQ and statistically significant, while CEO remuneration (LCEOR) was also statistically significant but negatively correlated, which means that both LBETHN and LCEOR appear to influence Tobin's Q (TBQ) in Nigerian Deposit money banks in opposite directions. Specifically, a percentage change in LBETHN, and LCEOR results in 0.87 and -0.56 percentage change in banks performance (TBQ) respectively, holding other variables at constant. However, in magnitude and direction it could be observed that

variations in LBETHN appears to exhibit the highest effect on banks performance. Detailed analysis of the result further suggests that while LBGDR and TM are significant at 10%, the degree of the responsiveness of the banks performance to the variations in LBETHN is elastic and statistically significant. Thus, all things being equal, a percentage change in LBETHN will result in a greater percentage change in banks performance. Therefore, it could be noted that LBETHN and LCEOR account for the most significant effect on banks performance albeit in opposite direction, and could be regarded to play the most significant role in determining the level of performance of the banks within the scope of the present study.

The predicted TBQ result from the model is:

LTBQ = 16.65798 -0.398601*LBGDR +0.042860*LCEOT -2.121786*LCEOA +0.867090*LBETHN -0.564132 *LCEOR +0.024871*TM

**4.4.4.1.4 Testing of Hypothesis 4 (d)**

To test the influence of agency mechanisms (proxied by board gender, board ethnicity, CEO remuneration, CEO tenure, CEO age, and insider ownership) on NIM (as a proxy for financial performance).

The implicit form of the equation is expressed as:

NIM = f (BGDR, BETHN, CEOR, CEOA, CEOT, INO) Eq. (11)

The explicit form of the equation could be expressed as:

NIM = **β**0 + **β**1 (BGDR)i,t + **β**2 (BETHN)i,t + **β**3 (CEOR)i,t + **β**4 (CEOA)i,t + **β**5 (CEOT)i,t + **β**6 (INO)i,t + Ui,t Eq. (12)

The log form of the equation therefore becomes:

NIM =**β**0 + **β**X1 (LBGDR)i,t + **β**X2 (LBETHN)i,t + **β**X3 (LCEOR)i,t + **β**X4 (LCOA)i,t + **β**X5 (LCEOT)i,t + **β**X6 (LINO)i,t + Ui,t - Eq. (13)

The result is shown below:

**Table 4.27d: Dependent Variable: LNIM**

| Variable | Coefficient | Std. Error | t-Statistic | Prob. |
|---|---|---|---|---|
| C | 7.532228 | 2.224660 | 3.385788 | 0.0015 |
| LBGDR | -0.019889 | 0.077338 | -0.257175 | 0.7983 |
| LCEOT | 0.087528 | 0.047261 | 1.852017 | 0.0709 |
| LCEOA | -0.454666 | 0.683575 | -0.665130 | 0.5095 |
| LINO | -0.004011 | 0.033101 | -0.121179 | 0.9041 |
| LBETHN | 0.108029 | 0.149340 | 0.723377 | 0.4734 |
| LCEOR | -0.188034 | 0.043144 | -4.358288 | 0.0001 |

| | | | |
|---|---|---|---|
| R-squared | 0.658402 | Durbin-Watson stat | 2.156740 |
| Adjusted R-squared | 0.563073 | | |
| F-statistic | 6.906590 | | |
| Prob(F-statistic) | 0.000001 | | |

**Source: Author's Computation (2017)**

Table 4.27d above includes an analysis of the Net Interest Margin (NIM) against the other independent variables. According to the results reported, it is concluded that the regression model fits the data and the whole model is statistically significant (R-squared = 0.66, P-Value = 0.00). The independent variables (LBGDR, LCEOT, LCEOA, LINO, LBETHN, and LCEOR) can therefore be relied on to explain 66% of the variations in the NIM. The Adjusted R-Squared is 0.56 which means about 56% of NIM is explained by independent variables in the model after adjusting for error in data. In other words, in Nigerian deposit money banks, NIM is affected by independent variables in the model. F-statistic (6.91; p-value<0.01) and the Durbin Watson (2.16) individually suggests that the model is of good fit and free from serial auto-correlation effect. Based on the results in Table 4.27d, only CEO remuneration (LCEOR) was statistically significant, and has negative coefficient to influence NIM. Other independent variables such as board gender (LBGDR), CEO age (LCEOA), and insider ownership (LINO) had negative coefficient and are statistically insignificant. Board ethnicity (LBETHN) and CEO tenure (LCEOT) both have positive coefficient but are also statistically insignificant, even though LCEOT was significant at 10% level, which means they all do not appear to influence Net Interest Margin (NIM) in Nigerian Deposit money banks. Specifically, a percentage change in LCEOR results in -0.19 percentage decrease in banks performance, holding other variables at constant.

The predicted NIM result from the model is:

LNIM = 7.532228 -0.019889*LBGDR +0.087528*LCEOT -0.454666*LCEOA -0.004011 *LINO +0.108029*LBETHN -0.188034*LCEOR

#### 4.4.4.1.5 Testing of Hypothesis 4 (e)

To test the influence of agency mechanisms (proxied by board gender, board ethnicity, CEO remuneration, CEO tenure, CEO age, and insider ownership) on NPL (as a proxy for financial performance).

The implicit form of the equation is expressed as:

NPL = f (BGDR, CEOT, CEOR, BETHN, CEOA, INO) Eq. (14)

The explicit form of the equation could be expressed as:

NPL = $\beta$0 + $\beta$1 (BGDR)i,t + $\beta$2 (CEOT)i,t + $\beta$3 (CEOR)i,t + $\beta$4 (BETHN)i,t + $\beta$5 (CEOA)i,t + $\beta$6 (INO)i,t + Ui,t Eq. (15)

The log form of the equation therefore becomes:

NPL =$\beta$0 + $\beta$X1 (LBGDR)i,t + $\beta$X2 (LCEOT)i,t + $\beta$X3 (LCEOR)i,t + $\beta$X4 (LBETHN)i,t + $\beta$X5 (LCEOA)i,t + $\beta$X6 (LINO)i,t + Ui,t - Eq. (16)

The result of the test is shown below:

**Table 4.27e: Dependent Variable: LNPL**

| Variable | Coefficient | Std. Error | t-Statistic | Prob. |
|---|---|---|---|---|
| C | 39.71337 | 9.203233 | 4.315154 | 0.0001 |
| LBETHN | -0.713733 | 0.617808 | -1.155265 | 0.2544 |
| LCEOT | 0.245957 | 0.195514 | 1.258006 | 0.2152 |
| LCEOA | -7.896647 | 2.827891 | -2.792416 | 0.0078 |
| LINO | 0.154127 | 0.136937 | 1.125534 | 0.2666 |
| LBGDR | 0.591347 | 0.319940 | 1.848304 | 0.0714 |
| LCEOR | 0.656598 | 0.178483 | 3.678765 | 0.0006 |

| | | | |
|---|---|---|---|
| R-squared | 0.514821 | Durbin-Watson stat | 1.492434 |
| Adjusted R-squared | 0.379422 | | |
| F-statistic | 3.802255 | | |
| Prob(F-statistic) | 0.000581 | | |

**Source: Author's Computation (2017)**

Table 4.27e above includes an analysis of the Non-Performing Loan (NPL) against the other independent variables. According to the results reported, it is concluded that the regression model fits the data and the whole model is statistically significant (R-squared = 0.51, P-Value = 0.00). The

independent variables (LBETHN, LCEOT, LCEOA, LINO, LBGDR, and LCEOR) can therefore be relied on to explain 51% of the variations in the NPL. The Adjusted R-Squared is 0.379 which means about 38% of NPL is explained by independent variables in the model after adjusting for error in data. In other words, in Nigerian deposit money banks, NPL is affected by independent variables in the model. F-statistic (3.80; p-value<0.01) suggests that the model is of good fit. Based on the results in Table 4.27e, CEO age (LCEOA) and CEO remuneration (LCEOR) were statistically significant, and both have contrasting effect on NPL. Board ethnicity (LBETHN) has a negative coefficient but insignificant effect, whilst Board gender (LBGDR), CEO tenure (LCEOT) and insider ownership (LINO) both have positive coefficient but also statistically insignificant, which means they all do not appear to influence non-performing loan (NPL) in Nigerian Deposit money banks. Specifically, a percentage change in LCEOA and LCEOR result in -7.9 and 0.66 percentage decrease and increase respectively in banks performance (NPL), holding other variables at constant.

The predicted NPL result from the model is:

LNPLR = 39.71337 -0.713733*LBETHN +0.245957*LCEOT -7.896647*LCEOA +0.154127*LINO +0.591347*LBGDR +0.656598*LCEOR

**Summary of Research Results**

This section has examined the main determinants of financial performance in Nigerian Deposit Money Banks. The first step was a panel data prepared with the inclusion of 88 firm-year observation. The important descriptive statistics of financial performance and other variables are provided in table 4.24. After testing multicollinearity (table 4.26), the results showed that there is no multicollinearity problem among the independent variables. The next step was to determine the appropriate model effects to choose, for which the Hausman test was performed. The Hausman test indicated that the fixed effects model is preferred (see appendix “D” for the full results). The panel regression analysis (table 4.27a to 4.27e) was used to test the hypothesis of the study. According to the results, four of the seven independent variables had significant influence on financial performance. In specific terms, ROA (was positively affected by board ethnicity-LBETHN, and the CEO age-LCEOA); ROE (was positively affected by the CEO age -LCEOA, but negatively influenced by insider ownership-LINO), TBQ (was positively influenced by board ethnicity-LBETHN, but negatively affected by CEO remuneration-LCEOR), NIM (was negatively influenced by CEO’s remuneration-LCEOR), and NPL (was improved by the CEO age-LCEOA, but worsened by the CEO remuneration -LCEOR). Of the other three

independent variables, when tested at 10% significant level, CEO tenure-LCEOT had a positive coefficient and statistical significance in influencing NIM; Total meeting frequency-TM had positive coefficient on TBQ, while board gender (LBGDR) negatively influenced TBQ, and worsened NPL. It should be noted that across all the five measures of financial performance, board gender had worsening effect and was not statistically significant at 5% level.

The hypothesis initially posed is thus confirmed after carrying out the statistical analysis, hence we can affirm that agency mechanisms influence financial performance as measured by ROA, ROE, TBQ, NIM and NPL.

**Table 4.28 Summary of Significance Tests on Financial Performance**

| | | Model 1 | Model 2 | Model 3 | Model 4 | Model 5 |
|---|---|---|---|---|---|---|
| | ***a priori*** | ROA | ROE | TBQ | NIM | NPL |
| LBETHN | - | +** | + | +** | + | - |
| LBDGR | + | - | - | -* | - | +* |
| LCEOT | +/- | - | - | + | +* | + |
| LCEOA | +/- | +** | +** | - | - | +*** |
| LCEOR | + | n | -* | -*** | -*** | -*** |
| TM | - | - | n | +* | n | n |
| LINO | + | -* | -** | n | - | + |

**Source: Author's Computation (2017)**

***** Significant at 0.01, **Significant at 0.05, * Significant at 0.10. + (positive relationship), – (negative relationship), n – not tested in the model*

Table 4.28 above shows the apriori expectation and the results of the tests performed.

### 4.4.5 Test of Hypothesis Five

Hypothesis five was formulated based on the following premise of research question and research objective

**Research Objective 5:**

To determine the role of bank characteristics in moderating the influence of agency mechanisms on financial performance

**Research Question 5:**

To what extent do bank characteristics moderate the influence of agency mechanisms on financial performance?

Based on the above research question and objective, hypothesis five was stated in both the null and alternate forms as below:

**Research Hypothesis Five**

$H_0$: Bank characteristics do not have significant influence in moderating the relationship between agency mechanisms and financial performance.

Ha: Bank characteristics have significant influence in moderating the relationship between agency mechanisms and financial performance.

Research objective 5 is an extension of research objective 4, as it seeks to determine the moderating role of bank characteristics in influencing the relationship between agency mechanisms and financial performance (tested in hypothesis 4).

In the process of carrying out the analysis, the moderating effect of the bank characteristics were tested. In this process three characteristics bank leverage, bank size measured by total assets, and bank age (LFLV, LTA and LFA respectively), were included in the model in addition to three other agency mechanisms indicators and their joint influence on the model determined. The basic idea was to evaluate the extent of the moderating effect of the bank characteristic variables in explaining the variations in the phenomenon under investigation and also in the overall significance of the estimated model. Consequently, the individual effect of the variables was considered in analysing the variations in the explained variable.

**Table 4.29: Descriptive Statistics Bank Characteristics**

| | Mean | Maximum | Minimum | Std. Dev. | # Obs |
|---|---|---|---|---|---|
| **Control Variables** | | | | | |
| FA (Bank Age) | 38.00 | 122.00 | 16.00 | 31.59 | 88 |
| FLV (Bank Leverage) | 7.12 | 18.10 | 3.33 | 2.36 | 88 |
| TA (Bank Size –Total Assets) | 1.63tri | 4.74 tri | 107bn | 1.15tri | 88 |

**Source: Author's Computation (2017)**

From table 4.29 above, descriptive statistics are provided per unit in absolute numbers for the variables: firm age (FA), and total assets (TA), while values are expressed in percentages for Firm leverage (FLV). The table shows eighty-eight observations, representing data for eleven years (2006-2016) for each of the eight sampled banks.

To investigate the moderating effects of bank characteristics on the influence of agency mechanisms on financial performance of Nigerian deposit money banks, this study used the following dynamic panel regression model:

$FINPERF_{it}$ = β0it + β1lnBGDRit + β2lnBETHNit + β3lnCEORit + β4TMit + β5lnCEOTit + β6lnCEOAit + β7lnINOit + β8lnTAit + β9lnFAit + β10lnFLVit + Uit Eq. (17)

In this model, the dependent variable is bank's financial performance (FINPERF) measured by return on assets (ROA), return on equity (ROE), Tobin's Q (Q), Net Interest Margin (NIM) and Non-Performing Loan (NPL). The independent variables consist of seven (7) agency mechanism variables, namely Board gender (in logarithm) (LBGDR), Board Ethnic composition (in logarithm) (LBETHN), CEO's remuneration (in logarithm) (LCEOR), Board's intensity proxied by total meetings of the board and the committees (TM), CEO's tenure (in logarithm) (LCEOT), CEO's age (in logarithm) (LCEOA), and Insider Ownership (in logarithm) (LINO), while the moderating/control variables are: firm age (LFA), firm size proxied by total assets (LTA), and Firm leverage (LFLV), Uit is the random error term and subscripts i and t represent firm and time period, respectively.

#### 4.4.5.1.1 Testing of Hypothesis 5 (a)

To test the moderating effect of bank characteristics on the influence of agency mechanisms on ROA (as a proxy for financial performance).

The implicit form of the equation is expressed as:

ROA = *f* (BETHN, CEOA, BGDR, FA, FLV, TA) ….. Eq (18)

The explicit form of the equation could be expressed as:

ROA = **β**0 + **β**1 (BETHN)it + **β**2 (CEOA)it + **β**3 (BGDR)it + **β**4 (FA)it + **β**5 (FLV)it + **β**6 (TA)it + Uit ……. Eq (19)

The log form of the equation therefore becomes:

ROA = 0 + **β**1 (LBETHN)it + **β**2 (LCEOA)it + **β**3 (LBGDR)it + **β**4 (LFA)it + **β**5 (LFLV)it + **β**6 (LTA)it + Uit Eq (20)

The result is shown below:

**Table 4.30a: Dependent Variable: LROA**

| Variable | Coefficient | Std. Error | t-Statistic | Prob. |
|---|---|---|---|---|
| LBETHN | 0.945746 | 0.399294 | 2.368546 | 0.0227 |
| LCEOA | 1.633195 | 1.574207 | 1.037471 | 0.3056 |
| LFA | 1.935839 | 1.475138 | 1.312310 | 0.1967 |
| LFLV | 0.093287 | 0.352940 | 0.264313 | 0.7929 |
| LTA | -0.326922 | 0.280985 | -1.163485 | 0.2514 |
| LBGDR | -0.314718 | 0.258723 | -1.216430 | 0.2308 |
| C | -4.743811 | 7.428377 | -0.638607 | 0.5266 |
| R-squared | 0.670800 | Durbin-Watson stat | | 1.721490 |
| Adjusted R-squared | 0.574448 | | | |
| F-statistic | 6.962017 | | | |
| Prob(F-statistic) | 0.000001 | | | |

**Source: Author's Computation (2017)**

The result of the moderating effect of the bank characteristics were examined with introduction of the variables firm age- LFA, firm size (as total assets) - LTA, and firm leverage - LFLV into the model with other internal governance indicators (LBETHN, LCEOA and LBGDR) as explanatory variables while firm performance was proxied by LROA. Given the unbalanced nature of the panel study which consists of eight cross sectional observation over a time period of 11 years, the model could only accommodate maximum of seven variables in its random effect estimation. The evidence from the table 4.27a (in hypothesis 4) and table 4.30a (in hypothesis 5) shows a slight decrease in the joint significance of the exogenous variables in explaining the model as shown in the reduced R-squared result (from 0.68 to 0.67). Further analysis of the moderating effect model also shows a higher value of the Durbin Watson statistic (from 1.69 to 1.72) suggesting a decreasing influence of autocorrelation though not significant. The firm characteristics - LFA LTA and LFLV individually and jointly indicate an insignificant effect in explaining the variations in bank performance (ROA), as evidenced by the model showing a decrease in F-statistic (7.41 to 6.96). Hence, specifically evidence from the result implies that the firm characteristics variables (LFLV and LFA) exhibit positive but insignificant effect, while LTA exhibits negative and insignificant effect on bank performance (ROA).

#### 4.4.5.1.2 Testing of Hypothesis 5(b)

To test the moderating effect of bank characteristics on the influence of agency mechanisms on ROE (as a proxy for financial performance).

The implicit form of the equation is expressed as:

ROE = *f* (BGDR, BETHN, CEOA, TA, FA, FLV) …….Eq (21)

The explicit form of the equation could be expressed as:

ROE = $\beta 0 + \beta 1$ (BGDR)it + $\beta 2$ (BETHN)it + $\beta 3$ (CEOA)it + $\beta 4$ (TA)it + $\beta 5$(FA)it + $\beta 6$ (FLV) it Uit …..Eq (22)

The log form of the equation therefore becomes:

ROE = $\beta 0 + \beta 1$ (LBGDR)i,t + $\beta 2$ (LBETHN)i,t + $\beta 3$ (LCEOA)i,t + $\beta 4$ (LTA)it + $\beta 5$(LFA)it + $\beta 6$ (LFLV) it Ui,t …(Eq 23)

The result is shown below:

**Table 4.30b: Dependent Variable: LROE**

| Variable | Coefficient | Std. Error | t-Statistic | Prob. |
|---|---|---|---|---|
| LBETHN | 0.938966 | 0.399050 | 2.353005 | 0.0235 |
| LCEOA | 1.598109 | 1.573244 | 1.015805 | 0.3157 |
| LFA | 1.925473 | 1.474236 | 1.306082 | 0.1988 |
| LFLV | 1.092057 | 0.352724 | 3.096067 | 0.0035 |
| LTA | -0.324977 | 0.280814 | -1.157268 | 0.2539 |
| LBGDR | -0.316882 | 0.258564 | -1.225543 | 0.2274 |
| C | -4.600717 | 7.423832 | -0.619723 | 0.5389 |

| | | | |
|---|---|---|---|
| R-squared | 0.670955 | Durbin-Watson stat | 1.720164 |
| Adjusted R-squared | 0.574650 | | |
| F-statistic | 6.966927 | | |
| Prob(F-statistic) | 0.000001 | | |

**Source: Author's Computation (2017)**

The evidence from the table 4.27b and 4.30b shows a slight increase in the joint significance of the exogenous variables in explaining the model as shown in the increased R-squared result (from 0.62 to 0.67). Further analysis of the moderating effect model also shows a higher value of the Durbin Watson statistic (from 1.43 to 1.72) suggesting a significant decreasing influence of autocorrelation.

While LFA, LCEOA, LTA, and LBGDR appear to be less significant in explanation of the variations in bank performance, LBETHN and LFLV indicate a significant effect with LFV having a direct effect on performance. Therefore, it could be observed that there is an increase in the joint explanatory power of the model with a reduced tendency for the presence of autocorrelation that accompanied the moderating effect. Furthermore, with the significant positive contribution of the (LFLV), the statistical significance of the model also increased as evidenced from the F-statistic (5.62 to 6.97). Hence, specific

evidence from the result implies that the bank characteristic variable (LFLV) exhibits both direct and indirect effect on bank performance (ROE).

#### 4.4.5.1.3 Testing of Hypothesis 5(c )

To test the moderating effect of bank characteristics on the influence of agency mechanisms on TBQ (as a proxy for financial performance).

The implicit form of the equation is expressed as:

TBQ = *f* (BETHN, CEOA, CEOR, TA, FA, FLV) ……Eq (24)

The explicit form of the equation could be expressed as:

TBQ = $\beta$0 + $\beta$1 (BETHN)i,t + $\beta$2 (CEOA)i,t + $\beta$3 (CEOR)i,t + $\beta$4 (TA)i,t + $\beta$5 (FA)i,t + $\beta$6 (FLV)i,t Ui,t …….Eq (25)

The log form of the equation therefore becomes:

TBQ = $\beta$0 + $\beta$1 (LBETHN)i,t + $\beta$2 (LCEOA)i,t + $\beta$3 (LCEOR)i,t + $\beta$4 (LTA)i,t + $\beta$5 (LFA)i,t + $\beta$6 (LFLV)i,t Ui,t … (Eq 26)

The result is shown below:

**Table 4.30c: Dependent Variable: LTBQ**

| Variable | Coefficient | Std. Error | t-Statistic | Prob. |
|---|---|---|---|---|
| LBETHN | 0.375495 | 0.238462 | 1.574656 | 0.1196 |
| LCEOA | -2.308693 | 0.748959 | -3.082537 | 0.0029 |
| LFA | -0.992260 | 1.007092 | -0.985272 | 0.3277 |
| LTA | -0.590052 | 0.200404 | -2.944316 | 0.0043 |
| LFLV | 0.654961 | 0.236973 | 2.763864 | 0.0072 |
| LCEOR | -0.095866 | 0.108403 | -0.884348 | 0.3794 |
| C | 28.32824 | 3.309670 | 8.559235 | 0.0000 |

| | | | |
|---|---|---|---|
| R-squared | 0.739169 | Durbin-Watson stat | 1.653715 |
| Adjusted R-squared | 0.693347 | | |
| F-statistic | 16.13140 | | |
| Prob(F-statistic) | 0.000000 | | |

**Source: Author's Computation (2017)**

The evidence from the table 4.27c and 4.30c shows a slight decrease in the joint significance of the exogenous variables in explaining the model as shown in the reduced R-squared result (from 0.78 to 0.74). Further analysis of the moderating effect model also shows a lower value of the Durbin Watson statistic (from 1.86 to 1.65) suggesting an increasing influence of autocorrelation though not significant. While LFA LBETHN and LCEOR appear to be less significant in explanation of the variations in bank

performance LCEOA, LTA and LFLV indicate a significant effect with LFLV having a direct positive effect on performance. Therefore, it could be observed that there is a reduction in the joint explanatory power of the model with increased tendency for the presence of autocorrelation that accompanied the moderating effect. Conversely, with the significant positive (LFLV) and negative (LTA) contribution of the two moderating variables the statistical significance of the model also increased as evidenced from the F-statistic (12.55 to 16.131). Hence, specific evidence from the result implies that the bank characteristics variables (LFLV and LTA) exhibit positive and negative direct effect respectively on bank performance (TBQ).

#### 4.4.5.1.4 Testing of Hypothesis 5 (d)

To test the moderating effect of bank characteristics on the influence of agency mechanisms on NIM (as a proxy for financial performance).

The implicit form of the equation is expressed as:

NIM = *f* (BGDR, BETHN, CEOR, FA, TA, FLV) …….Eq (27)

The explicit form of the equation could be expressed as:

NIM = **β**0 + **β**1 (BGDR)i,t + **β**2 (BETHN)i,t + **β**3 (CEOR)i,t + **β**4 (FA)i,t + **β**5 (TA)i,t + **β**6 (FLV)i,t Ui,t ……Eq (28)

The log form of the equation therefore becomes:

NIM = **β**0 + **β**1 (LBGDR)i,t + **β**2 (LBETHN)i,t + **β**3 (LCEOR)i,t + **β**4 (LFA)i,t + **β**5 (LTA)i,t + **β**6 (LFLV)i,t Ui,t …(Eq 29)

The result is shown below:

**Table 4.30d: Dependent Variable: LNIM**

| Variable | Coefficient | Std. Error | t-Statistic | Prob. |
|---|---|---|---|---|
| LBETHN | 0.111478 | 0.156768 | 0.711099 | 0.4809 |
| LBGDR | -0.014023 | 0.095280 | -0.147181 | 0.8837 |
| LFA | -0.003478 | 0.565681 | -0.006148 | 0.9951 |
| LTA | -0.061964 | 0.125595 | -0.493361 | 0.6243 |
| LFLV | -0.032626 | 0.130031 | -0.250906 | 0.8031 |
| LCEOR | -0.185382 | 0.058007 | -3.195858 | 0.0026 |
| C | 7.524686 | 2.178604 | 3.453903 | 0.0013 |

| | | | |
|---|---|---|---|
| R-squared | 0.631665 | Mean dependent var | 2.603225 |
| Adjusted R-squared | 0.528874 | S.D. dependent var | 0.274327 |
| F-statistic | 6.145127 | Durbin-Watson stat | 2.191827 |
| Prob(F-statistic) | 0.000004 | | |

**Source**: **Author's Computation (2017)**

The result of the moderating effect of the bank characteristics were examined with introduction of the variables bank age- LFA, bank size (as total assets) - LTA, and bank leverage - LFLV into the model with other agency mechanism indicators (LBETHN, LCEOR and LBGDR) as explanatory variables while firm performance was proxied by LNIM. The evidence from the table 4.27d and 4.30d shows a slight decrease in the joint significance of the exogenous variables in explaining the model as shown in the reduced R-squared result (from 0.66 to 0.63). Further analysis of the moderating effect model also shows a slightly higher value of the Durbin Watson statistic (from 2.16 to 2.19) both values considered appropriate with regards to influence of autocorrelation. The bank characteristics - LFA LTA and LFLV individually and jointly indicate an insignificant effect in explaining the variations in bank performance (NIM), as evidenced by the model showing a decrease in F-statistic (6.91 to 6.14). Hence, specific evidence from the result implies that the bank characteristics variables (LFLV, LTA, and LFA) exhibit negative and insignificant effect on bank performance (NIM).

#### 4.4.5.1.5 Testing of Hypothesis 5 (e)

To test the moderating effect of bank characteristics on the influence of agency mechanisms on NPL (as a proxy for financial performance).

The implicit form of the equation is expressed as:

NPL = *f* (INO, BETHN, CEOA, FA, TA, FLV) …….Eq (30)

The explicit form of the equation could be expressed as:

NPL = $\boldsymbol{\beta}0 + \boldsymbol{\beta}1$ (INO)i,t + $\boldsymbol{\beta}2$ (BETHN)i,t + $\boldsymbol{\beta}3$ (CEOA)i,t + $\boldsymbol{\beta}4$ (FA)i,t + $\boldsymbol{\beta}5$ (TA)i,t + $\boldsymbol{\beta}6$ (FLV)i,t ..……..Eq (31)

The log form of the equation therefore becomes:

NPL = $\boldsymbol{\beta}0 + \boldsymbol{\beta}1$ (LINO)i,t + $\boldsymbol{\beta}2$ (LBETHN)i,t + $\boldsymbol{\beta}3$ (LCEOA)i,t + $\boldsymbol{\beta}4$ (LFA)i,t + $\boldsymbol{\beta}5$ (LTA)i,t + $\boldsymbol{\beta}6$ (LFLV)i,t ……..… (Eq 32)

The result is shown below:

**Table 4.30e: Dependent Variable: LNPL**

| Variable | Coefficient | Std. Error | t-Statistic | Prob. |
|---|---|---|---|---|
| LFA | -0.260733 | 2.208982 | -0.118033 | 0.9066 |
| LFLV | -0.914857 | 0.474379 | -1.928536 | 0.0604 |
| LCEOA | -4.765713 | 2.376934 | -2.004984 | 0.0513 |
| LTA | 0.922207 | 0.466148 | 1.978358 | 0.0543 |
| LBGDR | 0.034931 | 0.349921 | 0.099826 | 0.9209 |
| LCEOR | 0.148928 | 0.232846 | 0.639601 | 0.5258 |
| C | 16.73560 | 11.36712 | 1.472281 | 0.1482 |

| | | | |
|---|---|---|---|
| R-squared | 0.556835 | Durbin-Watson stat | 1.469393 |
| Adjusted R-squared | 0.433162 | | |
| F-statistic | 4.502452 | | |
| Prob(F-statistic) | 0.000118 | | |

**Source: Author's Computation (2017)**

The evidence from the table 4.27e and 4.30e shows an increase in the joint significance of the exogenous variables in explaining the model as shown in the increased R-squared result (from 0.51 to 0.56). Further analysis of the moderating effect model also shows a lower value of the Durbin Watson statistic (from 1.49 to 1.47) suggesting an increasing influence of autocorrelation though not significant within the context of the two results, the statistical significance of the model also increased as evidenced from the F-statistic (3.80 to 4.50). The introduction of the moderating variables (LFA, LFLV, and LTA) only show significance at 10% level. LCEOA appears to be less significant in explanation of the variations in bank performance (NPL). Evidence from the result implies that the bank characteristics variables (LFA, LFLV and LTA) do not appear to exhibit direct effect on bank performance (NPL), though LFLV, and LTA were significant at 10%.

**Summary of Research Results**

This section has examined the moderating effects of bank characteristics – bank age (LFA), bank leverage (LFLV), and bank size- measured by total assets (LTA) on the influence of agency mechanisms on financial performance. According to the results, two of the three moderating variables had significant influence on financial performance. In specific terms, LFLV positively and significantly influenced LROE, and LTBQ, while LTA negatively and significantly influenced LTBQ. Bank leverage (FLV) and Bank size (LTA) have contrasting influence on NLPL at 10% significance level.

**Decision Rule:** Reject the Null hypothesis, when the significance value is below 0.05. Do not reject hypothesis, when significance value is greater than 0.05.

**Decision:** Reject the null hypothesis. Therefore, there is a significant effect of bank characteristics (LTA and LFLV) on the influence of agency mechanisms on financial performance.

The hypothesis initially posed is thus confirmed after carrying out the statistical analysis, hence we can affirm that bank characteristics moderate the influence of agency mechanisms on financial performance as measured by LTA and LFLV.

The main results (as shown in Table 4.30a-e) are:

**Table 4.31: Summary of Significance Tests-Bank Characteristics**

| | | Model 1 | Model 2 | Model 3 | Model 4 | Model 5 |
|---|---|---|---|---|---|---|
| | *a priori* | ROA | ROE | TBQ | NIM | NPL |
| LFA | - | + | + | - | - | - |
| LTA | + | - | - | -*** | - | +* |
| LFLV | + | + | +*** | +*** | - | +* |

**Source: Author's Computation (2017)**

**** Significant at 0.01, **Significant at 0.05, * Significant at 0.10. + (positive relationship), – (negative relationship), n – not tested in the model*

### 4.4.6 Test of Hypothesis Six

Hypothesis six was formulated based on the following premise of research question and research objective

**Research Objective 6:**

To evaluate the impact of corporate governance practices disclosure on banks' sustainability performance reporting.

**Research Question 6:**

To what extent does corporate governance practices disclosure enhance sustainability performance reporting by banks?

Based on the above research question and objective, hypothesis six was stated in both the null and alternate forms as below:

**Research Hypothesis Six**

$H_0$: Corporate governance practices disclosure does not have significant effect on sustainability performance reporting.

Ha: Corporate governance practices disclosure has significant effect on sustainability performance reporting.

**Steps 1, 2 and 3:**

The study carried out a descriptive statistics analysis, then identified whether or not there is a relationship, and examined the degree of the relationship between corporate governance practices disclosure and sustainability reporting using Pearson Product-Moment Correlation Coefficient.

The reliability of the corporate governance index and sustainability index was tested by Cronbach alpha, with the result showing 0.842 which is above the recommended threshold.

### 4.4.6.1 Descriptive Statistics

The table 4.32 below shows the descriptive statistics on the composite index used to measure the independent variable (corporate governance practices disclosure) and the dependent variable (sustainability performance reporting).

**Table 4.32: Descriptive Statistics on Governance and Sustainability Index**

| Item | N | MAX SCORE | Minimum | Maximum | Mean | Std. Deviation |
|---|---|---|---|---|---|---|
| IND | 32 | 6 | 4.00 | 6.00 | 4.9063 | .53033 |
| COM | 32 | 13 | 6.00 | 10.00 | 8.1406 | 1.17935 |
| BOD_OPS | 32 | 24 | 11.00 | 22.00 | 16.1875 | 3.04204 |
| DISC | 32 | 24 | 16.00 | 21.00 | 17.9063 | 1.57315 |
| RSK_MGT | 32 | 33 | 9.00 | 23.00 | 15.5000 | 3.86047 |
| TRA_INT | 32 | 12 | 4.00 | 12.00 | 8.7656 | 2.58714 |
| OWN | 32 | 16 | 9.00 | 15.00 | 13.8125 | 1.22967 |
| | | | | | | |
| FIN_RAT | 32 | 38 | 16.00 | 35.00 | 23.9375 | 5.25441 |
| FIN_INC | 32 | 8 | 2.00 | 8.00 | 5.3438 | 1.38213 |
| DIV | 32 | 25 | 5.00 | 22.00 | 10.0000 | 3.80153 |
| ETH | 32 | 12 | 4.00 | 10.00 | 5.9687 | 1.46979 |
| COMM | 32 | 16 | 2.00 | 13.00 | 7.5000 | 2.55267 |
| HUM_RT | 32 | 7 | 2.00 | 7.00 | 4.5625 | 1.18967 |
| EMP_LAB | 32 | 22 | 5.00 | 18.00 | 9.3750 | 3.66104 |
| PROD_RESP | 32 | 9 | 1.00 | 9.00 | 4.9062 | 1.71068 |
| ENV | 32 | 31 | 4.00 | 24.00 | 12.2813 | 4.69890 |

**Source: Author's Computation (2017)**

Table 4.32 above shows the performance of the banks on both the corporate governance practice disclosure (depicted from the IND row to OWN row), and the sustainability performance reporting (depicted from the FIN_RAT row to ENV row). The “n” column represents observations for 4 years (2013-2016) for each of the eight sampled banks, the “max score” shows the maximum score assigned to each evaluation category. The minimum, maximum, mean and standard deviation scores are statistics generated from SPSS.

**Interpretation:**

While the “maximum” compared to “max score” column suggests that the banks performed well in all categories, the mean scores shows that the maximum obtained by individual bank conceals the true general performance, as it apparently shows that a bank may be strong in one category and be woeful in another category, thus masking the true performance. From the mean scores, the banks performed below average in one category under corporate governance practices (the risk management - RISK_MGT), and in five categories under sustainability performance (diversity, ethics, community support, employment/labour, and environmental practices).

To determine the level of corporate governance practices disclosure, and the sustainability performance reporting of the eight selected banks over the periods 2013 and 2016, a trend analysis was carried out based on total scores obtained. Figure 4.2 below shows the performance trend:

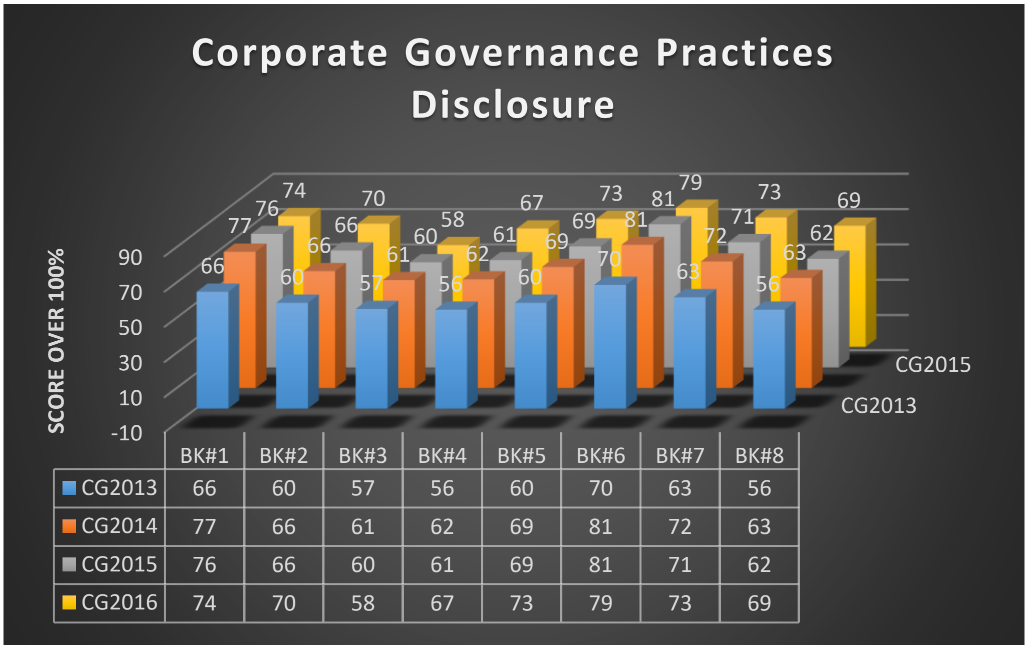


**Figure: 4.2: Corporate Governance Practices Disclosure**
**Source: Author’s Computation (2017)**

From the chart above, all the banks scored above 50% between 2013 and 2016. In addition, the performance revealed a general upward trend year on year for all the banks between 2013 and 2014, while there was a drop between 2015 and 2016 for BK#1, 3, and 6. For all the four years under review, BK#6 was consistently the leader. BK#3 lagged behind in all the years except in 2013 albeit marginally. Overall the chart shows a consistently improving performance by the selected banks in their corporate governance practices disclosure.

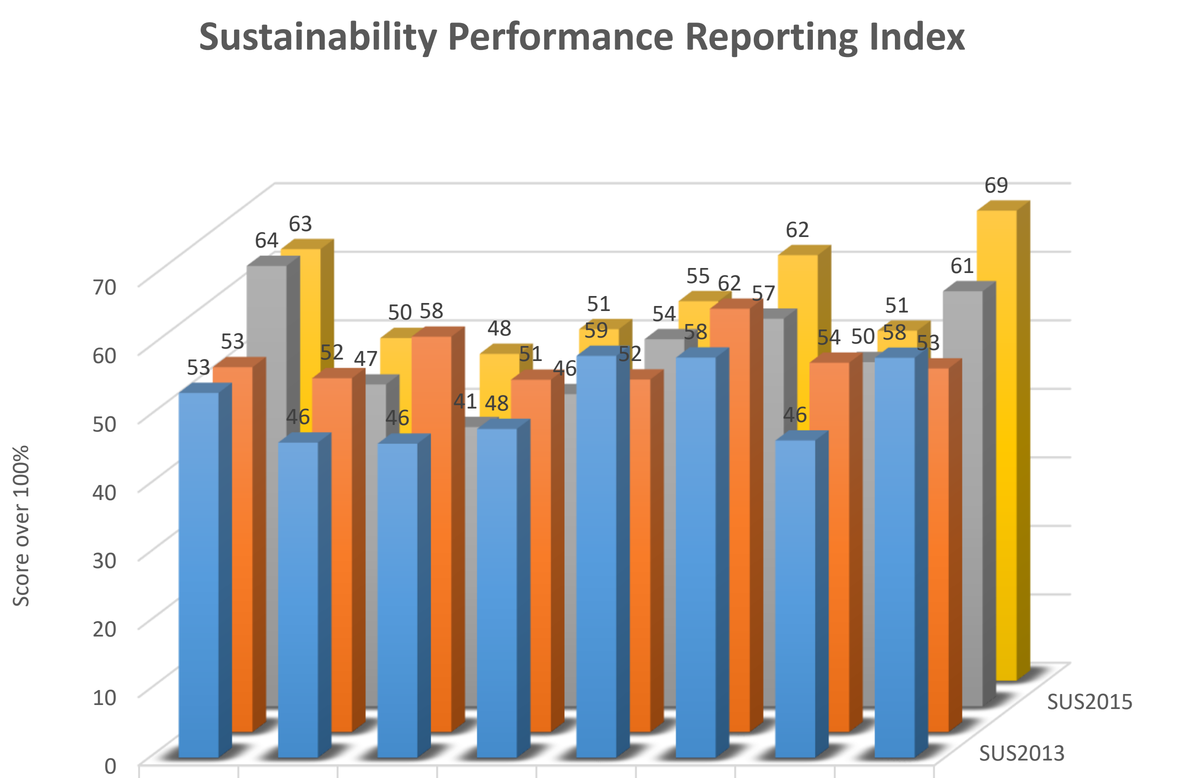


| | BK#1 | BK#2 | BK#3 | BK#4 | BK#5 | BK#6 | BK#7 | BK#8 |
|---|---|---|---|---|---|---|---|---|
| SUS2013 | 53 | 46 | 46 | 48 | 59 | 58 | 46 | 58 |
| SUS2014 | 53 | 52 | 58 | 51 | 52 | 62 | 54 | 53 |
| SUS2015 | 64 | 47 | 41 | 46 | 54 | 57 | 50 | 61 |
| SUS2016 | 63 | 50 | 48 | 51 | 55 | 62 | 51 | 69 |

**Figure: 4.3: Sustainability Performance Reporting Index**
**Source: Author's Computation (2017)**

From the chart above, there is an inconsistent performance by banks on sustainability. Only BK#1, BK#5, BK#6, and BK#8 recorded above fifty percent consistently over the periods 2013-2016. Using

2016 as the benchmark, and MSCI sustainability rating guide (MSCI, 2017), three (3) banks – BK#1, 6, and 8 emerged as leaders (with scores above 60%), three (3) banks – BK#4, 5, and 7 (with scores between 51 and 59%) as average, while BK#2 and 3 are laggards (with scores below 51%)

To gain a better understanding of the inconsistent performance, this study deconstructed sustainability performance scores into its sub-indices - economic, social, and environmental. Fig. 4.4a to Fig. 4.4c below show the results:

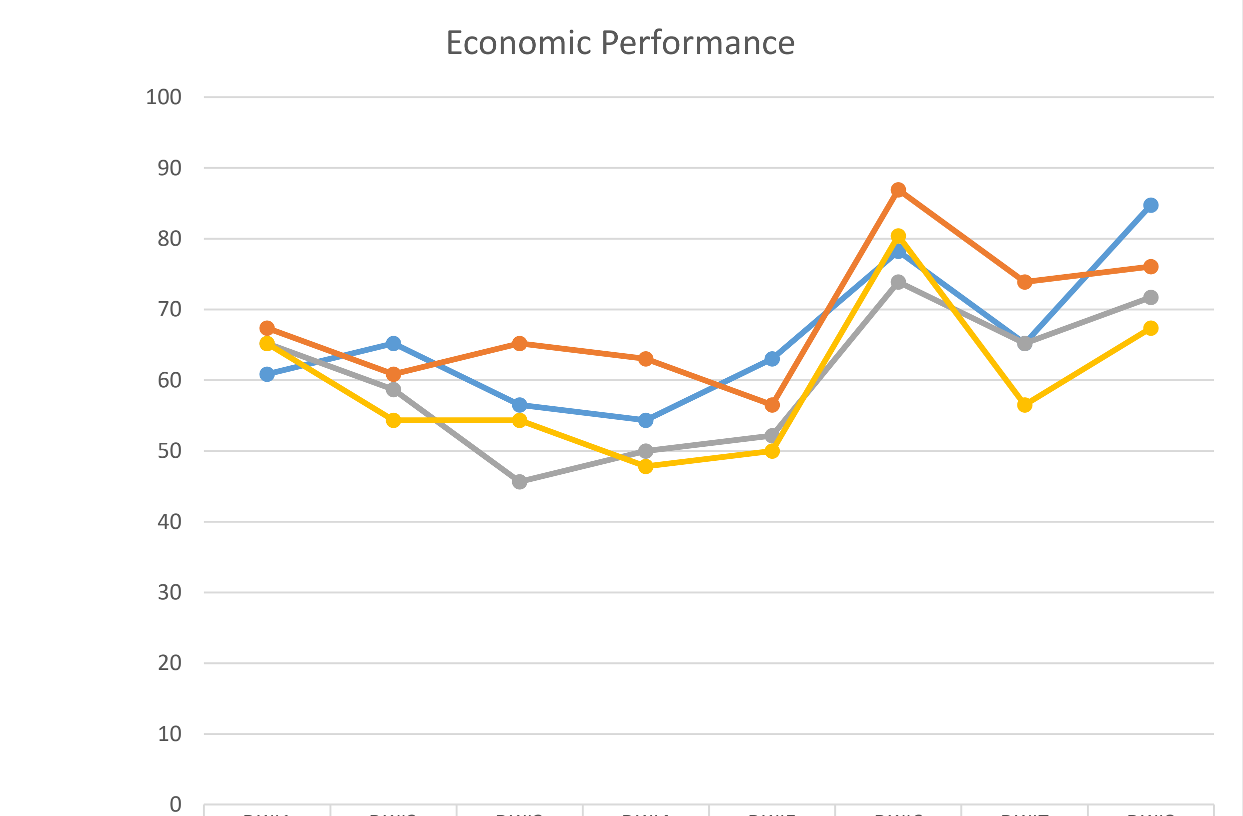


| | BK#1 | BK#2 | BK#3 | BK#4 | BK#5 | BK#6 | BK#7 | BK#8 |
|---|---|---|---|---|---|---|---|---|
| ECO2013 | 61 | 65 | 57 | 54 | 63 | 78 | 65 | 85 |
| ECO2014 | 67 | 61 | 65 | 63 | 57 | 87 | 74 | 76 |
| ECO2015 | 65 | 59 | 46 | 50 | 52 | 74 | 65 | 72 |
| ECO2016 | 65 | 54 | 54 | 48 | 50 | 80 | 57 | 67 |

**Fig: 4.4a: Economic Performance**
**Source: Author's Computation (2017)**

From the chart above, all the banks scored above 50% in economic performance (except BK#3 and BK#4 in 2015 and 2016 respectively). The two best performing banks are BK#6 and BK#8, with the chart showing a switch in position between the two banks from first to second over the four-year period.

Fig. 4.4a reveals a consistently strong performance of banks, with a minimum score of 46%, and a maximum score of 80%.

Economic performance was considered from financial performance and financial innovation perspectives. On financial performance, only BK#6 had a TBQ ratio above 1, meaning it's the only bank earning a rate higher than its replacement cost, which implies that the bank is trading overvalued. This is good for the shareholders as the contrary makes the bank attractive to corporate raiders or potential purchasers seeking to purchase the bank. BK#6 was also the leader among the selected banks on profitability ratios of ROE, ROA, and Net Interest Margin. In addition, it showed leadership status on cost-to-income ratio operating at a rate below fifty percent (50%) i.e. spending less than fifty (50) kobo in every one naira (₦1) on operating expenses. BK#1 and BK#3 had the best earnings growth ratio at a rate above twenty percent (20%). Most of the banks had poor record on non-performing loans, except for BK#1 with a commendable non-performing loan ratio below two percent (2%). All the banks however maintained a good leverage ratio.

On financial inclusion and financial innovation measure, key initiatives observed from this study include financial literacy programmes, provision of financial grants and capacity development trainings, financing and capacity building for SMEs, agent transactions, account opening with zero opening balance and zero minimum account balance, and financial advisory services. All the banks introduced new products and services as well as ICT initiatives such as using a short code on mobile phones to deposit, withdraw, transfer funds, pay bills and buy airtime. Other financial innovations include the introduction of PoS, mPoS, Webpay, mCash, mobile app, internet banking, automated-payroll for clients to pay their staff salaries, mobile banking, debit and credit electronic cards, Mobile money, and cash deposit ATM. It is apparent that all selected banks devoted much attention to financial inclusion and financial innovation because of the benefits to the banks. By expanding their customer base, and leveraging on financial innovation, the banks are able to reduce the cost of their operations (such as account opening, services, and transaction), and consequently boost their income.

Fig. 4.4b below shows the social performance of the banks over a 4-year period between 2013 and 2016:

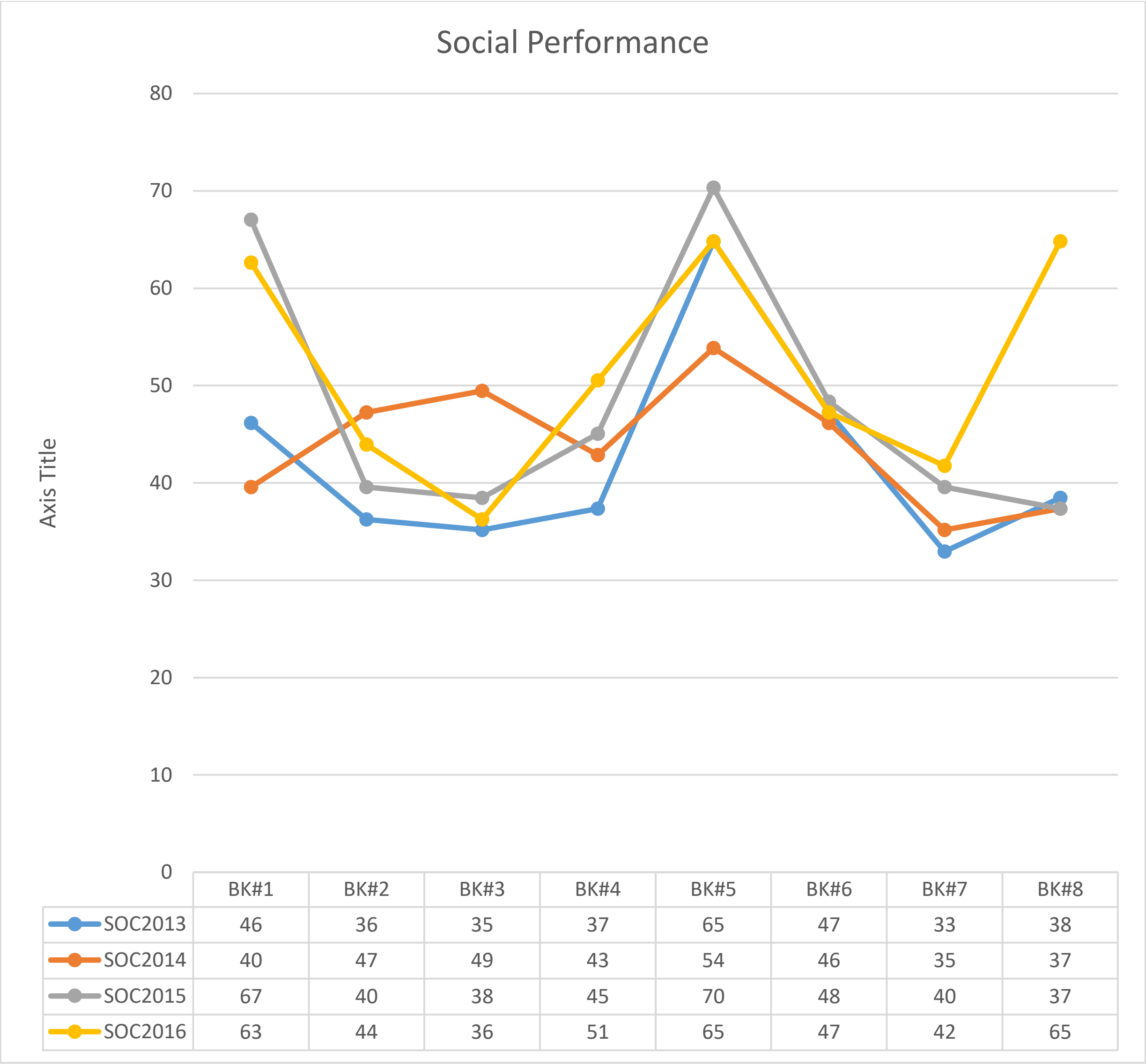


**Fig.: 4.4b: Social Performance**
**Source: Author's Computation (2017)**

From the chart above, the banks generally rated poorly in their social performance. Only BK#5 performed consistently above 50% mark across the four-year period. It should be pointed out though that BK#1 showed an appreciable improvement in 2015 and 2016, while BK#4 also recorded a year-on-year improvement. BK#6 performance was generally flat across the four-year period. Fig. 4.4b shows a generally weak trend among banks in social performance, even though 2016 reported the best performance with 4 banks (#1, #4, #5, and #8) scoring above 50%.

Findings on social performance reveal the following pattern in selected banks:

**Employees**

All banks disclosed a fair remuneration and parity in salary for male and female staff on the same level and performing the same function. Other common disclosure patterns include freedom of association, and non-discrimination in employment on account of gender, origin, and physical disabilities. They showed statistics of physically disabled employees bankwide, albeit low ranging from between three (3) and nine (9). Emphasis is laid on training and development, with most of the banks running their own training schools with opportunities for online and offline training facilities. There is evidence of deliberate policies to maintain a work/life balance, with the installation of gym facilities mostly in head-offices, early closure from office policy, and registration of staff with social and health clubs. Paid maternity leave is enforced for pregnant female staff while BK#8 also enforces paternity leave. Whilst there is a general disclosure of employees' rights to collective bargaining, only BK#5 has a trade union for employees, and a well-outlined grievance mechanism procedure. Only BK#1, 6 and 8 did not record attrition in total employees over previous year. Employees' share ownership was reflected in only one of the selected banks, whilst all the banks had health-and-safety (HS) initiatives. These include installation of fire-fighting equipment, insurance of employees against occupational health hazards, group personal accident and workmen's compensation insurance cover, contributory pension scheme, and national health insurance scheme coverage for full time staff. The staff attrition rate was a major concern for employees during our interview, as the prospect of job security was considered shaky, leading to a general expression of dissatisfaction. This was also reflected by the respondents in the questionnaire.

**Customers and Product responsibility**

All selected banks for the study disclosed different initiatives on customer-engagement. These include events, meetings and business fora, newsletters/publications specially designed for customers, electronic alerts, telemarketing, social media, marketing visits and calls, website, customer ombudsman, customer satisfaction survey, discussion and focus groups. In addition, there are mechanisms in place for resolving customer complaints, such as contact centre, emails, text messages, and a mandatory rendition of customer complaints to CBN on monthly basis. A review of customer complaints in 2016 reveals a high level of dissatisfaction based on the volume (number) and value (amount claimed for refund by customers) of complaints as tabulated below:

**Table 4.33. Customers' Complaints – Volume and Value (2016).**

| | BK#1 | BK#2 | BK#3 | BK#4 | BK#5 | BK#6 | BK#7 | BK#8 |
|---|---|---|---|---|---|---|---|---|
| Volume | 204,773 | 436,376 | 35,966 | 865 | 287,309 | 6,047 | 362,511 | 343 |
| Value | N31bn | N29bn | N5bn | N2.2bn | N68bn | N558m | N41bn | N2bn |

**Source: Annual Reports (2016)**

BK#8 disclosed having a technology platform for auto-reversal of debits for failed electronic transactions and network malfunction, which is a common complaint of account holders. There is generally a poor disclosure on product responsibility. Apart from BK#5, no other bank disclosed policies on ethical marketing practices. The bank's disclosed process includes the vetting of proposed marketing messages by the branding council internally, before submission to the Advertising Standards Panel (ASP) of the Advertising Practitioners Council of Nigeria (APCON) for vetting and examination. A few of the banks disclosed policies on protection of consumers' rights, and the health-and-safety of their customers. On customers' safety and health, some banks have installed wheelchair ramps for disabled customers, in addition to personalized in-bank service by staff. On vendor selection, only BK#1 disclosed a detailed vendor selction process, involving eight traditional assessment parameters, balanced with additional criteria such as ethical, environmental, societal, human rights, labour and governance practices, with the primary objective being to select and do business with sustainability-conscious suppliers.

**Community Development**

All banks had community development initiatives in place. In general, selected banks aligned their community development focus on some of the Sustainable Development Goals (SDGs) – elimination of hunger (goal 2) , provision of good health (goal 3), quality education (goal 4), and good jobs and economic growth (goal 8). Under the quality education goal, banks had several programmes such as adopt-a-school, computer literacy, scholarships, youth empowerment, and promotion of sports development. On goal 2 and 8 (elimination of hunger and creation of good jobs and economic growth), banks provided platform for young entrepreneurs to acquire basic business skills, offered employment opportunities, locally sourced supplies and items, and offered financial support for micro enterprises, minorities and the economically disadvantaged. To promote good health, selected banks invested in health programmes and special institutions such as autism disabilities, visually impaired, sickle cell

centre, cancer screening center, renovation of health centres, and provision of healthcare support for children, pregnant women with HIV, orphanages, and Internally Displaced Persons (IDP) camps. In addition to the selected SDGs, banks also promoted philanthropy through donations and charitable givings, support to government infrastructure such as the security trust funds, and road construction.
All the banks disclosed their employee volunteering programs, which entailed bank employees (individually or collectively under a department) embarking on community development projects such as teaching in public schools, construction of facilities and infrastructures, and donations. In summary, some banks had clear-cut strategy on Community Development (CD) targeted at some of the SDGs, while some still focused more on philanthropy and charitable giving. None of the banks had a policy to set CD spending at a percentage of profit. The study however observed that CD spending reduced significantly when operating profit dropped e.g. BK# 2, 3, 5.

**Human Rights and Ethics**

All banks under study paid penalties for regulatory violations in one aspect or the other during 2016, while there was a differing level of disclosure on their ethics and human rights policies. Only BK#1, 5 and 8 reported staff training on human rights. All banks have whistleblowing channels as mandated by CBN, but whilst some banks have external reporting links to auditors, some others restrict their whistleblowing reporting to internal channels. All the selected banks disclosed having code of conduct, but thee was scanty mention on the broad areas covered. In addition, there was a general one-line statement on the banks' stance on prohibition of child labour, and forced labour.

**Diversity**

There was a general acknowledgment of the importance of diversity by all selected banks, and whilst there was a disclosure of female representation at management and board levels, only two banks (BK#1 and 6) showed evidence of commitment via a set target on gender parity ratio over a specified period. Their sustainability reports also showed a tracking mechanism and an actual implementation of the diversity agenda. Fluctuations in gender ratio for other banks could not be attributed to any visible strategic plan towards achievement of the goal.  Still on diversity, only BK#1 showed evidence of a women's economic empowerment committee to oversee accountability for gender diversity and to steer gender inclusive strategies. It also disclosed investment and dedication of resources for a strong female talent pipeline to fast-track transition from middle management, to executive, and Board level positions.

All selected banks, except BK#7 disclosed initiatives to celebrate women empowerment and provide opportunities for them to connect with senior role models. Overall, the selected banks performed poorly under diversity goal.

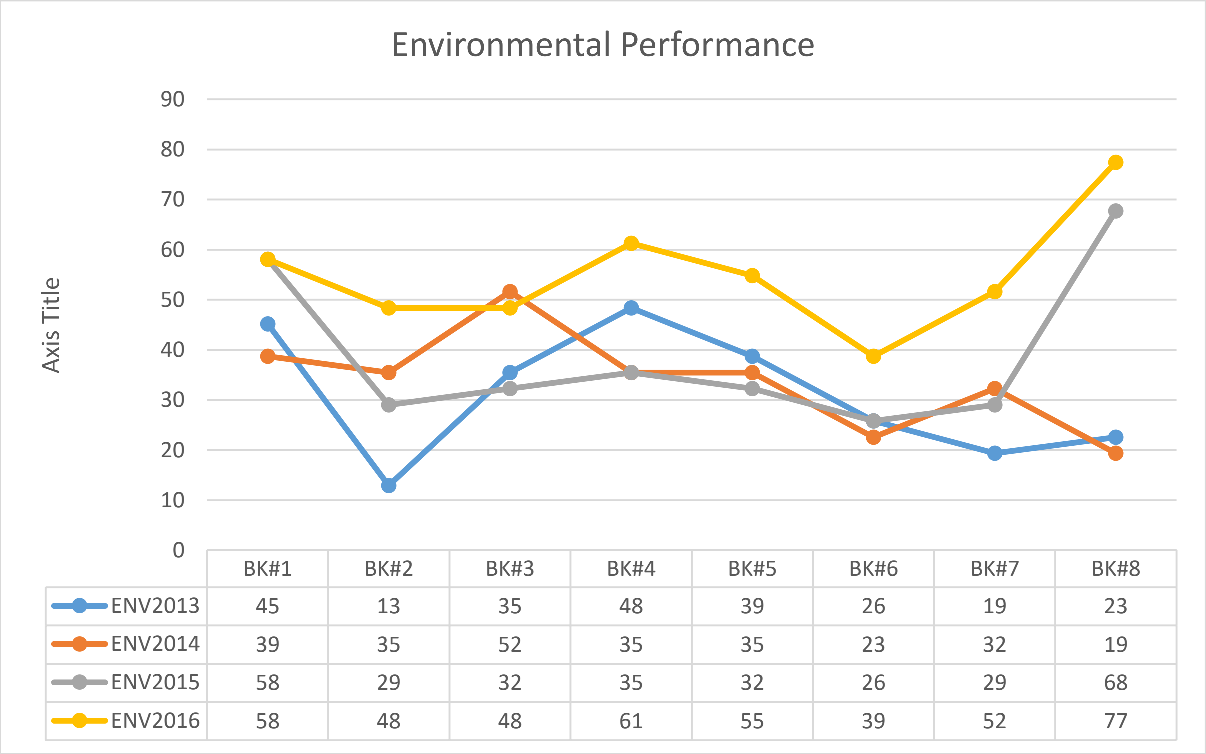


**Fig: 4.4c: Environmental Performance**
**Source: Author's Computation (2017)**

Fig. 4.4c shows a similar pattern to social performance, albeit 2016 had more banks (five) scoring above the 50% mark; these are BK#1, 4, 5, 7 and 8. The inference from the above charts is that economic performance by banks masked their poor performance in social and environmental dimensions. This may be attributable to the fact that economic (financial) performance is still being regarded as the most important indicator of performance by banks, based on factors such as mandatory regulatory requirement for reporting the financials, the perception of the economic indicator as the most appealing to shareholders, and the consistent reporting template for all banks. The lack of consistency in the reporting pattern by banks, and the non-enforcement of a reporting format by banks may account for the low scores recorded by banks. It may therefore suggest that the banks with favourable scores

embrace social and environmental performance reporting as a strategy or business model rather than doing it to fulfil the letter of the regulation rather than the spirit behind sustainable banking principles.

Environmental performance of banks can be categorized under direct impact and indirect impact of their activities. Direct impact relates to the activities of the banks with impact on the environment, while the indirect impact refers to the activities of the banks' customers with impact on the environment. The former category relates to energy efficiency, water consumption, material/paper reduction, waste management, and emissions. The latter relates to banks' focus on responsible lending, to ensure accountability of their borrowing customers in minimizing environmental impacts in their business operations.

On the direct impact performance, there was a generally similar approach by banks on reducing their environmental footprint. On energy for instance, common initiatives included the use of solar and inverter to power ATMs, early closure of branches to reduce energy consumed, LED energy saving bulbs, installation of traffic censors that ensure lightings are automatically turned off when there is no body within a specified perimeter in the work space. The differentiated initatives by BK#8 include the use of eco-friendly and energy-efficient equipment such as air-conditioners and IT servers, and installation of gas-powered generating sets.  Other environmental issues and intiatives are as follows:

Water - Installation of water-efficient cisterns and sanitary fittings to optimize use of water, reliance on boreholes rather than public water supply,
Material/Paper – paper re-use and recycling, use of front-and-back printing mode, allocated monthly quota, periodic reduction of monthly quota, monthly analysis of consumption, tracking of allocations electronically, and use of online documents for meetings. Only BK#1 had an additional unique initiative, the use of Forest Stewardship Council (FSC) certified papers.
Waste - disposal of fully depreciated items, partnership with e-waste management firms, investment in standard bins that differentiate and segment wastes into paper, glass, liquid (bio-degradable), and plastic wastes for ease of recycle. There is a partnership with waste management firms such as Lagos Waste Management Authority (LAWMA) to classify, measure and dispose of wastes. Banks wastes are generally not considered hazardous, as they include unused automobiles, computers and

accessories, generators, air conditioners, construction wastes, papers etc. BK#3 uniquely has a waste-to-wealth project partnership/franchise with a waste management company.

Emissions – Emissions are generated directly and indirectly. Direct emission is caused by fleet of vehicles, and generator sets; indirect emissions come from power from the national grid, refrigerants, business related flight travels, and paper consumption among others. Common initiatives by selected banks on managing their emissions include: provision of bus fleet for mass staff movement/carriage, carpooling, travel control measures (video-conferencing, tele-conferencing, Voice-Over-Internet-Protocol (VOIP) calls, elimination of diesel-powered vehicles, use/reduce-reuse-recycle initiative (on inverters, batteries, computer accessories, electronic wastes). BK#4 had a differentiated strategy, by investing in the use of roofing sheet gauge 0.75mm on its new branch buildings thereby reducing energy requirement for ventilation and air conditioning equipment. In addition, all glass material fittings used at its new branches are thicker, tinted or reflective. Other initiatives include planting of trees, beautification of gardens and parks, and formation of students' conservation clubs as vehicles to drive environmental protection and conservation.

While banks have shown their initiatives in reducing direct environmental impact, only BK#8 engaged professional advisors to conduct a greenhouse gas audit to measure the impact of its operation emissions on the environment. The 2016 report shows a total greenhouse gas emission of 14,579.9 tCO2e in the bank's Head-office alone. The contribution in terms of sources are: Car fleet 42.7%, generators 35.1%, refrigerants 10.8%, PHCN 9.1%, flights 2.1%, and paper consumption 0.3%. In layman's term, to offset the bank's ghg emissions, "the total emission equates to 1,534.6 trips around the equator; 539,996.3 kg of raw beef; a forest of 1,137 km2".

On the indirect impact activities on the environment, all selected banks maintain a software with a generic name Environmental, and Social Risk Management System (ESRMS) which screens all transactions (especially lending) for environmental impact assessment. The software allows banks to conduct due diligence assessments on qualifying transactions and follow-up onsite visitations for select projects to assess the customer's level of environmental performance. BK#5 reported screening and assessing a total of 133 transactions worth N559billion in 2016 for ESG risks, while BK#8 had 28% of all its transactions worth N840billion screened and accessed for ESG risks. Disclosure by banks corroborated by repondents during the interview sessions show that the level of Environmental Impact

Assessment (EIA) screening is still low as at the time of this study. The desired expectation is for all banks to achieve 100% in EIA, and other social and environmental screening. The study also found out fom respondents that CBN does not currently review/scrutinize sustainability reports by banks, which allows for inconsistent reporting pattern. The implication of this is that whilst a meticulous bank may decline a credit application on account of not passing the ESRMS test, another willing/compromising bank may approve the same credit application, thus defeating the sustainability objective.

The banks have differing level of structures in place for overseeing their sustainability programmes. While some have fully-fledged units on sustainability, others have the functions subsumed under different departments such as corporate affairs, risk management, controls and compliance. None of the selected banks has a Board Sustainability Committee, but have committees such as Board Risk Management Committee overseeing the sustainability reporting. Conversely, they all have collaborative partnerships with sustainability instituions such as United Nations Global Compact (UNGC), United Nations Environmental Program Finance Initiative (UNEP-FI), Nigeria Conservation Foundation (NCF), Sustainability Centre (of the Lagos Business School, Pan-Atlantic University), and IFC among others.

Only a few of the selected banks (BK#5, 7 and 8) engaged external consultants to provide independent assurance on their sustainability reports, whilst only BK#6 mentioned sustainability in the Chairman/CEO report embedded in the annual report. All the banks except BK#4 embraced ‘green’ technology in its buildings, whilst only BK#3 disclosed its Socially Responsible Investment (SRI) policy supporting sustainability innovative business opportunities.

All banks are subject to environmental compliance audit carried out by environmental regulatory agencies, which result in fines for non-compliance. Such audits include request for proof from banks on compliance with environmental impact assessments.

#### 4.4.6.2 Correlation Coefficient

Pearson Product-Moment Correlation Coefficient has been credited with establishing an index of relationship between two variables. The correlation analysis is represented by the symbol r and it reflects the degree of linear relationship between two variables.

**Table 4.34: Correlation Coefficient of Corporate Governance Practices Disclosure and Sustainability Performance Reporting**

**Correlations**

| | | SIND | CGIND |
|---|---|---|---|
| SIND | Pearson Correlation | 1 | .461** |
| | Sig. (2-tailed) | | .008 |
| | N | 32 | 32 |
| CGIND | Pearson Correlation | .461** | 1 |
| | Sig. (2-tailed) | .008 | |
| | N | 32 | 32 |

**. Correlation is significant at the 0.01 level (2-tailed).

**Source: Author's Computation (2017)**

In table 4.34 above, the relationship between corporate governance practices disclosure and sustainability performance reporting (as measured by economic sustainability, social sustainability, and environmental sustainability) was investigated using Pearson product-moment correlation coefficient. Preliminary analyses were performed to ensure no violation of the assumptions of normality, linearity and homoscedasticity. There was a moderate, positive correlation between the two variables (corporate governance practices disclosure and sustainability reporting), $r = .461$, $n = 32$, $p < .01$. The result shows that the positive association between corporate governance practices disclosure and sustainability performance reporting in the eight banks is an indication that as each of the banks responds strategically to changes in corporate governance practice disclosure, sustainability performance reporting also increases.

#### 4.4.6.3 Regression Analysis between Corporate Governance Practices Disclosure (CGPD) and Sustainability Performance Reporting (SPR)

Having determined the strength and direction of relationship between Corporate Governance Practices Disclosure and Sustainability Performance Reporting, a multiple regression analysis was conducted to determine the significant contribution of Corporate Governance Practices Disclosure on Sustainability Performance Reporting based on the combined eight banks sampled. The results of this analysis revealed how much of the sustainability performance reporting can be explained by corporate governance practices disclosure. Preliminary analyses were conducted to ensure no violation of the assumptions of normality, linearity, multi-collinearity and homoscedasticity.

The model specification tested is:

Sustainability reporting = *f* (Corporate governance practices disclosure)

The results are as shown below:

**Table 4.35: Model Summary –CGPD and SPR**

| Model | R | R Square | Adjusted R Square | Std. Error of the Estimate | Durbin-Watson |
|---|---|---|---|---|---|
| 1 | .461[a] | .212 | .186 | 11.169 | 1.434 |

a. Predictors: (Constant), CGIND

b. Dependent Variable: SIND

**Source: Author's Computation (2017)**

**Interpretation of Results:**

The results from the model summary table 4.35 above revealed the extent to which the variance in the dependent variable (Sustainability Performance Reporting) is explained by the independent variable (corporate governance practices disclosure). In this case the R square is .212, which expressed as a percentage equals 21.2%. This connotes that 21.2% of the variance in sustainability performance reporting can be predicted from the corporate governance practice disclosure variables. The adjusted R square shows .186 (that is 18.6%) which explains the variability of the independent variable (corporate governance practice disclosure).

**Table 4.36: ANOVA –CGPD and SPR**

| Model | | Sum of Squares | df | Mean Square | F | Sig. |
|---|---|---|---|---|---|---|
| 1 | Regression | 1009.090 | 1 | 1009.090 | 8.089 | .008[b] |
| | Residual | 3742.410 | 30 | 124.747 | | |
| | Total | 4751.500 | 31 | | | |

a. Dependent Variable: SIND

b. Predictors: (Constant), CGIND

**Source: Author's Computation (2017)**

Table 4.36 above assesses the statistical significance of corporate governance practices disclosure on sustainability reporting. This analysis tests the null hypothesis that multiple R in the population equals 0. The rule is that, a model reaches statistical significance when Sig. = .000; this in other word means that p< .05. Therefore, from table 4.36, corporate governance practices disclosure is statistically significant to sustainability reporting, where Sig. = .05 {F (1, 30) = 8.089}.

**Table: 4.37 Coefficients - CGPD and SPR**

| Model | | Unstandardized Coefficients | | Standardized Coefficients | t | Sig. |
|---|---|---|---|---|---|---|
| | | B | Std. Error | Beta | | |
| 1 | (Constant) | 31.372 | 18.565 | | 1.690 | .101 |
| | CGIND | .616 | .217 | .461 | 2.844 | .008 |

**Source: Author's Computation (2017)**

**Interpretation of Result:**

The coefficient table 4.37 above shows the simple model that expresses the extent to which corporate governance practice disclosure has an effect on sustainability performance reporting.

The model revealed that corporate governance practice disclosure records a beta value of (*beta* = .461, with a $T_{val}$ higher than 1.96, *Sig*. .05 $p < .05$). The regression equation from the above table is:

Sustainability Performance Reporting Predicted = 31.372 + .616*Corp_Gov.

This by interpretation means that for every unit increase in corporate governance practice disclosure, a .616 corresponding unit increase in sustainability performance reporting is predicted, holding all other variables constant.

**Decision Rule:** Reject the Null hypothesis, when the significance value is below 0.05. Do not reject hypothesis, when significance value is greater than 0.05.

**Interpretation of Result:** The ANOVA table shows that the F value is 8.089 at $.008^{b}$ Significance level. The implication is that corporate governance practices disclosure has a significant effect on sustainability reporting.

**Decision:** Reject the null hypothesis. Therefore, there is a significant effect of corporate governance practices disclosure on sustainability performance reporting.

**Table 4.38 Rejection or Acceptance of the Null hypotheses**

| **Null Hypotheses** | **Accept** | **Reject** |
|---|---|---|
| **$H_{01}$:** Internal governance controls do not have significant effect on corporate social performance in selected deposit-money banks in Nigeria. | No | Yes |
| **$H_{02}$:** Ethical leadership does not have significant effect on corporate reputation in selected deposit-money banks in Nigeria. | No | Yes |
| **$H_{03}$:** Regulation does not have significant effect on financial innovation in selected deposit-money banks in Nigeria. | No | Yes |

| | | |
|---|---|---|
| **$H_{04}$:** Agency mechanisms do not have significant effect on financial performance | No | Yes |
| **$H_{05}$:** Bank characteristics do not have significant influence in moderating the relationship between agency mechanisms and financial performance. | No | Yes |
| **$H_{06}$:** Corporate governance practices disclosure does not have significant effect on sustainability performance reporting. | No | Yes |

**Source: Author's Compilation (2017)**

## 4.5 Qualitative Findings Based on Thematic Analysis

This section presents the data gathered through the semi-structured interviews conducted for key players in the banking industry. A total of fifteen (15) respondents were interviewed. Table below shows the profile of respondents for the semi-structured interviews.

**Table 4.39 Interviewee Profile**

| **Profile** | **Number** |
|---|---|
| Bank Chairman | 1 |
| Bank Executive Director | 3 |
| Company Secretary -Internal/External | 2 |
| General Managers - Controls/Business Dev/ /Risk Management/IT/Operations | 7 |
| Heads Sustainability Departments | 2 |
| Regulator -Director | 1 |
| **TOTAL** | **15** |

**Source: Author's Computation (2017)**

The responses were analysed and the emergent themes from the interviews are stated below:

**Table 4.40 Emergent themes and Sub-themes from Interview Respondents**

| # | **Theme** | **Sub-themes** |
|---|---|---|
| 1 | Role of the Board | Appointment and removal, tenure, board size, board composition, independence, succession planning, diversity, remuneration, board chair characteristics, |
| 2 | Ownership Structure and Shareholders' Rights | Concentration, Institutional Investors, Insider ownership, minority shareholders, general meetings, shareholders' education |

| | | |
|---|---|---|
| 3 | Role of the Executives | Board Information, CEO attributes |
| 4 | Risk Management | Risk management practices, whistleblowing |
| 5 | Ethics | Ethical culture, Code of ethics, vision and mission |
| 6 | Strategy | Formulation, monitoring |
| 7 | Institutions | Regulators, Legal system, Culture |
| 8 | Performance and effectiveness | Training, evaluation, board's role in performance, effectiveness |
| 9 | Sustainability | Concept, performance metrics, social responsibility (customers, employees, community, environment), sustainability enablers, influence of corporate governance |

**Source: Author's Compilation (2017)**

### 4.5.1 Theme 1: The Role of the Board

***Appointment and Removal of Directors:*** *'The appointment process is more of a box-ticking in some banks, as the board is composed of the MD/Chairman's cronies'* (P#6). '*Appointment is largely non-transparent as the owners see it as an opportunity to entrench their interests. Paradoxically, as unethical as some owners are, they demand ethical behaviour from people, otherwise how would you justify armed robbers after stealing your money they want to share the money they start killing and shooting themselves, that means they too want fairness'* (P#4).

*'Poor-performing CEOs/directors not fired by the Board is due to the lack of independence of the board because of the over-bearing influence of the CEO, either he is the owner of the business or installed by the owner of the business'* P#12

***Tenure:*** *'Tenure limit is needed. People come in at a matured age, and in order not to have a geriatric board or people becoming part of the furniture there's a need to refresh the board'* P#12.

*'The governance code provides for a retired MD to return as chairman after observing a cooling-off period. Unfortunately, this is a farce because the retired MD never really left. He still remained a substantial shareholder therefore able to appoint a successor and management team he could control'* (Participant #6). '*Cooling-off period should be eliminated. No one is a monopoly of knowledge, and therefore should allow others to grow'* (Participant #5)

***Board Size:*** *'The board size should be a function of the organisation's size or its operations. To the extent that the Board is not expected to be involved in operations the nimbler, the more effective. Having a large board size may affect their ability to take decisions even though there is the benefit of having diverse opinions, but too many diverse opinions on the other hand may make decision-making cumbersome'.*

***Board Composition:*** *'A board with a lot of old people presents a problem especially in the light of products and services being churned out by banks. Too many old men on the board also gives room to management/CEO to bamboozle the board members, and while they won't want to publicly show their ignorance, they may nod in agreement to approve decisions/recommendations, but while in their private space they confess their shortcomings. When shareholders seek to appoint directors at AGM, they tend to look only at the same pool of so-called tried and tested people who have now turned to old men, and if care is not taken the CEOs will take advantage of their lack of knowledge of modern trend' P#6*

***Independence:*** *'Independence is not achieved just by designating some non-executive directors as Independent. What is required is to have board members with an independent mindset. To assure independence of the board, it is important that non-executive members be engaged in other income-earning ventures beyond directorship of the organisation in order not to becloud their judgment. They must be able to take a walk from the Board job rather than compromise their principles'* P#6

*'The degree of a board's independence depends on its level of greed or hunger' P*#12

*'You cannot have an independent board but a board with independent elements inside it for the organisation to be sustainable' P* #7

*'Independence from executive management and ownership is very important, and this can be determined from the transparency of the financials, proportion of insider-related transactions to total loan, level of disclosure by each director and reporting of directors violating disclosure policy among others'* P#10.

*'If anyone tells you the board is independent it's not true. Maybe on non-material issues, but when they talk about high level material decisions that would impact on the shareholders directly they just forget about independence. Those who run some of these organisations are not even in the organogram, and they are not even on the board. The power broker may be the wife of the chairman, because even when*

*you call the owner of the bank, he in turn would also say let me get back to you. He would now call madam and ask "who should we put in Paris office. Do you have anyone in mind? She'd say Yes'* (Participant #4)

***Succession Planning:*** *'Sometimes the board members tend to think that culture and succession planning are the remit of Human Resources (HR), but the board has to have a hands-on involvement in the succession plan particularly at senior management level' P#6*

***Diversity:*** *'Having members with geographical spread is important. This should not be misinterpreted as quota system or federal character. Female board members have important contributions on the board. For instance, they stand against cronyism, bring in different chemistry, ask more questions, pay more attention to details, may take longer in decision-making, risk averse, but pay more attention to softer issues. Nevertheless, the board should get the right woman rather than just tokenism' P* #6

***Remuneration:*** *'There should be standards on remuneration such that directors are not beholden to management. Huge remuneration may distort the board members' judgment, while inadequate remuneration may not bring out the best in members' productivity. While the organisation may not be able to compensate board members for the risks they take, the remuneration should not be such a quantum to affect their independence' P#2*

***Board Chair Characteristics:*** *'The board chair must be able to command peers' respect, have listening skills, have conversational intelligence, assertive, approachable, and consult/receive feedback' P#6. '.....must be a team builder -like the conductor of an orchestra, must also have humility, good listening skills, integrity, leadership by example, transparency, open-door policy and investing in understanding how the organisation makes money' P#12*

### 4.5.2 Theme 2: Ownership Structure and Shareholders' Rights

***Concentration:*** *'Ownership is concentrated in the hands of a few' P*#6. *'There are few dominant shareholders, people who don't qualify to be called controlling shareholders, yet they hijack the running of the organisation. They call the shots even outside the organisation. They play the music and ask people to dance; hold the bank by the jugular by recruiting family members and populating the various board committees with their cronies'*. P#5

***Institutional Investors:*** *'Institutional investors are the largest bloc of shareholders but are not doing enough in enforcing proper governance. When they see that banks are not performing rather than divest they should enforce changes for instance by removing non-performing CEOs at the AGM. Unfortunately, they don't even attend AGMs, because for as long as they keep getting returns on their investments they are less bothered about corporate governance. This is a mindset that needs to change. They need to be active in exercising their powers at the AGM rather than leave such meetings to rabble-rousers' P#6. 'Our institutional investors are not vocal because they are retail investors. Pension funds should be strong institutional investors but unfortunately the government takes all the money and determines where they are invested' P#*12

***Insider Ownership**: 'Having insider owners further concentrates ownership on the board, which gives them an overwhelming power to get involved in insider credits, conflicts of interests, influence the regulators, and expropriate or cream-off before other shareholders get the benefits'*P#6

***Minority Shareholders:*** *'Minority shareholders are not protected' P#6. 'Minorities would have their say but majority would have their way. However, they have certain inalienable rights which are well-protected. These include rights to vote, attend meetings, ask questions and receive answers, receive dividends, and participate in profit' P#13*

***General Meetings:*** *'AGM is one big farce in Nigeria. Look at the process of appointing shareholders' representatives to Statutory Audit Committees for instance, management subverts the process by allowing a confusion in form of unwieldy list of nominations, and supposedly come to the rescue by settling for the board or management's pre-determined representatives. Agenda of the AGM is mostly rubber-stamped by management behind the scenes. There has only been one instance of shareholders rejecting a director's nomination for re-appointment, and this was because the board and management was in reality not in support of the re-election of the director, and rather than force the director out of the board, he was presented at the AGM knowing the outcome in advance'* P#6.

***Shareholders Education**: 'Shareholder activism is very poor. Shareholders associations are fragmented hence not effective. They don't seem to know what they want beyond going to AGM to make*

*noise. If they are able to consolidate their associations to speak with one voice they will be a force to reckon with' P#6.* '*The nearest attempt at having a strong voice for the shareholders is the shareholders' association, but unfortunately, they are extortionists'* P#14. '*There is a weak shareholding system where people don't know what their rights are. There's little or no shareholder education'* P#15

### 4.5.3 Theme 3: The Role of Executives

***Board Information:*** *'The quality of information provided for Board's deliberations is largely dependent on the transparency of the management, and we have instances where a board fails to live to its responsibility by relying absolutely on what the MD provides without diving deep into it. In addition to quarterly reports, the board must be able to request for additional and ad-hoc reports'* P#6. *'Materials for board come from executive management, hence the board may be blank except they have a deep knowledge, and this is where the transparency of the management will determine the state of corporate governance in the bank'* P#15

***CEO Attributes:*** *'The attributes of the CEO required for success depends on the life-cycle of the business at the point in time, but it's important for the CEO to be up and moving rather than being armchair executive, assertive in managing domineering shareholders on the board, and accountable*' P#3. '*openness, team spirit, accessibility,* b*usiness knowledge and competence are assumed given' P#4 'ability to manage and motivate the team, and be ethical by not allowing personal interests to override the bank's overall objective' P#2*

### 4.5.4 Theme 4: Risk Management

***Risk Management Practices:*** *'Few banks have risk management experts. Moreover, there is the general tendency to pack risk management into a department rather than see risk management as a way of life and core competency for all staff, forgetting that everything in the bank is about risk-taking'* P#13. '*People making the credit decisions at the board level are rarely knowledgeable about the intricacies and therefore rely heavily on what management presents to them. It's the transparency of management that therefore matters'* P#2.

*'There is the problem of banks failing to properly assess risk but still blindly giving out credits on grounds that the borrower is a successful business man and therefore whatever he goes into must be successful. They argue that they have collaterals to fall back on, forgetting that banks are not in*

*collateral recovery business but cashflow business, because even when the collaterals are ten times the value of the credit, realising the collaterals sometimes becomes an issue'* P#12.

*'Our risk management practices adopt enterprise-wide risk management framework'* P#2, P#7

***Whistleblowing:*** *'There is a whistleblowing policy in place, though without independent external auditor's hotline. There have been cases referred to the board, but there haven't been ground-*breaking *discoveries in terms of amount or personality'* P#3. '…*cannot be considered very effective yet because internal fraud level hasn't subsided, while no reported case has been made at very top senior level. It has unearthed irregularities such as supervisors at branch level involved in frauds, misdemeanours, and collusion with outsiders'* P#7

### 4.5.5 Theme 5: Ethics

***Ethical Culture:*** *'There is annual disclosure by the Board members, of companies in which they have interest - be they contractors or borrowers to/from the bank. A board member has to recuse himself/herself when issues relating to his/her interest or credit is being discussed, and this is recorded in the minutes'* P#8

*'CBN reviews the bank's code of ethics during bank examinations, there is a code of ethical practices for banks which is binding on all banks'* P#1. '*Every employee gets a copy of the code of ethics from Human Resources department on resumption, while the soft-copy is updated semi-annually and forwarded to all staff. In addition, there is mandatory e-learning on values annually, with employees required to take a test. They have two attempts to pass, otherwise their overall performance appraisal score is scaled down'* P#7.

*'Ethics is highly prized in the bank. For instance, if the bank increases rate today on fixed deposit from 5% to 7% and the customer comes back to roll-over and you still book the customer at 5% without you telling him of the increase and the bank gets to know, it becomes a serious issue for the staff. I have also not seen where the MD stalls an investigation*' P#4. '*There is zero tolerance for unethical practices*. Our *foreign investors are not just concerned about the end, but the means to the end as well, especially how targets are met, they query management when media breaks out any news linking the bank to any criminal or fraudulent customer'* P#2.

*‘Good corporate governance is not achieved through regulations, otherwise it becomes a checklist. It should be a culture driven by values. People do crazy things, make it and are celebrated but in the long term they lose everything. Organisations must make employees see long-term benefit in decent behaviour, otherwise they see corporate governance as a set of rules to be complied with when the bosses are around and flouted when not around’ P#12*

*‘At a certain level profit becomes criminal. Making profit can become criminal if you are not careful as a business manager, for instance if you keep deposit for Evans the kidnapper, knowing full well that he is a criminal’* P#4

*‘By building values into the vision, and mission statement and walking at it, other organisations pay attention once there are a sample of visible rewards for decent behaviour’ P#12.*

*The founding fathers set out to build an organisation that has at its bedrock professionalism, integrity, honesty and discipline. They separated the business from personal life, and instilled a culture of "doing things well " for the employees to follow. Relationship with international institutions early on encouraged the inculcation of values and adherence to best practices’* P#5. ‘*It might be criminal for any accountant or finance manager to stay in an organisation that collapses on his head. Apart from the fact that he loses his job, he should be prosecuted and jailed because the financial indices are glaring on his table’* P#4.

### 4.5.6 Theme 6: Strategy

*‘The board's role is not to police management but to create a future for the organisation. The board must play a key role in strategy formulation’* P#12.

*‘There is a three -to- five-year rolling plan, bottom-up approach to strategy formulation, and the engagement of external consultants’* P#7, P#8

*‘The board reviews the policies, procedures, budgets, risk management framework and the strategy implementation on quarterly basis’* P#5.

*‘Strategy formulation has been dependent on the CEO in place. There was a former CEO that involved all staff, management, consultants and the board in formulating the bank's strategy, but the current CEO single-handedly prepared the strategy document and just dumped it on the board’* P#2.

### 4.5.7 Theme 7: Formal and Informal Institutions

**Formal Institutions -Legal System, Regulators**

***Regulators:*** *'The regulators have improved recently' #3. 'CBN now holds the board more responsible than ever before… The introduction of risk-based audit has improved the supervision function of the CBN' P#10. 'CBN has been very effective in protecting depositors. Policies on limits, sectoral concentration etc are aimed at stability. CBN's duty is not to protect the shareholders but the depositors. So, if the shareholders lose all their investment by allowing rogues to manage the bank, getting them profit over the years and not shouting where are you getting this profit from until the big stick comes they have themselves to blame'* P#4.

*'The fear of CBN is the beginning of wisdom in banks because of the regular threat of revocation of licence' P#2. 'They are also firmer in ensuring appropriate provisioning for bad assets, and their ability to sack the board puts fear in management and the board, but are still sometimes slow in reacting to emerging issues'* P#7. '*The level of supervision and control has improved, and they are so versatile unlike before. If they make recommendations two times and you don't comply they sanction you'* P#4

*'The failure of the past was caused by banks going into esoteric financial instruments in the name of financial innovation, which the practitioners didn't even understand until the bubble burst. Banks must be accountable and focus on pursuing sustainable returns'* P#1.

*'It's getting difficult to define the boundary of banking as the business goes into fintech. The issue of knowledge, risk etc becomes imperative for the regulators to monitor risk issues emerging from the internet, and automation'* P#7

*'The regulators are a product of the environment in which they operate' P#15*

*'The way regulatory policies come out are sometimes not well thought-out, but regulatory arrogance doesn't allow regulators to reverse their mistakes'* P#8.

*'Regulators need to partner and engage more with practitioners rather than just being rule-enforcer and sanctions-motivated, otherwise practitioners will focus more on compliance with letter of corporate governance rather than the spirit' P#6*

*'Regulators need to upgrade the knowledge of their staff on bank examination. Industry practitioners are far more knowledgeable than the regulators.* Compliance *with regulations is out of fear of penalty and not because the banks agree with their recommendations, which are sometimes sub-optimal and contradictory'* P#2.

**Legal System:** *'Can we actually expect to have mini-saints in the corporate world in this kind of society we have? In a society where you don't get justice, you pay for 'jankara' judgment, how will it affect corporate behaviour? As long as we have weak institutions we will not have widespread acceptance' P#12*

*'There is a need for review of Companies and Allied Matters Act (CAMA), but that doesn't explain corporate governance problems. The issue is mindset and weakness of enforcement because the laws are there. Administration of the legal system is the problem due to the Nigerian factor - indiscipline, corruption, lack of sincerity, and nepotism'* P#8

*'In an economy where the government is the biggest spender corporate governance shouldn't be in the private sector alone. The drive should significantly involve the public sector'* P#12

*'We are in a democracy and must follow the rule of law and due process irrespective of how people feel. There are cases of directors who ran banks aground in court but our legal system has a general problem with many cases in courts taking a long time to adjudicate say over seven to eight years. People after running banks aground have the resources to hire lawyers to ensure justice is delayed by bringing on technical issues. CBN's role is to sack the board and hand over to the authority in charge of prosecution. Even though there's pain about mismanaged banks, the errant directors still have their rights. This is unlike the era of a former military dictator when board members could just be herded into prison'* P#1

**Informal Institutions -Culture**

**Culture:** *'People believe that because they own an asset it must continue to feed them and provide for their needs even when the asset is sick. How do you explain a founding chairman paying salary from the bank's resources to wife, children and other relatives who are not in the bank's employ?'* P#5.

*'People still ask, how can a regulator prevent me from having my children on the board after founding the organisation and nurturing it to a thriving business entity? How can I hand over the sweat of my labour to another person rather than my family members?' P#6*

*'It is part of our cultural problem to believe that this asset is mine and therefore we should continue to hold all our assets by the jugular forgetting the fact that sometimes you allow an asset to be optimised by allowing professionals to manage'* P#5

*'We run a culture that requires blind loyalty. A boss says if he puts something inside his office and you work for him, you must defend his interest, protect him, be loyal to him so that he can increase your wages and treat you well' P#4*

*'We have a poor culture of tolerating badness. We see elders or supervisors engaged in bad practices but choose to keep quiet because we are not expected to rebuke our elders or bosses'* P#3

*'Check very well, bank failure has been associated with banks owned by Nigerians. You have to go back to history of these organisations. They all start as family-owned and eventually go public, but like most Nigerian companies they find it difficult to let go even when they have sold a substantial share of the company to others or the public. They find it difficult to work under a framework where they can be guided by a board that can exercise strong oversight. Therefore, it's not surprising that the organisations don't last beyond the first generation' P#12*

*'To demonstrate our cultural problem with assets ownership, my father bought a motorbike in 1979 and still keeps it in the house (thirty-eight years after), even though it has fallen into disuse'* P#5

*'Ego drives some CEOs. For instance, they approve bad credits for friends because they believe that 'I am the CEO and that's my friend, how can I deny my friend something, he'll probably think I'm not as powerful as I project myself'. It's the same factor accounting for their foray into stupid mergers, acquisitions, and mega-deals because they want to run the biggest bank and in order to feel good about it'* P#12.

*'The level of greed of senior management and the board will have significant impact on corporate governance practices'* P#12

### 4.5.8 Theme 8: Performance and Effectiveness

*'Where an organisation does well, management takes credit because they are the visible players. Where the organisation performs poorly on the other hand the board takes most of the blame* as people ask "*who are the directors that have bled the bank*"' P#12.

*'The board can be likened to the father, when the company does well the board gets praise, but if the company doesn't do well the board gets the blame, therefore a company rises and falls with leadership'* P#8.

***Training:*** *'There is continuous directors' education (local and offshore) relevant to their committee functions. In addition, they are exposed to regulatory-mandated training such as those prescribed by SEC, NSE, CBN etc' P#8*

***Evaluation: '****The board, and board members are evaluated by directors and external consultants, with areas of evaluation including knowledge, leadership, meeting preparation and participation among others' P#5.*

***Role in Performance:*** *'I sincerely cannot see any situation in which you'll say "if not for the board, or board member we would not have had this business or lost this business opportunity*" *The bank doesn't rely on that aspect of their presence -bringing in business, but their influence on performance is in the area of providing support, value-addition to policy proposals, their oversight function, monitoring of achievement of annual plans and set targets'* P#3. '*The board's role in the bank's performance comes from the level of interaction between the board and management. The board critically reviews budgets, strategies, and goals of the bank as presented by management, and monitors the results on quarterly basis'* P#10

***Effectiveness:*** *'The composition of the board committees, leadership, and cohesiveness of the board and board-management relationships determine the effectiveness of a board. A good relationship between the board and management can be determined by measuring the quality and timeliness of management information at the board's disposal' P#12*

### 4.5.9 Theme 9: Sustainability and Corporate Reputation

***Sustainability Concept:*** *'Anything that can inhibit the long-term success and survival of your business is a sustainability issue, and these include factors such as social, economic, environmental, corruption, and youth empowerment among others'* P*#12.*

*'We are in business to make money today, and tomorrow, and to leave an institution that will survive and outlive the founders'* P#*8.*

*'We're looking at the whole of economic, social and environmental impact of our lending activities and our operations'* P#*9.*

*'It means responsibility. Ensuring that as an organisation, in your everyday operation there is the consideration of the security of future generation on every activity as it affects the profit, people and planet'* P#*11.*

*'We are in the business of lending and when you lend to companies that are irresponsible either in the area of social or environmental consideration, you are adding to that narrative. For instance, you lend to a company that wants to build a plant and they have to displace people in a particular community, if we support such a company at the end of the day, it is the big fish like the bank that will be mentioned not the small company that violated the environmental requirement' P#9.*

**Performance Metrics:** 'The *board's performance measurement focus comprises of a balanced scorecard approach' #12*

'The *key focus of the board on the bank's performance include financial, credit and other risks, employee satisfaction, engagement and attrition, sustainability metrics, and the board's annual objectives'* P#8.

*'Unlike in the past where performance focus was primarily on boasting about balance sheet size, the recent trend is a focus on both financial and non-financial measures' P#3*

'The *focus of performance measure is largely on the quality of the credit portfolio, effective risk management, achievement of the banks strategic goals - market, deposit mix, branch outlay etc, with little pressure on profitability, on the premise that once the credit quality mess has been cleared, profitability will follow' P#2*

*'... secret to sustainability rests on recreating our institution, investing in research into market needs, innovation, adopting local and international standards, rejuvenating the workforce by letting those who are not pulling their weights go, and replacing them with capable hands. In addition, a focus on customer needs, which are: speed of service, security, and convenience, .... continually work on social responsibility, fine-tune and maintain the strategy focus, and embark on dynamic transformation program'* P#3.

*'Our sustainability program includes environmental and social risk management framework, and sustainability policy which speaks to financial inclusion, women empowerment, financial literacy, capacity building, employee volunteering schemes and support to the community'* P#9.

*'.....we focus on financial inclusion, women empowerment, community support, human rights issues, initiatives and intervention programmes addressing environmental issues such as resource efficiency, providing strategic funding and financing solutions, driving innovation and leadership in the industry'* P#11.

*'Commitment to maintain the corporate reputation is vital for sustainability. There is a constant messaging about passing on the brand legacy to others at every forum …..Commitment to regulatory compliance is also key for sustainability. Here for instance, the bank will not touch a transaction if we are going to make N1 billion income and pay a fine of ten thousand naira'* P#4

**Innovation:** *'We have an in-house innovation team, a partnership with Telcos and an investment in a Fintech firm. We also have a robust governance framework in to support our innovative activities'. P#14*

**Social Responsibility:** *'How can you operate in a place where you are the only prosperous person, and every other firm around you is poor? How can workers come from slums and go back to meet hungry family? Every social problem is an opportunity. It does not necessarily mean you do philanthropy, but ask how to bring economic and social benefits to the people and community' P* #12

**Customers:** *'For our customers, our role is to make them happy. They want convenience, service, and innovative products'* P#5. *'…we focus on services, channels' expansion and improvements, customer complaints'* P#7. '*Our customers are quite loyal to us'* P#4. '*We work with our customers to improve their business operations to be more environmentally-friendly. We have similar partnership to promote good governance structure in our customers' businesses by setting up structures, processes, governance system etc in order to cultivate a long-term relationship'* P#8

**Employees:** *'The bank has an efficient talent management system, performance measurement system, human resources practices covering grievance management, disciplinary process, transparent appraisal system, career path, job rotation, equal opportunity, and gender sensitivity'* P#5. '*We actively*

*pursue employee satisfaction through various measures such as a work-life balance, job security, grievance mechanisms, diversity promotion, training, and career advancement' P#2, P #3*
*'…rather than lay off staff, management opted for a cut in pay across board. When the economy nose-dived, HR considered poor performance over 3-4 appraisal periods before laying off staff, thus there is no arbitrariness'* P#7. '*Commitment to employees' welfare is key to sustainability. Attrition rate is very low in the bank, the bank has won the "great place to work" awards severally, there is longevity of stay with some having spent 15-20 years on the job, good medical scheme, talent management and succession plan'* P#4

***Community:*** *'The bank invests in the community in which it operates, and is guided by its elaborate strategy. There are programmes for poverty alleviation, youth and women empowerment, skills acquisition, charity donations among others'* P#7

***Environment:*** *'The problem with loan losses in the oil and gas sector is not because of the drop-in oil prices. Banks have lent significant amount to the sector, and while the firms have licenses to operate, they are denied the social license to operate as a result of the neglect of the society, which becomes a risk issue for banks hence the risk management function needs to be alive to these issues'* P#12.

*'…boards show little interest, buy-in, and understanding in CSR, except where there are representations of foreign interests/experience or CSR experts. Boards therefore pay lip service and leave the policy and strategy at management's realm'* P#6.

*'Metrics for measuring our sustainability progress include carbon-di-oxide emission reduction, material consumption reduction such as paper, water, energy; women empowerment milestones, financial inclusion among others'* P#9. '*Our metrics cover targets set on social responsibility, environment, economic indicators, innovation, leadership and partnership, regulatory compliance, stakeholders' engagement. Cost-benefit analysis shows that embedding sustainability improves return on investment' P#11. 'SME support, and other cost-saving initiatives which in the short run may appear money-gulping but in the long run will pay for itself'* P#9. '*While we embark on initiatives to reduce consumption of water, energy, materials etc, our primary focus on these areas is about cost containment objective, not directly environmental sustainability'* P#5.

**Sustainability Enablers**: '*……more influence from the regulatory bodies such as CBN and Nigerian Stock Exchange (NSE). I am not calling for regulations because sustainability by its nature shouldn't be regulated. For NSE, they should be able to impose a requirement on every company listed on the stock exchange to have sustainability indices reported in their financials in order to create a level-playing ground. For instance, while we turn down credits because of prospective client's failure to meet the environmental test, another bank may approve the same facility and declare a much better profit.* There also *needs to be a strong buy-in from the top, and implementing sustainability as a corporate strategy rather than a stand-alone departmental function. Education is also important. People need to be enlightened that sustainability is not about philanthropy or just building bore holes in the community'* P#9.

*'While CBN is credited with introducing sustainable banking principles, the reality is that sustainability reports prepared by banks and submitted using the CBN's prescribed template are never reviewed by CBN'* P#9

***How corporate governance influences Sustainability:*** *'Corporate governance leads to sustainability in several ways. It prevents abuses such as insider abuse, fraud and expropriation, and other unethical practices. It is also a self-examination watchdog that helps the organisation to correct itself rather than being corrected by outsiders. When corporate governance fails, several stakeholders suffer losses - government, employees, customers, shareholders etc, which cuts short the sustainability of the stakeholders' P #8. 'Good corporate governance practices such as risk management checks excessive risk-taking; transparency leads to good returns on investments; ethical practices engender confidence in the organisation and a positive corporate reputation' P#7. 'Corporate governance practices allow for succession planning, performance measurement, long-term strategies, and institution of processes, structures and culture in place for continuity and long-term performance' P#6. 'Corporate governance engenders ethical practices, accountability, transparency, and avoids excessive risk-taking all leading to stakeholders' satisfaction' (Open-ended questionnaire respondents).*

# CHAPTER FIVE

## DISCUSSION OF FINDINGS

### 5.0 Preamble

This section covers the discussion of theoretical and empirical findings arising from data and results presentation in Chapter four.

### 5.1 Discussion of Theoretical Findings

This study's findings are substantially connected with the adopted theoretical framework – which combined the agency theory and stakeholder theory. For objective one (1) which sought to find the influence of internal governance controls on corporate social performance, the stakeholder theory supported the influence of the board and management structure on corporate social performance. The ownership dimension of the theory does not correspond with the stated assumptions.

Objective two (2) sought to establish the influence of ethical leadership on corporate reputation. The study's finding validates certain aspects of the stakeholders' theory, which postulates ethics amongst other integral issues in stakeholder management relationships.

Objective three (3) set out to determine the influence of regulation on financial innovation. The agency theory supports findings on financial innovation because economic regulation reduces information asymmetry between the banks and their customers who are the primary beneficiaries of financial innovation.

Objective four (4) and five (5) determined the effect of agency mechanisms on financial performance, and the moderating effects of bank characteristics respectively. This objective is the central thesis of the agency theory. Findings show a mixed result. Whilst mechanisms to align managers and owners' interests such as diversity (in form of CEO's age, board ethnicity), managerial incentive (in form of CEO's tenure), bank characteristics (in form of bank size, and bank leverage) partly supported this study's findings, the converse was the case with mechanisms such as CEOs remuneration, board gender, insider ownership, and meeting frequency.

Objective six (6) assessed the role of corporate governance practices disclosure on sustainability performance reporting. Finding in this regard further affirms the tenets of the stakeholder theory which requires business managers to be responsible, accountable and transparent.

Overall, specific aspects of the agency and stakeholder theories largely explain the findings. It should however be pointed out that there are exogenous factors that could have accounted for the discrepant results, such as the impact or a lack thereof of various institutions such as cultural, legal system, and political system which bring to light the relevance of the institutional theory. This suggests that the institutional theory could be complementary to the study of corporate governance and sustainability in a developing economy like Nigeria.

## 5.2 Discussion of Empirical Findings

### Introduction

This study adopted a descriptive and inferential research design. Objectives 1-3 were tested using administered questionnaire. Objectives 4 and 5 were based on ex-post facto design, and a content analysis of sampled banks' annual reports. Objective 6 was tested using the researcher-designed composite index for both the independent and dependent construct. Data was measured using scores derived from the content analysis of the banks' annual reports, corporate governance reports, sustainability reports, websites and other secondary documents. Interviews was also conducted to gain in-depth understanding of stakeholders' views on the different dimensions and objectives of the study, leading to a thematic analysis.

For objectives 1-3, the study adopted a three-step approach: descriptive statistics, correlation coefficient, and multiple regression analysis. The descriptive statistics provided important statistics to understand the characteristics of the sample, while the correlation coefficient was interpreted in line with decision criteria (Evans, 1996). For the regression analysis, the results satisfied tests for autocorrelation performed by Durbin-Watson statistic; and multi-collinearity performed by simple correlation, and variance inflation factors. A decision was also made to reject or accept the null hypothesis based on the significant t-statistics as expressed by the probability values (P-values) (Agbonifoh and Yomere, 1999). For objectives 4 and 5, a panel data regression was used. In addition to carrying out descriptive statistics, correlation coefficient, and panel regression, multicollinearity and

hausman tests were carried out. For objective 6, the corporate governance practices disclosure composite index was correlated, and regressed against the sustainability performance reporting composite index.

This study set out to determine six objectives as spelt out in chapter four, resulting in six major hypotheses. The results of the tests are in chapter four, while the discussion of the findings is stated below:

**Objective 1: Assess the effects of internal governance controls on corporate social performance (social and environmental performance).**

Evidence from the descriptive results (Table 4.6a-d) suggests that employees rank environmental performance (.89) more important than social performance (.79) in explaining corporate social performance. A further analysis shows that customers, community, and employees are ranked in descending order respectively in evaluating banks’ social performance. From the results in Table 4.10, there is a moderate positive (r= .479) relationship between internal governance controls and corporate social performance, which the regression table 4.12 confirms to be statistically significant at 1 percent ($P<0.001$), thus the null hypothesis that internal governance control does not influence corporate social performance is rejected. It can be deduced from the result that the more the internal governance controls are strengthened, the greater the corporate social performance is achieved.

Table 4.13 shows there is a positive and statistically significant relationship between board structure; management structure and corporate social performance at 1% significance level. The positive coefficient of .039 and .034 respectively indicate that as board structure and management structures improve, corporate social performance is enhanced in Nigerian deposit money banks. A further disaggregation of the results indicate that under board structure, board size has positive significant effect on both social and environmental performance, while board committees has significance on environmental performance only. Under management structure, CEO Tenure, and Incentive-based compensation have a positive significant influence on social, and environmental performance, while CEO remuneration has positive significant effect only on social performance. Finally under ownership structure, directors share ownership, and concentrated ownership both have significant influence on environmental performance, while employee share ownership has influence on social performance.

The above findings as supported or contradicted in literature include the following reasons:

Stuebs and Sun (2015), found that corporate governance is positively associated with corporate social performance and also has a positive impact. Huang (2013) found that if social and environmental responsibility is treated as a strategic choice, the role of the CEO is very critical as the most powerful actor in strategic decision-making. The CEO is guided by orientation, experiences, values and personality (Hambrick, 2007) and personal demographics, such as educational specialisation, (MBA and MS), gender and tenure. Having female members on the board of directors of an organisation has concrete effects on the corporate social performance strategy of the organisation (Bernardi and Threadgill, 2010). Board characteristics such as a large board size (Arani, 2016; Majeed, Aziz and Saleem, 2015; Aktaruddin, Hossain and Yao, 2009), female directors/board diversity (Carter et al, 2003), and foreign nationals (Haniffa and Cooke (2005) intensify social responsibility of firms. Barnea and Rubin (2010) found that top management teams engage in social responsibility to build their own personal reputation as good global citizens, whilst Cespa and Cestone (2007) found support for social responsibility motivated by CEOs' reliance on social and environmental activists to protect their job. Walls, Berrone and Phan (2012) concluded that the board, management and ownership structures are relevant to environmental performance.

Effective boards see corporate social performance such as environmental issues, labour and employment issues, diversity and human rights issues as a stakeholder management leverage for value/wealth maximisation. This is more so in firms with large board size comprised of members with diverse experience, education, and age mix. A large board size also affords the board to break into strategic committees for effective advisory and monitoring role. While none of the selected banks has a board committee specifically on environmental issues, there are board committees overseeing risk management and sustainability departments, thus having an oversight on social and environmental performance. The bulk of environmental activities of the banks is carried out through customers' credit application process. All the selected banks have an Environmental Social Risk Management banking application used to evaluate the environmental impact assessment on every credit application. Having legal experts on the board (as is common to all the selected banks' boards) helps to sensitise the board and management on the implication of non-compliance with environmental regulations.

Management structure influences corporate social performance among other reasons in order to avoid backlash from the media, community, employees, customers or even the government. If there is no

accountability, management would likely do without the additional costs of social responsibility investments. 'CEO tenure' and 'incentive-based' compensation motivate management to focus on social and environmental performance. Other incentives include the need for legitimacy, reputation, and utilisation of social and environmental expenditure as tax planning strategies. Although social responsibility is everyone's duty, management structure provides for a team that will anchor the process and drive awareness of social responsibility as well as ensure compliance across the bank. It is also plausible that some management engage in social and environmental responsibility for 'green washing', in which case they promote excessively the little they do, or actually put up the appearance of being socially responsible when they are not. Some organisations also restrict their social responsibility to certain locations, or activities to achieve mileage or image bragging rights.

Corporate social performance is gaining importance in the Nigerian banking industry, with a shift from the narrow lens of philanthropy to embrace wider issues such as human rights, product responsibility, financial inclusion, and diversity among others. In the environmental sphere, Nigerian banks have 30% of total credit exposure to the oil and gas sector (CBN, 2016), which is the most prominent industry facing environmental issues, and while the banks are not directly involved in activities that may significantly damage the environment, they face risk management issues (credit and reputational risks) that may arise from environmentally irresponsible practices of their credit customers. Management support for socially responsible issues may also be viewed from the cost-benefit consideration as opined by interview respondents in this study. Another conceivable explanation for support of social responsibility by management and/or board is the potential to use it as a conduit pipe to funnel out resources and assets of the bank. Social responsibility investments (especially philanthropy and charitable donations) are rarely accounted for in terms of receipts/invoices documentation. There is barely a mechanism to match value for money spent, therefore accounting for social responsibility may be considered weak in the banking industry, and therefore provides opportunity for greedy management and board to utilise social responsibility as a vehicle to award contracts to themselves or their cronies.

Directors' share ownership and concentrated ownership both have significant effect on environmental performance, whilst employee share ownership has significant influence on social performance. As managerial and concentrated ownership increases, it is expected that the level of risk-taking will decrease, thereby creating opportunities for investment in socially responsible practices. In the Nigerian

context, interview respondents regard the institutional investors as retail investors, with the bulk of their investments coming from government pension, hence their minimal or no oversight influence over management activities. Furthermore, they vote with their 'feet rather than their voice' to influence bank practices. The combination of these two (2) factors imply that the ownership structure from the institutional perspective plays little or no role in corporate social performance strategy of the bank, perhaps because institutional investors are likely to see investment in social responsibility as a waste of resources. This is contrary to findings by Harjoto and Jo, (2011), Ho et al. (2011), and Cox, Brammer and Millington (2004) who found that institutional investors prefer to invest in firms with positive social responsibility and because of their strong influence on the board, are able to steer the strategic direction of the firm towards social responsibility practices.

**Objective 2: Examine the extent to which ethical leadership influence corporate reputation**

Evidence from the descriptive results (Table 4.5a-4.5c) suggests that respondents rank ethical leadership components in the following order of importance – ethical program, ethical culture, and CEO's personal ethics.

From the results in Table 4.14, there is a moderate positive ($r$= .468) relationship between ethical leadership and corporate reputation, which the regression table 4.16 confirms to be statistically significant at 1 percent ($P<0.001$), thus the null hypothesis that ethical leadership does not influence corporate reputation is rejected. It can be deduced from the result that the more the ethical leadership is strengthened, the greater the corporate reputation is achieved.

When the result (model) is further deconstructed, Table 4.17 shows there is a positive and statistically significant relationship between ethical program; ethical culture and corporate reputation at 1% and 5% significance level respectively. The positive coefficient of .613 and .132 respectively indicate that Nigerian deposit money banks desirous of improving their corporate reputation need to strengthen their ethical program and build an ethical culture, with ethical program contributing the most influence on corporate reputation. Through these mechanisms, banks can attain a reputation for quality of their products/services, financial soundness and global appeal. As the mechanisms improve, corporate reputation is enhanced in Nigerian deposit money banks. The result however shows a positive relationship between CEO's personal ethics and corporate reputation, but not a statistically significant effect.

The above findings as supported or contradicted in literature include the following reasons:

The findings are supported by Roberts and Dowling (2002) on the premise that organisational ethics contribute to positive relationships between a firm and its external stakeholders, thereby enhancing firm reputation. Similarly, Chun, Shin, Choi and Kim (2011) and Zhu, Sun and Leung (2013) respectively posited that corporate ethics through the mediating role of corporate reputation lead to financial performance, and ethical leadership moderated an indirect positive effect of social responsibility on firm performance through firm reputation when ethical leadership was strong. Walker and Dyck (2014) found that all stakeholders (respondents) placed greater emphasis on the criterion of ethicality than the criterion of profitability for rating a firm's reputation. Stephenson (2004) found that more women on the board results in a major increase in the use of non-financial performance measures, such as innovation and social and community responsibility, a similar view shared by Williams (2003) that having women on boards of directors was positively related to firms' corporate philanthropy.

Banks in Nigeria are mandated by the regulatory authority (CBN) to have codes of ethics for employees and directors, as well as compliance officers at top management level to report on unethical practices. Banks also give out copies of ethical codes to every employee, and update their intranets with modifications to ethical codes. In addition, the regulatory authorities also have disciplinary power to sanction unethical practices, which may explain the importance of ethical programs in building ethical culture in the banks. This is complemented by setting the right tone at the top. As some of the respondents opined with these statements: *'there is zero tolerance for unethical practices'; 'our foreign investors are not just concerned about the end, but the means to the end as well, especially how targets are met, they query management when media breaks out any news linking the bank to any criminal or fraudulent customer' and 'Leadership in the bank is highly ethical. I have not seen where the MD stalls an investigation'*

Whilst the findings show a positive relationship between CEO's personal ethics and corporate reputation, a non-significant effect is established. The CEO has a critical responsibility in the organisation to build a strong ethical culture supported by ethical programs. The external stakeholders - public, government, media, and even the internal stakeholders - employees, customers form an impression of a bank's brand based on what they perceive. Surveys (Weber Shandwick, 2015; Burson-

Marsteller, 2003) show that 49% of a firm's reputation is tied to the CEO. In further support of this study’s findings, Roberts and Dowling (2002) found ethics affecting reputation to enhance firm performance. Walker and Dyck (2014) found that the reputation of firms with a primary focus on profits was rated lower than firms with focus on corporate social responsibility, and that all stakeholders placed great emphasis on the criterion of ethicality. Wu, Kwan, Yim and He (2015) found that CEO ethical leadership positively influences social responsibility through ethical culture. De Hoogh and Den Hartog (2008) also report that leaders high on social responsibility were rated higher on ethical leadership.

Ethics, corporate reputation and social responsibility are intertwined, and common to the three variables is the issue of fairness, and justice to stakeholders. Being strong on social responsibility is a means of improving a firm’s reputation. Social responsibility means taking cognisance of different stakeholders and not just the shareholders. A bank’s perspective may be gleaned from the personality trait of the CEO and the influence of the Board in driving the organisational culture. Social responsibility may be considered by some board members as an idealistic view, which may not appeal to executives/directors who make decisions based on a careful consideration of facts, reliable data, logic, and entrepreneurial mindset rather than emotional feelings. On the contrary, there are board or executive management members who do not treat decision-making with cold calculations, and both categories of personality traits are needed to achieve an equilibrium in the functioning of an organisation. The bank’s direction on social responsibility would therefore depend on the ability of the latter group to exercise influence over the former group.

The situation in Nigerian banks however is that there is no (visible) rating agency evaluating corporate reputation or CEOs reputation officially. Furthermore, CEOs in Nigerian Banks are generally chaperoned in their external engagements, whilst restricted in their internal engagements mostly to top management, thus information about CEO’s personal ethics is therefore scarce and largely controlled. This point was aptly put by Trevino, Brown and Hartman (2003) who identified the role of being at a vantage point to truly gain a proper perception of ethical leadership, suggesting that in order to receive a perception of being ethical, the executive needs to step out of the executive suite and engage in behaviour that make them stand out as an ethical figure.

Each Nigerian deposit money bank has a corporate affairs department or its equivalent saddled with the responsibility of putting out the fire on bad press, and they dutifully do whatever is required to avoid negative press, especially in an environment where poverty and greed (as described by interview respondents) dictate the slant of media reporting. With the increased influence of online and social media however, negative and sometimes fake news can easily be spread, or stories slanted to portray a wrong impression of reality. The legal system is also not very strong to encourage wronged party to sue, as cases often run for years unresolved. These factors therefore may account for why it is difficult to get an objective evaluation of the CEOs' personal ethics and influence on reputation. The cultural values of greed and poverty, and the role of social media have important roles in forming opinion about a bank's corporate reputation. Ethical leadership of the CEO must however be backed up by organisational ethical programs to have a significant influence on firm performance (Mayer *et al.*, 2009). Ethical programs consist of documented ethical codes and policies, training, reward and sanction systems among others.

**Objective 3: Evaluate the role of regulation on financial innovation**

Correlation results in Table 4.18 show there is a moderate positive (r= .500) relationship between regulation and financial innovation, which the regression results in Table 4.20 confirm to be statistically significant at 1 percent (P<0.001), thus the null hypothesis that regulation does not influence financial innovation is rejected. It can be deduced from the result that the more the regulation is strengthened, the greater the financial innovation achieved.

When the result (model) is further deconstructed, Table 4.21 shows that quality of supervision (at 1%) had the most statistical significance in predicting financial innovation, followed by capital requirements (at 1%), both with positive coefficients, while activity restriction had a negative coefficient but statistically significant (at 5%). The positive coefficients of .613 (quality of supervision) and .132 (capital requirements) indicate that Nigerian deposit money banks desirous of improving their financial innovation need to adapt and be proactive in their strategies towards regulation and supervision. The result also shows that both disclosure, and entry and exit regulations have positive coefficients, while deposit insurance has negative coefficient. The three variables however do not make a significant contribution in explaining financial innovation.

The above findings as supported or contradicted in literature include the following reasons:

Mwangi (2007) found that laws protecting investors are some of the factors influencing financial innovation in Kenya.

Innovation can promote economic growth or cause systemic distress as experienced with the corporate scandals in the early twenty-first century. Nigerian banks are highly regulated by CBN, and elements of regulation with impact on financial innovation include the requirement for banks to regularly provide tailored reports, automate some business processes (for instance risk assets/risk management), report on new products and services and receive approval before implementation. The fact that Nigerian banks offer basically homogeneous products and services, and that CBN intervenes from time to time in the interest of customers through reduction of various bank charges and other unfair trading practices means banks have to find ways to gain competitive edge. Similarly, disclosure and reporting requirements would at least mean potential investment in innovation etc; unfortunately on the other hand, bank examination and inspection by CBN examiners may also hinder innovation as the skills level of examiners still require improvement hence they insist on hardcopies of reports rather than a soft copy during their audit/examination. CBN examiners also check banks' exposure to additional risks due to financial innovation to ensure that the risks are recognised, evaluated and managed, but given the relative knowledge gap of the regulators, the level of check and value of this exercise is limited. Furthermore, CBN influences innovation through granting of tax exemption for expenses on innovation, recognition and award for innovation implemented across the industry, and special status for players perceived as leaders in innovation. Barriers on entry and exit into the banking industry may be an opportunity for portfolio investors to have a stake in the existing banks, and thus enhance innovation. Since the completion of consolidation exercise in 2005, the number of Deposit Money Banks has shrunk from twenty-five (25) to twenty-two (22). In addition, the foreign exchange restrictions may hinder payments settlement and prevent banks from importing technological innovations, thus over regulation and restrictive policies hinder innovation.

**Objective 4: Ascertain the influence of agency mechanisms on financial performance**

From the panel regression results (Tables 4.27a - 4.27e) there is a statistically significant effect of agency mechanisms on financial performance. Specifically, Board Ethnicity (LBETHN) had positive coefficient and statistically significant effect at 5% on ROA and TBQ as measures of financial performance. CEO's age (CEOA) had positive coefficient and statistically significant effect on ROA

and ROE, but a negative coefficient and statistically significant effect on NPL at 1% significance level. Insider ownership (LINO) had a negative coefficient but statistically significant effect on ROE. CEO's remuneration (LCEOR) had a negative coefficient but statistically significant effect on TBQ and NIM. The results thus indicate that Board ethnicity, CEO's age, CEO's remuneration and Insider ownership play significant role in influencing the financial performance of Nigerian Deposit Money Banks.

The result however shows that board gender while not statistically significant at 5%, had negative coefficient and impact on all measures of financial performance. Board intensity proxied by total meeting frequency (at full board and committee levels) appear not to play significant role in explaining the financial performance of Nigerian deposit money banks. It had a negative coefficient on ROA, and positive coefficient on ROE (at 10% significance level). Similarly, CEO tenure, showed non-significant effect on financial performance with a negative coefficient on ROA, and ROE, and a positive coefficient on TBQ, NIM, and NPL.

The above findings as supported or contradicted in literature include the following reasons:

ETHNICITY (LBETHN): In support of this study's findings, Olaoti (2016) found ethnicity of board of directors to have a positive and significant impact on Nigerian deposit money banks' financial performance. On the contrary though, Ujunwa (2012) found ethnic diversity was negatively related to firm performance but not significant; Carter *et al.,* (2010) found no significant relationship between the ethnic diversity of the board and financial performance.

Within the Nigerian banking industry context, there is strength in having birds of the same feather flocking together, which explains the effect of ethnicity on performance. Members are able to engender trust on account of ethnicity. The culture in the society supports people of the same geographical section (fraternity) coming together, rather than breeding mistrust and rancour that comes from a forced quota system that operates in the country or tokenism of ethnic diversity. The strength of cultural affinity allows for misunderstandings and quarrels at board level to be settled if need be at clan meetings. This is what Byrne (1971), cited in Talavera, Yin and Zhang (2018) referred to as 'similarity-attraction paradigm', where individuals are unwilling to share information with people from other ethnic groups viewed as outsiders, making it difficult for interpersonal bonding.

CEO Age (CEOA): In support of this study's findings, Peni (2014) found that CEO age has a positive impact on ROA suggesting that as CEO's age increases, the ROA seems to increase. Bertrand and

Mullainathan (2003) however found that older executives may be more prone to advancing their own goals and ‘‘enjoying the quiet life’’, which may cause a lower performance of their firms. Bernasek and Shwiff (2001) report a negative relationship between age and risk aversion, that is, as the CEO gets older, the less risk he takes and vice-versa, which could create differences in firm performance based on the age of the executive, a view also shared by Bertrand and Schoar (2003) which suggests that older executives are more conservative in their work, which may have an impact on firm performance. Davidson *et al*. (2007) also found a negative relationship between executive age and firm performance.

Findings suggest that as the CEOs age increases, ROA and ROE increase, which ordinarily could be explained as experience and industry knowledge. This is contrary to respondents’ opinions, which believe that in the Nigerian banks’ context, younger CEOs demonstrate the energy and drive to push for strong performance. At their youth they are interested in building a reputation for themselves in the industry. They are also able to take risks, they read more or build on their knowledge through foreign schools and certification and are more likely to try new ways of doing things rather than being conservative. It is contended that they took over the management of the deposit money banks from the older founders who ran the affairs of the banks till December 2005 (the period before consolidation of banks), and can be credited with introducing a lot of new ideas into banking in Nigeria. They assert that young CEOs encourage and embrace innovation, while older men have less drive, are geriatric, risk-averse and appear to have less understanding of trends to drive harder, stronger performance, but more importantly there are fewer of them available as bank CEOs. Descriptive statistics shows that the average age of selected banks’ CEOs is 48 years old (minimum 34, maximum 58). The inverse relationship between CEOs age and non-performing loan, which means as the CEOs age increases, non-performing loan decreases may buttress the argument about older executives being conservative and more risk averse. A supplemental sensitivity analysis carried out on the results using dummy variables shows that CEO-age at 54years and above had a positive significant effect on financial performance (appendix H), while other age categories below 54years were insignificant.

TOTAL MEETING – (TM). In support of this study’s finding, Jiraporn *et al.* (2008) and Ahn *et al.* (2010) found executive busyness tends to decrease firm performance, while in a slight departure Fernandes and Fitch (2009), and Aebi *et al*. (2012) did not find significant effects in their studies of the relationship between board busyness and performance (as this study found only significance in one of

the five measures of financial performance). Contrary to this study's findings, Ntim and Osei (2011) found a positive relationship between frequency of meetings and firm performance.

Selected banks' board meetings are averagely held about four times annually, with board committee meetings taking the rest of the board intensity. The most frequent meeting comes from the credit or risk management committee, and mostly to approve credits above the executive management's obligor limit. At other times, the full board meets to attend to emergency or crisis situation, while the audit committee, which is very critical to ensure efficient utilisation of resources and control over processes meets averagely about four times annually, which may suggest that meetings are largely not much about rigorous strategy and performance review but matters routinely reported by management.

CEO REMUNERATION – (CEOR). In line with this study's findings, Aduda (2011) and Nyaoga (2014) found negative and significant relationship between remuneration and firm performance, buttressed by Cooper, Rau and Gulen (2009) who posited that a high pay results in overconfidence of CEOs. Similarly, Zalewska, (2016) posited that the use of remuneration to reduce agency problem is sub-optimal for the banking sector because of the conflict of interests (separation of ownership and control) between the shareholders and other stakeholders.

In a clear departure from this study though, Kurawa and Saidu (2014), Fald Al-Heizan (2011), and Sigler (2011) found positive and significant relationship between executive compensation and financial performance, while Shah, Javed, and Abbas (2009); Hussain, Obaid and Khan (2014) found there is non-significant association between executive remuneration and firm performance.

A poorly structured remuneration policy not tied to long-term and multi-dimensional performance is counter-productive. Nigerian banks' CEOs receive as compensation mainly cash salary and allowances (guaranteed irrespective of performance), and bonuses. A CEO may receive bonus and reward for this year's performance and under-perform next year, thus the remuneration may bring a lagged influence on subsequent performance. Some form of remuneration can also only achieve little in influencing performance, as the perks of office are normally generous relative to Nigeria's socio-economic environment. In addition, there is no evidence from the review of annual reports that CEOs remuneration is disclosed to shareholders at AGM, or subject to their approval. Further analysis of pay ratios between a selected bank CEO and the average employee is as high as eleven-to-one (11:1). Even though this study found the existence of board remuneration committees in selected banks, there is no evidence to suggest that executive pay is independently and rigorously designed. Respondents affirmed

that external consultants are engaged to carry out industry survey and make recommendations to the board remuneration committee. This negates the objective of tying pay to performance, as surveys may be flawed in masking extraneous variables.

The increasing deterioration of non-performing loans (CBN, 2016) shows the less-than-conservative approach adopted by banks in creating risk assets. Deposit money banks are engaged in significant exposure to oil and gas industry for instance, while risk management is still viewed as a departmental responsibility. As respondents opined, *'people making credit decisions at the board level are rarely knowledgeable about the intricacies and therefore rely heavily on what management presents to them'* and since income is earned mainly from creation of risk assets, CEOs are generally bullish towards this objective. In the process, questionable credits are approved, resulting in bank income boost, and consequently enhanced CEO remuneration. Two years down the line, asset delinquency sets in, resulting in mandatory prescription by CBN for a full risk provision, yet no remedial action is taken by the board to reduce the CEO's pay and recover the lost credit. This shows the effect of excessive risk-taking, and when juxtaposed with the finding on influence of CEO's age on non-performing loan, it can be established that younger CEOs exercise less due care in risk-taking.

INSIDER OWNERSHIP (LINO): In line with this study's finding, Demsetz (1983) found inverse relationship between firm performance and increasing insider ownership, Iturralde, Maseda and Arosa (2011) found the optimal insider ownership level to be between 0-35 percent for improved firm performance, beyond which performance begins to decline due to the entrenchment risk. In contrast, Abor and Biekpe (2007) found that inside ownership and family ownership have significant positive impacts on profitability among SMEs in Ghana. The characteristics and performance metrics of an SME are however quite different from a large listed company such as deposit money banks.

Insider owners protect their interests to the detriment of other shareholders. As some respondent states, '*they cream the profits, and take their share of value above the line rather than below the line*'. Where dividends are not forthcoming, neither is the share price encouraging, they are left to cater for their interests depending on their level of executive recklessness, and selfish motivation. They may also make decisions, and vote in favour of decisions that will benefit them individually rather than what benefits the organisation. Abuse is inevitable, as business decisions may rest in the hand of few people,

which may encourage insider abuse and insider-related cases known to have led to collapse of several banks in the past.

A supplemental sensitivity analysis carried out on the results using dummy variables shows that Insider Ownership (if) pegged at an aggregate of 2% had a positive significant effect on financial performance –ROA and ROE (appendix H), while other categories above 2% were insignificant.

BOARD GENDER – (BGDR): In line with this study's findings, Ujunwa (2012), Akpan and Amran (2014) found board gender has negative (significant) effect on financial performance, while in a departure from our findings, Campbell and Minguez-Vera (2008), Carter, Simkins and Simpson (2003), Krishnan and Park (2005); and Smith et al. (2006) suggest that the gender diversity of the board has a positive impact on firm value. Randoy (2006) and Gregory-Smith (2013) found no evidence to support the argument that gender diverse boards enhance corporate performance. Having female representation on the board of banks is a recent initiative introduced by CBN as part of the sustainable banking principles, even though there is no mandatory percentage prescribed. Banks are however required to disclose the percentage of women represented at senior and executive management positions. This study found female representation to be very low at board level, with a mean of 11.67%, which may account for the insignificant influence on financial performance. Richard, Barnett, Dwyer and Chadwick (2004) found that a management team must either be homogenous or highly diversified to influence performance. Where there are only a handful, they may be subject to stereo-typing and therefore find it difficult to convince the male-dominated board. Female board members are also found to perform better on soft-issues such as social responsibility rather than financial performance. It is also plausible that despite the education level and proven competence of women, the societal culture may not have yet come to terms or accept wholeheartedly the importance or ability of women to be significantly represented in banks, which are unique for their intense competition and high-energy level. Female members may be assumed not rugged, nor daring for hazardous endeavours. They may also be considered vulnerable, even though they are known to pay more attention to details which ensures decisions are more robust.

CEO TENURE – (CEOT): This study finds a mixed result on association of CEO's tenure with performance. Baysinger and Hoskisson (1990) posited that the longer an executive director stays in a

position, the more of firm and industry knowledge s/he is expected to acquire which should lead to an improvement in the firm performance. Kyereboah-Coleman (2007) stated that a guaranteed tenure provides job security to the CEO, as well as the benefit of being able to see the outcome of implemented strategies. Nigerian banks' CEOs generally have a guaranteed tenure based on their contracts (which may be between 3-5 years, subject to a total maximum of 10 years), and are rarely fired, except by CBN in the case of fraud or significant unethical practices, hence tenure may not be an influential factor in determining performance.

In contextualising financial performance of deposit money banks in Nigeria, it is important to note the roles played by structural distortions and arbitrage opportunities. For instance, while intermediation is a key role of the banks, the fluctuation in foreign exchange market, floatation of government bonds, and macro-economic uncertainties are variables that create avenues for banks with government connection/leverage to tap into these opportunities for random profit-making. As a respondent opined, '*there is no level-playing field with regards to foreign exchange access through CBN, thus benefitting some banks to the detriment of others*'. As stated by another executive in one of the deposit money banks, '*if the government is issuing Treasury Bills at 18% or 19%, by definition, the after-tax yield on all of those things is already in the 20s, about 24% or 23%. Now why do I want to start exposing myself to companies and take the credit and liquidity risks when the after-tax yield is returning at the same level?*' (Africareport, 2017). This assertion conveys the mindset of deposit-money bank executives towards their primary role concerning profit maximisation objective, thus banks leverage on the high-yield environment of government securities to increase their interest income. Similarly, large foreign exchange rate movement (such as experienced in 2016) from \$/N199 to \$/N305 led to substantial foreign exchange revaluation gains which could have distorted financial performance.

**Objective 5: Determine the role of bank characteristics in moderating the influence of agency mechanisms on financial performance**

From the panel regression results (Tables 4.30a – 4.30e) there is a statistically significant moderating effect of bank characteristics on the influence of agency mechanisms and financial performance. Specifically, bank size, proxied by total assets (LTA) had negative coefficient but statistically significant effect on TBQ (at 1% level), and NPL (at 10%), while bank leverage (LFLV) had positive coefficient and statistically significant effect (at 1% level) on both ROE and TBQ, but negative

coefficient with statistical significance on NPL (at 10%). The results indicate that bank characteristics play significant role in moderating the influence of agency mechanisms on the financial performance of Nigerian deposit money banks. The result however shows that bank age (LFA) was not statistically significant to explain financial performance of deposit money banks in Nigeria.

The above findings as supported or contradicted in literature include the following reasons:

**Bank Size:** Large banks may have reached their maturity and therefore lose out on economies of scale, whereas small-sized banks have higher growth rate (Hu and Izumida, 2008).

**Bank Leverage:** Penman and Penman (2007) held that leverage puts pressure on firms to achieve corporate performance.

**Bank Age:** An older bank is expected to have built reputation in the market, developed facilities and infrastructures, and gained substantial industry knowledge compared to a new or emerging bank with less experience, and higher cost structure (Gregory, Rutherford, Oswald and Gardiner, 2005; and Boone, Field, Karpoff and Raheja, 2007; Baker and Kennedy, 2002), while Evan (1987) believed that age is counter-productive to firm development.

**Objective 6: Evaluate the impact of corporate governance practices disclosure on banks' performance sustainability reporting**

From the results in Table 4.34, there is a moderately positive ($r$= .461) relationship between corporate governance practices disclosure and sustainability performance reporting, while the regression results in Table 4.36 shows the effect to be statistically significant at 5 percent ($P<0.05$), thus the null hypothesis that corporate governance practices disclosure does not influence sustainability performance reporting is rejected. The positive coefficient of .616 indicates that as corporate governance practices disclosure improves, sustainability performance reporting is enhanced in Nigerian Deposit Money Banks.

The above findings as explained from literature include the following studies:

In support, Janggu, Darus, Zain and Sawani (2014) established a relationship between corporate governance and sustainability disclosure. On the other hand, Aras and Crowther (2008) stated that it was not possible to demonstrate a relationship between governance and sustainability, because a greater

understanding of the issues by respondents is required, thus recommending for more information to be disseminated about sustainability and corporate governance.

A summary of sustainability performance reporting shows that all the selected banks placed more reliance on their economic performance than social or environmental performance. There was a generally above-average performance except for BK#3 and 4. BK#6 dominated leadership position in most of the financial performance indicators, closely followed by BK#8. From the market indices, only BK#6 had a TBQ ratio above 1, meaning it's the only bank earning a rate higher than its replacement cost, which implies that the bank is trading overvalued, which is positive for the shareholders. Non-Performing Loan ratio was poor for most of the banks, except for BK#1 at a rate below 2%. Under the social performance category, all the banks except three had employees' attrition, which was considered by respondents as a source of concern with regards to job security in the industry. Performance towards customers and product responsibility was generally poor. This manifested in the volume and value of customer complaints. Total volume and value of customers' complaints in the eight selected banks for 2016 was 1,334,190reported complaints, translating to a refund claim of N179billion. In addition, there was no evidence of ethical marketing disclosure except BK#5. On Community Development (CD), some banks had clear-cut strategies targeted at attaining some of the SDGs, while some still focused on philanthropy and charitable giving. None of the banks had a policy to set CD spending at a percentage of profit. The study however observed that CD spending reduced significantly when operating profit dropped e.g. BK# 2, 3, 5. On diversity, only two banks (BK#1 and 6) showed evidence of commitment via a set target on gender parity ratio over a specified period. Environmental performance was generally poor for the selected banks. There was evidence of largely tokenism in areas of resource efficiency/management – energy, water, waste, emission – with most of the banks engaged in similar routine initiatives, with only a few instances of differentiated strategies. Most initiatives are also prominently implemented in Head-Offices. It is apparent that most of the selected banks engaged in environmental initiatives that only bring about cost reduction. The study also observed that there was no evidence that the sustainability reports sent to CBN monthly were being scrutinised or reviewed in details by the regulators. As gathered from the interview, banks adopt different reporting standards, which makes performance comparison difficult, if not impossible. In addition, the low level of credit transactions passing through the banks' Environmental Social Risk Management System translates to an insignificant effect on environmental sustainability. One implication of this is that a loan/credit

application declined in a meticulous bank may be approved and granted in another less painstaking bank, thereby creating a contoured playing ground for practitioners. Nevertheless, the regulatory backing for sustainable banking in Nigeria explains the disclosure and reporting of these items.

**Qualitative Findings**

Objective 1:

Internal governance controls – According to respondents, the selected banks have imbibed social responsibility practices in their business. Awareness of environmental issues ranks high because of the Environmental and Social Risk Management (ESRM) System instituted to screen credit applications and reject socially and environmentally non-compliant applications. All sampled banks engage in philanthropic activities to a varying degree. In addition, management of the banks see a motivation towards social and environmental responsibility first and foremost from the apparent short and long-term benefits in cost reduction. Boards however have minimal influence on environmental performance, even though social issues, especially relating to employees' welfare is reviewed as a KPI.

Objective 2:

According to respondents, ethical culture is strong in banks, and there are ethical programs to institutionalise ethical culture. Board members and employees sign ethical codes. In addition, regulatory authorities play key role in enforcing sanctions for observed unethical practices.

Objective 3.

Respondents believe that regulation mechanism has improved in quality of supervision, introduced risk-based audit and recognised the need for involvement in managing the risks associated with financial innovation (new products, new markets and technology). The introduction of sustainable banking principles by CBN, and a focus on one of the pillars –financial inclusion is seen by respondents as a major driver for financial innovation in deposit money banks.

Objective 4:

According to respondents, board influences financial performance through strategy and performance target setting, oversight function, and budget reviews, but not as resource providers. Management structure is the most critical mechanism to influence financial performance. Insider ownership is the

most influential form of ownership in banks. In addition, structural distortions in the financial system provides a control variable in explaining financial performance of banks.

Objective 5:

Respondents expect bank age to have a mixed effect on financial performance. In one breadth, it is considered a disadvantage because of the perception of being old, archaic and not nimble to maximise market opportunities. In another breadth, it is considered a benefit because of the goodwill enjoyed over time and the patronage of having a ‘captured-market’. Bank Size is also viewed with mixed opinions. With a large portfolio of total assets, respondents expect their banks to have opportunities for greater income-yielding opporunities, but on the other hand, the risk management mechanism may not be robust and sophisticated enough to track effectively and completely the assets created, thus giving room for non-performing loans and consequently reduction in income. Leverage is considered by participants to be unavoidable in the industry in order to enhance income-generating potentials.

Objective 6:

Study participants believe that the sampled banks have mechanisms for measuring their sustainability albeit at different level of sophistication. They assert that banks disclose their corporate governance practices and sustainability performance reporting mainly because of the regulatory requirement for transparency, and fear of regulatory penalty for non-compliance. Respondents also opined that there is inconsistency in reporting format by banks, whilst the regulatory authority does not show evidence of review or oversight on the sustainability performance reports. This creates opportunities for some banks to take-on and approve customer loan applications already rejected by other meticulous bank(s) on grounds of non-compliance with Environmental Impact Assessment.

# CHAPTER SIX

## CONCLUSIONS AND RECOMMENDATIONS

### 6.0 Preamble

This section presents the summary of the entire research work, conclusions and recommendations, with a view to drawing practicable policy implications for Deposit Money Banks in the area of corporate governance and sustainability performance.

### 6.1 Summary of the Study

The overarching objective of the study was to examine corporate governance dimensions and their influence on sustainability.

In specific terms, the study sought to:

i. assess the effects of internal governance controls on corporate social performance in selected deposit-money banks in Nigeria.
ii. examine the extent to which ethical leadership influence corporate reputation.
iii. evaluate the role of regulation on financial innovation.
iv. ascertain the influence of agency mechanisms on financial performance
v. determine the role of bank characteristics in moderating the influence of agency mechanisms on financial performance
vi. evaluate the impact of corporate governance practices disclosure on banks' performance sustainability reporting.

Chapter one provided a background to the study, and the statement of research problem. It went further to explore the objectives, and the generated research questions which are: To what extent do internal governance controls influence corporate social performance? What effect does ethical leadership have on banks' corporate reputation? To what degree does regulation influence banks' financial innovation? In what way do agency mechanisms influence financial performance? To what extent do bank characteristics moderate the influence of agency mechanisms on financial performance? To what extent does corporate governance practices disclosure enhance sustainability performance reporting by banks?

Six hypotheses were built in null form in line with the research questions. The scope of study was restricted to Head-Offices and Lagos branches of the selected banks – eight (8) publicly listed deposit money banks in Nigeria, which include: Access Bank, Diamond Bank, FCMB, Fidelity Bank, First Bank, GT Bank, UBA, and Zenith Bank. These banks were selected based on a set of criteria, such as being publicly listed, Nigerian-owned, in operation before the banking consolidation exercise, and still retaining its business name, and not being a candidate of restructuring by the apex bank (CBN). The chapter also identified the significance of the study to several stakeholders, and presented a schematic model and definition of operational terms.

In chapter two, the concept of corporate governance dimensions and its influence on sustainability was examined. Subsequently, the conceptual framework, theoretical perspectives and empirical findings were thoroughly reviewed. The chapter identified gaps in literature (which formed the basis for the statement of the research problem), and also introduced the theoretical framework adopted for the study, which consisted of the agency theory, and stakeholders' theory.

Chapter three presented the research design, procedures for data gathering, population, sample size, sampling techniques, sampling frame, research instruments, and the techniques adopted in analysing the data. It also addressed validity and reliability of methods adopted, and ethical considerations among others. In this study, the descriptive and inferential designs were adopted. The justification for these designs was to secure a better in-depth understanding of the phenomenon under investigation (Denzin and Lincoln, 1994; Maxwell, 2005), as well as to establish causal relationships between the two constructs i.e. corporate governance dimensions and sustainability in order to gain a clearer view of the relationship (Saunders et al., 2009). In addition, the study also adopted Ex-post facto approach to explain the effect of the independent variables (agency mechanisms) on dependent variables (financial performance) based on precursor conditions and therefore was able to test the proposed hypothesis using statistical tools (Kerlinger and Rint, 1986; Cohen, Manion, and Morison, 2000; Simon and Goes, 2013), and a mixed-methods approach to overcome the weaknesses identified in either strictly quantitative or strictly qualitative study (Bryman, 2012) among other reasons. Population was estimated as fourteen thousand one hundred and forty-seven (14,147), which translated to a sample size of 771 using Krejcie and Morgan's formula, while a multi-stage sampling technique involving proportion-to-size, stratified, and purposive method was adopted.

Chapter four presented the data and results of the various statistical tests. 771 questionnaires were administered, out of which 573 (representing 74%) were returned and found usable. Descriptive statistics showed information about the 573 respondents along demographics such as gender, age, marital status, education, job function, job position, and years spent in their current bank. The study also showed the responses to the questionnaire adopted for objective 1-3 (in tables 4.3a-4.9), along two reporting formats – individual bank choice, and Likert-scale choice. For objective 4 and 5, the descriptive (in table 4.24 and 4.29) showed common statistics – mean, standard deviation, minimum, and maximum for the variables considered using secondary data and panel regression. For objective 6, the descriptive (in Table 4.33) showed the banks' statistics score (mean, minimum, maximum, standard deviation) in the composite index built for the corporate governance practices disclosure (independent variable) and the sustainability performance reporting index (dependent variable). Thematic analysis of the interview transcripts (in table 4.40) which identifies nine (9) major themes was also provided in this chapter. Statistical tests conducted include descriptive (mean, standard deviation, minimum, maximum), inferential (correlation coefficient, multiple regression, panel data regression), while software used in this study included PASW (formerly IBM SPSS V23), IBM SPSS Amos V22, and Eviews V9. The tests of hypotheses revealed that all null hypotheses (1-6) were rejected while the alternative hypotheses were accepted.

Chapter five provided discussions on the findings from chapter four. For each finding, the researcher listed evidence from literature to back up or contradict the findings, while in addition discussed plausible explanations for the findings. Findings show that internal governance controls of board and management structure have positive, significant effect on corporate social performance, while ownership structure was positive but not significant. Certain elements of ethical leadership (ethical program, and ethical culture) were found to have positive significant effect on corporate reputation, though CEO's ethics was found positive but not significant. Components of regulation (quality of supervision, and capital requirements) have positive significant influence on financial innovation, while another (restriction of activities) has a negative influence on financial innovation. Other findings show that elements of agency mechanisms (board ethnicity, and CEO Age) have positive significant influence on some proxies of financial performance (ROA, ROE, TBQ), while CEO's remuneration (against TBQ and NIM) and insider ownership (against ROE) have negative significant influence on

financial performance. Corporate governance practices disclosure also had a positive significant influence on sustainability performance reporting.

The surprising results include the negative (though not significant) effect of board gender (female) on all proxies of financial performance; the non-significant (but positive) effect of CEO's personal ethics on corporate reputation; as well as ownership structure on corporate social performance.

## 6.2 Conclusion

Based on the findings from the test of hypotheses, the study concludes that:

i. Internal governance controls have positive significant influence on corporate social performance.
ii. Ethical leadership has positive significant effect on corporate reputation.
iii. Regulation enhances positively and significantly financial innovation.
iv. Agency mechanisms positively and significantly influence financial performance.
v. Bank charcteristics moderate positively the influence of agency mechanisms on financial performance.
vi. Corporate governance practices disclosure has significant influence on sustainability performance reporting.

This study has provided empirical evidence that sustainability of deposit money banks is achievable through an effective corporate governance system anchored on a consideration of responsibility to not only the shareholders, but all relevant stakeholders. This requires a foundation based on strong internal governance mechanisms and complemented by ethical leadership and regulation. The strategic input of the board, and effective implementation by the management enhance a deposit money bank's corporate social performance. Satisfied employees, customers, community and the environment repay banks' social responsible activities by their loyalty, continuous patronage, government recognition and incentives. Banks that consciously work on being ethical through the institution of ethical programs, ethical culture and setting the right tone at the top are able to attract employees, customers, and investors, as well as commanding premium prices, thereby enhancing their corporate reputation. As banks also seek to gain a competitive advantage through financial innovation, regulators indeed have a critical balancing role to play, in providing supportive laws and incentives on one hand, whilst protecting the customers against exploitation (in particular), and the industry against systemic collapse

(in general) on the other. Other gains of corporate governance include superior financial performance resulting from business strategies woven around the interdependency of the board, managerial incentives, and ownership mechanisms. The capacity is further enhanced by a bank's ability to maximise the potentials from its internal strengths in form of assets, age, and leverage to achieve economies of scale, industry knowledge, and high growth rate. Similarly, disclosure of banks' governance practices engender accountability and transparency, which ultimately provides the stimulus for sustainable performance. If all these programs are implemented, deposit money banks have a formula for attaining sustainability.

## 6.3 Recommendations

In line with the study's findings, we propose the following recommendations:

**Regulation and Financial Innovation:**

i. Bank boards should have a dedicated committee in charge of IT, and also have key resource members with cognate IT skills and competencies. In addition, the board should also develop a robust risk management framework that specifies an overall risk appetite, risk tolerance, processes and procedures, trial phase for new products, and a monitoring, risk-reporting and compliance processes for all forms of financial innovation.

ii. CBN as the regulatory authority should ensure that its supervisory function keeps up with innovation through skilled supervisors, requisite training, and recruitment drive. In addition, there should be a regulatory risk management framework and appropriate infrastructure to promote innovation in the financial services industry.

iii. CBN should consider relaxing its policies on restriction of activities by banks. While it is understandable that there are risks in banks engaging in securities, real estate and insurance, the future of banking lies in fintech, and hence banks should be able to adopt corporate level strategies that encourage integration – be it lateral or allied integration.

iv. In the alternative, banks should consider establishing strategic partnerships with fintech companies to drive financial innovation.

v. This study makes it imperative for states and federal government to create enabling environment and institute policies that will promote financial innovation. Enabling environment includes infrastructure and social amenities, while policies should award recognition and awards to banks driving innovation leading to economic growth. In addition, government regulations

(facilitated by CBN) should relax rules on portfolio investment, and foreign exchange restrictions hindering financial innovation.

**Internal Governance Controls and Corporate Social Performance:**

i. Whilst CBN has introduced the sustainable banking practices, compliance is currently left at banks' discretion. CBN is advised to ensure a mandatory enforcement by all banks. This would provide a level-playing field and allow a critical mass of practitioners.

ii. Furthermore, CBN should make it mandatory for female representation on the board (subject to qualification) to further promote diversity.

iii. It is imperative that the board of directors should ensure that a female director is given first right of refusal to oversee the initiatives and execution of social responsibility practices and activities.

iv. Banks are encouraged to communicate their verifiable social responsibility practices (affecting employees, customers, and the community) on monthly basis in mass media and social media platform.

v. Credible local and international rating agencies are encouraged to take up a yearning gap in provision of independent rating services - on banks' social responsibility practices, reputation, and sustainability among others for investment decisions and advice, institutional money managers and by academics.

vi. Banks are encouraged to partner with non-governmental organisations (NGOs) or develop strategic partnerships with organisations at the forefront of social responsibility.

vii. Managements of banks are advised to adopt a social responsibility strategic plan (rather than a quick-fix, or feel-good exercise), develop metrics to measure the impact of their social responsibility practices, involve everyone, and focus on key advocacy rather than random activities.

viii. CBN, communities, and governments are encouraged to introduce awards and recognition for socially responsible banks.

**Ethical leadership and Corporate Reputation:**

i. An ethics commission/tribunal (involving the collaboration of the private and public sector) backed by statutes, and headed by a judge is required as an ombudsman to receice, review, adjudicate, and sanction erring bank officials involved in unethical practices, up to and

including prosecution. Representation on the commission should include members of the professional bodies (such as ICAN, NBA, NMA, COREN, NUJ), academia, NGOs, EFCC, CBN, and NDIC among others.

ii. NGOs, media, social activists are encouraged to be more vocal and proactive in keeping bank board and management on their toes by identifying corporate reputation issues affecting banks (both positively and negatively).

iii. CEOs as the face of their banks are encouraged to develop initiatives such as stakeholders' matrix map on reputational issues; metrics to measure and a plan to manage the bank's corporate reputation.

iv. Management of banks are encouraged to engage actively in social responsibility practices, network actively, engage credible third parties to speak for the company, foster innovation, and establish trusted partnerships (especially with the IT industry).

v. CBN as regulator is encouraged to adopt reward and sanctions framework for promoting ethical practices.

**Agency Mechanisms and Financial Performance:**

i. CEO's age – CBN is advised to regulate the minimum age for banks' CEO. The law currently provides for a maximum tenure of 10 years, and considering the findings which show a positive association and significance between CEO's age and financial performance, and the descriptive statistics that show the mean age at 45, this study recommends a minimum age of 54 years for bank CEOs (based on the sensitivity analysis performed using a dummy variable on CEO age).

ii. CEO's remuneration – The study's findings show that current remuneration structure is sub-optimal, suggesting that CEOs may be earning compensation largely because of their bank size rather than because they are maximising shareholders' value. It is therefore imperative that Boards of Directors (remuneration and nomination committees) need to redesign the incentive structure such that CEOs' remuneration is within a reasonable threshold. Average annual remuneration of CEOs stands at N66m (over the last 11 years) and N98m (over the last 3 years), while the annual minimum wage is N216,000. CBN is also advised to introduce regulations tying CEOs salary structure to a benchmark. The operating cost of banks is huge, but the most significant contributor is remuneration, which in Nigeria is very disproportional to other industries. In addition, based on data from the selected banks, the inequality between CEO's

remuneration and average bank employee's salary is significant at 11:1. A revised remuneration structure will thus create opportunities for more employment and less pressure on bank CEOs to do unethical things in order to achieve short-term results. In order to promote transparency and accountability, it is imperative to legislate the publication in national dailies and annual reports the pay ratios between CEO and other employees. In addition, it is recommended that an independent audit of executive pay is carried out by CBN on bi-annual basis.

iii. Insider ownership – Our findings show the mean share ownership by board at approximately 7%. CBN currently requires individual share ownership of 5% and above to apply for approval from CBN, but the mean aggregate insider share ownership in selected banks is above the individual threshold (5%) thus constituting a significant shareholding. CBN is advised to regulate share ownership by directors (executive and non-executive). This study prescribes a total maximum insider share ownership limit of 2% (based on the sensitivity analysis performed using a dummy variable on insider ownership) to encourage professionalism and prevent insider abuse.

iv. Board ethnicity – The average board ethnic concentration based on our descriptive statistics worked out as 60% of the total board size. The nomination and governance committees of banks are advised to recognise the influence of cultural affinity and hence be guided by pragmatism (of the local context) rather than tokenism (of imported practices) in composition of the board.

**Corporate Governance Practices Disclosure and Sustainability Reporting**

i. The accounting regulatory body, the Institute of Chartered Accountants of Nigeria (ICAN) is encouraged to develop a statement of accounting standards, and a measurement system that can integrate financial, social, and environmental performance, which can be published in the annual reports in a common and comprehensible format like the financial statements. In addition, the body should organise training and education for accountants, non-accountants, and all users of financial statements.

ii. Management of banks are encouraged to develop sustainability strategic plan, with quantitative and qualitative metrics for measuring the plan and progress achieved. Management Information System must also be developed to provide information for management and the board on sustainability.

iii. It is imperative for CBN to mandate a 100% screening of all credit applications via the ESRM system in banks. In addition, a standardised template should be developed for banks' monthly

reporting. It is also advised that a department should be created in CBN for vetting the reports, whilst bank examiners should carry out on the spot assessments of the banks' sustainability performance.

iv. In order to reduce customer complaints to the barest minimum, it is imperative for CBN to introduce a 'name and shame' program wherein unscrupulous banking activities and unethical practices are published, whilst involved banks are sanctioned.

v. The academia is encouraged to engage in research studies that can track sustainable practices of selected companies over a long period of time – say twenty years or more (using experimental research design) with a view to establishing influence of sustainable practices on long-term survival of organisations.

## 6.4 Contributions to Knowledge

In addition to providing suggestions for further studies,

i. This study extends extant literature on corporate governance, by providing empirical validation that internal governance controls (specifically board and management structure) have implications for deposit money banks to enhance their corporate social performance.

ii. This study established that ethical leadership (in form of organizational ethical programs and ethical culture) influences corporate reputation.

iii. This study added to existing knowledge on the role of regulation, by providing empirical evidence on the different elements of regulation (supervision, capital requirements, and activity restriction) that stimulate financial innovation in deposit money banks.

iv. This study expounded knowledge on the role of agency mechanisms in financial performance, by substantiating that components of agency mechanisms (such as Board ethnicity, CEO Age, CEO remuneration, insider ownership, and to a lesser extent board gender, CEO tenure and total meetings) impact significantly on deposit money banks' financial performance.

v. This study provided empirical evidence to show that bank characteristics (bank size, bank leverage and bank age) have a significant role to play in moderating the influence of agency mechanisms on financial performance.

vi. This study has also brought to the fore the importance of corporate governance practices disclosure in stimulating deposit money banks' sustainability performance reporting.

vii. As mentioned in section 2.3.1 on gaps in literature, few studies have empirically tested the causal analysis between corporate governance and sustainability. No attempt has also been made to develop an integrated instrument to measure sustainability. This study has filled the gap by designing a Sustainability Index to measure the economic, social and environmental performance of deposit money banks in Nigeria. Practitioners would thus be able to measure their level of progress in attaining sustainability. The index is adaptable and applicable to other segments of the financial services sector and industries in the private corporate sector. Similarly, the study developed a Corporate Governance Practices Disclosure Index for the banking sector.

viii. The study provides methodological contribution in corporate governance literature through the measuring instruments (questionnaire, interview questions), mixed-methods design, and thematic analysis from the interview. The study generated fourteen (14) mathematical models – one each for objective 1, 2, 3, and 6, and five each for objective 4 and 5 based on equations specified in the study variables. The predicted outcomes from the various model specifications provide guide for practitioners.

ix. The study extends corporate governance literature by blending the application of the agency and stakeholders' theory in proposing a conceptual model based on the integrated theories. The model below suggests that corporate governance dimensions enhance the deposit money banks' sustainability. The dimensions involve the interdependency of internal mechanisms (internal governance controls and agency mechanisms), external mechanisms (regulation), and stakeholder management (ethical leadership and governance practices disclosure). The interplay leads to sustainability outcomes which manifest in economic sustainability (financial performance and financial innovation), and social and environmental sustainability (corporate social performance and corporate reputation). These outcomes give full representation of the effect of corporate governance. It would therefore guide bank managements, regulators,

investors, and other stakeholders in adopting the best strategies for promoting corporate governance to enhance sustainability.

**Corporate Governance -Sustainability Model**

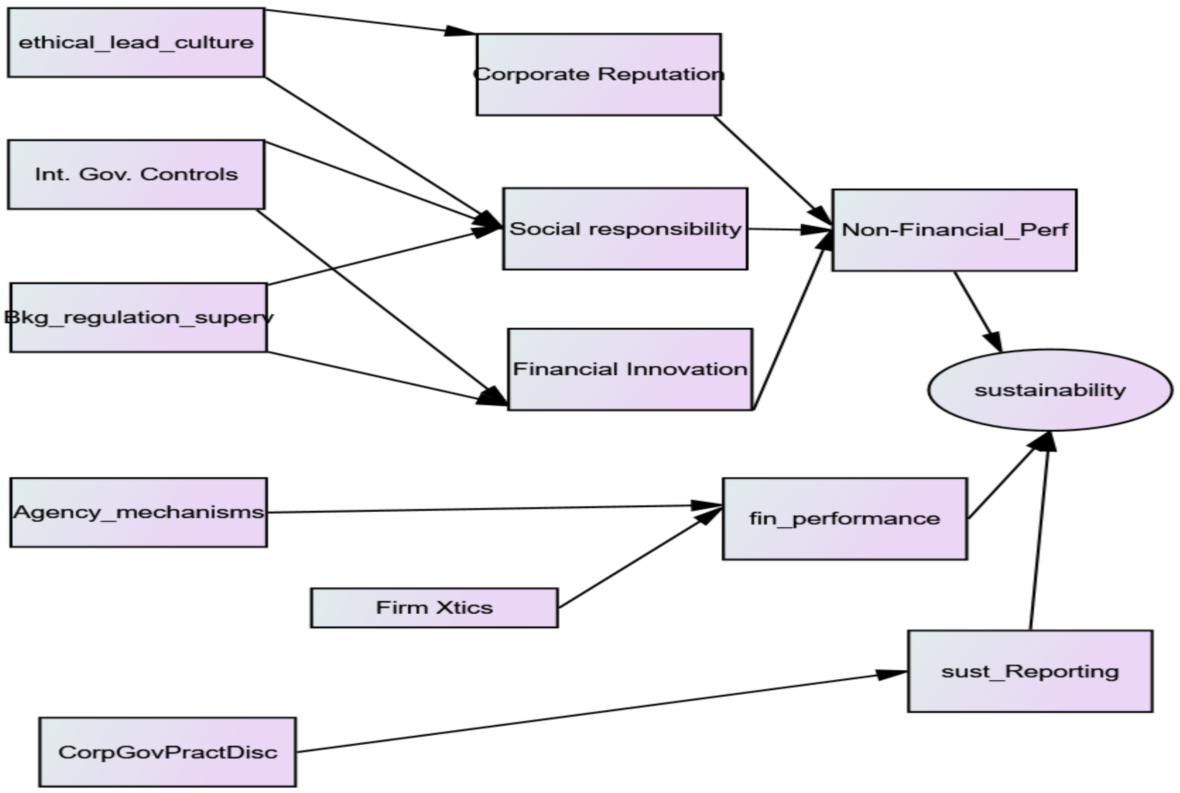


**Fig. 6.1 Corporate Governance - Sustainability Model**

**Source: Author's Model (2017)**

## 6.5 Limitations of the study

This study was limited in the following areas:

i. Eight (8) publicly listed banks were adopted for the study without being able to include private banks due to their financial statements not being made public. By implication, the results of this study may not be generalised to privately-owned banks, as well as those that failed to meet the selection criteria for the selected sample.

ii. The study sought access to observe board meeting and or review minutes of board meeting but access was denied.

iii. Five hundred and seventy-three employees from Lagos branches and Head-Office constituted the study's respondents. Their views may not be generalised to the banks' branches outside Nigeria.

iv. Some of the study variables such as financial innovation and corporate reputation were collected in one single moment in time. Carrying out a study over a longer period (through longitudinal designs) may yield a different result.

v. In evaluating the selected banks' sustainability performance reporting, specifically on corporate social performance (social and environmental performance), some of the banks had stand-alone sustainability performance records (online brochures, website contents etc.) in addition to the disclosure in the annual reports. The approach afforded such banks the opportunity to express their activities in more details, compared to banks that restricted their disclosure to the annual report template format.

## 6.6 Suggestions for Further Studies

i. This study has examined the influence of agency mechanisms on financial performance using secondary data extracted from the banks' annual reports. Other studies may want to consider the use of other methodologies such as a matched-pair sample (of publicly listed and private banks, or banks meeting the criteria in this study versus those not meeting the criteria), a fuzzy multi-criteria decision-making technique (such as Fuzzy Analytic Hierarchy Process, or Fuzzy Technique for Order Preference), or a case study. A suitable opportunity would be: '*Agency Mechanisms and Financial Performance – A fuzzy Multi-Criteria Decision-Making Approach'*.

ii. The study on internal governance controls and corporate social performance was carried out using the questionnaire and interview. This provides information collected in one single

moment in time, rather than a study of opinions over a fairly long time (i.e. longitudinal design). The use of a different theoretical framework involving social theory, contingency theory, or system theory may also be considered. Suggested research areas may therefore include: '*Determinants of motivation for social responsibility as a mediator for financial performance*', and '*Evaluative Study of Corporate Governance in driving Environmental performance– A juxtaposition of Mechanisms*'.

iii. Whilst corporate reputation, and financial innovation (as indicators of sustainability) were studied from the employees' perspective, and within a point in time, further studies may consider external stakeholders' perspectives (such as customers, community, regulators, and rating agencies), and a longitudinal study. Potential line of research may include: '*The environmental factors influencing financial innovation*', and '*Ethical Leadership and Corporate Reputation – A Stakeholders' Perspective*'.

iv. This study considered bank characteristics as moderator of the influence of agency mechanisms on financial performance, further studies can consider macro-economic variables such as GDP, inflation, interest rates, foreign exchange rates, and level of competition. This is very critical for a developing country such as Nigeria, where the fortunes or misfortune of a firm may be strongly tied to such macro-economic variables. Future research may look at: '*Macro-economic Indices as moderator of corporate governance and financial performance – A Study of Selected Banks'*. Other opportunities for research may include: '*Evaluative Study of Diversity in Firm Performance – An Emerging Economy Perspective'*, and '*Corporate Governance and Performance – The moderating and mediating role of Culture and Corporate Strategy'*.

v. The sustainability performance index developed in this study may be used by other researchers to practically evaluate sustainability of banks or financial services firms, under a potential study '*Corporate Governance and Sustainability in Nigerian Banking – The Journey So far*'

vi. Other aspects of this study which may offer avenue for further studies include the following: This study used an unbalanced panel data for objective 4 and 5; limited its study scope to Lagos branches and publicly listed deposit money banks among others. Future research may consider expanding the number of study observations from eighty-eight (88) in this study, sample size to include respondents in other branches within and outside Nigeria, and privately-owned banks.

## 7.0 References


Abor, J. and Biekpe, N. (2007) Corporate Governance, ownership structure and performance of SMEs in Ghana: implications for financing opportunities, *Corporate Governance* **l7**: 288–300.

Acharya, V.V., Carpenter, J., Gabaix, X., John, K., Richardson, M., Subrahmanyam, M., Sundaram, R. and Zemel, E. (2009). Corporate governance in the modern financial sector. In V.V. Acharya and M. Richardson (eds.), Restoring Financial Stability. Hoboken, NJ: Wiley and Sons.

Adams and Maher (2003). Is corporate governance different for bank holding companies? Federal Reserve Bank of New York *Economic Policy Review*, **9(1):** 123-142.

Adams, R.B. and Ferreira, D. (2007) A theory of friendly boards. *Journal of Finance* **62(1):** 217–250.

Adams, R. and Ferreira, D. (2009). Women in the boardroom and their impact on governance and performance. Journal of Financial Economics, 94, 291-309.

Adams, R.B. and Mehran, H. (2012) Bank board structure and performance: evidence for large bank holding companies. *Journal of Financial Intermediation* **21**: 243–267.

Adams, R.B., Hermalin, B.E. and Weisbach, M.S. (2010). The Role of Boards of Directors in Corporate Governance: A Conceptual Framework and Survey. *Journal of Economic Literature* **48(1):**58–107

Adegbite, E. and Amaeshi, K. (2010). Multiple Influences on Corporate Governance in sub-Saharan Africa: Actors, Strategies and Implications. *CSGR Working Paper* 267/10

Adegbite, E. and Nakajima, C. (2011). Corporate governance and responsibility in Nigeria. International *Journal of Disclosure and Governance*. **8:** 252 – 271.

Adegbite, E. (2012). "Corporate governance regulation in Nigeria", Corporate Governance: *The International Journal of Business in Society*, **12(2):** 257 – 276

Adelopo, .I. (2011). Voluntary disclosure practices amongst listed companies in Nigeria. Advances in Accounting, incorporating Advances in International Accounting **27** (2011): 338–345

Adeoye, A. and Amupitan, M.D. (2015). Corporate governance in the Nigerian banking sector: issues and challenges. *European Journal of Accounting Auditing and Finance Research*, **3 (5):** 64-89.

Adeyemi, K.S. (2005). "Banking Sector Consolidation in Nigeria: Issues and Challenges". *Union Digest.* **9(3 and 4).**

Aduda, J (2011). The relationship between executive compensation and firm performance in the Kenyan banking sector. *Journal of Accounting and Taxation* **3(6):** 130-139.

Aebi, V., Sabato, G. and Schmid, M. (2012) Risk management, corporate governance, and bank performance in the financial crisis. *Journal of Banking and Finance* **36(12)**: 3213–3226.

Agbonifoh B.A and Yomere G.O, (1999). Research Methodology: in the Management and Social Sciences, Benin City, Uniben Press.

Aggarwal, P. (2013). Sustainability reporting and its impact on corporate financial performance: A literature review. Indian Journal of Commerce and Management Studies. **4(3):** 51-59.

Agle, B. R., Mitchell, R. K., and Sonnenfeld, J. A. (1999). Who matter to CEOs? An investigation of stakeholder attributes and salience corporate performance, and CEO values. *Academy of Management Journal*, **42(5):** 507-525.

Aguilera, Ruth V. and Jackson, Gregory (2003) 'The Cross-National Diversity of Corporate Governance: Dimensions and Determinants', *Academy of Management Review* **28(3)**: 447-465.

Agusto (2006). Challenges and opportunities in the consolidating Nigerian banking sector. Retrieved from https://www.cbn.gov.ng/OUT/PUBLICATIONS/BSD/2005/AGUSTO.PDF

Ahmad, B.A. and Mansur, L.K. (2012). Corporate governance and financial performance of banks in the post-consolidation era in Nigeria. *International Journal of Social Sciences and Humanity Studies*. **4(2)**: 27-36

Ahn, S., Jiraporn, P., and Kim, Y. S. (2010). Multiple directorships and acquirer returns. *Journal of Banking and Finance*, **34(9):** 2011–2026.

Ahunwan, B. (2002). Corporate governance in Nigeria. *Journal of Business Ethics*, **37(3):** 269-287.

Aina, K (2016). Why businesses don't outlive founders. Retrieved from: http://theinterview.com.ng/2016-07/why-nigerian-businesses-dont-outlive-founders-kola-aina

Ajibo, K.I. (2015) "Risk-based regulation: the future of Nigerian banking industry", *International Journal of Law and Management* **57 (3):** 201-216, https://doi.org/10.1108/IJLMA-02-2014-0014

Akers, J. (1989). ``Ethics and competitiveness putting first things first". *Sloan Management Review*, **30(2):** 69-71.

Akpan, E.O., and Amran, N.A. (2014). Board characteristics and company performance: Evidence from Nigeria. *Journal of Finance and Accounting*. **2(3):** 81-89

Akpan, E.S., and Riman, H.B. (2012). Does corporate governance affect bank profitability? Evidence from Nigeria. *American International Journal of Contemporary Research*. **2(7):** 135-145

Aktaruddin, M.; Hossain, M.A.; Yao, L. (2009). Corporate Governance and Voluntary Disclosure in Corporate Annual Reports of Malaysian Listed Firms. *J. Appl. Manag. Account. Res.* **7:** 1–20

Al-Baidhani, A.M. (2014). Review of Corporate Governance Bundle. Retrieved from: http://ssrn.com/abstract=2462863

Al- Faki, M (2006). Transparency and corporate governance for capital market development in Africa: The Nigerian case study, Securities Market Journal, :9- 28.

Alam, N. (2012). The Impact of Regulatory and Supervisory Structures on Bank Risk and Efficiency: Evidence from Dual Banking System. *Asian Journal of Finance and Accounting*, **4 (1):** 216-244.

Al-Amarneh, A. (2014). Corporate Governance, Ownership Structure and Bank Performance in Jordan. *International Journal of Economics and Finance* **6 (6)**

Albassam, W.M. (2014). Corporate Governance, Voluntary Disclosure and Financial Performance: An Empirical Analysis of Saudi Listed Firms Using a Mixed-Methods Research Design. *Thesis,* University of Glasgow.

Ali, A. (2013). How Board Structure Influences the Corporate Social Responsibility Strategy of the Firm? Pakistan's Perspective. *Global Journal of Management and Business Research Administration and Management* **13 (11)**

Ali, J.I, Ekpe, M.J., and Aigba, M.O. (2016). Banking Sector Reforms and Bank Performance in Sub-Saharan Africa: Empirical Evidence from Nigeria. IOSR Journal of Business and Management **18 (5): 36-47**

Allen, F. and Gale, D. (2000) Comparing Financial Systems. Cambridge, MA: MIT Press.

Allet, M. (2011). Measuring the environmental performance of microfinance. Solvay Brussels School Economics and Management.Centre Emile Bernheim. *Research Institute in Management Sciences*: 1-27

Al-Tuwaijri, S.A., Christensen, T.E., and Hughes II, K.H. (2004). "The relations among environmental disclosure, environmental performance, and economic performance: A simultaneous equations approach", *Accounting, Organisations and Society*, **29 (25):** 447-471.

Amaeshi, K. (2017). Nigerian Financial Regulators and the Prospects of Sustainable Finance. Retrieved from http://opinion.premiumtimesng.com/2017/04/30/nigerian-financial-regulators-and-the-prospects-of-sustainable-finance-by-kenneth-amaeshi/

Amaeshi, K, Adi, B, Ogbechie, C and Amao, O. (2006) "Corporate Social Responsibility in Nigeria: Western Mimicry or Indigenous Influences?". No. 39-2006, ICCSR Research Paper Series – ISSN 1479 – 5124, The University of Nottingham, :4,17, 25.

Ameer, R., and Othman, R. (2012). Sustainability Practices and Corporate Financial Performance: A Study Based on the Top Global Corporations. *Journal of Business Ethics* **108**:61–79 DOI 10.1007/s10551-011-1063

Anderson, J. C., and Gerbing, D. W. (1998). Structural equation modeling in practice: a review and recommended two-step approach. *Psychological Bulletin*, **103(3):** 411–423

Angelides, P., and Thomas, B. (2011). The financial crisis inquiry report: Final report of the National Commission on the Causes of the Financial and Economic Crisis in the United States. *Choice Reviews Online* **48:** 1-663. doi:10.5860/choice.48-7034

Anjum, Z. (2010). Determinants of CEO Compensation in Pakistan from the year 2007-2009

Aragon-Correa, J. A. (1998). Strategic proactivity and firm approach to the natural environment. *Academy of Management Journal* **41**: 556–567.

Arani, M.H.Z (2016). The Effect of Corporate Governance Mechanisms on Social Responsibility Disclosure. *Mediterranean Journal of Social Sciences* **7(4)**

Aras, G., and Crowther, D. (2008). Governance and sustainability. *Management Decision*, **46(3):** 433-448. http://dx.doi.org/10.1108/00251740810863870

Arjoon, S. (2005), "Corporate governance: An ethical perspective", *Journal of Business Ethics*, **61(4):** 343-352.

Arnaboldi, F. and Rossignoli, B. (2015). "Financial Innovation in banking", *Department of Law Working paper*, University of Milan

Arora, P, and Petrova, M.T. (2009). Corporate Social Performance, Stakeholder Coalitions, Corporate Governance and Performance. Available at SSRN: https://ssrn.com/abstract=1364993. Accessed on 12 December, 2016.

Asher, C.C., Mahoney, J.M. and Mahoney, J.T. (2005). Towards a Property Rights Foundation for a Stakeholder Theory of the Firm. *Journal of Management and Governance* **9**:5–32

Babalola, A., and Adedipe, O.A. (2014). Corporate Governance and Sustainable Banking Sector: Evidence from Nigeria. *Research Journal of Finance and Accounting* **5(12)**:32-43

Backstrom, S-L, and Karlsson, J. (2015). Corporate Sustainability and Financial Performance - The influence of board diversity in a Swedish context. *Master's Thesis* Department of Business Studies Uppsala University Spring Semester of 2015

Bagozzi, R. P., and Yi, Y. (1988). On the evaluation of structural equation models. *Journal of the Academy of Marketing Science*, **16(1):** 74–94.

Baicu, C.G. (n.d). The impact of financial innovation on banking regulation. Evidence from the global financial crisis. :31-40.

Bainbridge S.M. (2002). Why a board? Group decision-making in corporate governance. *Vanderbilt Law Review*. **55(1)**: 1-55.

Baker, H.K., and Anderson, R. (2010). Corporate governance: A synthesis of theory, research and practice. The Robert W. Kolb Series in Finance, John Wiley and Sons, Inc.

Baker, G. P., and Kennedy, R. E. (2002). Survivorship and the Economic Grim Reaper. *The Journal of Law, Economics, and Organisation,* **18 (2):** 324-361.

Balkin, D. B., Markman, G., and Gomez-Mejia, L. R. (2000). Is CEO pay in high-technology firms related to innovation? *Academy of Management Journal* **43:** 1118–1130.

Baltagi, B.H. (2005). Econometric analysis of panel data. Third Edition. John Wiley and Sons Ltd, The Atrium, Southern Gate, Chichester, West Sussex PO19 8SQ, England

Banks for International Settlements (BIS), (2004). Bank failures in Mature Economies. Working Paper No. 13. Retrieved from: http://www.bis.org/publ/bcbs_wp13.pdf Accessed 5 January, 2017.

Banks for International Settlements (BIS), (2016). History of Basel Committee. Retrieved from: http://www.bis.org/bcbs/history.htm Accessed 5 January, 2017.

Bansal, P., and Roth, K. (2000). Why companies go green: A model of ecological responsiveness. *Academy of Management Journal,* **43**: 717–736.

Bansal, P. and DesJardine, M. R. (2014). "Business sustainability: It is about time", *Strategic Organization*, **12** (1): 70-78.

Bartlett, J.E., Kotrlik, J.W., and Higgins, C.C. (2001). Organizational research: Determining appropriate sample size in survey research. Information Technology, Learning, and Performance Journal, 19(1).

Barnea, A. and Rubin, A. (2010). 'Corporate Social Responsibility as a Conflict between Shareholders', *Journal of Business Ethics* **97:** 71–86.

Barnett, Jermier and Lafferty (2006). Corporate Reputation: The Definitional Landscape. Corporate Reputation Review **9(1):** 26–38 © 2006 Palgrave Macmillan Ltd. 1363–3589

Barney, J., and Clark, D. N. (2007). Resource-based theory. New York: Oxford.

Barth, J.R., Caprio, G. and Levine, R.  (2003). Bank Regulation and Supervision: Lessons from a New Database, In Garza J.A.M. (Ed), Macroeconomic Stability, Financial Markets, and Economic Development, Mexico City: Banco de Mexico.

Barth, J.R., Caprio, G. and Levine, R. (2004), "Bank Regulation and Supervision: What Works Best?" *Journal of Financial Intermediation* **13:** 205-248.

Bartholdy, J., Boyle, G.W. and Stover, R.D. (2003). "Deposit Insurance and the Risk Premium in Bank Deposit Rates", *Journal of Banking and Finance* **27(4):** 699-717

Basel (2004). Bank failures in Mature Economies. *Working Paper* No. **13**:1-69

Basel (2015). Basel Committee on Banking Supervision Guidelines. Corporate governance principles for banks. Retrieved from: http://www.bis.org/bcbs/publ/d328.pdf. Accessed on 5 January, 2017.

Battaggion, M.R and Tajoli, L (2000). Ownership structure, innovation process and competitive performance: the case of Italy. Being paper presented at the "Corporate Governance and Investment Project Workshop, Aarhus, 13-14 November, 1999

Baumgartner, R.J., and Ebner, D. (2010). Corporate sustainability strategies: sustainability profiles and maturity levels. *Sustainable Development* **18(2)**: 76–89. DOI: 10.1002/sd.447

Baysinger, B., and Hoskisson, R. E. (1990). The composition of boards of directors and strategic control: Effects on corporate strategy. *Academy of Management Review* **15(1):** 72–87.

Bebchuk, L. and Grinstein, Y. (2005). The growth of executive pay. The Social Science Research Network Electronic Paper Collection: http://papers.ssrn.com/abstract_id=648682"

Bebchuk, L.A. and Spamann, H. (2010) Regulating bankers' pay. *Georgetown Law Journal* **98(2)**: 247–287.

Bebchuk, L.A. and Weisbach, M.S.  (2010). The state of corporate governance research. *The Review of Financial Studies* **23(3)**: 939–961.

Bebchuk, L.A., Cohen, A. and Spamann, Hr. (2010) The wages of failure: executive compensation at Bear Stearns and Lehman 2000–2008. *Harvard Law School Working Paper* 657.

Becht, M., Bolton, P., and Roell, A. (2003). Corporate governance and control. In G. M. Constantinides, M. Harris and R. M. Stulz (Eds.), *Handbook of the Economics of Finance* **1:** 1-109: Elsevier.

Beck, T., Chen, T., Lin, C. and Song, F. (2012) Financial Innovation: The Bright and the Dark Sides, *Working Papers* 052012, Hong Kong Institute for Monetary Research.

Beck, T., Demirguc-Kunt, A. and Levine, R. (2006), "Bank Supervision and Corruption in Lending", *Journal of Monetary Economics* **53:** 2131-2163.

Becker, G., (1983), "A Theory of Competition among Pressure Groups for Political Influence", *Quarterly Journal of Economics* **98:** 371-400.

Behr, P., Schmidt, R.H., and Xie, R. (2010). "Market Structure, Capital Regulation and Bank Risk Taking", *Journal of Financial Services Research* **37 (2):** 131-158

Bekhet, A. K., and Zauszniewski, J. A. (2012). Methodological triangulation: An approach to understanding data. *Nurse Researcher* **20(2):** 40-43. doi:10.7748/nr2012.11.20.2.40.c9442

Bektas, E. and Kaymak, T. (2009). Governance Mechanisms and Ownership in an Emerging Market: The Case of Turkish Banks. *Emerging Markets Finance and Trade* **45(6):** 20–32.

Beltratti, A. and Stulz, R.M. (2012) The credit crisis around the globe: why did some banks perform better during the credit crisis? *Journal of Financial Economics* **105(1)**: 1–17.

Bentler, P.M., and Bonett, D.G. (1980). Significance tests and goodness of fit in the analysis of covariance structures. *Psychological Bulletin* **88:** 588-606.

Bentler, P.M., and Wu, E.J.C. (2002). EQS 6 for Windows user's guide. Encino, CA: Multivariate Software.

Berens, G., and van Riel, C. B. M. 2004. Corporate associations in the academic literature: Three main streams of thought in the reputation measurement literature. Corporate Reputation Review, **7**: 161–178.

Berle, A A and Means, G C (1932): The Modern Corporation and Private Property, Macmillan, New York.

Berman, S.L., Wicks, A.C., Kotha, S., and Jones, T.M. (1999). Does stakeholder orientation matter? The relationship between stakeholder management models and firm financial performance. *Academy of Management Journal* **42(5)**: 488–506.

Bernardi, R.A. and Threadgill, V. H. (2010). Women Directors and Corporate Social Responsibility. *EJBO*, **15 (2):** 15-21.

Bernasek, A., and Shwiff, S. (2001). Gender, Risk, and Retirement June 2001. *Journal of Economic Issues* **35(2)** DOI10.1080/00213624.2001.11506368

Berrone, P. and Gomez-Mejia, L.R. (2009). Environmental performance and executive compensation: An integrated agency-institutional perspective. *Academy of Management Journal*, **52 (1):** 103–126

Bertrand, M., and Mullainathan, S. (2003). Enjoying the quiet life? Corporate governance and managerial preferences. *Journal of Political Economy* **111 (5):** 1043-1075.

Bertrand, M., and Schoar, A. (2003). Managing with style: The effect of managers on firm policies. *Quarterly Journal of Economics*, **118(4):** 1169–1208.

Bhagat, S., and Bolton, B. (2008). Corporate governance and firm performance. *Journal of Corporate Finance*, **14(3):** 257–273.

Bhasa, M.P. (2004) Understanding the corporate governance quadrilateral, *Corporate Governance*, 4:7.

Bhasin, M.L. (2010). Corporate Governance Disclosure Practices: The Portrait of a Developing Country. International Journal of Business and Management, **5(4):** 150-167

Black, B., Jang, H. and Kim, W. (2003). Does Corporate Governance Affect Firm Value? *Working paper* 327, Stanford Law School.

Blair, M.M. (1995). Ownership and Control: Rethinking Corporate Governance for the Twenty-First Century. Washington, D.C.: Brookings Institution.

Boadu, M. (2013). Ethical dimensions of corporate governance practice in Ghana: Building a theoretical perspective. A thesis submitted to the Plymouth University in partial fulfilment for the degree of doctor of philosophy.

BOFIA (2002). Banks and Other Financial Institutions Act (Amended). Retrieved from: https://www.cbn.gov.ng/OUT/PUBLICATIONS/BSD/1991/BOFIA.PDF. Accessed on 4 October, 2016.

Boone, A.L., Field, L.C., Karpoff, J.M. and Raheja, C.G. (2007), ‘‘The determinants of corporate board size and composition: an empirical analysis’’, *Journal of Financial Economics*, **85(1):** 66-101.

Bradford, M, Earp, J.B., Showalter, D.S., and Williams, P.F. (2017). Corporate Sustainability Reporting and Stakeholder Concerns: Is There a Disconnect? *Accounting Horizons*, *American Accounting Association* **31(1):** 83-102

Brammer, S. and Pavelin, S. (2006). Corporate reputation and social performance: the importance of fit, *Journal of Management Studies*, **43(3)**: 436-56

Brennan, N., and Solomon, J. (2008). Corporate governance, accountability and mechanisms of accountability: An overview. Accounting, Auditing and Accountability Journal, **21:** 885-906.

Brenner, S. N., and Cochran. P. (1991). The stakeholder theory of the firm: Implications for business and society theory and research. Paper presented at the annual meeting of the International Association for Business and Society. Sundance, UT.

Brown, L. D. and M. L. Caylor, M.L. (2006). “Corporate Governance and Firm Valuation”. *Journal of Accounting and Public Policy* **25:** 409 – 434.

Brown, M. E., Trevin ˜o, L. K., and Harrison, D. A. (2005). Ethical leadership: A social learning perspective for construct development and testing. *Organisational Behavior and Human Decision Processes*, **97:** 117–134.

Brown, M., Whysall, P. (2010). Performance, reputation, and social responsibility in the UK’s financial services: a post-credit crunch interpretation. *Service Industries Journal* **30(12):** 1991–2006.

Brown, W., Helland, E., and Smith, K. (2006). Corporate philanthropic practices. *Journal of Corporate Finance* **12**: 855-877.

Browne, M.W. and Cudeck, R. (1993). Alternative ways of assessing model fit. In Bollen, K.A. and Long, J.S. [Eds.] Testing structural equation models. Newbury Park, CA: Sage, 136–162.

Brunklaus, B., Malmqvist, T. and Baumann, H. (2009) ‘Managing stakeholders or the environment? The challenge of relating indicators in practice’. *Corporate Social Responsibility and Environmental Management*, **16**: 27-37

Bryman, A. (1988) Quantity and Quality in Social Research, London, Routledge

Bryman, A. (2012). Social Research Methods, 4th Edition, Oxford Press, New York, US.

Byrnes, J. P., Miller, D. C., and Schafer, W. D. (1999). Gender differences in risk taking: A meta-analysis. *Psychological Bulletin*, **125(3):** 367–383.

Bukhari, M.S.D. (2014). The Impact of Institutions on the Development of Corporate Governance in Saudi Arabia. Thesis Submitted to the University of Nottingham for the Degree of Doctor of Philosophy

Burson-Marsteller and Research International (2003). CEO reputation study –Belgium 2003. Retrieved from https://issuu.com/burson-marsteller-emea/docs/ceoreport

Cadbury, A. (2003). Corporate Governance: A Framework for Implementation. Overview Foreword by Sir Adrian Cadbury. Retrieved from: http://documents.worldbank.org/curated/en/831651468781818619/pdf/30446.pdf

Cadbury Committee Report (1992). The Report of the Committee on the Financial Aspects of Corporate Governance, Gee Publishing, London.

Cai, J., Liu, Y., Qian, Y., and Yu, M. (2015). Information asymmetry and corporate governance. *The Quarterly Journal of Finance,* **5(3).**

Calton, J. and Payne, S. (2003). 'Coping with Paradox', *Business and Society* **42:** 7–42.

CAMA (1990). Companies and Allied Matters Act.

Campbell, K., and Minguez-Vera, A. (2008). Gender diversity in the boardroom and firm financial performance. *Journal of Business Ethics*, **83(3):** 435–451.

Campbell, K., and Minguez-Vera, A. (2010). Female board appointments and firm valuation: Short and long-term effects. *Journal of Management and Governance*, **14(1):** 37–59.

Caprio, G., Laeven, L., and Levine, R. (2007). Governance and bank valuations, *Journal of Financial Intermediation*, **16**:584–617.

Carney, M. (2005). Corporate governance and competitive advantage in family-controlled firms. *Entrepreneurship, Theory, and Practice,* **29(3):** 249-265.

Carter D.A., Simkins B.J., and Simpson W.G. (2010), "The Gender and Ethnic Diversity of US Boards and Board Committees and Firm Financial Performance", *Corporate Governance: An International Review*, **18(5):** 396-414.

Carter, D., Simkins, B., and Simpson, W. (2003). Corporate governance, board diversity, and firm value. *Financial Review*, **38(1):** 33–53.

Carton, R. B., and Hofer, C. W. (2006). Measuring organisational performance: metrics for entrepreneurship and strategic management research. Cheltenham, UK; Northampton, MA: Edward Elgar.

Casson, .J. (2013). A review of the ethical aspects of corporate governance regulation and guidance in the EU. Occasional paper 8, Institute of Business Ethics. :1-44.

CBN (2006). Code of corporate governance for banks in Nigeria post-Consolidation: 1-20

CBN (2008) Annual report of central bank of Nigeria 2008.

CBN (2012). Nigerian Sustainable Banking Principles

CBN (2013). Financial Inclusion In Nigeria: Issues And Challenges. :1-45

CBN (2014). Revised code of Corporate Governance for Banks and Discount Houses in Nigeria. Retrieved from: https://www.cbn.gov.ng/.../2014/.../circular%20on%20code%20of%20circular%20on... (Accessed 15 October, 2016).

CBN (2015). Financial Stability Report 2015. :1-78

CBN (2016a). List of financial institutions –Deposit money banks. Retrieved from: https://www.cbn.gov.ng/Supervision/Inst-DM.asp

CBN (2016b). Financial Stability Report 2016. :1-71

Central Banks Guide (2015). Banking Industry Regulation. Retrieved from: http://www.centralbanksguide.com/banking+industry+regulation/

Cespa, G. and Cestone, G. (2007). 'Corporate Social Responsibility and Managerial Entrenchment', *Journal of Economics and Management Strategy* **16:** 741–771.

Chardine-Baumann, E., and Botta-Genoulaz, V. (2014). A framework for sustainable performance assessment of supply chain management practices. *Computers and Industrial Engineering*, **76:** 138-147. http://dx.doi.org/10.1016/j.cie.2014.07.029

Charkham, J.P. (1994). Keeping good Company. A Study of Corporate Governance in Five Countries. Oxford University Press

Cheffins, B.R. (1997). "Corporate Governance in the United Kingdom: Lessons for: Canada." *Canadian Business Law Journal*, **28:** 69-106.

Cheffins, B.R. (2012). The history of corporate governance. Working Paper No. 184/2012

Chen, C., Steiner, T. and Whyte, A.M. (1998) Risk-taking behavior and management ownership in depository institutions. *Journal of Financial Research* **21**: 1–16.

Chenhall, R. H., and Langfield-Smith, K. (2007). Multiple perspectives of performance measures. *European Management Journal*, **25:** 266−282.

Cherotich, K.M., Sang, W., Shisia, A.and Mutung'u, C. (2015). Financial innovations and performance of commercial banks in Kenya. *International Journal of Economics, Commerce and Management United Kingdom* **3(5):** 1242-1265

Cho, H., and Pucik, V. (2005). Relationship between innovativeness, quality, growth, profitability, and market value. *Strategic Management Journal*, **26(6)**: 555-575.

Chortareas, G.E., Girardone, C. and Ventouri, A. (2010). Bank Supervision, Regulation and Efficiency: Evidence from the European Union

Chu, K.H. (2011). "Deposit Insurance and Banking Stability", *Cato Journal*, **31 (1):** 99-117.

Chun, J.S., Shin, Y, Choi, J.N, and Kim, M.S. (2011). How Does Corporate Ethics Contribute to Firm Financial Performance? The Mediating Role of Collective Organisational Commitment and Organisational Citizenship Behavior. **39 (4):** 853-877

Chun, R (2005). Corporate reputation: Meaning and Measurement. *International Journal of Management Reviews* **7(2):** 91–109

Claessens, S. (2006). Corporate Governance and Development, World Bank Research Observer.

Claessens, S., and Fan, J.P.H. (2003) Corporate Governance in Asia: A survey. *International Review of finance* **3 (2)**: 71-103

Clarkson, M. B. E. (1995). A stakeholder framework for analyzing and evaluating corporate social performance. *The Academy of Management Review*, **20(1)**: 92-117.

Clarkson. M. B. E. (1991). Defining, evaluating, and managing corporate social performance: A stakeholder management model. In J. E. Post (Ed.), A research in corporate social performance and policy: 331-358. Greenwich, CT: JAI Press.

Clemens, B., and Bakstran, L. (2010). A framework of theoretical lenses and strategic purposes to describe relationships among firm environmental strategy, financial performance, and environmental performance. Management Research Review **33 (4):** 393–405.

Coase, R. H. (1937). The nature of the firm. *Economica,* **4(16):** 386-405.

Cohen, J. (1998). Statistical power analysis for the behavioral sciences. Lawrence Erlbaum Associates, 2nd ed. World Business Council for Sustainable Development. (1992) Eco-efficiency and Cleaner Production: Charting the Course to Sustainability The World Business Council for Sustainable Development United Nations Development Programme Retrieved from http://www.iisd.ca/consume/unep.html http://www.wbcsd.org/web/publications/eco_efficiency_creating_more_value.pdf

Cohen, L., Manion, L. and Morison, K. (2000). Research Methods in Education. London: Routledge Falmer.

Cole, M., Elliotta, R. and Stroblb, E. (2008) 'The environmental performance of firms: The role of foreign ownership, training, and experience'. *Ecological Economics* **65:** 538-546

Coleman, K. A. (2008). Corporate Governance and Firm Performance in Africa: a Dynamic Panel Data Analysis. *Journal for studies in economics and econometrics,* **32 (2):** 1-24

Coles, J., Daniel, N. and Naveen, L. (2004). "Boards: Does One Size Fit All?" Working Paper, Arizona State University.

Combs, J. G., Crook, T. R., and Shook, C. L. (2005). The dimension of organisational performance and its implications for strategic management research. In D. J. Ketchen and D. D. Bergh (Eds.), Research methodology in strategy and management: 259-286. San Diego: Elsevier.

Cooper, D.R, Schindler, P.S and Sun, J. (2006). Business research methods, 9th edn, McGraw-Hill Irwin.

Cooper, M.J., Gulen, H., and Rau, P.R. (2013). Performance for Pay? The Relation Between CEO Incentive Compensation and Future Stock Price Performance. January 2013 DOI10.2139/ssrn.1572085

Cornelissen, J. (2004). Corporate Communications, Theory and Practice. London, U.K.: SAGE Publications

Cornett, M.M., Guo, L.,Khaksari, S. and Tehranian, H. (2010a) The impact of state ownership on performance differences in privately-owned versus state owned banks: an international comparison. *Journal of Financial Intermediation* **19(1):** 74–94.

Cornett, M.M., McNutt, J.J. and Tehranian, H. (2010b) The financial crisis, internal corporate governance, and the performance of publicly-traded U.S. bank holding companies. https://www2.bc.edu/˜tehranih/Hassan%20published%20paper/Crisis%2001-19-10.pdf. (Accessed on October 15, 2016).

Cox, P.; Brammer, S.; and Millington, A. (2004). An Empirical Examination of Institutional Investor Preferences for Corporate Social Performance. *J. Bus. Ethics,* **52:** 27–42

Crane, A., Matten, D., and Moon J. (2008). Ecological citizenship and the corporation – politicizing the new corporate environmentalism. *Organisation and Environment* **21(4)**: 371–389.

Dahya, J., Dimitrov, O., and McConnell, J. (2008). Dominant shareholders, corporate boards, and corporate value: A cross-country analysis. *Journal of Financial Economics*, **87 (1):** 73 100.

Dallas, L. (2002). The new managerialism and diversity on corporate boards of directors. *Tulane Law Review* **76(5–6):** 1363–1405.

Dalton, D.R., Hitt, M.A., Certo, S.T., and Dalton, C.M. (2007). The fundamental agency problem and its mitigation. *The Academy of Management Annals*, **1**: 1 –64.

Daub, C.H. (2007), Assessing the quality of sustainability reporting: an alternative methodological approach, *Journal of Cleaner Production*, **15(1):** 75-85.

Davidson, W. N., III., Xie, B., Xu, W., and Ning, Y. (2007). The influence of executive age, career horizon and incentives on pre-turnover earnings management. *Journal of Management and Governance*, **11(1):** 45–60.

Dawes, J. (1999). The relationship between subjective and objective company performance measures in market orientation research: further empirical evidence. *Marketing Bulletin*, **10(3):** 65-75.

De Hoogh, A.H.B., and Den Hartog, D.N. (2008). Ethical and despotic leadership, relationships with leader's social responsibility, top management team effectiveness and subordinates' optimism: A multi-method study. *ScienceDirect Elsevier, The Leadership Quarterly* **19 (3):** 297-311

Deloitte (2017). Sustainable Banking as a driver for Growth. A survey of Nigerian Banks: 1-39.

De Vaus, D.A. (2002). Surveys in social research, Allen and Unwin, 5th edn, St. Leonards, N.S.W.

Deephouse, D.L. (2000). Media reputation as a strategic resource: an integration of mass communication and resource-based theories *Journal of Management*, **26(6):** 1091–1112.

Demsetz, H. and Lehn, K. (1985) The structure of corporate ownership: causes and consequences, *Journal of Political Economy*, **93:** 1155–77.

Demsetz, H. (1983) The structure of corporate ownership and the theory of the firm, *Journal of Law and Economics*, **26:** 375–390.

Denscombe, M. (2007) The Good Research Guide (3rd edn). Buckingham: Open University Press.

Denzin, NK and Lincoln, Y.S. (2004), Handbook of qualitative research, Sage Publications, Thousand Oaks.

Desender, K.A. and Epure, M. (2013). Corporate Governance and Corporate Social Performance: The Influence of Boards, Ownership and Institutions. Barcelona GSE Working Paper Series nº 730

Dessain. V, Meier, O., and Salas, V. (2008) corporate governance and ethics: shareholder reality, social responsibility or institutional necessity? https://www.cairn.info/revue-management-2008-2-page-65.htm M@n@gement, **11 (2):** 65-79 Special Issue: Corporate Governance and Ethics

Devriese, J., Dewatripont, M., Heremans, D., and Nguyen, G. (2004). Corporate governance, regulation and supervision of banks, *Financial Stability Review*, **2:** 95–120.

DeYoung, R., Peng, E., and Yan, M. (2013). Executive compensation and business policy choices at US commercial banks, *Journal of Financial and Quantitative Analysis*, **48:** 165–196.

Dezso, C. L., and Ross, D. G. (2008). ''Girl power'': Female participation in top management and firm quality. Working paper: University of Maryland.

Diamond, D.W. and Dybvig, P.H. (1983) Bank runs, deposit insurance, and liquidity. *Journal of Political Economy* **91(3):** 401–419.

Dillenburg, S., Green, T., and Erekson, H. (2003). Approaching socially responsible investment with comprehensive rating scheme: Total social impact. *Journal of Business Ethics* **43:** 167–177.

DiMaggio, P. J., and Powell, W. W. (1983). The iron cage revisited: Institutional isomorphism and collective rationality in organisational fields. *American Sociological Review*, **48:** 147–160.

Dobbin F. and Zorn, D. (2005). Corporate Malfeasance and the Myth of Shareholder Value." *Political Power and Social Theory*, **17:** 179-98.

Dochartaigh, N.O. (2002) The Internet Research Handbook: A Practical Guide for Students and Researchers in the Social Sciences. London: Sage.

Domeher, D., Frimpong, J.M., and Appiah, T. (2014). Adoption of financial innovation in the Ghanaian banking industry. *African Review of Economics and Finance* **6(2):** 88–114

Donaldson, T., and Preston, L. E. (1995). The stakeholder theory of the corporation: concepts, evidence, and implications. *Academy of Management Review,* **20(1):** 65-91.

Dorger, M. (2011). Size Matters: Right Sizing Your Board of Directors. Retrieved from: http://dorgerconsulting.com/2011/07/20/size-matters-right-sizing-your-board-of-directors/

Dowell, G., Hart, S., and Yeung, B. (2000). Do corporate global environmental standards create or destroy market value? *Management Science* **46(8):** 1059–1074.

Eagly, A. H., and Carli, L. L. (2003). The female leadership advantage: An evaluation of the evidence. *Leadership Quarterly,* **14(6):** 807–834.

ECB (2003). Structural change and growth prospects in Asia – challenges to central banking, Speech by Solans E.D., Member of the governing council and of the executive board of the European Central Bank, delivered at the 38th SEACEN governors conference and 22nd meeting of the SEACEN board of governors, Manila, Philippines, http://www.ecb.europa.eu/press/key/date/2003/html/sp030213.en.html

Eccles, Ioannou, and Serafeim (2014). The Impact of Corporate Sustainability on Organisational Processes and Performance. *Management Science,* **60 (11):** 2835-2857.

Ehikioya, B.I. (2009). 'Corporate governance structure and firm performance in developing economies: evidence from Nigeria', *Corporate Governance*, **9(3):** 231–243.

Eisenbeiss, S. A. (2012). Re-thinking ethical leadership: An interdisciplinary integrative approach. *Leadership Quarterly*, **23(5):** 791–808.

Eisenbeiss, S.A., Knippenberg, D, and Farbrach (2015). Doing Well by Doing Good? Analyzing the Relationship between CEO Ethical Leadership and Firm Performance. *Journal of Business Ethics* **128:**635–651

Ekpu, V. (2015). Measuring and reporting financial innovation performance and its impact: A review of methodologies. Lecture Presented at a three-day seminar for Financial Regulators and Supervisors, organized by the West African Institute for Financial and Economic Management (WAIFEM) Dubai, UAE -Aug 2015

Elijido-Ten, E., Kloot, L., and Clarkson, P. (2010). Extending the application of stakeholder influence strategies to environmental disclosures: An exploratory study from a developing country. *Accounting, Auditing and Accountability Journal* **23 (8):** 1032–1059.

Elkington, J. (1997). Cannibals with Forks: The Triple Bottom Line of 21st Century Business. Capstone, Oxford.

Enendu, C.L, Abba, M.A., Fagge, A.I., Nakarji, M., Kure, E.U., Bewaji P.N., Nwosu, C.P., Ben-Obi, O.A., Adigun, M.A., Elisha, J.D., Okoro, A.E., and Ukeje, N.H. (2013). Bank Intermediation in Nigeria: Growth, Competition and Performance of the Banking Industry, 1990 – 2010 Central Bank of Nigeria – Occasional Paper No. 48

Enofe, A.O., Ekpulu, G.A., Onobun, S.I., and Onyeokweni, V.O. (2015) Ethical Challenges and Financial Performance in the Nigerian Banking Sector. Research Journal of Finance and Accounting. **6 (10):** 1-11

European Commission (2011). Communication from the commission of the European Communities concerning Corporate Social Responsibility: A business contribution to Sustainable Development "Promoting a European Framework for Corporate Social Responsibility"

Evan, D. (1987). The relationship between firm growth, size, and age: Estimates for 100 manufacturing companies. *The Journal of Industrial economics*, :567-581.

Evangelinos K.I, and Nikolaou, I.E. (2009). Environmental accounting and the banking sector: a framework for measuring environmental–financial risks. *International Journal of Services Sciences* **2(3/4):** 366.

Evans, J.D. (1996). Straightforward Statistics for the Behavioral Sciences. Brooks/Cole Publishing; Pacific Grove, Calif.

Fama, E.F. and Jensen, M.C. (1983), 'Agency Problems and Residual Claims', *Journal of Law and Economics*, **26**: 327–49.

Fama, E.F. (1980). Agency problems and the theory of the firm, *Journal of Political Economy*, **88**: 288–307.

Fanta, K. Kemal, K., and Waka, Y. (2013). Corporate Governance and Impact on Bank Performance, *Journal of Finance and Accounting*, **1(1):** 19-26.

Fauzi, H., Mahoney, L., and Rahman, A.A. (2007). Institutional Ownership and Corporate Social Performance: Empirical Evidence from Indonesian Companies. *Issues in Social and Environmental Accounting* **1 (2):** 334-347

Fauzi, H., Svensson, G., and Rahman, A.A. (2010). "Triple Bottom Line" as "Sustainable Corporate Performance": A Proposition for the Future. *Sustainability* **2:** 1345-1360

Fernandes, N. and Fich, E.M. (2009) Does financial experience help banks during credit crises? https://www.cemfi.es/ftp/pdf/papers/wshop/WPM$60DE.pdf. (Accessed on October 15, 2016).

Ferrarini, G. (2017). Understanding the Role of Corporate Governance in Financial Institutions: A Research Agenda. European Corporate Governance Institute (ECGI) Law Working Paper N° 347/2017. 1-26. Available https://ssrn.com/abstract=2925721

Fisman, R., Heal, G. and Nair, V. (2006). 'A Model of Corporate Philanthropy', Working Paper (Wharton School, University of Pennsylvania).

Fitzgerald, L., and Storbeck, J. E. (2003). Pluralistic views of performance. *Management Decision*, **41(8):** 741-750.

Flannery, M.J. (1998) Using market information in prudential bank supervision: a review of the U.S. empirical evidence. *Journal of Money, Credit and Banking* **30(3):** 273–305.

Fombrun, C.J. (1996). Reputation: Realizing Value from the Corporate Image. Boston: Harvard Business School Press.

Fombrun, C.J., Gardberg, N.A. and Sever, J.M. (2000). The reputation quotient: a multiple stakeholder measure of corporate reputation. *Journal of Brand Management*, **7(4):** 241–255.

Forbes (2013). The Facts of Family Business Retrieved from: https://www.forbes.com/sites/aileron/2013/07/31/the-facts-of-family-business/#606fb0e49884

Forbes, D.P. and Milliken, F.J. (1999) Cognition and corporate governance: understanding boards of directors as strategic decision-making groups. *Academy of Management Review* **24(3):** 489–505.

Fornell, C., and Larcker, D. F. (1981). Evaluating structural equations with unobservable variables and measurement error. *Journal of Marketing Research*, **18:** 39–50.

Frame, W. S. and White, L J. (2002). Empirical Studies of Financial Innovation: Lots of Talk, Little Action? (Working Paper 2002-12). Atlanta, GA, USA: Federal Reserve Bank of Atlanta: 1-40

Frankfort-Nachmias, C and Nachmias, D. (2008). Research methods in the social sciences, 7th edn, Worth Publishers, New York.

Freeman, R.E. and Reed, D. (1983) ‘Stockholders and Stakeholders: A New Perspective on Corporate Governance’, *California Management Review*, **25 (3):** 88-106.

Freeman, R.E. (1984) Strategic Management: A Stakeholder Approach, Boston: Pitman-Ballinger.

Freeman, R.E. (2001) The stakeholder approach revisited

Friedman, M. (1970). The social responsibility of business is to increase its profits. New York Times Magazine, 13 Sept., **32 (33):** 122-126.

Fulbert, T.T. (2008). Regulation and Banking Stability: A Survey of Empirical Studies

Fulmer, R. M. (2004). The challenge of ethical leadership. Organisational Dynamics**, 33(3):** 307-317.

Fung, S., and Tsai, S. (2012). Institutional Ownership and Corporate Investment Performance. *Canadian Journal of Administrative Sciences/Revue Canadienne des Sciences de l'Administration*, **29 (4):** 348-365.

Galbreath, J. (2012). Are boards on board? A model of corporate board influence on sustainability performance. *Journal of Management and Organization* **18** (4): 445-460.

Gberevbie, D.E. (2011). Leadership, the financial sector and development in Nigeria. Inkanyiso, Jnl Hum and Soc Sci **3(2):**148-157

Gefen, D., Straub, D. W., and Boudreau, M.C. (2000). “Structural Equation Modeling and Regression: Guidelines for Research Practice,” *Communications of the Association for Information Systems* **4(7):**1-70.

Gentile, M. C. (2010). Keeping Your Colleagues Honest. Harvard Business Review, **88(3):** 114-117.

George, D. and Mallery, P. (2003). SPSS for windows step by step. A simple guide and reference. 11.0 update (4 th ed.). Boston, M.A: Allyn and Bacon, ISBN-13: 978-0205375523, ISBN-10:0205375529

Ghauri, P. and Grønhaug, K. (2005) Research Methods in Business Studies: A Practical Guide (3rd edn). Harlow: Financial Times Prentice Hall.

Ghillyer A. (2014). Business Ethics Now, 4th edition, McGraw-Hill Education, New York.

Ghosh, C. and Sirmans, C.F. (2003) "On REIT Compensation: Does Board Structure Matter?" Paper presented at the Cambridge-Maastricht real estate conference, June.

Gibbs, G. R. (2007). Analyzing qualitative data. In U. Flick (Ed.), The Sage qualitative research kit. London: Sage.

Gibson, K. and O'Donovan, G. (2007). Corporate governance and environmental reporting: An Australian study. Corporate Governance: An International Review, **15(5):** 944-956.

Gillibrand, M. (2004). Corporate Management Essential for Industrialization, The Bangladesh Observer.

Gilmore, S., and Sillince, J. (2014). Institutional theory and change: the deinstitutionalisation of sports science at Club X. *Journal of Organisational Change Management*, **27(2):** 314-330.

Glassman, C. and Rhoades, S. (1980) Owner vs manager control effects on bank performance. *Review of Economics and Statistics* **62:** 263–270.

Glick, W. H., Washburn, N. T., and Miller, C. C. (2005). The myth of firm performance. Proceedings of the Annual Meeting of American Academy of Management. Honolulu, Hawaii.

Global Reporting Initiative. (2011). G3.1 Sustainability Reporting Guidelines. Global Reporting Initiative.

Gomez-Mejia, L.R. and Wiseman, R.M. (2007). Commentary: Does agency theory have universal relevance? A reply to Lubatkin, Lane, Collin, and Very. *Journal of Organisational Behaviour J. Organiz. Behav.* **28:** 81–88

Gompers, P., J. Ishii, and Metrick, A. (2003). Corporate Governance and Equity Prices. *The Quarterly Journal of Economics* **118 (1):** 107-155.

Gordon, J.N. (2007). "The Rise of Independent Directors in the United States, 1950-2005: Of Shareholder Value and Stock Market Prices." *Stanford Law Review*, **59:** 1465-1568.

Graffin, S., Pfarrer, M., and Hill, M. (2012). Untangling executive reputation and corporate reputation: Who made who? In M. L. Barnett, and T. G. Pollock (Eds.), The Oxford handbook of corporate reputation: 221–239. Oxford, U.K.: Oxford University Press.

Gregory, B.T., Rutherford, M.W., Oswald, S. and Gardiner, L. (2005). ''An empirical investigation of the growth cycle theory of small firm financing'', *Journal of Small Business Management*, **43(4):** 382-92.

Gregory-Smith, I., Main, B. G. M., and O'Reilly, C. A. (2014). Appointments, Pay and Performance in Uk Boardrooms By Gender. *The Economic Journal*, 124.

Groom, B. (2011). Bosses put profit before ethics, says survey. Financial Times. Retrieved from http://www. ft.com/intl/cms/s/0/ceb08890-edd8-11e0-acc7-00144feab49a. html#axzz2w9htlolX. Accessed 21 November, 2016.

Grove, H., Patelli, L., Victoravich, L.M. and Xu, P. (2011) corporate governance and performance in the wake of the financial crisis: evidence from US commercial money banks. *Corporate Governance: An International Review* **19(5):** 418–436.

Guest, G., Bunce, A., and Johnson, L. (2006) 'How many interviews are enough? An experiment with data saturation and validity', *Field Methods,* **18(1):** 59–82.

Guillen, M. (2002). A global view of corporate governance: one size doesn't fit all. Retrieved from: http://knowledge.wharton.upenn.edu/article/a-global-view-of-corporate-governance-one-size-doesnt-fit-all/. Accessed 15 November, 2016

Guo, L., Smallman, C., and Radford, J. (2013). A critique of corporate governance in China. *International Journal of Law and Management,* **55:** 257-272. doi:10.1108/IJLMA-10-2011-0012

Guo, Z. and Kumara, U. (2012). Corporate Governance and Firm Performance of Listed Firms in Sri Lanka. *Asia pacific business innovation and technology management society*. **40:** 664-667

Guthrie, J. P., (2001). High-involvement Work Practices, Turnover, and Productivity: Evidence from New Zealand, *Academy of Management Journal* **44:** 180-190.

Haan, J, and Vlahu, R. (2016). Corporate governance of banks: *A Survey Journal of Economic Surveys* **30(2):** 228–277

Hambrick, D. (2007). Upper Echelons theory: an update. *Academy of Management Review* **32:** 334–343.

Hambrick, D.C. and Jackson, E.M. (2000). Outside directors with a stake: The linchpin in improving governance. *California Management Review* **42 (4):** 108-127.

Hambrick, D.C. and Mason, P.A. (1984). Upper Echelons: The Organisation as a Reflection of Its Top Managers. *Academy of Management Review* **9(2):** 193-206

Haniffa, R., and Cooke, T. E. (2002). Culture, corporate governance and disclosure in Malaysian corporations. Abacus, **3:** 317-349

Haniffa, R.; Cooke, T. (2005). The Impact of Culture and Governance on Corporate Social Reporting. *J. Account. Public Policy*, **24**: 391–430

Haniffa, R. and Hudaib, M. (2007). 'Locating Audit Expectations Gap within a Cultural Context: The Case of Saudi Arabia', *Journal of International Accounting, Auditing and Taxation,* **16(2):**179-206.

Haque, F. and Arun, T.G. (2016). Corporate governance and financial performance: an emerging economy perspective. Investment Management and Financial Innovations, **13(3):**228-235

Harjoto, M.A. and Jo, H. (2011). Corporate Governance and CSR Nexus. *Journal of Business Ethics* **100:**45–67

Harris, M. and Raviv, A. (2008) A theory of board control and size. *Review of Financial Studies* **21(4):** 1797–1832.

Hart, O.D. (1995). Firms, Contracts and Financial Structure, Oxford University Press, Oxford.

Harter, J. K., Schmidt, F. L., and Hayes, T. (2002). Business-unit-level relationship between employee satisfaction: a meta-analysis. *Journal of Applied Psychology* **87(2):** 268-279.

Hashim, H. A., and Devi, S. S. (2010). Corporate governance, ownership structure and earnings quality: Malaysian evidence. Working Paper, University Malaya.

Hatch, M.J. and Schultz, M. (2001). Are the strategic stars aligned for your corporate brand? *Harvard Business Review*, **79(2):** 128–134.

Hausman, J.A. (1978). Specification tests in econometrics. *Econometrica* **46 (6):** 1251-1271.

Hausman, J.A., and Taylor, W.E. (1981). Panel data and unobservable individual effects. *Econometrica*, **49 (6):** 1377-1398.

Haw, I., Ho, S., Hu, B., and Wu, D. (2010). Concentrated control, institutions, and banking sector: An international study, *Journal of Banking and Finance* **34:** 485–497.

Hayes, R., Mehran, H., and Schaefer, S. (2004). "Board Committee Structures, Ownership, and Firm Performance". A Revised Version of the Paper Presented at the Federal Reserve Bank of New York Finance Seminar Series, at New York University.

Heentigala, K. and Armstrong, A. (2011). The Impact of Corporate Governance on Firm Performance in an Unstable Economic and Political Environment: Evidence from Sri Lanka. Retrieved from http://papers.ssrn.com/sol3/papers.cfm?abstract_id=1971927

Henderson, B. J. and Pearson, N. D., 2011. The dark side of financial innovation: a case study of the pricing of a retail financial product. Journal of Financial Economics, **100:** 227-47.

Hendry, J. (2001) 'Missing the target: Normative stakeholder theory and the corporate governance debate'. *Business Ethics Quarterly*, **11 (1):** 159-176.

Henri, J.F. and Journeault, M. (2008) 'Environmental performance indicators: An empirical study of Canadian manufacturing firms'. *Journal of Environmental Management* **87:** 165-176

Henriques, I., and Sadorsky, P. (1999). The relationship between environmental commitment and managerial perceptions of stakeholder importance. *Academy of Management Journal*, **42:** 87–99.

Herbohn, K., Walker, J. and Loo, H.Y.M. (2014). "Corporate social responsibility: The link between sustainability disclosure and sustainability performance", *Abacus* **50(4):** 422-459.

Hermann, B., Kroeze, C. and Jawjit, W. (2007) 'Assessing environmental performance by combining life cycle assessment, multicriteria analysis and environmental performance indicators'. *Journal of Cleaner Production* **15:** 1787-1796

Herrigel, G. (2006) Corporate Governance: History without Historians. Paper prepared for Geoffrey Jones and Jonathan Zeitlin, eds. Handbook of Business History (Oxford: Oxford University Press, 2006-forthcoming):1-35

Hess, G. (1996). Zum Stand der Diskussion in den Vereinigten Staaten. In Feddersen, D., Hommelhoff, P. and Schneider, U. H. (eds.). Corporate Governance. Otto Schmidt-Verlag, Köln.

Hilb, M. (2006). New corporate governance: successful board management tools. 2nd ed. Switzerland: Springer

Hill, R. P., Ainscough, T., Shank, T. and Manullang, D. (2007). 'Corporate Social Responsibility and Socially Responsible Investing: A Global Perspective', *Journal of Business Ethics* **70:** 165–174.

Hillman, A. J., and Dalziel, T. (2003). Boards of directors and firm performance: Integrating agency and resource dependence perspectives. *Academy of Management Review* **28:** 383-396.

Hillman, A.J., Canella, A.A., and Paetzold, R.L. (2000). "The Resource Dependency Role of Corporate Directors: Strategic Adaptation of Board Composition in Response to Environmental Change". *Journal of Management Studies* **37 (2):** 235-255

Hirshleifer, D. (1993). Managerial reputation and corporate investment decisions. *Financial Management* **22(2):** 145–160.

Ho, C.K. (2005). 'Corporate governance and corporate competitiveness: an international analysis', Corporate Governance: *An International Review* **13 (2):** 211–253.

Hoelscher, D.S., Taylor, M., and Klueh, U.H. (2006). The Design and Implementation of Deposit Insurance Systems, International Monetary Fund, Washington D.C., US.

Hopkins, M. (2002). CSR and Global Business Principles: What a Mess! MHC International Corporate and Social Research. Retrieved from: https://pdfs.semanticscholar.org/57e2/2989c2cf2c3d6733a0a514db53bf2fcabb8e.pdf

Hopkins, M. (2001). 'What, if any, is the Relation between Corporate Governance and Corporate Social Responsibility?' World Bank Monthly Report January.

Hoyle, K. and Ingram, R. (1991). Statistics for Business (Made Simple Books).

Hu, Y., and Izumida, S. (2008). Ownership concentration and corporate performance: A causal analysis Japanese panel data. *An International Review*, **16 (4):** 342-358.

Huang (2013). The Impact of CEO Characteristics on Corporate Sustainable Development. Corporate Social Responsibility and Environmental Management Corp. Soc. Responsib. *Environ. Mgmt*. **20:** 234–244

Huson, M. R., Malatesta, P. H., and Parrino, R. (2004). Managerial succession and firm performance. *Journal of Financial Economics*, **74(2):** 237–275.

Hussain, A., Obaid, Z., and Khan, S. (2014). CEO Compensation Determinants: "Is the Size or Performance of the Firm a determinant of CEO Compensation in Pakistan. PUTAJ – *Humanities and Social Sciences* **21(1)**

Hussey, J., and Hussey, R. (1997). Business Research: A Practical Guide for Undergraduate and Postgraduate Students. Macmillan Press, London.

Hutton, W. (1995) The State We're in: Why Britain Is in Crisis and How to Overcome It, London: Random House UK.

Iannotta, G., Nocera, G. and Sironi, A. (2007) Ownership structure, risk and performance in the European banking industry. *Journal of Banking and Finance* **31:** 2127–2149.

IFC (2005). Developing Value The business case for sustainability in emerging markets Retrieved from: http://www.ifc.org/wps/wcm/connect/84a59480488559ca842cd66a6515bb18/Developing_Value_full.pdf?MOD=AJPERES

Ikpefan, O.A. (2013). Corporate Governance as a Tool for Curbing Bank Distress in Nigeria commercial Banks: Empirical Evidence. *Research Journal of Finance and Accounting*, **4(13):** 41-51

Imeokparia, L. (2013). Corporate governance and financial reporting in the Nigerian banking sector: an empirical study. Asian Economic and Financial Review, 2013, **3(8):**1083-1095

Irshad, R, Hashmi, H.H., Kausar, S., and Nazir, M.I. (2015) Board Effectiveness, Ownership Structure and Corporate Performance: Evidence from Pakistan. *Journal of Business Studies Quarterly* **7 (2):** 46-60

Isaac, S., and Michael, W. B. (1981). Handbook in research and evaluation· A collection of principles, methods, and strategies useful in the planning, design, and evaluation of studies in education and the behavioral sciences (2nd ed.). San Diego, CA: EdiTS.

Islam, M.A. (2014). An Analysis of the Financial Performance of National Bank Limited Using Financial Ratio. Journal of Behavioural Economics, Finance, Entrepreneurship, Accounting and Transport, **2 (5):** 121-129

Isukul, A.C. and Chizea, J.J. (2017). Corporate Governance Disclosure in Developing Countries: A Comparative Analysis in Nigerian and South African Banks. SAGE Open July-September 2017: 1–17 DOI: 10.1177/2158244017719112

Iszatt-White, M. (2011). Methodological crisis and contextual solution: An ethnomethodologically informed approach to understand leadership. *Leadership*, **7:** 119-135. doi:10.1177/1742715010394734

Iturralde, T., Maseda, A., and Arosa, B. (2011). Insiders Ownership and Firm Performance. Empirical Evidence. *International Research Journal of Finance and Economics*, **67(120).**

Jackson, G. (2001) 'The origins of nonliberal corporate governance in Germany and Japan', in W. Streeck and K. Yamamura (eds.) The Origins of Nonliberal Capitalism: German and Japan, Ithaca: Cornell University Press, 121-170.

Jalilian, H., Kirkpatrick, C., and Parker, D. (2007). The Impact of Regulation on Economic Growth in Developing Countries: A Cross-Country Analysis", *World Development* **35(1):** 87-103.

Janggu, T., Darus, F., Zain, M., and Sawani, Y. (2014). Does good corporate governance lead to better sustainability reporting? An analysis using structural equation modeling. *Procedia—Social and Behavioral Sciences*, **145:** 138-145 http://dx.doi.org/10.1016/j.sbspro.2014.06.020

Janis, I.L. (1983) Groupthink: Psychological Studies of Policy Decisions and Fiascoes, 2nd edn. Boston, MA: Houghton Mifflin.

Jankowicz, A.D. (2005) Business Research Projects (4th edn). London: Thomson Learning.

Jensen M. C., (1993). "The Modern Industrial Revolution, Exit and the Failure of Internal Control Systems", *Journal of Finance*, :831-880

Jensen M.C, and Murphy K.J. (1990a). Performance pay and top management Incentives. *J. Polit. Econ.* **98(2):**225-264

Jensen M.C, and Murphy KJ. (1990b). CEO incentives: it's not how much, but how. *Harvard Bus. Rev.* **68(3):**138-53

Jensen, M. (1986). Agency cost of free cash flow, corporate finance, and takeovers. Corporate Finance and Takeovers. *American Economic Review*, **76 (2).**

Jensen, M. (2001) 'Value maximisation, stakeholder theory, and the corporate objective function'. *Journal of applied corporate finance*, **14(3):** 8-21.

Jensen, M. (2010) ‘Value maximisation, stakeholder theory, and the corporate objective function’. *Journal of Applied Corporate Finance* **22(1):** 32-42.

Jensen, M.C. and Meckling, W.H. (1976) Theory of the Firm: Managerial Behavior, Agency Costs and Ownership Structure. *Journal of Financial Economics* **3(4):** 305-360

Jeucken, M. H. A. (2004) Sustainability in Finance- A retroductive exploration, PhD-thesis Erasmus University Rotterdam, Eburon, Delft.

Jiraporn, P., Kim, Y. S. and Davidson, W. N. (2008). Multiple directorships and corporate diversification. *Journal of Empirical Finance*. **15(3):** 418-435.

Johl, S. Bruce, A. and Binks, M. (2012). ‘A Study on the Use of Mixed Method Approach via Sequential Procedure to Investigate Corporate Governance in Corporate Entrepreneurship among the 100 UK Financial Times Stock Exchange (FTSE) Companies’, *African Journal of Business Management*, **6 (21):** 6369-6377.

John, K. and L.W. Senbet (1998). Corporate Governance and Board Effectiveness, *Journal of Banking and Finance*, **22(4):** 371–403.

Johnson, R. D. A., and Greening, D. W. (1999). The effects of corporate governance and institutional ownership types on corporate social performance. *Academy of Management Journal* **42(5):** 564-576.

Jones, P., Hillier, D. and Comfort, D. (2017). The Sustainable Development Goals and the Financial Services Industry. Athens Journal of Business and Economics, **3 (1):** 37-50

Jones, M. T., and Millar, C. C. (2010). "About Global Leadership and Global Ethics, and a Possible Moral Compass: An Introduction to the Special Issue", *Journal of Business Ethics* **93(1)**

Jones, T. (1995) ‘Instrumental stakeholder theory: A synthesis of ethics and economics’. *Academy of Management Review* **20(2):** 404-437.

Joseph. P, and Loretta. J. (2013). Working Paper No. 13-31 measuring the performance of banks: Theory, Practice, Evidence, and Some Policy Implications

Juras, P.E. and Hinson, Y.L. (2008). Examining the effect of board characteristics on agency costs and selected performance measures in banks. *Academy of Banking Studies Journal* **7(2)**

Kajola, S.O. (2008). Corporate Governance and Firm Performance: The Case of Nigerian Listed Firms. *European Journal of Economics, Finance and Administrative Sciences* (**14):** 16-28

Kalshoven, K., Den Hartog, D. N., and De Hoogh, A. H. B. (2011). Ethical Leadership at Work Questionnaire (ELW): Development and validation of a multidimensional measure. *Leadership Quarterly* **22(1):** 51–69.

Kaplan, D. (2000). Structural equation modelling: Foundations and extensions. Thousand Oaks, CA: Sage.

Kaplan Financial Limited (2012). Governance. Retrieved from http://kfknowledgebank.kaplan.co.uk/KFKB/Wiki%20Pages/Sarbanes-Oxley%20(SOX).aspx. Accessed on 20 December, 2016.

Kaplan, R. S. and Norton, D.P. (1992). The Balanced Scorecard: Measures that Drive Performance, Harvard Business Review, (January-February): 71-79.

Kassinis, G., and Vafeas, N. (2002). Corporate boards and outside stakeholders as determinants of environmental litigation. *Strategic Management Journal* **(23):** 399-415.

Kerlinger, F.N. and Rint, N (1986). Foundations of Behaviour Research. Lomdon: Winston Inc

Ketokivi, M. A., and Schroeder, R. G. (2004). Perceptual measures of performance: fact or fiction? *Journal of Operations Management* **22(3):** 247-264.

King'ang'ai, P.M., Kigabo, T., Kihonge, E. and Kibachia, .J. (2016). Effect of agency banking on financial performance of commercial banks in Rwanda. A study of four commercial banks in Rwanda. European Journal of Business and Social Sciences, **5(1):** 181 - 201

Kitchen, P.J. and Laurence, A. (2003). Corporate Reputation: An Eight-Country Analysis. *Corporate Reputation Review* **2 (4):** 363-381.

Klapper, L. F., and Love, I. (2003). Corporate Governance, Investor Protection, and Performance in Emerging Markets, *Journal of Corporate Finance* **(195):** 1-26.

Klassen, R.D. and McLaughlin, C.P. (1996). The Impact of Environmental Management on Firm Performance. *Management Science*. **42 (8):** 1199-1214

Klettner, A., Clarke, T., and Boersma, M. (2014). The Governance of Corporate Sustainability: Empirical Insights into the Development, Leadership and Implementation of Responsible Business Strategy. *J Bus Ethics* **(122):**145-165. http://dx.doi.org/10.1007/s10551-013-1750-y

Kocmanova, A. and Docekalova, M. (2011). Corporate sustainability: Environmental, social, economic and corporate performance. Acta univ. agric. et silvic. Mendel. Brun., **59 (7):** 203-208

Kor, Y. Y., and Sundaramurthy, C. (2009). Experience-based human capital and social capital of outside directors. *Journal of Management* **(35):** 981-1006.

Krejcie, R.V., and Morgan, D.W. (1970). Determining Sample Size for Research Activities. *Educational and Psychological Measurement* **(30):** 607-610

Kremmling, M.D. (2011). The Influence of Financial Sector Regulation on Bank Performance

Krishnan, H.A., and Park, D. (2005). A Few Good Women—On Top Management Teams. *Journal of Business Research* **58(12):**1712-1720 DOI10.1016/j.jbusres.2004.09.003

Kumar, K. (2013). Sustainability performance measurement: an investigation into corporate best practices. A doctorate degree thesis, School of Public Administration, National Institute of Development Administration

Kurawa, J.M. and Saidu, S.K. (2014) Executive Compensation and Financial Performance of Listed Banks in Nigeria: An Empirical Analysis. *Research Journal of Accounting* **2(3)**

Kyereboah-Coleman, A. and Biekpe, N. (2005). Corporate Governance and the Performance of Microfinance Institutions (MFIs) in Ghana. Working Paper, UGBS, Legon.

Kyereboah-Coleman, A. (2007). Corporate governance and firm performance in Africa: a dynamic panel data analysis. A Paper Prepared for the "International Conference on Corporate Governance in Emerging Markets"

Kyereboah-Coleman, A. (2007). Corporate Governance and Shareholder Value Maximisation: An African Perspective. African Development Review, Blackwell Publishing Ltd, Oxford, UK, 351-367.

La Porta, R., Lopez-de-Silanes F., Shleifer A., and Vishny, R., (2000). Investor Protection and Corporate Valuation. *Journal of Finance* **(57):** 1147-1170.

Laeven, L. and Levine, R. (2009). “Bank Governance, Regulation and Risk Taking” *Journal of Financial Economics* **93(2)**: 259-275

Laeven, L. and R. Levine, (2007). “Is There a Diversification Discount in Financial Conglomerates?” *Journal of Financial Economics* **(85):** 331-367.

Laeven, L. (2012). Corporate governance: what’s special about banks? Keynote lecture at the DNBCGIC conference on corporate governance of financial institutions, Amsterdam, November 2012.

Lasfer, M. A. (2002). “Board Structure and Agency Costs”. Discussion Paper, City University Business School, Barbican Centre, London.

Lerner J., and Tufano P. (2011) The Consequences of Financial Innovation: A Counterfactual Research Agenda, NBER Working Paper 16780, February.

Li, L. and Song, F.M. (2013). Do bank regulation affect board independence? Across-country analysis. *Journal of Banking and Finance* **(37):** 2714–2732.

Liang, N. and J. Li. (1999). “Board Structure and Firm Performance: New Evidence from China’s Private Firms”. Paper presented at the Academy of Management Annual Conference, Chicago, USA, 7-10 August.

Libecap, G.D. (1989). Contracting for Property Rights (New York, NY: Cambridge University Press).

Lin, D, Kuo, H-C, and Wang, L-H (2013). Chief executive Compensation: an empirical study of fat cat CEOs. *The International Journal of Business and Finance Research* **7(2):** 27-42

Lipton, M., and Lorsch, J.W. (1992). A modest proposal for improved corporate governance. *Business lawyer* **(48):** 59-77.

Liu, Z., and Zhuang, W. (2013). Governance of global supply chains vulnerability by business-based interorganisational information platform. J Syst Sci Syst Eng, 22(1), 1-20. http://dx.doi.org/10.1007/s11518-013-5211-z

Ljubojevic, C and Ljubojevic, G.  (2008). Building Corporate Reputation through Corporate Governance. *Management* **3 (3):** 221–233

Llewellyn, D. (2010). The global banking crisis and the post-crisis banking and regulatory scenario. Research papers in corporate finance. University of Amsterdam, June, 2010.

Llewellyn, D., (1999), “The Economic Rational for Financial Regulation”, FSA

Love, E.G. Lim, J. and Bednar, M .K (2017). The face of the firm: the influence of CEOs on corporate reputation. Academy of Management Journal, 60 (4): 1462–1481. https://doi.org/10.5465/amj.2014.0862

Luo, X., and Bhattacharya, C. B. (2006). Corporate social responsibility, customer satisfaction, and market value. *Journal of Marketing* **(70)**: 1-18.

MacDonald, S.S and Koch, T.W (2006). Management of Banking, 6th edition, Thomson, South Western, USA.

Macey, J.R. and O’Hara, M (2003). The corporate governance of banks. FRBNY *Economic Policy Review* / **April** :91-107

Maher, M and Andersson, T. (1999). Corporate governance: Effects on firm performance and Economic growth.

Majeed, S, Aziz, T and Saleem, S (2015). The Effect of Corporate Governance Elements on Corporate Social Responsibility (CSR) Disclosure: An Empirical Evidence from Listed Companies at KSE Pakistan. Open Access *Int. J. Financial Stud*. **3(4):** 530-556; doi:10.3390/ijfs3040530

Manawaduge, A. and Zoysa, A. (2013). The structure of corporate ownership and firm performance: Sri Lankan evidence. *Corporate Ownership and Control* **11(1):** 723-734.

Martin, A. D., Nishikawa, T., and Williams, M. A. (2009). CEO gender: Effects on valuation and risk. *Quarterly Journal of Finance and Accounting* **48(3):** 23–40.

Martinez, R., Ruiz, C., and Ruiz, P. (2011). "Improving the 'Leader-Follower' Relationship: Top Manager or Supervisor? The Ethical Leadership Trickle-Down Effect on Follower Job Response", *Journal of Business Ethics,* **99(4):** 587-608.

Masouros, P.E. (2014). Corporate Governance and the Great Recession: An Alternative Explanation for Germany's Success in the Post2008 World. Research Paper No 8/2014. Retrieved from: http://ssrn.com/abstract=2388611 Accessed 11 November, 2016.

Mathiesen, H. (2002). Managerial Ownership and Financial Performance. Ph.D. dissertation Thesis. Advisor professor S. Thomsen, Copenhagen, Copenhagen Business School Department of International Economics and Management.

Matten, D., Crane, A. (2005). Corporate citizenship: toward an extended theoretical conceptualization. *Academy of Management Review* **30(1):** 166–179.

Maxwell, J. A. (2005). Qualitative research design: An interactive approach. (2nd edn) Sage

Mayer, D. M., Kuenzi, M., Greenbaum, R., Bardes, M., and Salvador, R. (2009). How does ethical leadership flow? Test of a trickledown model. *Organizational Behavior and Human Decision Processes*, **108**, 1–13.

McKinsey and company (2002). Global Investor Opinion Survey: Key Findings

McNulty, T. Zattoni, A. and Douglas, T. (2013). 'Developing Corporate Governance Research through Qualitative Methods: A Review of Previous Studies', Corporate Governance: An International Review, **21 (2):**183-198.

McNulty, T., Zattoni A., and Douglas, T. (2013). Developing Corporate Governance Research Through Qualitative Methods: A Review of Previous Studies. *Corporate Governance an International Review* **21(2)** March 2013. DOI: 10.1111/corg.12006

McWilliams, A., and Siegel, D. (2000). Corporate social responsibility and financial performance: Correlation or misspecification? *Strategic Management Journal* **21 (5):** 603–609.

Mehran, H., Morrison, A. and Shapiro, J. (2011). Corporate governance and banks: what have we learned from the financial crisis? Federal Reserve Bank of New York Staff Report 502.

Menard, S. (1995). Applied logistic regression analysis. Thousand Oaks: Sage University paper series on quantitative applications in the social sciences, 07-106.

Mercier, S. (2004). L'éthique dans les entreprises, Nouvelle édition, Paris: La Découverte.

Michelon, G. and Parbonetti, A. (2012). The effect of corporate governance on sustainability disclosure. Journal of Management and Governance, **16(3):** 477-509.

Mihelic, K.K, Lipicnik, B. and Tekavcic, M. (2010). Ethical Leadership. *International Journal of Management and Information Systems* – **14(5)**: 31-42

Milgrom, P. and J. Roberts (1992). Economics, Organisation and Management, Englewood Cliffs, NJ: Prentice Hall.

Millstein, I.M. (1998). 'The Evolution of Corporate Governance in the United States', Remarks to the World Economic Forum, Davos, Switzerland (February 2, 1998).

Minton, B.A., Taillard, J.P.A. and Williamson, R. (2010) Board composition, risk taking and value: Evidence from financial firms. https://www.eurofidai.org/Taillard_2010.pdf. (Accessed on October 15, 2016).

Mishra, C. S. and Nielsen, J.F. (2000). "Board Independence and Compensation Policies in Large Banks Holding Companies", *Financial Management*, **29(3):**51-70.

Mishra, S., and Mohanty, P. (2014). Corporate governance as a value driver for firm performance: Evidence from India. *Corporate Governance*, **14**: 265-280. doi:10.1108/CG-12-2012-0089

Mitchell, R., Agle, B. and Wood, D. (1997). Toward a Theory of Stakeholder Identification and Salience: Defining the Principle of Who and What Really Counts. *The Academy of Management Review*, **22 (4):**853-886. Available at: http://links.jstor.org/sici?sici=0363-7425%28199710%2922%3A4%3C853%3ATATOSI%3E2.0.CO%3B2-0 [Accessed: 20 November, 2016].

Mnasri, K. (2015). Ownership structure, board structure and performance in the Tunisian banking industry. *Asian academy of management journal of accounting and finance*. **11 (2):** 57–82

Mohamad, S. (2004). The importance of effective corporate governance. SSRN Electronic Journal.

Monks, R.A.G and Minnow, N. (2008). Corporate Governance, 4th Ed John Wiley and Sons, Ltd

Montiel, I. and Delgado-Ceballos, J. (2014). "Defining and measuring corporate sustainability: Are we there yet?" *Organisation and Environment*, **27(2):** 113-139.

Morck, R. and Yeung, B. (2003) "Agency Problems in Large Family Business Group" Entrepreneurship Theory and Practice, Baylor University

Morck, R., Shleifer, A. and Vishny, R.W. (1988). Management ownership and market valuation: an empirical analysis, *Journal of Financial Economics* **20**: 293-315.

Morrison, J. (2004) 'Legislating for good corporate governance' *Journal of Corporate Citizenship*, **15:** 121-133.

Morse, J. (2000). Determining sample size. *Qualitative health research*, **10:** 3-5

MSCI (2017). MSCI-ESG rating. Retrieved from https://www.msci.com/esg-rating-client-segmentation 17 September, 2017

Mueller, D.C. (2006). Corporate Governance and Economic Performance. International Review of Applied Economics, 20 (5): 623–643

Mullineux, A. (2006). "The Corporate Governance of Banks", *Journal of Financial Regulation and Compliance*, **14 (4 0):** 375-382.

Mwangi, M.K. (2007). Factors Influencing Financial Innovation in Kenya‟s Securities Market: A Study of Firms Listed at the NSE. Unpublished Master of Business Administration Project, University of Nairobi.

Myers, R.H. (1990). Classical and modern regression application. 2nd edition. Duxbury press. CA

Nam, S-W, and Nam, I.C. (2004). Corporate governance in Asia. Recent Evidence from Indonesia, Republic of Korea, Malaysia, and Thailand.

NDIC (2009). NDIC Annual Report 2009.

Neely, A., Gregory, M. and Platts, K. (1995). Performance Measurement System Design: A Literature Review and Research Agenda. *International Journal of Operations and Production Management*. **15 (4):** 80-116.

Neves, P. and Story, J. (2013). Ethical Leadership and Reputation: Combined Indirect Effects on Employee Organizational Deviance, Journal of Business Ethics 127(1):1-15

Ngaoga, R. (2014). Executive remuneration nexus and corporate performance in Kenya, *Journal of Finance,* **8(4):** 11-22.

Nidasio, C. (2006). The Integration of Corporate Responsibility into Business Strategy: Theoretical Perspectives and Empirical Evidence (September 20, 2006). European Academy of Business in Society 5th Colloquium (Bocconi University, 2006). Available at
SRN: https://ssrn.com/abstract=2379387 or http://dx.doi.org/10.2139/ssrn.2379387

Nordberg, D. (2007). The ethics of corporate governance.

North, D. (2005) Understanding the Process of Economic Change. Princeton, NJ: Princeton University Press

Ntim C.G., and Osei K.A. (2011). The impact of corporate board meetings on corporate performance in South Africa, *African Review of Economics and Finance*, **2(2):** 83-103.

Ntim, C.G. (2009). Internal Corporate Governance Structures and Firm Financial Performance: Evidence from South African Listed Firms. Thesis Submitted in Fulfilment of the Requirements for the Degree of Doctor of Philosophy in Finance Department of Accounting and Finance Faculty of Law, Business and Social Sciences University of Glasgow

Nyor, T. and Mejabi, S.K. (2013). Impact of Corporate Governance on Non-Performing Loans of Nigerian Deposit Money Banks. Journal of Business and Management, **2 (3):** 12-21

O'connor, M and Rafferty, M (2012). Corporate Governance and Innovation. *Journal of financial and quantitative analysis* **47(2):** 397–413

Obamuyi, T.M. (2013). Determinants of banks' profitability in a developing economy: evidence from Nigeria. Online ISSN 2345-0037. *Organisations and markets in emerging economies* **4(2(8):** 97-111

OECD (2004). Corporate Governance: A survey of OECD countries.

OECD 2009. 'Corporate governance and the financial crisis: key findings and main messages',
June, available at http://www.oecd.org/corporate/ca/corporategovernanceprinciples/43056196.pdf Accessed 31 October, 2016.

OECD (2014). 2014 Review of OECD Principles of Corporate Governance. Retrieved from: https://www.oecd.org/daf/ca/WBCSD2015CGP.pdf Accessed 3 September, 2016.

OECD (2015). G20/OECD Principles of Corporate Governance - OECD.org - OECD. Retrieved from: www.oecd.org/daf/ca/Corporate-Governance-Principles-ENG.pdf Accessed on 22 December, 2016.

Official Journal European Union (2005). Retrieved from: http://eur-lex.europa.eu/LexUriServ/LexUriServ.do?uri=OJ:L:2005:052:0051:0063:EN:PDF. Accessed on 15 December, 2016

Ofo, N. (2016). Corporate Governance as springboard of socio-economic renaissance. ICSAN annual conference held at the Sheraton Lagos Hotel, Ikeja Lagos on Thursday November 17, 2016.

Ogbechie, C and Koufopoulos, D.N. (2010). Corporate governance and board practices in the Nigerian banking industry.

Ogechukwu, A.D. (2013). The Current Ethical Challenges in the Nigerian Commercial Banking Sector. Global Journal of Management and Business Research **13 (10):**35-50

Ojeka, S.A., Iyoha, F.O, and Ikpefan, O.A. (2014). CEOs Removal and Bailed Out Banks in Nigeria: Does Absence of Good Corporate Governance Practices Responsible? *Euro-Asian Journal of Economics and Finance* **2 (4):** 418-430

Okike, E. N. M., Adegbite, E. A., Nakpodia, F. A., and Adegbite, S. (2015). A review of internal and external influences on corporate governance and financial accountability in Nigeria. International *Journal of Business Governance and Ethics*, **10(2):** 165-185.

Olaoti, Y.I. (2016). Board of directors' heterogeneity and financial performance of listed deposit money banks in Nigeria. Thesis, Ahmadu Bello University Zaria.

Oliver, C. (1992). The antecedents of deinstitutionalization. Organisation Studies, **13**(*4*): 563-588.

Oluwagbemi, O., Abah, J., and Achimugu, P. (2011). The Impact of Information Technology in Nigeria's Banking Industry. Journal of computer science and engineering, **7(2):**63-67.

Oman, C., S. Fries; and Buiter, W. (2003). "Corporate Governance in Developing, Transition and Emerging-Market Economies." Policy Brief No. 23. OECD Development Centre, Le Seine Saint-German, France.

Ong, T. and Djajadikerta, H.G. (2016). Impact of corporate governance on sustainability reporting: Empirical study in the Australian resources industry. :1-20

Onuoha, B. C., Ogbuji, C. N., Ameh, A. A., and Oba, U. O. (2013). Strategies for improving corporate governance by organisations in Nigeria. *International Business and Management* **7(2):** 26-31.

Oserogho, O.E. (2003). Corporate Governance in Nigeria. Are there laws and principles? Retrieved from: http://www.oseroghoassociates.com/articles/126-corporate-governance-in-nigeria-are-there-laws-principles. Accessed on 11 November, 2016.

Otman, K.A.M. (2014). Corporate Governance and Firm Performance in Listed Companies in the United Arab Emirates. Thesis submitted in fulfilment of the requirements of the degree of Doctor of Philosophy, College of Business Victoria University of Melbourne Australia

Oyewunmi, O. A., Olusanmi, O., Olujobi, O., and Adegboye, F. (2017). Corporate regulation of unethical practices: Assessment of Nigeria's commercial banking industry. International Journal of Economic and

Financial Issues, **7(2):** 551-555.Ozili, P.K., and Uadiale, O. (2017). Ownership concentration and bank profitability. *Science Direct Future Business Journal* **3:** 159–171

Pasiouras, F., (2008), International Evidence on the Impact of Regulations and Supervision on Banks' Technical Efficiency: An Application of two-stage Data Envelopment Analysis", *Review of Quantitative Finance and Accounting* **30 (2):** 187-223.

Pasiouras, F., Tanna, S. and Zopounidis, C. (2009), "The Impact of Banking Regulations on Banks' Cost and Profit Efficiency: Cross-Country Evidence", *International Review of Financial Analysis* **(18):** 294-302.

Pass, C. (2004). "Corporate Governance and the Role of Non-Executive Directors in Large UK Companies: An Empirical Study". *Corporate Governance* **4(2):** 52-63.

Pathan, S. and Skully, M. (2010). Endogenously structured boards of directors in banks. *Journal of Banking and Finance* **34(7)**: 1590–1606.

Pathan, S. (2009) Strong boards, CEO power and bank risk-taking. *Journal of Banking and Finance* **33(7):** 1340–1350.

Peni, E. (2014). CEO and Chairperson Characteristics and firm performance. *J Manag Gov* **18:**185–205

Penman, S., and Penman, S. (2007). Financial statement analysis and security vauation (:476). New York: McGraw-Hill.

Perihan, I and Kienpin, T (2017). Boardroom Diversity, Corporate Governance and Innovation in the UAE Banks. Zayed University, Working Paper No. ZU-WP 2017-04-02. Pg 1-22

Persico, N. (2004) Committee design with endogenous information. *Review of Economic Studies* **71(1):** 165–194.

Pinto, F. and Sobreira,R. (2010). Financial innovations, Crises and regulation: Some assessments. Journal of Innovation Economics **2(6):**9-23

Podsakoff, N. P., Whiting, S. W., Podsakoff, P. M., and Blume, B. D. (2009). Individual- and organizational-level consequences of organizational citizenship behaviors: A meta-analysis. *Journal of Applied Psychology*, **94(1):** 122–141.

Porter, M. E., and M. R. Kramer (2006). Strategy and Society: The Link between Competitive Advantage and Corporate Social Responsibility, *Harvard Business Review* **84(12):** 78-92.

Racic, D., Cvijanovic, V. and Aralica, Z. (2007). The Effects of the Corporate Governance System on Innovation Activities in Croatia

Rahman, A.N.A, and Reja, B.A.F (2015). Ownership structure and bank performance. Journal of Economics, Business and Management, **3 (5):** 483-488

Randoy, T., Oxelheim, L., and Thomsen, S. (2006). A Nordic perspective on corporate board diversity. Retrieved from: http://www.nordicinnovation.org/Global/_Publications/Reports/2006/The%20performance%20effects%20of%20board%20diversity%20in%20Nordic%20Firms.pdf

Rebai, S, Azaiez, M.N., and Saidane, D. (2012) Sustainable performance evaluation of banks using a multi-attribute utility model: an application to French banks. *Procedia Economics and Finance* **2:**363 – 372

Rebai-Bouricha, M.S. (2014). New Banking Performance Evaluation Approach: Sustainable Finance and Sustainable Banking Based. Doctorate Thesis Universite De Tunis

Renee, B.A. (2010) Introduction to chapter 23of corporate governance: governance of banking institutions. Extracts from corporate governance edited by
H. Kent Baker and Ronald Anderson. Copyright (c) 2010 by John Wiley and Sons, Inc. Electronic copy available at: http://ssrn.com/abstract=1541670

Retzer, K.F. (2003). Introduction to survey sampling. Sociology 381 –Survey research, University of Illinois at Urbana –Champaign.

Richard, O.C., Barnett, T., Dwyer, S. and Chadwick, K. (2004). Cultural diversity in management, firm performance and the moderating role of entrepreneurial orientation dimensions. *Academy of Management Journal* **47(2):** 255-266

Richard, P. J., Devinney, T. M., Yip, G. S., and Johnson, G. (2009). Measuring organisational performance: towards methodological best practice. *Journal of Management*, **35(3):** 718-804.

Richardson, B. (2009). Climate finance and its governance: moving to a low carbon economy through socially responsible financing? *International and Comparative Law Quarterly* **58:** 597–626.

Rime, B. (2001). Capital Requirements and Bank Behaviour: Empirical Evidence for Switzerland, *Journal of Banking and Finance* **25:** 789-805

Roberts, P. W., and Dowling, G. R. (2002). Corporate reputation and sustained superior financial performance. *Strategic Management Journal* **23:** 1077-1093.

Robins, N, and Krosinsky C. (2008). After the credit crunch: the future of sustainable investing. *Public Policy Research* **15(4):** 192–197.

Robson, C. (2002) Real World Research (2nd edn). Oxford: Blackwell.

Roca, Laurence Clément - Searcy, Cory (2012), "An Analysis of Indicators Disclosed in Corporate Sustainability Reports", Journal of Cleaner Production Volume 20, Issue 1, p. 103-118.

Rogosic, A. (2014). Corporate social responsibility reporting of the banks in Bosnia and Herzegovina, Croatia and Montenegro. *Theoretical and Applied Economics* **21(9):** 71-82

Ross, S. (1973). The economic theory of agency: the principal problem, American Economic Review, **63(2):** 134–139.

Roy, M.K., Sarker, A, and Parvez, S. (2015). Sustainability in Banking Industry: Which way to move? *ASA University Review* **9(2)**

Rudden, R and Rudden, K. (2012). The Importance of Being Sustainable. Retrieved from: https://www.fortnightly.com/fortnightly/2012/06/importance-being-sustainable?page=0%2C1

Rupley, K., Brown, D. and Marshall, R. (2012). Governance, media and the quality of environmental disclosure. Journal of Accounting and Public Policy, **31(6):** 610-640

Sanda, A, Mikailu, A.S., and Garba, T. (2005). Corporate governance mechanisms and firm financial performance in Nigeria. AERC Research Paper 149 African Economic Research Consortium, Nairobi March 2005:1-41.

Sanda, A.U, Garba, T, and Mikailu, A.S. (2008). Board Independence and Firm Financial Performance: Evidence from Nigeria. A Paper Submitted to the Centre for the Study of African Economies (CSAE) for presentation at the CSAE Conference 2008 titled Economic Development in Africa at St Catherine's College, University of Oxford, Oxford, 16-18 March 2008

Santos, J.B. and Brito, L.A.L. (2012). Towards a subjective measurement model. *BAR, Rio de Janeiro*, **9 (6):** 95-117.

Sanusi, L.S (2012). Banking reform and its impact on the Nigerian economy. Being a lecture delivered at the University of Warwick's Economic Summit, UK 17th February, 2012

Sarre, R. (2003) Corporate governance in the wake of contemporary corporate collapses: some agenda items for evaluators, *Evaluation Journal of Australasia*, **3:** 48–55.

Saunders, M., Lewis, P. and Thornhill, A. (2009). Research Methods for Business Students. London: Pitman Publishing.

Scherer, A., Palazzo, G. and Baumann, D. (2006). 'Global Rules and Private Actors, Toward a New Role of the TNC in Global Governance', *Business Ethics Quarterly* **16**: 502–532.

Schaltegger, S. and Ludeke-Freund, F. (2012). The "Business Case for Sustainability" Concept. A Short Introduction. Centre for Sustainability Management (CSM) Leuphana Universität Lüneburg Scharnhorststr. 1D-21335 Lüneburg. :1-12

Schneider, J., and Chan, S.Y. (2000). A comparison of corporate governance systems in four countries, School of Business, Business Research Centre, Hong Kong Baptist University.

Schnyder, G. (2012). Measuring corporate governance: lessons from the 'bundles approach'. Centre for Business Research, University of Cambridge Working Paper No. **438** :1-37

Scholtens, B., and Zhou, Y. (2008). Stakeholder relations and financial performance. *Sustainable Development* **16(3):** 213–232. DOI: 10.1002/sd.364

Schubert, R. (2006). Analyzing and managing risks—on the importance of gender difference in risk attitudes. *Managerial Finance*, **32(9):** 706–715.

Scott, W. (1994) 'Conceptualising organisational fields: Linking organisations and societal systems', in Derlien, H., Gerhardt, U., and Scharpf, F. (eds.) Systemrationalitat und Partialinteresse [Systems rationality and partial interests]. Baden: Nomos Verlagsgesellschaft, : 203-221.

Scott, W. R. (2005). Institutional theory: contributing to a theoretical research program. In K. G. Smith and M. A. Hitt (Eds.), Great minds in management: the process of theory development (:460-484). Oxford: Oxford University Press.

Sebhatu, S.P. (2008). Sustainability Performance Measurement for sustainable organisations: beyond compliance and reporting. 11th QMOD Conference. Quality Management and Organisational Development Attaining Sustainability from Organisational Excellence to Sustainable Excellence; 20-22 August; 2008 in Helsingborg; Sweden :75-87

Shadab, H.B. (n.d.). Innovation and corporate governance: The impact of Sarbanes-Oxley. U. PA. *Journal of business and employment law* **10(4):** 955-1008.

Shah, S., Butt, S., and Saeed, M. (2011). Ownership structure and performance of firms: Empirical evidence from an emerging market. *African Journal of Business Management*, **5 (2):** 515-523.

Shah, S., Javed, T. and Abbas M. (2009). Determinants of CEO Compensation Empirical Evidence from Pakistani Listed Companies. *International Research Journal of Finance and Economics* retrieved from http://www.eurojournals.com/finance.htm

Shehzad, C.T., de Haan, J. and Scholtens, B. (2010) The impact of bank ownership concentration on impaired loans and capital adequacy. *Journal of Banking and Finance* **34:** 399–408.

Shivdasani, A. (1993). "Board Composition, Ownership Structure and Hostile Takeover". *Journal of Accounting and Finance*, **16:** 167-198.

Shleifer, A. and Vishny, R.W. (1997). A survey of corporate governance, *The Journal of Finance*, **52(2):** 737-783.

Shleifer, A., and R.W. Vishny, (1989), "Management entrenchment: The case of manager-specific investments. *Journal of Financial Economics* **25:** 123-139. North-Holland

Sigler, K.J. (2011). CEO Compensation and Company Performance. *Business and Economics Journal, BEJ* - **31:**1-8.

Simon, M.K., and Goes, J. (2013). Dissertation and Scholarly Research: Recipes for success. Seattle, WA: Dissertation Success LLC

Slater, S. F., and Olson, E. M. (2000). Strategy type and performance: the influence of sales force management. *Strategic Management Journal*, **21(8):** 813-829.

Smith, A. (1776). An Inquiry into the Nature and Causes of the Wealth of Nations. Edwin Cannan, ed. 1904. Library of Economics and Liberty. Retrieved January 24, 2017 from the World Wide Web: http://www.econlib.org/library/Smith/smWN.html

Smith, N., Smith, V. and Verner, M. (2006). Do women in top management affect firm performance? A panel study of 2,500 Danish firms. *International Journal of Productivity and Performance Management*, **55(5):** 569–593.

Solomon, J. (2013). Corporate governance and accountability (4th ed.), Chichester: John Wiley and Sons.

Soludo C. (2004). Consolidating the Nigerian Banking Industry to meet the development Challenges of the 21st century. Being an address delivered to the special meeting of The Bankers Committee, held on July 6, 2004 at the CBN Head Quarter, Abuja.

Soludo, C. (2006). Beyond banking sector consolidation in Nigeria. Paper presented at the 12th Annual Nigerian Economic Summit, Transcorp Hilton, Abuja.

Stankevicienea, J., and Nikonorova, M. (2013) Sustainable Value Creation in Commercial Banks during Financial Crisis. *Procedia - Social and Behavioral Sciences* **110:** 1197 – 1208

Stephenson, C. (2004). "Leveraging Diversity to Maximum Advantage: The Business Case for Appointing More Women to Boards", *Ivey Business Journal* **69(1):** 1-5.

Sternberg, E. (1998). Corporate Governance: Accountability in the Marketplace, Institute of Economic Affairs, London.

Stewart, L.A. (2010). The Impact of Regulation on Innovation in the United States: A Cross-Industry Literature Review. A paper commissioned by the Institute of Medicine Committee on Patient Safety and Health IT. Retrieved from: www.iom.edu/hitsafety.

Stiglitz J.E. (2010) Financial innovation, The Economist debates, http://www.economist.com/debate/days/view/471

Streeck, W. and Yamamura, K. (2001) The Origins of Nonliberal Capitalism: Germany and Japan, Ithaca, NY and London: Cornell University Press.

Stubbs, W., and Cocklin, C., (2008). Conceptualizing a "sustainability business model". *Organization and Environment* **21**(2): 103-127.

Stuebs, M and Sun, L. (2015) Corporate governance and social responsibility. *International Journal of Law and Management* **57(1):** 38-52.

Subrahmanyam, V., N. Rangan and S. Rosenstein (1997). The Role of Outside Directors in Bank Acquisitions. Financial Management, Autumn, :23-36.

Suddaby, R. (2013). Institutional theory. In Encyclopedia of Management Theory. Sage Publications, Inc. **1**:379-383

Sun, J. and Liu, G. (2014). Audit committees' oversight of bank risk-taking, *Journal of Banking and Finance* **40:** 376–387.

Sun, W, Stewart, J., and Pollard, D. (2012). A Systemic Failure of Corporate Governance: Lessons from the On-going Financial Crisis. Retrieved from: http://www.europeanfinancialreview.com/?p=2042 Accessed 31 October, 2016

Sustentare-SAM (2010). Sustainability governance –Portuguese companies in an international context. Retrieved from http://www.sustentare.pt/pdf/doc.suste+sam(ENG1).pdf Accessed on 15 November, 2016.

Talavera, Yin and Zhang (2018). Age diversity, directors' personal values, and bank performance. International Review of Financial Analysis **55 :**60–79

Tarraf, H. (2011). "The Role of Corporate Governance in the Events Leading up to the Global Financial Crisis: Analysis of Aggressive Risk-Taking", *Global Journal of Business Research,* **5 (4):** 93-105.

Tashakkori, A., and Teddlie, C. (2003). Handbook of Mixed Methods in Social and Behavioral Research. Thousand Oaks: Sage.

Teng, L. L., Aun, L. K., and Fook, O. S. (2011). Corporate governance assessment in company board structure. *African Journal Business Management,* **4:** 1175-1183. doi:10.5897/AJM10.535

The Vanguard Newspaper (2018). Shareholders threaten court action over CBN's policy on bad loans, dividends. Retrieved from: https://www.vanguardngr.com/2018/02/breaking-shareholders-threaten-court-action-cbns-policy-bad-loans-dividends/

Tihanyi, L., Graffin, S. and George, G. (2015). Rethinking governance in management research. *Academy of Management Journal* **1015(1):** 1–9.

Tilakasiri, K.K (2013) Construct an Index for Measuring Corporate Social Responsibility Activities for Developing World: Evidence from Sri Lanka. International Journal of Science and Research (IJSR) ISSN (Online): 2319-7064 Index Copernicus Value (2013): 6.14

Trevino, L. K., and Brown, M. E. (2005). Managing to be ethical: Debunking five business ethics myths. Strategic human resource management: theory and practice.

Trevino, L.K., Brown, M., and Hartman, L.P. (2003). A Qualitative Investigation of Perceived Executive Ethical Leadership: Perceptions from Inside and Outside the Executive Suite. *Sage journals pub* **56(1):** 5-37 https://doi.org/10.1177/0018726703056001448

Trichet J.C. (2009) The financial crisis and the ECB's response so far, Keynote address by Mr Jean-Claude Trichet, President of the European Central Bank, at the Chatham House Global Financial Forum, New York, 27 April, BIS Review 51/2009

Tricker, B. (2015). Corporate governance: principles, policies and practices (3rd ed.), Oxford: Oxford University Press.

Tsegba, I., Herbert, W., and Ene, E. (2014). Corporate Ownership, Corporate Control and Corporate Performance in Sub-Saharan African: Evidence from Nigeria. *International Business Research*, 73.

Turnbull, S. (1997) Corporate Governance: Its scope, concerns and theories. *Corporate Governance: An International Review* **5:**180–205.

Udayasankar, K. and Das, S.S. (2007) Corporate Governance and Firm Performance: the effects of regulation and competitiveness, *Corporate Governance: An International Review* **15:** 262–271

Udueni, H. (1998). Power Dimension in the Board and Outside Director Independence: Evidence from Large Industrial UK Firms". Discussion Paper, School of Management and Finance, University of Nottingham, June.

Ugwuanyi, G.O. (2015). Regulation of Bank Capital Requirements and Bank Risk-Taking Behaviour: Evidence from the Nigerian Banking Industry. *International Journal of Economics and Finance*; **7(8)** 2015 ISSN 1916-971XE-ISSN 1916-9728

Ugwuanyi, G.O. (2014). Banking distress and the erosion of public confidence in the Nigerian banking system. *European International Journal of Business and Management* **6(9):**118-126 ISSN 2222-1905 (Paper) ISSN 2222-2839 (Online)

Ujunwa, A. (2012). Board characteristics and the financial performance of Nigerian quoted firms. *Corporate Governance* **12 (5):** 656-674

Ungureanu, M. (2013). Models and practices of corporate governance worldwide - CES Working Papers: 625

Ungureanu, M-C. (2008). Banks: Regulation and Corporate Governance Framework. *Journal of Ownership and Control.* **5(2).** Electronic copy available at: http://ssrn.com/abstract=1084042

United Nations Global Compact -UNGC (2015) SDG Industry Matrix; Financial Services. Available at: https://www.unglobalcompact.org/doc s/issues_doc/development/SDGMatrix_FinancialSvcs.pdf

Upadhyay, A. and Sriram, R. (2011). Board size, corporate information environment and cost of capital. *Journal of Business Finance and Accounting* **38(9, 10):** 1238–1261.

Uwuigbe, O.R. and Fakile, A.S. (2012). The effects of board size on financial performance of banks: A study of listed banks in Nigeria. *International Journal of Economics and Finance*. **4(2).**

Uwuigbe, O.R. (2011). Corporate governance and financial performance of banks: a study of listed banks in Nigeria. Doctoral thesis, department of accounting, Covenant University, Ota.

Vafeas, N., Waegelein, J.F. and Papamichael, M. (2003). The Response of Commercial banks to Compensation Reform. *Review of Quantitative Finance and Accounting*, **20 (4):** 335-354.

Vargas, A.R. (2009). Assessing the contribution of financial innovations to the production of implicit services of financial intermediation in Costa Rica. **31:** 445-466

Varshney, P., Kaul, V., and Vasal, V. (2012). Corporate Governance Mechanisms and Firm Performance: A Study of Select Indian Firms.

Villiers, C., Naiker, V. and Staden, C.J. (2011). The Effect of Board Characteristics on Firm Environmental Performance. *Journal of Management* **37(6):** 1636-1663

Vinten, G. (2002). The corporate governance lessons of Enron. *Corporate Governance* **2(4–9)**.

Vitols, S and Kluge, N. (2011). The Sustainable Company: a new approach to corporate governance. Brussels: ETUI aisbl, Brussels.

Waddock, S. A., and Graves, S.B. (1997). The Corporate Social Performance–Financial Performance Link, *Strategic Management Journal* **18:** 303-319.

Waddock, S.A. and Graves, S.B. (1994) "Industry Performance and Investment in RandD and Capital Goods". *Journal of High Technology Management Research*, **5 (1):** 1-17

Wahl, M. (n.d.). The ownership structure of corporations: owners' classification and typology. *EBS Review: Special issue on Corporate Governance*. :1-14.

Walker, K. and Dyck, B. (2014). The Primary Importance of Corporate Social Responsibility and Ethicality in Corporate Reputation: An Empirical Study. *Journal of the center for business review- Business and Society Review* **119(1):** 147–174.

Walker, S.D. (2009). A review of corporate governance in UK banks and other financial industry entities, Final recommendations. http://www.hm-treasury.gov.uk/d/walker_review_261109.pdf. (Accessed on October 15, 2016).

Walls, J.L, Phan, P.H, and Berrone, P. (2011). Measuring environmental strategy: construct development, reliability and validity. *Business and Society* **50(1):** 71–115.

Walls, J.L., Berrone, P. and Phan, P.H. (2012). Corporate governance and environmental performance: is there really a link? *Strategic Management Journal*, **33:** 885–913

Wang, I., and Dewhirst, H. D. (1992). Boards of directors and stakeholder orientation. *Journal of Business Ethics.* **11:** 115-123

Ward, A. J., Brown, J. A., and Rodriguez, D. (2009). Governance Bundles, Firm Performance, and the Substitutability and Complementarity of Governance Mechanisms. Corporate Governance: An International Review, **17(5):** 646-660.

Warren-Myers, G. (2013). Is the valuer the barrier to identifying the value of sustainability? Journal of Property Investment and Finance, 31(4), 345-359. http://dx.doi.org/10.1108/JPIF-01-2013-0004

Warren, J., and Thomsen, M. (2012). The Case for Corporate Responsibility Reporting: Valuing and Communicating the Intangibles. White Paper. VT: One Report, Inc. Alternative citation: Retrieved from: https://one-report.com/wp-content/uploads/2017/08/white_paper_why_report_08_10_17_final.pdf

Watson, T. (2007). Reputation and ethical behaviour in a crisis: predicting survival, Journal of Communication Management **11(4):** 371-384

Wartick, S. (2002). Measuring corporate reputation: Definition and data, Business and Society **41(4):** 371–392.

WBCSD (2003). Sustainable development reporting: Striking the balance. Retrieved from: http://wbcsdservers.org/wbcsdpublications/cd_files/datas/financial_capital/reporting_investment/pdf/SustainableDevReporting-Striking-the-balance.pdf Accessed on 24 January, 2017

Webb, E.J., Campbell, D. T., Schwartz, R. D. and Sechrest, L. (1966). Unobtrusive methods in social research. Open University Press Buckingham · Philadelphia

Weber, O, Diaz, M and Schwegler, R. (2014). Corporate Social Responsibility of the Financial Sector – Strengths, Weaknesses and the Impact on Sustainable Development. *Sustainable Development Sust. Dev.* **22:** 321–335

Weber, O., and Oni, O. (2015). The impact of financial sector sustainability regulations on banks. CIGI Papers **77**: 1-13.

Weber Shandwick and KRC research (2015). The company behind the brand: in reputation we trust – CEO spotlight. Retrieved from: http://webershandwick.co.uk/wp-content/uploads/2015/02/CEO-FULL-REPORT-FINAL-Digital-ML.pdf

Wepukhulu, J.M., (2016). Relationship between corporate governance and performance of commercial banks in Kenya. A thesis submitted for the degree of Doctor of Philosophy in Business Administration in Jomo Kenyatta University of Agriculture and Technology

Westman, H. (2011). The impact of management and board ownership on profitability in banks with different strategies. *Journal of Banking and Finance* **35:** 3300–3318.

Westphal, J.D. and Bednar, M.K. (2005) Pluralistic ignorance in corporate boards and firms' strategic persistence in response to low firm performance. *Administrative Science Quarterly* **50(2):** 262–298.

White, J. (2006). Family business, the future…Running your business, September :46-47. Retrieved from: http://www.jbw.ie/the_accountant/RYB_Sep_06_Finance.pdf

Wiederman, K. P., and Buxel, H. (2005). Corporate reputation management in Germany: Results of an empirical study. *Corporate Reputation Review* **8 (2):** 145–163.

Wiklund, J., and Shepherd, D. (2003). Knowledge-based resources, entrepreneurial orientation, and the performance of small and medium-sized businesses. *Strategic Management Journal,* **24(13):** 1307-1314.

Wilcox, J.C. (1997). A 10-Year Quest for Director Accountability. *Directors and Boards*, **22:** 46-50.

Williams, R.J. (2003). Women on Corporate Boards of Directors and Their Influence on Corporate Philanthropy *Journal of Business Ethics* **42(1):**1-10 OI10.1023/A:1021626024014

Williamson, O.E. (1985). The Economic Institution of Capitalism, The Free Press, New York.

Williamson, O.E. (1975). Markets and Hierarchies: Analysis and Antitrust Implications, The Free Press, New York.

Wilson, M. (2003). Corporate sustainability: what is it and where does it come from?. Retrieved from: https://iveybusinessjournal.com/publication/corporate-sustainability-what-is-it-and-where-does-it-come-from/

Woo, K.L. (2017). How Chinese commercial banks innovate: process and Practice. *Journal of Innovation Management* **5(2):** 81-110

Wong, H. and Wong, R (2015). Corporate Social Responsibility Practices in Banking Industry. Journal of Management Research **7(4):**205-221

Wood, D. J. (1991). Corporate social performance revisited. *Academy of Management Review* **16***(4):*691-718

Wu, M. W. and Shen, C. H. (2013) Corporate social responsibility in the banking industry: Motives and financial performance, *Journal of Banking and Finance* **37:** 3529–3547.

Wu, S. and Cui, H. (2002) Consequences of the concentrated ownership structure in Mainland China — evidence of Year 2000, working paper, City University of Hong Kong, Hong Kong.

Xi'an, J.W., Xi'an, G.T., Fan, W, and Luo, D (2017). The Effect of Mandatory Regulation on Corporate Social Responsibility Reporting Quality: Evidence from China. *The Journal of Applied Business Research* –**33(1)**

Yakasai, G.A. (2001). Corporate governance in a third world country with particular reference to Nigeria. *Corporate Governance: An International Review* **9(3):** 239 –240

Yamak, S., and Süer, Ö. (2005) "State as a stakeholder", Corporate Governance: *The international journal of business in society* **5(2):** 111 – 120

Yeoh, P. (2009). Sustainable banking: the commercial and legal impacts of private self-regulatory initiatives. *European Energy and Environmental Law Review* **18(6):** 274–288.

Yermack, D. (1996). Higher market valuation of companies with a small board of directors. *Journal of Financial Economics* **40:** 185-211.

Yidawi, A. (2005). A survey of ethics in the Nigerian banking industry. Being a dissertation submitted in partial fulfilment of the requirements for the award of the Doctor of Philosophy (PhD) Management of St. Clements University

Yin, R. K. (2011). Qualitative research from start to finish (3rd ed.). Thousand Oaks, CA: Sage.

Yin, R. K. (2014). Case study research design and methods (5th ed.). Thousand Oaks, CA: Sage.

Yount, W.R. (2006). Research Design and Statistical Analysis for Christian Ministry. 4th Edition. Fort Worth, Texas: Southwest Baptist Theological Seminary.

Zalewska, A. (2016). A new look at regulating bankers' remuneration. *Corporate Governance: An International Review*.

Zattoni, A. Douglas, T. and Judge, W. (2013). 'Developing Corporate Governance Theory through Qualitative Research', *Corporate Governance: An International Review*, **21(2):**119-122.

Zeitun, R. and Gary, G.T. (2007) Does ownership affect a firm's performance and default risk in Jordan? *Corporate Governance* **7:** 66–82.

Zhao, T and Nurinde, V. (2011). Bank Deregulation and Performance in Nigeria. *African Development Review* **23(1):** 30–43

Zhu, Y., Sun, LY. and Leung, A.S.M. Asia Pac J Manag (2014) 31: 925. https://doi.org/10.1007/s10490-013-9369-1 :272: A good way of constructing recommendation

Zhu, Y., Sun, L-Y., and Leung, A.S.M. (2013). Corporate social responsibility, firm reputation, and firm performance: The role of ethical leadership *Asia Pacific Journal of Management* **31(4):**925-947 DOI10.1007/s10490-013-9369-1

Zubaidah, Z., Nurmala, M., and Kamaruuzaman, J. (2009). Board structure and corporate performance in Malaysia. *International Journal of Economic and Finance* **1 (1):** 150 164.

# 8.0 Appendices

## Appendix "A": Questionnaire - Survey on Corporate Governance Dimensions and Sustainability of Deposit Money Banks in Nigeria

Department of Business Management,
College of Business and Social Sciences,
Covenant University,
Canaan Land, Ota
Ogun State.

**Appendix:**

Dear Respondent,

I am conducting a doctoral research on "**Corporate Governance Dimensions and Sustainability of Banks in Nigeria**", and I have the pleasure of inviting you to fill this questionnaire as you have been selected to participate in the study. Your co-operation in participating in this study will be highly appreciated.

Please note that the results of the survey will be used only in aggregated form and therefore the confidentiality of your responses is assured. Insights from this study will be beneficial to several groups; of interest are academics and researchers, managers of banking firms, policy makers, regulatory boards and the society in general.

The questionnaire which will take approximately 15-20 minutes has been structured in a very simple and convenient pattern.

Thank you and God bless.

Onakoya, Olorunfemi Adebisi

**SECTION A: DEMOGRAPHIC DATA (Please tick √ whichever is applicable)**

1. **Gender:** Male ( ) Female ( )
2. **Age Grade**: 18-24 ( ) 25-34 ( ) 35-44 ( ) 45-54 ( ) Above 54 ( )
3. **Marital Status**: Single ( ) Married ( ) Divorced ( ) Separated ( )
4. **Highest Education Degree**: Bachelors ( ) Masters ( ) Doctorate ( ) Others Pls. specify……….
5. **Job Function**: Business Development ( ) Operations and Technology ( ) Risk Management ( ) Corporate Development ( ) Others Pls. specify ……..................
6. **Job Position**: Trainee ( ) Banking Officer ( ) Management ( )Senior Management ( )

   Executive Management ( )
7. **Years Spent in the Bank**: Below 3yrs ( ) 3-5yrs ( ) 6-10yrs ( ) Above 10yrs ( )
8. **Bank's Name:** ………………………………………………..

**SECTION B: STUDY SPECIFIC DATA**

Please tick √ the appropriate answer from the alternatives given as they best describe your opinion:

**Strongly Agree (SA)**  **Agree (A)**  **Neutral (N)**  **Disagree (D)**  **Strongly Disagree (SD)**

| | | | | | | |
|---|---|---|---|---|---|---|
| **A** | **CORPORATE SOCIAL PERFORMANCE** | | | | | |
| **A1** | **Social Performance** | | | | | |
| **A1i** | **Employees Relationship: In my bank ….** | **SA** | **A** | **N** | **D** | **SD** |
| 1 | Training and development is highly valued and promoted | | | | | |
| 2 | Employees are remunerated and rewarded fairly | | | | | |
| 3 | Staff turnover is minimised | | | | | |
| 4 | Employees have a sense of job satisfaction | | | | | |
| 5 | Career and advancement path for employees is clear and fair | | | | | |
| **A1ii** | **Customers Relationship** | **SA** | **A** | **N** | **D** | **SD** |
| 1 | My bank promotes consumer rights protection | | | | | |
| 2 | Our service delivery is timely | | | | | |
| 3 | My bank provides a conducive environment for customers' transactions | | | | | |
| 4 | My bank continuously introduces innovative products and services to meet customers' expectations | | | | | |
| 5 | Our customers do word-of-mouth marketing on our behalf | | | | | |
| **A1iii** | **Community and Society Relationship** | **SA** | **A** | **N** | **D** | **SD** |
| 1 | My bank supports philanthropic activities financially on a regular basis | | | | | |
| 2 | My bank's policy encourages staff to engage in community service | | | | | |
| 3 | My bank participates in local or state meetings to discuss issues affecting its host community | | | | | |
| 4 | My bank executes initiatives for the marginalised groups in the society regularly | | | | | |
| 5 | My bank has a policy statement on bribery and corruption | | | | | |
| **A2** | **Environmental Performance: My bank …** | **SA** | **A** | **N** | **D** | **SD** |
| 1 | continuously introduces initiatives to reduce resource consumption (water, energy, paper) | | | | | |
| 2 | carries out environmental impact assessment on each credit application | | | | | |
| 3 | actively supports customers who want to develop environmentally-friendly business activities | | | | | |
| 4 | promotes employees' awareness and training on environmental issues | | | | | |
| 5 | invests in pollution prevention (e.g. reduction in emissions to air and water, smokes, fumes and noise). | | | | | |
| 6 | has initiatives in place to manage waste, water discharges, and spills | | | | | |
| 7 | embraces new environmental technologies and processes | | | | | |
| **B** | **REGULATION** | **SA** | **A** | **N** | **D** | **SD** |
| 1 | Regulators from CBN/NDIC demonstrate strong ethical values during their inspections and examinations exercise. | | | | | |
| 2 | Disclosure of bank's performance as published in the annual report adequately provides stakeholders with transparent information for their decision-making. | | | | | |

| | | | | | | |
|---|---|---|---|---|---|---|
| 3 | Banks' restrictions from non-core activities (such as real estate, insurance and securities) limits their benefits of economies of scale. | | | | | |
| 4 | Mandatory minimum capital requirement helps banks to improve their operational efficiency. | | | | | |
| 5 | Mandatory minimum capital requirement intensifies banks' risk appetite. | | | | | |
| 6 | The Deposit Insurance Scheme of NDIC to protect depositors' funds has minimal impact on banks' risk-taking. | | | | | |
| 7 | The supervisory and regulatory power of CBN to discipline bank management, owners, and auditors is vital to the banking system. | | | | | |
| 8 | Entry restrictions in licensing of prospective banks is essential to promote healthy competition in the industry | | | | | |
| 9 | CBN's supervisory and regulatory activities impact the pricing of banks' products to the benefit of customers. | | | | | |
| 10 | Supervisors and examiners from CBN/NDIC demonstrate high-quality skills during inspections and examinations. | | | | | |
| 11 | CBN's regulations on Capital Reserve requirement, liquidity, and non-performing loans help to mitigate against bank failures. | | | | | |
| **C** | **FINANCIAL INNOVATION** | **SA** | **A** | **N** | **D** | **SD** |
| 1 | My bank develops innovative products and services | | | | | |
| 2 | My bank outperforms peers in Information Communication Technology (ICT) | | | | | |
| 3 | My bank regularly improves its operating systems and processes | | | | | |
| 4 | My bank regularly updates its delivery channels | | | | | |
| **D** | **INTERNAL GOVERNANCE CONTROLS** | **SA** | **A** | **N** | **D** | **SD** |
| **D1** | **Board Structure** | | | | | |
| 1 | The Board size is adequate to positively influence the bank's performance. | | | | | |
| 2 | Having more Non-Executive Directors acts as a check and balance on management | | | | | |
| 3 | Board independence facilitates objective decision-making process | | | | | |
| 4 | The more Board meetings held annually, the more board oversight on business is achieved | | | | | |
| 5 | Board committees have strong effect in shaping the bank's performance | | | | | |
| 6 | A diverse Board (in gender, age, ethnicity) enriches its decision-making quality | | | | | |
| 7 | The Board plays a key role in setting the bank's strategy. | | | | | |
| **D2** | **Management Structure** | | | | | |
| 1 | Promoting an employee to CEO internally boosts bank's performance | | | | | |
| 2 | A longer tenure in office by the CEO increases the likelihood of improved bank performance | | | | | |
| 3 | Performance-based remuneration for the CEO enhances the drive for improved bank's performance. | | | | | |
| 4 | CEO's diversity (age, gender, ethnicity) has significant impact on performance | | | | | |
| 5 | The CEO's reputation in the society has impact on performance | | | | | |
| 6 | Incentive-based compensation for the CEO drives financial performance focus | | | | | |

| D3 | Ownership Structure | | | | | |
|---|---|---|---|---|---|---|
| 1 | Institutional investors provide effective monitoring on the Board | | | | | |
| 2 | Directors' share ownership is most helpful to improve stakeholders' interests | | | | | |
| 3 | Diffused ownership (i.e. many with few shares) encourages little or no monitoring of management | | | | | |
| 4 | Concentrated ownership (i.e. few with many shares) encourages consideration of other stakeholders' interests | | | | | |
| 5 | Employees' share ownership scheme enhances commitment to performance | | | | | |
| **E** | **ETHICAL LEADERSHIP** | **SA** | **A** | **N** | **D** | **SD** |
| 1 | In my bank, employees are never asked to do things that conflict with their moral principles. | | | | | |
| 2 | The CEO is interested in how employees feel and perform | | | | | |
| 3 | The CEO rewards performance in a fair manner. | | | | | |
| 4 | The bank makes it clear to employees how to deal with conflicts of interests | | | | | |
| 5 | The CEO enforces the long-term bank success against short-term wins. | | | | | |
| 6 | The CEO can be trusted to do the things he/she says. | | | | | |
| 7 | Unethical conduct reported is disciplined fairly. | | | | | |
| 8 | The top management sets a good example in terms of ethical behaviour. | | | | | |
| 9 | Employees with integrity stand a greater chance to receive a positive performance appraisal. | | | | | |
| 10 | The bank has documented codes and practices of ethics known officially to all staff | | | | | |
| 11 | A designated ethical officer monitors and reports on staff unethical behaviours | | | | | |
| 12 | My bank promotes ethical awareness through training programmes for staff | | | | | |
| 13 | The bank makes it clear to employees how to obtain proper authorisations | | | | | |
| **F** | **CORPORATE REPUTATION** | **SA** | **A** | **N** | **D** | **SD** |
| 1 | My bank is highly regarded for the quality of its products and services | | | | | |
| 2 | My bank has strong record of financial soundness | | | | | |
| 3 | My bank has global competitiveness | | | | | |
| **G** | **FINANCIAL PERFORMANCE of my bank relative to industry-average** | **1** | **2** | **3** | **4** | **5** |
| | **Very poor (1) Below Average (2) Average (3) Above Average (4) Excellent (5)** | | | | | |
| 1 | Profitability | | | | | |
| 2 | Revenue Growth (Turnover) | | | | | |
| 3 | Share price | | | | | |
| 4 | Market share | | | | | |

**SECTION C: OPEN ENDED QUESTIONS**
**PLEASE COMMENT ON THE FOLLOWING QUESTIONS:**

| | |
|---|---|
| 1 | In your opinion, what mechanisms do you consider will strengthen your bank's corporate governance? |

| | |
|---|---|
| 2 | In what ways can your bank contribute to the improvement of its community, and preservation of the natural environment? |
| 3 | In your opinion, what measures do you think can be introduced to improve (a) ethical culture in your bank? and (b) reputation of your bank? |
| 4 | In what ways do you think CBN/NDIC regulation influence financial innovation? |
| 5 | In what ways can your bank utilise corporate governance as a strategy to achieve long-term survival? |

Please state any additional comments you may have below:

Thank you for your co-operation in completing this questionnaire.

## Appendix B: Semi-Structured Interview

1. What in your opinion are the causes of recent bank failure in Nigeria?

2. a). How would you describe the term 'corporate governance', and b). what do you consider the elements of your bank's corporate governance framework?

3. In what ways do you consider corporate governance as an influence on your bank's performance?

4. In your opinion, what role does ethical leadership play in promoting your bank's reputation?

5. How will you describe the role of regulators (CBN/NDIC) in promoting financial innovation?

6. In what practical ways do the board, management and ownership structure of your bank influence its social and environmental responsibility?

7. What do you consider the necessary requirements for achieving sustainability in your bank?

8. What do you consider the state of corporate governance in Nigeria, and how can it be improved?

9. In your opinion, how can corporate governance enhance sustainability?

10. What additional information would you like to share on what we have or have not covered?

## Appendix C: Results of Hausman Tests on Influence of Agency Mechanisms on Financial Performance

### ROA:

Dependent Variable: LROA
Total panel (unbalanced) observations: 54
Swamy and Arora estimator of component variances

| Variable | Coefficient | Std. Error | t-Statistic | Prob. |
|---|---|---|---|---|
| C | -10.96172 | 5.096409 | -2.150872 | 0.0367 |
| LBETHN | 1.649493 | 0.322721 | 5.111204 | 0.0000 |
| LCEOT | 0.019871 | 0.096035 | 0.206913 | 0.8370 |
| LCEOA | 2.826744 | 1.128744 | 2.504326 | 0.0158 |
| LINO | -0.104525 | 0.055825 | -1.872378 | 0.0674 |
| LBGDR | 0.003557 | 0.173901 | 0.020452 | 0.9838 |
| TM | -0.034636 | 0.007322 | -4.730369 | 0.0000 |

| | | | |
|---|---|---|---|
| R-squared | 0.539177 | | |
| Adjusted R-squared | 0.480349 | | |
| F-statistic | 9.165251 | Durbin-Watson stat | 1.382851 |
| Prob(F-statistic) | 0.000001 | | |

Unweighted Statistics

| | | | |
|---|---|---|---|
| R-squared | 0.539177 | Mean dependent var | 0.698073 |
| Sum squared resid | 12.50485 | Durbin-Watson stat | 1.382851 |

Correlated Random Effects - Hausman Test
Equation: Untitled
Test cross-section random effects

| Test Summary | Chi-Sq. Statistic | Chi-Sq. d.f. | Prob. |
|---|---|---|---|
| Cross-section random | 18.864212 | 6 | 0.0044 |

Cross-section random effects test comparisons:

| Variable | Fixed | Random | Var(Diff.) | Prob. |
|---|---|---|---|---|
| LBETHN | 0.963083 | 1.649493 | 0.070349 | 0.0097 |
| LCEOT | -0.051011 | 0.019871 | 0.006088 | 0.3637 |
| LCEOA | 3.542714 | 2.826744 | 1.523608 | 0.5619 |
| LINO | -0.154484 | -0.104525 | 0.004175 | 0.4394 |
| LBGDR | -0.264949 | 0.003557 | 0.008816 | 0.0042 |
| TM | -0.016045 | -0.034636 | 0.000127 | 0.0996 |

### ROE

Dependent Variable: LROE
Total panel (unbalanced) observations: 54
Swamy and Arora estimator of component variances

| Variable | Coefficient | Std. Error | t-Statistic | Prob. |
|---|---|---|---|---|
| C | -0.646982 | 5.786674 | -0.111806 | 0.9115 |
| LBETHN | 0.642524 | 0.323677 | 1.985078 | 0.0530 |
| LCEOT | 0.031133 | 0.106031 | 0.293620 | 0.7703 |
| LCEOA | 1.876966 | 1.545839 | 1.214206 | 0.2307 |
| LINO | -0.197588 | 0.057526 | -3.434746 | 0.0012 |
| LBGDR | 0.069959 | 0.226251 | 0.309208 | 0.7585 |
| LCEOR | -0.091152 | 0.114075 | -0.799054 | 0.4283 |

Effects Specification

| | S.D. | Rho |
|---|---|---|
| Cross-section random | 3.87E-06 | 0.0000 |
| Idiosyncratic random | 0.499942 | 1.0000 |

Weighted Statistics

| | | | |
|---|---|---|---|
| R-squared | 0.226450 | Mean dependent var | 2.688720 |
| Adjusted R-squared | 0.127699 | S.D. dependent var | 0.715272 |
| S.E. of regression | 0.668043 | Sum squared resid | 20.97523 |
| F-statistic | 2.293141 | Durbin-Watson stat | 0.763663 |
| Prob(F-statistic) | 0.050515 | | |

Unweighted Statistics

| | | | |
|---|---|---|---|
| R-squared | 0.226450 | Mean dependent var | 2.688720 |

| | | | |
|---|---|---|---|
| Sum squared resid | 20.97523 | Durbin-Watson stat | 0.763663 |

Correlated Random Effects - Hausman Test
Equation: Untitled
Test cross-section random effects

| Test Summary | Chi-Sq. Statistic | Chi-Sq. d.f. | Prob. |
|---|---|---|---|
| Cross-section random | 42.920495 | 6 | 0.0000 |

Cross-section random effects test comparisons:

| Variable | Fixed | Random | Var(Diff.) | Prob. |
|---|---|---|---|---|
| LBETHN | 0.278775 | 0.642524 | 0.066285 | 0.1577 |
| LCEOT | -0.150373 | 0.031133 | 0.006248 | 0.0217 |
| LCEOA | 5.266132 | 1.876966 | 1.926780 | 0.0146 |
| LINO | -0.215735 | -0.197588 | 0.005043 | 0.7983 |
| LBGDR | -0.117252 | 0.069959 | 0.005464 | 0.0113 |
| LCEOR | -0.223202 | -0.091152 | 0.002536 | 0.0087 |

NIM
Dependent Variable: LNIM
Total panel (unbalanced) observations: 56
Swamy and Arora estimator of component variances

| Variable | Coefficient | Std. Error | t-Statistic | Prob. |
|---|---|---|---|---|
| C | 7.978122 | 1.863545 | 4.281154 | 0.0001 |
| LBGDR | 0.004109 | 0.073209 | 0.056129 | 0.9555 |
| LCEOT | 0.055658 | 0.037557 | 1.481938 | 0.1448 |
| LCEOA | 0.003705 | 0.477451 | 0.007759 | 0.9938 |
| LINO | -0.078172 | 0.020750 | -3.767296 | 0.0004 |
| LBETHN | -0.153718 | 0.117332 | -1.310119 | 0.1963 |
| LCEOR | -0.197434 | 0.038669 | -5.105677 | 0.0000 |

Effects Specification

| | S.D. | Rho |
|---|---|---|
| Cross-section random | 1.59E-07 | 0.0000 |
| Idiosyncratic random | 0.181331 | 1.0000 |

Weighted Statistics

| | | | |
|---|---|---|---|
| R-squared | 0.440309 | Mean dependent var | 2.603225 |
| Adjusted R-squared | 0.371775 | S.D. dependent var | 0.274327 |
| S.E. of regression | 0.217433 | Sum squared resid | 2.316576 |
| F-statistic | 6.424719 | Durbin-Watson stat | 1.411811 |
| Prob(F-statistic) | 0.000048 | | |

Unweighted Statistics

| | | | |
|---|---|---|---|
| R-squared | 0.440309 | Mean dependent var | 2.603225 |
| Sum squared resid | 2.316576 | Durbin-Watson stat | 1.411811 |

Correlated Random Effects - Hausman Test
Equation: Untitled
Test cross-section random effects

| Test Summary | Chi-Sq. Statistic | Chi-Sq. d.f. | Prob. |
|---|---|---|---|
| Cross-section random | 27.453388 | 6 | 0.0001 |

Cross-section random effects test comparisons:

| Variable | Fixed | Random | Var(Diff.) | Prob. |
|---|---|---|---|---|
| LBGDR | -0.019889 | 0.004109 | 0.000622 | 0.3358 |
| LCEOT | 0.087528 | 0.055658 | 0.000823 | 0.2666 |
| LCEOA | -0.454666 | 0.003705 | 0.239315 | 0.3488 |
| LINO | -0.004011 | -0.078172 | 0.000665 | 0.0040 |
| LBETHN | 0.108029 | -0.153718 | 0.008536 | 0.0046 |
| LCEOR | -0.188034 | -0.197434 | 0.000366 | 0.6232 |

TBQ

Dependent Variable: LTBQ
Total panel (unbalanced) observations: 56
Swamy and Arora estimator of component variances

| Variable | Coefficient | Std. Error | t-Statistic | Prob. |
|---|---|---|---|---|
| C | -1.622811 | 4.346774 | -0.373337 | 0.7105 |
| LBGDR | -0.156379 | 0.194371 | -0.804541 | 0.4250 |
| LCEOT | -0.081040 | 0.099783 | -0.812168 | 0.4206 |
| LCEOA | 1.276900 | 1.239248 | 1.030383 | 0.3079 |
| LBETHN | 1.591719 | 0.321120 | 4.956765 | 0.0000 |
| LCEOR | -0.292571 | 0.101824 | -2.873312 | 0.0060 |
| TM | -0.046965 | 0.006954 | -6.753802 | 0.0000 |

Effects Specification

| | S.D. | Rho |
|---|---|---|
| Cross-section random | 2.13E-05 | 0.0000 |
| Idiosyncratic random | 0.478505 | 1.0000 |

Weighted Statistics

| | | | |
|---|---|---|---|
| R-squared | 0.376351 | Mean dependent var | -0.157485 |
| Adjusted R-squared | 0.299986 | S.D. dependent var | 0.897819 |
| S.E. of regression | 0.751177 | Sum squared resid | 27.64906 |
| F-statistic | 4.928306 | Durbin-Watson stat | 0.914736 |
| Prob(F-statistic) | 0.000515 | | |

Unweighted Statistics

| | | | |
|---|---|---|---|
| R-squared | 0.376351 | Mean dependent var | -0.157485 |
| Sum squared resid | 27.64906 | Durbin-Watson stat | 0.914736 |

Correlated Random Effects - Hausman Test
Equation: Untitled
Test cross-section random effects

| Test Summary | Chi-Sq. Statistic | Chi-Sq. d.f. | Prob. |
|---|---|---|---|
| Cross-section random | 77.755878 | 6 | 0.0000 |

Cross-section random effects test comparisons:

| Variable | Fixed | Random | Var(Diff.) | Prob. |
|---|---|---|---|---|
| LBGDR | -0.398601 | -0.156379 | 0.003723 | 0.0001 |
| LCEOT | 0.042860 | -0.081040 | 0.005821 | 0.1044 |
| LCEOA | -2.121786 | 1.276900 | 1.427477 | 0.0044 |
| LBETHN | 0.867090 | 1.591719 | 0.072564 | 0.0071 |
| LCEOR | -0.564132 | -0.292571 | 0.003165 | 0.0000 |
| TM | 0.024871 | -0.046965 | 0.000114 | 0.0000 |

NPL

Dependent Variable: LNPL
Total panel (unbalanced) observations: 56
Swamy and Arora estimator of component variances

| Variable | Coefficient | Std. Error | t-Statistic | Prob. |
|---|---|---|---|---|
| C | 27.94120 | 7.709329 | 3.624336 | 0.0007 |
| LBETHN | -1.424269 | 0.485391 | -2.934270 | 0.0051 |
| LCEOT | -0.193680 | 0.155371 | -1.246560 | 0.2185 |
| LCEOA | -2.328565 | 1.975174 | -1.178916 | 0.2441 |
| LINO | 0.061339 | 0.085841 | 0.714567 | 0.4783 |
| LBGDR | 0.300295 | 0.302860 | 0.991531 | 0.3263 |
| LCEOR | 0.366001 | 0.159972 | 2.287898 | 0.0265 |

Effects Specification

| | S.D. | Rho |
|---|---|---|
| Cross-section random | 4.14E-06 | 0.0000 |
| Idiosyncratic random | 0.750152 | 1.0000 |

Weighted Statistics

| | | | |
|---|---|---|---|
| R-squared | 0.266837 | Mean dependent var | 24.08805 |
| Adjusted R-squared | 0.177062 | S.D. dependent var | 0.952250 |
| S.E. of regression | 0.863842 | Sum squared resid | 36.56497 |
| F-statistic | 2.972284 | Durbin-Watson stat | 0.965865 |
| Prob(F-statistic) | 0.014836 | | |

| Unweighted Statistics | | | |
|---|---|---|---|
| R-squared | 0.266837 | Mean dependent var | 24.08805 |
| Sum squared resid | 36.56497 | Durbin-Watson stat | 0.965865 |

Correlated Random Effects - Hausman Test
Equation: Untitled
Test cross-section random effects

| Test Summary | Chi-Sq. Statistic | Chi-Sq. d.f. | Prob. |
|---|---|---|---|
| Cross-section random | 21.978079 | 6 | 0.0012 |

Cross-section random effects test comparisons:

| Variable | Fixed | Random | Var(Diff.) | Prob. |
|---|---|---|---|---|
| LBETHN | -0.713733 | -1.424269 | 0.146083 | 0.0630 |
| LCEOT | 0.245957 | -0.193680 | 0.014085 | 0.0002 |
| LCEOA | -7.896647 | -2.328565 | 4.095655 | 0.0059 |
| LINO | 0.154127 | 0.061339 | 0.011383 | 0.3845 |
| LBGDR | 0.591347 | 0.300295 | 0.010638 | 0.0048 |
| LCEOR | 0.656598 | 0.366001 | 0.006265 | 0.0002 |

**Appendix "D" Criteria for Selection of Study Population - Deposit Money Banks**

1. The bank must have been in operation before the conclusion of the bank consolidation exercise in

| | | **INCLUSION CRITERIA** | | | | |
|---|---|---|---|---|---|---|
| | **DEPOSIT MONEY BANK** | **1** | **2** | **3** | **4** | **5** |
| 1 | Access Bank Plc | ✓ | ✓ | ✓ | ✓ | ✓ |
| 2 | Citibank Nigeria Limited | ✓ | X | ✓ | X | ✓ |
| 3 | Diamond Bank Plc | ✓ | ✓ | ✓ | ✓ | ✓ |
| 4 | Ecobank Nigeria Plc | ✓ | X | ✓ | ✓ | ✓ |
| 5 | Enterprise Bank | X | ✓ | X | X | X |
| 6 | Fidelity Bank Plc | ✓ | ✓ | ✓ | √ | ✓ |
| 7 | First City Monument Bank Plc | ✓ | ✓ | ✓ | ✓ | ✓ |
| 8 | Guaranty Trust Bank Plc | ✓ | ✓ | ✓ | ✓ | ✓ |
| 9 | Key Stone Bank | X | ✓ | X | X | X |
| 10 | MainStreet Bank | X | ✓ | X | X | X |
| 11 | Skye Bank Plc | X | ✓ | X | X | ✓ |
| 12 | Stanbic-IBTC Bank Ltd. | X | X | X | X | ✓ |
| 13 | Standard Chartered Bank Nigeria Ltd. | ✓ | X | ✓ | X | ✓ |
| 14 | Sterling Bank Plc | X | ✓ | X | X | ✓ |
| 15 | SunTrust Bank Nigeria Limited | X | ✓ | X | X | ✓ |
| 16 | Union Bank of Nigeria Plc | ✓ | X | ✓ | ✓ | X |
| 17 | United Bank of Africa Plc | ✓ | ✓ | ✓ | ✓ | ✓ |
| 18 | Unity Bank Plc | X | X | X | X | X |
| 19 | Wema Bank Plc | ✓ | ✓ | ✓ | ✓ | X |
| 20 | Zenith Bank Plc | ✓ | ✓ | ✓ | ✓ | ✓ |
| 21 | Heritage Banking Company Ltd. | X | ✓ | X | X | X |
| 22 | First Bank Nigeria Limited | ✓ | ✓ | ✓ | ✓ | ✓ |

December 2005;

2. Bank must be publicly-listed, and not be foreign-owned (due to the financial reporting availability);
3. Bank must have its name/identity retained from pre-consolidation to date (some banks have gone through several mergers and acquisition arrangements leading to loss of their identity and a possible distortion of their data);
4. Bank must have their financial data for 2006-2016 (the 11-year period represents the whole post-consolidation financial years to-date) available in the public domain.
5. Bank must not have been a product of restructure, resuscitation or take-over by CBN, and, or Assets Management Company of Nigeria (AMCON)

**Legends:**

X – Fails inclusion test

✓ - Passes inclusion test

## Appendix “E”: Nigerian Deposit Money Banks Corporate Governance Practices Index

| | | | |
|---|---|---|---|
| | **NIGERIAN DEPOSIT MONEY BANKS' CORPORATE GOVERNANCE INDEX SCORE** | | |
| | **BOARD STRUCTURE INDEX** | **RATING** | **MAX** |
| **CODE** | **Independence elements** | | |
| IND001 | Full Board consists of greater number of non-executive directors | 0-1 | 1 |
| IND002 | CEO is NOT board chairman | 0-1 | 1 |
| IND003 | Board chairman is a Non-Executive director | 0-1 | 1 |
| IND004 | Board has at least two (2) non-executive directors designated as Independent directors | 0-1 | 1 |
| IND005 | Board member(s) has/have access to independent professional legal advice on matters affecting the bank | 0-1 | 1 |
| IND006 | Board chairman not a member/chairman of any board committee | 0-1 | 1 |
| | **SUB-TOTAL MAX SCORE** | | **6** |
| | | | |
| | **Committee elements** | | |
| COM001 | Board Audit committee exists, comprising of NEDs, and chaired by NED | 0-1 | 1 |
| COM002 | Board Risk Management Committee exists, with over 50% as NEDs, and chaired by NED | 0-1 | 1 |
| COM003 | A stand-alone Remuneration/Compensation committee exists, with over 50% as NEDs, and chaired by NED | 0-1 | 1 |
| COM004 | A stand-alone Nomination committee exists , with over 50% as NEDs, and chaired by NED | 0-1 | 1 |
| COM005 | A stand-alone Corporate Governance committee exists, with over 50% as NEDs, and chaired by NED | 0-1 | 1 |
| COM006 | Statutory Audit Committee exists, with at least 50% as shareholders representatives, and chaired by a shareholder representative who is professionally qualified (Chartered Accountant) | 0-1 | 1 |
| COM007 | Existence of a Board Information Technology Governance Committee | 0-1 | 1 |
| COM008 | Board composition made up of a mix of characteristics: i) functional/sector experts, ii) diversity, iii) senior level leadership experience | 0-6 | 6 |
| | **SUB-TOTAL MAX SCORE** | | **13** |
| | **Board Operations and Processes index** | | |
| BOD_OPS001 | The board monitors the bank’s corporate governance practices for improvement. | 0-1 | 1 |
| BOD_OPS002 | The board provides strategic direction for the bank | 0-1 | 1 |
| BOD_OPS003 | Board size is between five (5) and twenty (20) members | 0-1 | 1 |
| BOD_OPS004 | The board elects, monitors and replaces executives when necessary. | 0-1 | 1 |
| BOD_OPS005 | The Board has a succession plan in place for the CEO, other executive Directors and top management staff | 0-1 | 1 |
| BOD_OPS006 | At least quarterly (4) regular board meetings per year | 0-1 | 1 |
| BOD_OPS007 | At least quarterly (4) regular board committee meetings per year | 0-1 | 1 |
| BOD_OPS008 | Bank has a system/mechanism to evaluate CEO | 0-1 | 1 |
| BOD_OPS009 | Bank evaluates executive and non-executive directors (Peer evaluation of Board Members) | 0-1 | 1 |
| BOD_OPS010 | Directors receive regular board training | 0-1 | 1 |
| BOD_OPS011 | Board receives materials in advance of meeting | 0-1 | 1 |
| BOD_OPS012 | Bank has code of ethics | 0-1 | 1 |
| BOD_OPS013 | Bank has specific charter/bylaw/policy to govern board and board committees | 0-1 | 1 |
| BOD_OPS014 | Shareholders approve directors’ remuneration | 0-1 | 1 |

| | | | |
|---|---|---|---|
| BOD_OPS015 | Non-executive directors attend at least 75% of meetings | 0-1 | 1 |
| BOD_OPS016 | CBN approves (every) director's appointment | 0-1 | 1 |
| BOD_OPS017 | Annual Board appraisal conducted by independent consultant | 0-1 | 1 |
| BOD_OPS018 | Provides biographical information (name, age, qualification, country of residence, class of directorship, work experience/occupation in the last ten (10) years) on any proposed director for appointment/re-election | 0-1 | 1 |
| BOD_OPS019 | Provides information (about current directorships and appointments with statutory or regulatory authorities in the preceding five (5) years) on any proposed director for appointment/re-election | 0-1 | 1 |
| BOD_OPS020 | Provides information (about shareholding in the company and its subsidiary) on any proposed director for appointment/re-election | 0-1 | 1 |
| BOD_OPS021 | Attendance at AGM by Chairmen of board committees and statutory audit committee | 0-1 | 1 |
| BOD_OPS022 | Tenure of directors (chairman, independent, non-executive, executive) in compliance with laws | 0-1 | 1 |
| BOD_OPS023 | High quality information request by NED from Senior management/EXCO, and access to critical information from inside and outside the firm | 0-2 | 2 |
| | **SUB-TOTAL MAX SCORE** | | **24** |
| | | | |
| | **<u>Disclosure index</u>** | | |
| DISC001 | Disclosure of total number of meetings held in the financial year and attendance by each director | 0-1 | 1 |
| DISC002 | Disclosure of details of shares held by Directors and their related parties (Direct and Indirect Shareholding) | 0-1 | 1 |
| DISC003 | International Accounting and Auditing Standards adopted on major items | 0-1 | 1 |
| DISC004 | Directors' details - remuneration, shareholding, performance evaluation | 0-3 | 3 |
| DISC005 | Corporate governance structure/framework | 0-1 | 1 |
| DISC006 | Board committee composition, membership, and chairmanship | 0-1 | 1 |
| DISC007 | Bank puts quarterly financial statements on its website | 0-1 | 1 |
| DISC008 | Bank puts annual report on its website | 0-1 | 1 |
| DISC009 | Executive director compensation policy disclosed | 0-1 | 1 |
| DISC010 | Disclosure on concentration of assets, liabilities and off-balance sheet engagements by sector, geography, and product | 0-1 | 1 |
| DISC011 | Loans and advances / Credit quality | 0-1 | 1 |
| DISC012 | Lending/borrowing to/from subsidiaries and associates | 0-1 | 1 |
| DISC013 | Loans and advances/funding or commitment lines from foreign institution | 0-1 | 1 |
| DISC014 | Related party transactions | 0-1 | 1 |
| DISC015 | Insider-related credits (key personnel, directors etc) | 0-1 | 1 |
| DISC016 | Disclosure of regulatory sanctions and penalties during the year | 0-1 | 1 |
| DISC017 | Capital Structure/Adequacy Ratio | 0-1 | 1 |
| DISC018 | Information on strategic modification to the core business | 0-1 | 1 |
| DISC019 | Frauds and forgeries | 0-1 | 1 |
| DISC020 | Contingency Planning Framework | 0-1 | 1 |
| DISC021 | Disclosure of shareholding structure analysis | 0-1 | 1 |
| DISC022 | Bank discloses shareholders with 5% or more shareholding, | 0-1 | 1 |
| | **SUB-TOTAL MAX SCORE** | | **24** |
| | **<u>Risk Management</u>** | | |
| RSK_MGT001 | Existence of a risk management framework | 0-1 | 1 |

| | | | |
|---|---|---|---|
| RSK_MGT002 | Disclosure of risk management practices | 0-1 | 1 |
| RSK_MGT003 | Board reviews risk management and internal control system effectiveness at least annually | 0-1 | 1 |
| RSK_MGT004 | Practices indicating the Board's responsibility | 0-1 | 1 |
| RSK_MGT005 | Summary of external auditors observed lapses (Key Audit Matters) on risk management practices | 0-1 | 1 |
| RSK_MGT006 | Significant risks disclosure (actual and potential bank-specific risks) | 0-1 | 1 |
| RSK_MGT007 | Existence of a whistle-blowing policy | 0-1 | 1 |
| RSK_MGT008 | Whistleblowing policy known to employees and other stakeholders (Hotline/Website/Email) | 0-1 | 1 |
| RSK_MGT009 | Whistleblowing policy assures confidentiality, call for action, and protection | 0-1 | 1 |
| RSK_MGT010 | All related party transactions are reviewed and monitored by a sufficient number of directors capable of exercising objective and independent judgment (specifically the Independent Non-Executive Directors or Statutory Audit Committee) | 0-3 | 3 |
| RSK_MGT011 | Bank has a competent and independent Compliance function that reports to the board audit committee | 0-3 | 3 |
| RSK_MGT012 | Bank has risk management and approval processes for new or expanded products or services, lines of business and markets, as well as for large and complex transactions that require significant use of resources or have hard-to-quantify risks | 0-3 | 3 |
| RSK_MGT013 | Bank has an effective independent risk management function, under the direction of a Chief Risk Officer (CRO), with sufficient stature, independence, resources and direct access to the board/board committee on risk management | 0-3 | 3 |
| RSK_MGT014 | The risk management function is sufficiently independent of the business units and not involved in revenue generation. | 0-3 | 3 |
| RSK_MGT015 | Independent, external review of bank's internal control and risk management function periodically | 0-3 | 3 |
| RSK_MGT016 | Board discloses detailed governance procedures and practices over its subsidiaries covering at a minimum - group structure, appropriate subsidiary board and management structure, effective system to facilitate information exchange, sufficient resources to monitor subsidiaries' compliance with all regulatory and governance requirements, and effective relationship with regulators in home and foreign countries) | 0-3 | 3 |
| RSK_MGT017 | Reporting on monitoring of bank's own client's corporate governance practices (e.g. insisting on provision of independent auditor-verified consolidated financial reporting) | 0-1 | 1 |
| RSK_MGT018 | Disclosure of senior management (AGM-GM) compensation for stakeholders' evaluation vis-à-vis bank's performance | 0-2 | 2 |
| | **SUB-TOTAL MAX SCORE** | | **33** |
| | **Transparency and Integrity in Reporting elements** | | |
| TRA_INT001 | Review of financial statements by Board Audit Committee (BAC)/Statutory Audit Committee (SAC) and the full Board | 0-1 | 1 |
| TRA_INT002 | BAC composition – made up of only Non-Executive Directors, has at least three (3) members, and Chaired by Independent Director | 0-3 | 3 |
| TRA_INT003 | BAC – independent, with technical expertise (in finance), and sufficiently-sized | 0-1 | 1 |
| TRA_INT004 | BAC meets at least once every quarter | 0-1 | 1 |
| TRA_INT005 | Existence of a code of conduct to guide operations and members | 0-1 | 1 |
| TRA_INT006 | External auditors not providing conflicting client services e.g. bookkeeping, valuation services, actuarial services, internal audit, HR, investment banking services, legal or expert services | 0-1 | 1 |
| TRA_INT007 | Tenure of external auditors not exceeding ten (10) cumulative years | 0-1 | 1 |
| TRA_INT008 | Audit committee includes accounting or finance expert (and chaired by accounting expert) | 0-1 | 1 |
| TRA_INT009 | BAC is distinct and separate from other Board committees | 0-1 | 1 |
| TRA_INT010 | SAC – made up of at least 6 members, of which 50% or more are representatives appointed by shareholders, with technical expertise (in finance/banking/accounting), and one (1) in committee chair | 0-1 | 1 |
| | **SUB-TOTAL MAX SCORE** | | **12** |
| | | | |

| | | | |
|---|---|---|---|
| | **Ownership and Shareholders' rights** | | |
| OWN001 | Largest shareholder has fractional ownership of common/voting shares | 0-1 | 1 |
| OWN002 | Controlling shareholders do not have special nomination rights | 0-1 | 1 |
| OWN003 | Class of shares with preferred voting rights does not exist | 0-1 | 1 |
| OWN004 | Bank allows voting by postal ballot | 0-1 | 1 |
| OWN005 | Bank has policy against insider trading | 0-1 | 1 |
| OWN006 | Board includes at least one member elected by minority shareholders | 0-1 | 1 |
| OWN007 | No class of shares with special nomination rights | 0-1 | 1 |
| OWN008 | Bank has investor relations department (or contact person) | 0-1 | 1 |
| OWN009 | Shareholders have rights to register and transfer their share ownership | 0-1 | 1 |
| OWN010 | All shareholders of the same class are treated equally. | 0-1 | 1 |
| OWN011 | Shareholders have opportunity for effective participation and voting in key decisions at AGMs – election, removal, and remuneration of board members and/or key executives, asking questions on external audit, proposing resolutions, and placing items on the AGM agenda. | 0-1 | 1 |
| OWN012 | Shareholders are sufficiently informed about fundamental corporate changes – such as additional shares, extraordinary transactions, and changes to the bank's articles of incorporation | 0-1 | 1 |
| OWN013 | Shareholders have opportunity to consult with each other on issues concerning their basic rights (e.g. shareholders association) | 0-1 | 1 |
| OWN014 | Shareholders have right to obtain relevant and material information on a timely and regular basis (notice of meetings, statutory notices, other relevant information) | 0-1 | 1 |
| OWN015 | prompt provision of shareholders documentary evidence of ownership interest - such as share certificates, dividend warrants and related instruments | 0-1 | 1 |
| OWN016 | Notices of general meetings not earlier than twenty-one (21) days from the date on which the notice was sent out. | 0-1 | 1 |
| | **SUB-TOTAL MAX SCORE** | | **16** |
| | **TOTAL RAW SCORE OBTAINED** | | **128** |
| | **TOTAL CONVERTED SCORE (100%)** | | **100** |

## Appendix “F”: Nigerian Deposit Money Banks Sustainability Performance Reporting Index

| **DEPENDENT VARIABLE : SUSTAINABILITY PERFORMANCE REPORTING INDEX** | | |
|---|---|---|
| | | |
| **ECONOMIC INDEX** | | |
| **DIRECT ECONOMIC SUB-INDEX** | | |
| **Financial Performance - Accounting Ratios** | **SCORE** | **MAX** |
| Return On Equity | 0-3 | 3 |
| Return On Asset | 0-3 | 3 |
| Earnings growth - (PBT) | 0-3 | 3 |
| Tobin’s Q Ratio/M-T-B Ratio | 0-3 | 3 |
| Cost-to-Income Ratio or efficiency ratio | 0-5 | 5 |
| Net interest margin | 0-3 | 3 |
| Non-Performing Loans (NPL) Ratio | 0-5 | 5 |
| Liquidity Ratio Proxy (Loans to Deposit) | 0-3 | 3 |
| Leverage ratio | 0-5 | 5 |
| Capital adequacy ratio: | 0-5 | 5 |
| | | **38** |
| **INDIRECT ECONOMIC SUB-INDEX** | | |
| **Financial Inclusion** | | |
| Providing development and growth support to SMEs | 0-3 | 3 |
| Improving financial literacy and institutional practices | 0-3 | 3 |
| Does the bank report on improving access to its facilities and services: through platforms such as cash centres, e-branches, mobile money and internet? | 0-1 | 1 |
| Does the bank report on its programmes to support minorities and disadvantaged? | 0-1 | 1 |
| | | **8** |
| **TOTAL WEIGHTED ECONOMIC PERFORMANCE INDEX (50%)** | | |
| | | |
| **SOCIAL INDEX** | | |
| **Diversity/Women Economic Empowerment** | | |
| Developing and implementing a women’s economic empowerment policy | 0-3 | 3 |
| Establish a Women’s economic empowerment committee { to oversee accountability for gender diversity and to steer gender inclusive strategies}, and invest and dedicate resources for a strong female talent pipeline (to fast-track transition from middle management, to executive, and Board level positions | 0-3 | 3 |
| Develop initiatives and programmes to promote and celebrate women empowerment { support and establish initiatives designed to educate and empower women with new skills and provide opportunities for them to connect with senior role models | 0-3 | 3 |
| Bank runs a women empowerment fund? | 0-3 | 3 |
| Does the bank report on Percentage of women on the board? | 0-1 | 1 |

| | | |
|---|---|---|
| Contd… | | |
| Does the bank report on Percentage of women among senior management (AGM-GM)? | 0-1 | 1 |
| Has the company demonstrated its commitment to diversity through strong representation of women, minorities, and the disabled on boards of directors, in top management, and/or among the company's highest paid employees? | 0-3 | 3 |
| Has the company demonstrated its commitment to diversity through its training and advancement programs (e.g., support networks, management reviews, mentoring), and conducting diversity training for its employees? | 0-3 | 3 |
| Has the company demonstrated its commitment to diversity through participation in women and minority vendor and banking programs? | 0-3 | 3 |
| Does the company, at a minimum, have in place specifically stated policies against discrimination in hiring and promotion? | 0-1 | 1 |
| Does the company have gender equity in wages? | 0-1 | 1 |
| | | **25** |
| | | |
| **Ethics/Governance** | | |
| Develop robust and transparent procedures, which entail a clear governance structure, limits of authority, standards and codes of conduct | 0-1 | 1 |
| Establish internal and external Environmental & Social (E&S) audit procedures for continuous improvement | 0-3 | 3 |
| Does the bank report on anti-bribery and corruption policy statement? | 0-1 | 1 |
| Does the bank have a written Code of Business Conduct used as a guide to help employees live up to the company's ethical standards? | 0-1 | 1 |
| **Does the bank have policy with specific focus on:** | | |
| · Equal Employment Opportunity? (1) | 0-1 | 1 |
| · Conflicts of interest? (1) | 0-1 | 1 |
| · Bribery and corruption? (1) | 0-1 | 1 |
| · Use and public disclosure of inside info, and the use of confidential and proprietary information? (1) | 0-1 | 1 |
| · Health, safety, and environment? (1) | 0-1 | 1 |
| · Harassment? (1) | 0-1 | 1 |
| | | **12** |
| **CSR/Community Investment/Philanthropy** | | |
| Does the bank report on its community involvement? | 0-1 | 1 |
| Does the bank devote a reasonable percentage of its pre-tax profit to charitable contribution? | 0-3 | 3 |
| Does the bank have a charitable foundation ? | 0-1 | 1 |
| Is there evidence of new initiatives implemented by or awards given to the company with respect to its performance in community program? | 0-3 | 3 |
| Does the company disclose the level of community programs involved in during the year? | 0-3 | 3 |
| Does the bank report on program to support employee giving and volunteerism programs? | 0-3 | 3 |
| Does the company have partnerships with local schools, hospitals or community-based groups? | 0-1 | 1 |

| | | |
|---|---|---|
| Contd…. | | |
| Is the company committed to donating a given percentage of its pretax profits to charitable organizations and if so, what percentage is the target goal? | 0-1 | 1 |
| | | **16** |
| **Human Rights** | | |
| Does the bank report on human rights policy? | 0-1 | 1 |
| Does the bank report on ban on discrimination against any group or individual, based on race, gender, religion, culture, politics or economic background? | 0-1 | 1 |
| Does the bank report on recognition of employees' entitlement to safe and fair labour conditions? | 0-1 | 1 |
| Does the bank report on collective and individual rights to associate and speak freely? | 0-1 | 1 |
| Does the bank report on prohibition of child labour and forced labour? | 0-1 | 1 |
| Does the bank report on training of staff on human rights issues? | 0-1 | 1 |
| Does the bank report on regulatory and legal compliance, and penalties during the year? | 0-1 | 1 |
| | | **7** |
| **Employees/Labour Laws** | | |
| Does the bank report a reduction in total employees (attrition) over prior year? | 0-1 | 1 |
| Does the bank report on Employee training and development? | 0-3 | 3 |
| Does the bank report on its commitment to employees through implementation of innovative work/life programs (e.g. flextime, job sharing, child care, employees' work/life balance)? | 0-3 | 3 |
| Does the bank report on employees' Profit sharing program | 0-1 | 1 |
| Does the bank report on Employees' Share Ownership program | 0-1 | 1 |
| Does the bank report on how to improve employee health & safety? | 0-3 | 3 |
| Does the bank report on employees' grievance mechanisms? | 0-3 | 3 |
| Does the bank report on employees with disabilities? | 0-3 | 3 |
| Does the bank report on recognition of the right to collective bargaining? | 0-3 | 3 |
| Does the bank report on non-discrimination in respect of employment and occupation? | 0-1 | 1 |
| | | **22** |
| | | |
| **Product Responsibility** | | |
| Does the bank have a statement on the treatment of its customers? | 0-1 | 1 |
| Does the bank report on ethical marketing practices for its products and services? | 0-3 | 3 |
| Does the bank report on how to monitor or improve the quality and safety of its products/services? | 0-3 | 3 |
| Does the bank report on Consumer rights and protection? | 0-1 | 1 |
| Does the bank report on health, safety and welfare of its customers? | 0-1 | 1 |
| | | **9** |
| **TOTAL WEIGHTED SOCIAL PERFORMANCE INDEX (30%)** | | |
| | | |

| | | |
|---|---|---|
| <u>**ENVIRONMENTAL INDEX**</u> | | |
| Environmental & Social Risk Management Policy - The bank reports a developed Environmental & Social management procedures as a formal part of client engagement and approval process | 0-3 | 3 |
| Emissions Reduction - reducing business travel and commuting Does the bank report on carbon dioxide (CO2) emissions footprint reduction/lowering (greenhouse gas emissions)? | 0-3 | 3 |
| Water Efficiency - reduce, reuse and recycle water consumption | 0-3 | 3 |
| Paper Efficiency - reduce, reuse and recycle paper consumption. Does the bank report on initiatives on resource efficiency (e.g. paper, materials)? Or does the bank report on initiatives to reduce, reuse, or recycle etc.? | 0-3 | 3 |
| Energy Efficiency - utilising renewable power generation, Does the bank report on initiatives to use renewable energy sources (e.g. wind, solar)? Or does the bank report on reducing energy consumption in general? | 0-3 | 3 |
| Waste Management - eliminating, reducing, and recycling product and paper waste; using less material and implementing more efficient waste management systems to reduce the amount of waste produced. Does the bank report on waste management and disposal initiatives? | 0-3 | 3 |
| Green Technology - incorporate relevant or leading "green" technology or energy efficient building standards in new and existing facilities | 0-3 | 3 |
| Socially Responsible Investment - Is there a disclosure of Socially Responsible Investment policy or practice supporting investment in sustainable, innovative business opportunities | 0-1 | 1 |
| Does the bank report on sustainability criteria in vendor/supplier selection, responsible sourcing? | 0-1 | 1 |
| Does the bank report on the environmental impacts of its financial products? | 0-1 | 1 |
| Does the bank have a managerial structure and responsibility (e.g. a department, a manager) for sustainability initiatives and monitoring? | 0-1 | 1 |
| Is there a disclosure of sustainability reports audit – Internal or external? | 0-1 | 1 |
| Is there a disclosure of employee training and communication on sustainability practices? | 0-1 | 1 |
| Does the Chairman/CEO statement mention sustainability (i.e. regarding environmental, social, and/or governance) in the Annual Report? | 0-1 | 1 |
| Does the bank have a Sustainability Report or (if integrated) a separate section for sustainability reporting in the Annual Report? | 0-1 | 1 |
| Does the bank report on the Board involvement in sustainability e.g. a Board committee? | 0-1 | 1 |
| Does the bank report on sustainability collaborative partnerships e.g. with the private/public sector, NGO etc on improving sustainability related issues? | 0-1 | 1 |
| **TOTAL WEIGHTED ENVIRONMENTAL PERFORMANCE INDEX (20%)** | | **31** |
| | | |
| **GRAND-TOTAL (RAW SCORE)** | | **168** |
| | | |
| **OVERALL WEIGHTED SCORE (100%)** | | |
| | | |
| **Key to Non-financial score rating:** | | |
| No-0; Yes-1 | | |
| No-0; General Statement-1; Detailed reporting 2; Magnitude with data 3 | | |

| | | |
|---|---|---|
| | | |
| | | |
| | | |
| | | |
| **SCORING GUIDE (Rating agencies and Bankers Ratio Guide)** | | |
| | | |
| | | |
| ROE: Negative = 0, Below 11% = 1, 11% & 20% = 2, Above 20% = 3 | | |
| ROA: Negative = 0, Below 1.6% = 1, 1.6% & 3% = 2, Above 3% = 3 | | |
| Earnings Growth: Negative = 0, Below 11% = 1, 11% & 20% = 2, Above 20% = 3 | | |
| Tobin's Q: Below 1 = 0, Btw 1 & 1.5 = 1, Above 1.5 = 3 | | |
| Cost-to-Income Ratio: 50% or Below = 5, 51 -65% = 3, 66-75% = 1, Above 75% = 0 | | |
| Net Interest Margin: Negative = 0, Below 50% = 1, 50% & 60% = 2, Above 60% = 3 | | |
| Non-Performing Loans Ratio: Between 0 & 2% = 5, Btw 2 and 5% = 2, Above 5% = 0 | | |
| Loans-to-Deposit Ratio: Above 90% = 0, Between 80 and 89% = 1, 70-79% = 2, 50-69% = 3 | | |
| Leverage Ratio: Below 3% = 0, 3-5% = 3, Above 5% = 5 | | |
| Capital Adequacy Ratio: Between 0 & 15% = 0, >15 <= 20% = 3, Above 20% = 5 | | |
| | | |

## APPENDIX 'G': SENSITIVITY ANALYSIS – AGENCY MECHANISMS ON FINANCIAL PERFORMANCE

### CEO's age (54years and above) on Financial Performance

Dependent Variable: LROA
Method: Panel Least Squares
Sample: 2006 2016
Periods included: 11
Cross-sections included: 7
Total panel (unbalanced) observations: 54

| Variable | Coefficient | Std. Error | t-Statistic | Prob. |
|---|---|---|---|---|
| C | 2.236287 | 2.186684 | 1.022684 | 0.3125 |
| LBETHN | 0.777658 | 0.410196 | 1.895822 | 0.0650 |
| LCEOT | 0.002652 | 0.111137 | 0.023858 | 0.9811 |
| LINO | -0.125315 | 0.079253 | -1.581202 | 0.1215 |
| LBGDR | -0.144446 | 0.206137 | -0.700729 | 0.4874 |
| TM | -0.012963 | 0.012723 | -1.018863 | 0.3142 |
| CEOA_ABOVE_54 | 0.661548 | 0.277515 | 2.383827 | 0.0218 |

Effects Specification

Cross-section fixed (dummy variables)

| | | | |
|---|---|---|---|
| R-squared | 0.692479 | Durbin-Watson stat | 1.753239 |
| Adjusted R-squared | 0.602473 | | |
| F-statistic | 7.693693 | | |
| Prob(F-statistic) | 0.000000 | | |

Dependent Variable: LROE
Method: Panel Least Squares
Sample: 2006 2016
Periods included: 11
Cross-sections included: 7
Total panel (unbalanced) observations: 54

| Variable | Coefficient | Std. Error | t-Statistic | Prob. |
|---|---|---|---|---|
| C | 7.142210 | 2.804643 | 2.546567 | 0.0147 |
| LBETHN | 0.136030 | 0.423669 | 0.321077 | 0.7498 |
| LCEOT | -0.047156 | 0.118602 | -0.397598 | 0.6930 |
| LINO | -0.177657 | 0.088061 | -2.017427 | 0.0502 |
| LBGDR | -0.131832 | 0.238920 | -0.551782 | 0.5841 |
| LCEOR | -0.042647 | 0.102594 | -0.415684 | 0.6798 |
| CEOA_ABOVE_54 | 0.720204 | 0.299936 | 2.401194 | 0.0210 |

Effects Specification

Cross-section fixed (dummy variables)

| | | | |
|---|---|---|---|
| R-squared | 0.616750 | Durbin-Watson stat | 1.474986 |
| Adjusted R-squared | 0.504579 | | |
| F-statistic | 5.498312 | | |
| Prob(F-statistic) | 0.000018 | | |

**Insider Ownership (pegged at 2% aggregate) on Financial Performance**

Dependent Variable: LROA
Method: Panel Least Squares
Sample: 2006 2016
Periods included: 11
Cross-sections included: 7
Total panel (unbalanced) observations: 54

| Variable | Coefficient | Std. Error | t-Statistic | Prob. |
|---|---|---|---|---|
| C | -12.29638 | 6.311631 | -1.948210 | 0.0583 |
| LBETHN | 1.186733 | 0.409763 | 2.896148 | 0.0060 |
| LCEOT | -0.032258 | 0.120743 | -0.267159 | 0.7907 |
| LCEOA | 3.001163 | 1.573674 | 1.907106 | 0.0635 |
| INO_2P | 0.319906 | 0.175335 | 1.824545 | 0.0754 |
| LBGDR | -0.334516 | 0.205117 | -1.630850 | 0.1106 |
| TM | -0.017987 | 0.013726 | -1.310497 | 0.1973 |

Effects Specification

Cross-section fixed (dummy variables)

| | | | |
|---|---|---|---|
| R-squared | 0.684787 | Mean dependent var | 0.698073 |
| Adjusted R-squared | 0.592530 | S.D. dependent var | 0.715541 |
| S.E. of regression | 0.456754 | Akaike info criterion | 1.476725 |
| Sum squared resid | 8.553586 | Schwarz criterion | 1.955555 |
| Log likelihood | -26.87158 | Hannan-Quinn criter. | 1.661391 |
| F-statistic | 7.422574 | Durbin-Watson stat | 1.512566 |
| Prob(F-statistic) | 0.000001 | | |

Dependent Variable: LROE
Method: Panel Least Squares
Date: 01/11/18 Time: 15:24
Sample: 2006 2016
Periods included: 11
Cross-sections included: 7
Total panel (unbalanced) observations: 54

| Variable | Coefficient | Std. Error | t-Statistic | Prob. |
|---|---|---|---|---|
| C | -12.97480 | 7.069925 | -1.835211 | 0.0737 |
| LBETHN | 0.549537 | 0.388990 | 1.412727 | 0.1653 |
| LCEOT | -0.144960 | 0.131921 | -1.098842 | 0.2782 |
| LCEOA | 5.225970 | 2.077201 | 2.515871 | 0.0159 |
| INO_2P | 0.462299 | 0.198188 | 2.332636 | 0.0247 |
| LBGDR | -0.151589 | 0.240196 | -0.631105 | 0.5315 |
| LCEOR | -0.310865 | 0.134337 | -2.314067 | 0.0258 |

Effects Specification

Cross-section fixed (dummy variables)

| | | | |
|---|---|---|---|
| R-squared | 0.621011 | Mean dependent var | 2.688720 |
| Adjusted R-squared | 0.510087 | S.D. dependent var | 0.715272 |
| S.E. of regression | 0.500646 | Akaike info criterion | 1.660235 |
| Sum squared resid | 10.27650 | Schwarz criterion | 2.139064 |
| Log likelihood | -31.82633 | Hannan-Quinn criter. | 1.844900 |
| F-statistic | 5.598538 | Durbin-Watson stat | 1.258056 |
| Prob(F-statistic) | 0.000015 | | |

## APPENDIX "H": DISAGGREGATED ANALYSIS – INTERNAL GOVERNANCE CONTROLS AND CORPORATE SOCIAL PERFORMANCE

### 1) BOARD STRUCTURE:

**Model Summary – Board Structure and Social Performance**

| Model | R | R Square | Adjusted R Square | Std. Error of the Estimate | F | Sig. |
|---|---|---|---|---|---|---|
| 1 | .358[a] | .129 | .118 | .39803 | 11.903 | .000[b] |

a. Predictors: (Constant), Strategy., Board size, Board meetings, Board independence, Board committees, Board composition, Diverse Board

**Coefficients[a] - Board Structure and Social Performance**

| Model | | Unstandardized Coefficients | | Standardized Coefficients | t | Sig. |
|---|---|---|---|---|---|---|
| | | B | Std. Error | Beta | | |
| 1 | (Constant) | 2.789 | .151 | | 18.444 | .000 |
| | Board size | .098 | .019 | .222 | 5.176 | .000 |
| | Board Composition | .050 | .025 | .096 | 1.943 | .053 |
| | Board Independence | .050 | .029 | .079 | 1.683 | .093 |
| | Board meetings | -.005 | .023 | -.009 | -.204 | .838 |
| | Board committees | .056 | .035 | .079 | 1.592 | .112 |
| | Diverse Board | .038 | .033 | .060 | 1.151 | .250 |
| | Strategy. | .003 | .033 | .004 | .087 | .931 |

a. Dependent Variable: Social performance

**Model Summary - Board Structure and Environmental Performance**

| Model | R | R Square | Adjusted R Square | Std. Error of the Estimate | F | Sig. |
|---|---|---|---|---|---|---|
| 1 | .417[a] | .174 | .164 | .52597 | 16.998 | .000[b] |

a. Predictors: (Constant), Strategy., Board size, Board meetings, Board independence, Board committees, Board composition, Diverse Board

**Coefficients[a] - Board Structure and Environmental Performance**

| Model | | Unstandardized Coefficients | | Standardized Coefficients | t | Sig. |
|---|---|---|---|---|---|---|
| | | B | Std. Error | Beta | | |
| 1 | (Constant) | 2.018 | .200 | | 10.097 | .000 |
| | Board size | .142 | .025 | .239 | 5.711 | .000 |
| | Board Composition | .029 | .034 | .042 | .866 | .387 |
| | Board Independence | .024 | .039 | .028 | .621 | .535 |
| | Board meetings | -.002 | .030 | -.004 | -.083 | .934 |
| | Board committees | .225 | .046 | .235 | 4.883 | .000 |
| | Diverse Board | .057 | .043 | .067 | 1.309 | .191 |
| | Strategy. | .005 | .044 | .005 | .104 | .917 |

a. Dependent Variable: Corporate Social Performance - Environmental

### 2) MANAGEMENT STRUCTURE:

**Model Summary - Management Structure and Social Performance**

| Model | R | R Square | Adjusted R Square | Std. Error of the Estimate | F | Sig. |
|---|---|---|---|---|---|---|
| 1 | .390[a] | .152 | .143 | .39231 | 16.896 | .000[b] |

a. Predictors: (Constant), Incentive-based compensation, CEO's diversity, internal CEO promotion, CEO's reputation, Tenure , Remuneration

**Coefficients[a] - Management Structure and Social Performance**

| Model | | Unstandardized Coefficients | | Standardized Coefficients | t | Sig. |
|---|---|---|---|---|---|---|
| | | B | Std. Error | Beta | | |
| 1 | (Constant) | 2.962 | .107 | | 27.625 | .000 |
| | Internal CEO Promotion | .005 | .023 | .010 | .216 | .829 |
| | Tenure | .117 | .023 | .239 | 5.006 | .000 |
| | Remuneration | .058 | .025 | .112 | 2.297 | .022 |
| | CEO's diversity | .026 | .021 | .056 | 1.196 | .232 |
| | CEO's reputation | .008 | .025 | .014 | .301 | .763 |
| | Incentive-based compensation | .049 | .024 | .097 | 2.026 | .043 |

a. Dependent Variable: Social performance

**Model Summary - Management Structure and Environmental Performance**

| Model | R | R Square | Adjusted R Square | Std. Error of the Estimate | F | Sig. |
|---|---|---|---|---|---|---|
| 1 | .414[a] | .172 | .163 | .52624 | 19.545 | .000[b] |

a. Predictors: (Constant), Incentive-based compensation, CEO's diversity, internal CEO promotion, CEO's reputation, Tenure , Remuneration

**Coefficients[a] - Management Structure and Environmental Performance**

| Model | | Unstandardized Coefficients | | Standardized Coefficients | t | Sig. |
|---|---|---|---|---|---|---|
| | | B | Std. Error | Beta | | |
| 1 | (Constant) | 2.437 | .144 | | 16.945 | .000 |
| | Internal CEO Promotion | .058 | .031 | .088 | 1.839 | .066 |
| | Tenure | .075 | .031 | .113 | 2.396 | .017 |
| | Remuneration | .036 | .034 | .051 | 1.055 | .292 |
| | CEO's diversity | .047 | .029 | .075 | 1.644 | .101 |
| | CEO's reputation | .037 | .034 | .049 | 1.078 | .282 |
| | Incentive-based compensation | .142 | .032 | .208 | 4.400 | .000 |

a. Dependent Variable: Corporate Social Performance - Environmental

### 3) OWNERSHIP STRUCTURE:

**Model Summary - Ownership Structure and Social Performance**

| Model | R | R Square | Adjusted R Square | Std. Error of the Estimate | F | Sig. |
|---|---|---|---|---|---|---|
| 1 | .209[a] | .044 | .035 | .41623 | 5.174 | .000[b] |

a. Predictors: (Constant), Employees' share, Concentrated ownership, Institutional investors, Diffused ownership, Directors' share ownership

**Coefficients[a] - Ownership Structure and Social Performance**

| Model | | Unstandardized Coefficients | | Standardized Coefficients | t | Sig. |
|---|---|---|---|---|---|---|
| | | B | Std. Error | Beta | | |
| 1 | (Constant) | 3.223 | .133 | | 24.269 | .000 |
| | Institutional investors | .045 | .031 | .074 | 1.469 | .142 |
| | Directors' share ownership | .023 | .030 | .039 | .764 | .445 |
| | Diffused ownership | .008 | .021 | .017 | .371 | .711 |
| | Concentrated ownership | .039 | .024 | .073 | 1.652 | .099 |
| | Employees' share ownership | .058 | .023 | .110 | 2.475 | .014 |

a. Dependent Variable: Social performance

**Model Summary - Ownership Structure and Environmental Performance**

| Model | R | R Square | Adjusted R Square | Std. Error of the Estimate | F | Sig. |
|---|---|---|---|---|---|---|
| 1 | .332[a] | .110 | .102 | .54499 | 14.016 | .000[b] |

a. Predictors: (Constant), Employees' share, Concentrated ownership, Institutional investors, Diffused ownership, Directors' share ownership

**Coefficients[a] - Ownership Structure and Environmental Performance**

| Model | | Unstandardized Coefficients | | Standardized Coefficients | t | Sig. |
|---|---|---|---|---|---|---|
| | | B | Std. Error | Beta | | |
| 1 | (Constant) | 2.500 | .174 | | 14.375 | .000 |
| | Institutional investors | .066 | .040 | .079 | 1.633 | .103 |
| | Directors' share ownership | .142 | .039 | .179 | 3.653 | .000 |
| | Diffused ownership | -.015 | .027 | -.024 | -.561 | .575 |
| | Concentrated ownership | .112 | .031 | .153 | 3.609 | .000 |
| | Employees' share ownership | .052 | .031 | .073 | 1.698 | .090 |

a. Dependent Variable: Corporate Social Performance - Environmental

**APPENDIX "H" *Contd.***

**Summary of Significance Tests on Board Structure and Social & Environmental Performance**

| | Social Performance | Environmental Performance |
|---|---|---|
| **BOARD STRUCTURE** | | |
| Board size | +*** | +*** |
| Board Composition | +* | |
| Board Independence | +* | |
| Board meetings | | |
| Board committees | | +*** |
| Diverse Board | | |
| Strategy. | | |

**Summary of Significance Tests on Management Structure and Social & Environmental Performance**

| **MANAGEMENT STRUCTURE** | Social Performance | Environmental Performance |
|---|---|---|
| Internal CEO Promotion | | +* |
| Tenure | **+*** ** | **+** ** |
| Remuneration | +** | |
| CEO's diversity | | |
| CEO's reputation | | |
| Incentive-based compensation | +** | +*** |

**Summary of Significance Tests on Ownership Structure and Social & Environmental Performance**

| **OWNERSHIP STRUCTURE** | Social Performance | Environmental Performance |
|---|---|---|
| Institutional investors | | |
| Directors' share ownership | | +*** |
| Diffused ownership | | |
| Concentrated ownership | +* | +*** |
| Employees' share ownership | +** | +* |
