# Supplementary material for: Dataset on ethical leadership and corporate reputation – Nigerian deposit money banks׳ perspective
Source: Data Brief. 2018 May 23;19:847–52. doi: 10.1016/j.dib.2018.05.094 (PMC5997921; doi:10.1016/j.dib.2018.05.094)
Supplement: Supplementary file 4 — Supplementary material [file mmc4.docx]

**Questionnaire –** **Survey on Ethical Leadership and Corporate Reputation** **of Deposit Money Banks in Nigeria**


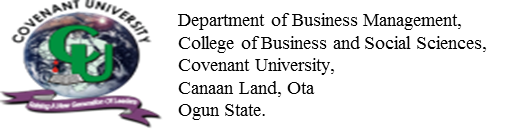


|  |  |
| --- | --- |

**SECTION A: DEMOGRAPHIC DATA (Please tick √ whichever is applicable)**

1. **Gender:** Male ( ) Female ( )
2. **Age Grade**: 18-24 ( ) 25-34 ( ) 35-44 ( ) 45-54 ( ) Above 54 ( )
3. **Marital Status**: Single ( ) Married ( ) Divorced ( ) Separated ( )
4. **Highest Education Degree**: Bachelors ( ) Masters ( ) Doctorate ( ) Others Pls. specify……….
5. **Job Function**: Business Development ( ) Operations and Technology ( ) Risk Management ( ) Corporate Development ( ) Others Pls. specify …….................
6. **Job Position**: Trainee ( ) Banking Officer ( ) Management ( )Senior Management ( )

Executive Management ( )

1. **Years Spent in the Bank**: Below 3yrs ( ) 3-5yrs ( ) 6-10yrs ( ) Above 10yrs ( )
2. **Bank’s Name:** ………………………………………………..

**SECTION B: STUDY SPECIFIC DATA**

Please tick √ the appropriate answer from the alternatives given as they best describe your opinion:

**Strongly Agree (SA) Agree (A) Neutral (N) Disagree (D) Strongly Disagree (SD)**

| **E** | **ETHICAL LEADERSHIP** | **SA** | **A** | **N** | **D** | **SD** |
| --- | --- | --- | --- | --- | --- | --- |
| 1 | In my bank, employees are never asked to do things that conﬂict with their moral principles. |  |  |  |  |  |
| 2 | The CEO is interested in how employees feel and perform |  |  |  |  |  |
| 3 | The CEO rewards performance in a fair manner. |  |  |  |  |  |
| 4 | The bank makes it clear to employees how to deal with conflicts of interests |  |  |  |  |  |
| 5 | The CEO enforces the long-term bank success against short-term wins. |  |  |  |  |  |
| 6 | The CEO can be trusted to do the things he/she says. |  |  |  |  |  |
| 7 | Unethical conduct reported is disciplined fairly. |  |  |  |  |  |
| 8 | The top management sets a good example in terms of ethical behaviour. |  |  |  |  |  |
| 9 | Employees with integrity stand a greater chance to receive a positive performance appraisal. |  |  |  |  |  |
| 10 | The bank has documented codes and practices of ethics known officially to all staff |  |  |  |  |  |
| 11 | A designated ethical officer monitors and reports on staff unethical behaviours |  |  |  |  |  |
| 12 | My bank promotes ethical awareness through training programmes for staff |  |  |  |  |  |
| 13 | The bank makes it clear to employees how to obtain proper authorisations |  |  |  |  |  |
|  |  |  |  |  |  |  |
| **F** | **CORPORATE REPUTATION** | **SA** | **A** | **N** | **D** | **SD** |
| 14 | My bank is highly regarded for the quality of its products and services |  |  |  |  |  |
| 15 | My bank has strong record of financial soundness |  |  |  |  |  |
| 16 | My bank has global competitiveness |  |  |  |  |  |

Thank you for your co-operation in completing this questionnaire.
